# From Plastic Waste to Pharmaceutical Precursors: PET Upcycling Through Ruthenium Catalyzed Semi‐Hydrogenation

**DOI:** 10.1002/anie.202521838

**Published:** 2025-12-18

**Authors:** Pavel S. Kulyabin, James Luk, Evgeny A. Uslamin, Alexander A. Kolganov, Garima Saini, Raymundo Marcial‐Hernandez, Ketan Pancholi, Benjamin Kühne, Alexander Dauth, Aidan P. McKay, David B. Cordes, Evgeny A. Pidko, Amit Kumar

**Affiliations:** ^1^ EaStCHEM School of Chemistry University of St Andrews North Haugh, St Andrews KY16 9ST UK; ^2^ Inorganic Systems Engineering group, Department of Chemical Engineering, Faculty of Applied Sciences Delft University of Technology Van der Maasweg 9 Delft, HZ 2629 The Netherlands; ^3^ The Sir Ian Wood Building Robert Gordon University Garthee Rd Garthee Aberdeen AB10 7GE UK; ^4^ Corporate Sustainability Merck Group Frankfurter Straße 250 Darmstadt 64293 Germany

**Keywords:** Hydrogenation, PET, Pincer, Recycling, Ruthenium

## Abstract

We report here the upcycling of PET (polyethylene terephthalate) waste via semihydrogenation to make ethyl 4‐(hydroxymethyl)benzoate. The reaction is catalyzed by a ruthenium pincer catalyst at 80 °C in bioderived solvents – a combination of 2‐methyl THF and ethanol. A detailed mechanistic investigation through organometallic and kinetic studies, as well as chemical exchange saturation transfer (CEST) NMR spectroscopy, provides insights into the nature of active species and factors that promote and inhibit the catalytic hydrogenation of PET. Using this mechanistic knowledge, a record high turnover number of >30 000 was achieved for the hydrogenative depolymerization of end‐of‐life PET waste (e.g., bottles and textiles). The semihydrogenation product, ethyl 4‐(hydroxymethyl)benzoate, was utilized to make precursors of various known pharmaceutical drugs, an agrochemical, as well as a new and recyclable polyester. A cradle‐to‐gate life cycle assessment demonstrated that using PET waste as a feedstock for EHMB production significantly reduces the environmental footprint compared to the conventional route from *p*‐toluic acid.

## Graphical abstract

We present a new catalytic process that converts real‐life PET waste into ethyl 4‐hydroxymethylbenzoate (EHMB), which serves as a valuable intermediate for the synthesis of pharmaceuticals, an insecticide, and a novel polyester.

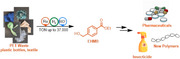

## Introduction

PET (Polyethylene terephthalate) is the most used polyester plastic with a global production of more than 80 million tons annually.^[^ ^1^ ^]^ Although a large percentage of PET is currently recycled,^[^ ^2^ ^]^ most of it is “downcycling” as it is conducted by mechanical processing that produces relatively poor‐quality plastics.^[^ ^3^ ^]^ The current state‐of‐the‐art chemical recycling technologies are based on glycolysis and hydrolysis, which can depolymerize PET to make ethylene glycol and terephthalic acid or corresponding esters, which can be used to make virgin PET.^[^ ^4^, ^5^, ^6^ ^]^ Although these processes are useful for circularity, the high energy needed for depolymerization can make the processes expensive, especially for making feedstock to make virgin PET. An alternative approach to tackle these challenges will be to upcycle PET waste to higher‐value products such as feedstock or intermediates for pharmaceuticals, agrochemicals, and other higher‐value polymers. Pharmaceuticals are particularly important as this industry has a substantial carbon footprint, generating more than 4.5% of global greenhouse gas emissions.^[^ ^7^ ^]^ In terms of manufacturing, the pharmaceutical industry produces the maximum amount of waste per kilogram of product (E‐factor = 25–100) in comparison to other chemical industries, such as the bulk chemical, fine chemical, or oil refining industry.^[^ ^8^ ^]^ The atom‐economic valorization of plastic waste, e.g. PET to pharmaceutical intermediates, will allow us to reduce the carbon footprint of the pharmaceutical industry. At the same time, it will allow upcycling of plastic waste rather than downcycling.^[^ ^9^ ^]^ Upcycling PET waste to make feedstock for pharmaceutical drugs (e.g., vanillin, gallic acid, and vanillic acid) has only been studied a few times using enzymatic catalysis in the past and needs more attention.^[^ ^10^, ^11^, ^12^ ^]^

**Figure 1:**
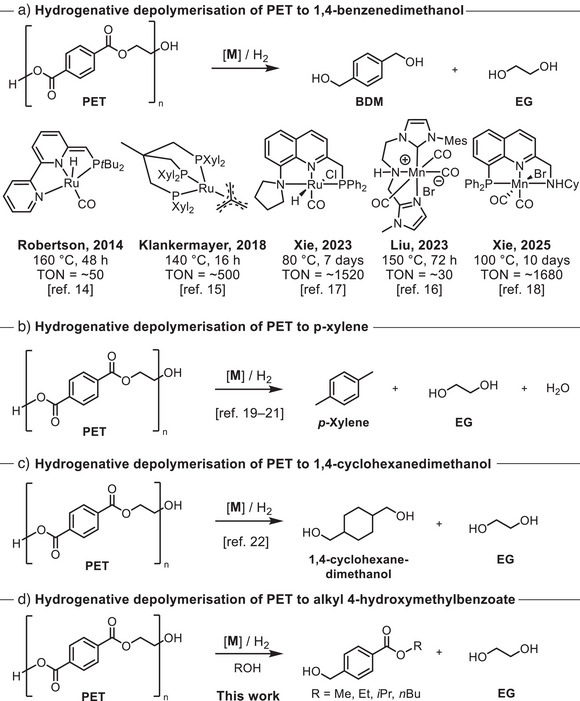
Hydrogenative depolymerization of PET to a) 1,4‐benzendimethanol, b) *p*‐xylene, c) 1,4‐cyclohexanedimethanol, and d) alkyl 4‐(hydroxymethyl)benzoates.

Catalytic hydrogenation is a green and atom‐economic approach in organic synthesis that has been utilized for the depolymerization of PET waste.^[^ ^13^ ^]^ For example, Robertson,^[^ ^14^ ^]^ Klankermayer,^[^ ^15^ ^]^ Liu,^[^ ^16^ ^]^ and Xie^[^ ^17^, ^18^ ^]^ have reported the use of ruthenium and manganese‐based homogeneous catalysts for the hydrogenative depolymerization of PET to make 1,4‐benzendimethanol (Figure 1a). Low‐to‐moderate turnover numbers (30–1680) of these homogeneous catalysts for making 1,4‐benzendimethanol present the main bottleneck in the large‐scale application of these processes. A few heterogeneous catalysts have been reported for the hydrogenative depolymerization of PET to make *p‐*xylene,^[^ ^19^, ^20^, ^21^ ^]^ 1,4‐cyclohexanedimethanol (Figure 1b and c),^[^ ^22^ ^]^ or *p‐*toluic acid.^[^ ^23^, ^24^ ^]^ We envisioned that combining hydrogenation and transesterification could allow us to selectively obtain alkyl 4‐(hydroxymethyl)benzoate, which can be used as a potential feedstock to make various intermediates of therapeutic importance (Figure 1d).

## Results and Discussion

We started our investigation by studying the hydrogenative depolymerization of PET (1 mmol) using Gusev's ruthenium‐based complex **1**, which has been reported to show high TON for the catalytic hydrogenation of esters to alcohols.^[^ ^25^, ^26^ ^]^ The catalytic activity was studied at 80 °C, and 50 bar H_2_ pressure for 18 h by the variation of solvent and base (e.g., *tert*‐amyl alcohol, THF, or 2‐MeTHF in combination with alcohols such as ethanol or butanol, KO*t*Bu, NaO*t*Bu, or K_2_CO_3_ Tables S1, S3). Initial studies suggested the mixture of 2‐MeTHF and ethanol to be an optimum solvent and that premixing the precatalyst with base in a 2‐MeTHF/EtOH mixture is important for higher hydrogenation yield (Table S3, S5, and S6). 1,4‐Benzenedimethanol (BDM) was obtained as the major product when 0.1–1.0 mol% loading of precatalyst **1** was used (Table S1–S4), whereas, interestingly, when lower catalytic loading (e.g., 0.05 mol%) was used, the formation of a semihydrogenation product – ethyl 4‐(hydroxymethyl)benzoate (EHMB) was observed along with the transesterification product – diethyl terephthalate (DETP) (Table S5, S6). For example, using 0.05 mol% of precatalyst **1** and 5 mol% of KO*t*Bu led to the formation of 46% of EHMB along with 51% of BDM from the hydrogenation of commercial PET powder at 80 °C, 50 bar H_2_ for 18 h (Table 1, entry 1). Motivated by this, we studied other catalysts that have been reported to exhibit high activity for the hydrogenation of esters to alcohols. The use of ruthenium complexes **2** ^[^ ^27^ ^]^ and **3**.^[^ ^28^ ^]^ led to a slightly lower yield of EHMB (24, and 26%, respectively; entries 2, 3), whereas the precatalysts **4**,^[^ ^28^ ^]^ **5**.^[^ ^29^ ^]^ and **6**.^[^ ^30^ ^]^ led to higher yields of EHMB (63%, and 64%, respectively; entries 4–6) but lower yields of BDM. Using an iridium pincer catalyst **7** ^[^ ^31^, ^32^ ^]^ did not lead to the formation of any EHMB under identical reaction conditions (entry 7). Increasing substrate concentration 2‐fold while keeping the same catalytic loading had a notable effect on the selectivity and produced more BDM than EHMB (75% and 22% respectively, entry 8). Performing the reaction for a longer time (67 h) led to a higher yield of BDM (88%, entry 9), which was found to be even higher when the reaction was conducted using waste plastic bottles (95%, entry 10). These optimization studies suggest that precatalyst **1** was the most active one for the hydrogenation of the ester group, as the formation of BDM requires the hydrogenation of two ester groups in DETP. We envisioned that this catalyst could be made more selective toward semihydrogenation if the reaction was conducted at lower catalytic loading. Indeed, lowering the catalytic loading to 0.01 mol% led to the formation of EHMB in 84% yield (entry 11) along with 11% BDM and 3% DETP.

**Table 1:** Hydrogenative depolymerization of PET.^a)^

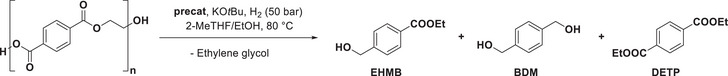							
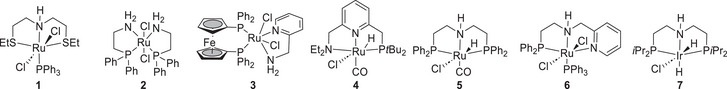							
Entry	PET	KO*t*Bu mol%	Precat	Time, h	EHMB, %	BDM, %	DETP, %
1	1 mmol	5	0.05% **1**	18	46	51	<1
2	1 mmol	5	0.05% **2**	18	24	<1	75
3	1 mmol	5	0.05% **3**	18	26	<1	71
4	1 mmol	5	0.05% **4**	18	63	2	32
5	1 mmol	5	0.05% **5**	18	64	2	33
6	1 mmol	5	0.05% **6**	18	76	14	7
7	1 mmol	5	0.05% **7**	18	<1	<1	98
8	2 mmol	5	0.05% **1**	18	22	75	<1
9	2 mmol	5	0.05% **1**	67	11	88	<1
10^b)^	2 mmol	5	0.05% **1**	67	2	95	<1
11	2 mmol	5	0.01% **1**	18	84	11	3

^a)^ Polyethylene terephthalate powder (1 or 2 mmol of monomeric unit), 4.5 mL 2‐MeTHF and 0.5 EtOH solvent, KO*t*Bu (1 M solution in THF). The precatalyst and KO*t*Bu were premixed in 2‐MeTHF and EtOH solution. Yields were estimated by ^1^H NMR spectroscopy using mesitylene as an internal standard. For the detailed information see Tables S7–S9.

^b)^ PET from used plastic bottles.

**Scheme 1:**
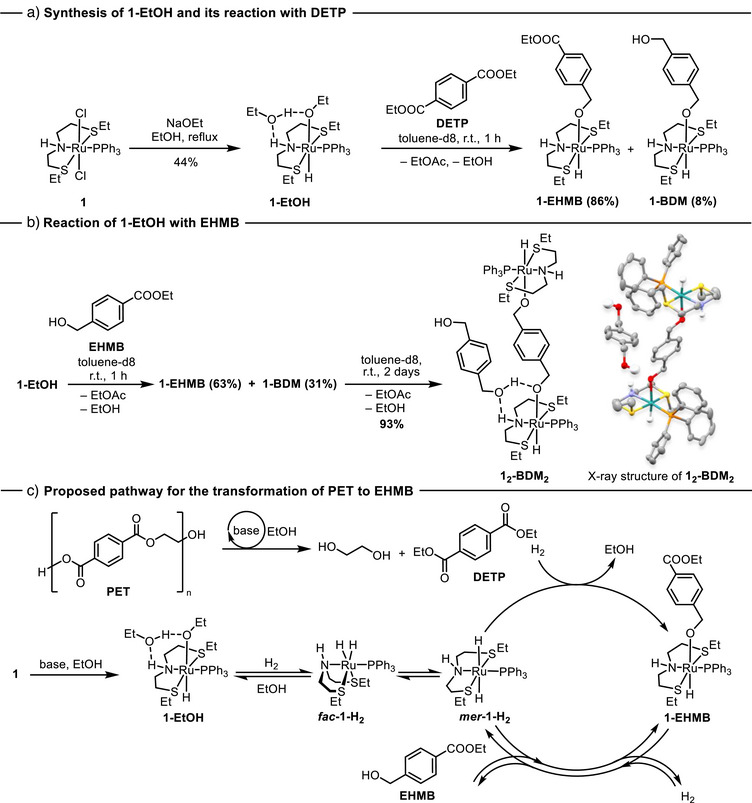
a) Synthesis of **1‐EtOH** from **1** and its reaction with DETP. EHMB – ethyl 4‐(hydroxymethyl)benzoate. b) Reaction of **1‐EtOH** with **EHMB**. DETP – diethyl terephthalate, r.t. – room temperature. c) Proposed general pathway for the transformation of PET to EHMB.

The TON of 10600 (based on the number of ester groups hydrogenated) obtained in entry 11, Table 1 is higher than those of previous reports on the hydrogenative depolymerization of PET.^[^ ^15^, ^17^ ^]^ However, we believed that through a detailed insight into the nature of active species, as well as factors promoting and inhibiting the catalytic activity, even greater TONs could be achieved. Adopting the method from Gusev's original work,^[^ ^25^ ^]^ we first synthesized the ethoxide complex **1‐EtOH** through the reaction of complex **1** with NaOEt in ethanol (Scheme 1a). Evaluating it in the catalytic hydrogenation of PET revealed that complex **1‐EtOH** produced a similar product distribution as that of complex **1** in 1 h in the presence of KO*t*Bu (Table 2, entries 1, 2). However, when **1‐EtOH** was used in the absence of KO*t*Bu, keeping the remaining conditions the same, negligible conversion of PET was obtained, presumably as KO*t*Bu is needed for the depolymerization via ethanolysis (entry 3). Indeed, performing the reaction just in the presence of KO*t*Bu without using any ruthenium complex led to the formation of DETP in 92% yield in 1 h, whereas the formation of EHMB and BDM was not observed (entry 4). The same result was observed when the reaction was carried out without hydrogen pressure, demonstrating that catalytic transfer hydrogenation using ethanol does not happen under these conditions (entry 5). These experiments also confirm that a catalytic loading of KO*t*Bu (e.g., 5 mol%) is sufficient to perform the ethanolysis of PET in 1 h at 80 °C.

**Table 2:** Hydrogenative depolymerization of PET using complex 1 and 1‐EtOH.^a)^

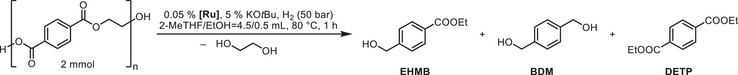					
Entry	[Ru]	KO*t*Bu, %	EHMB, %	BDM, %	DETP, %
1	**1**	5	72	10	4
2	**1‐EtOH**	5	65	12	5
3	**1‐EtOH**	none	1	1	n.d.
4	none	5	n.d.	n.d.	92
5^b)^	**1‐EtOH**	5	n.d.	n.d.	96

^a)^ Polyethylene terephthalate powder (2 mmol of monomeric unit), 4.5 mL 2‐MeTHF and 0.5 mL ethanol, 5% of KO*t*Bu (0.1 mL, 1 M solution in THF), 0.05 mol% of **1** or **1‐EtOH**, and 50 bar of H_2_. The yields were estimated by ^1^H NMR spectroscopy using mesitylene as an internal standard. For the detailed information see Table S12, n.d. – not detected.

^b)^ Reaction was run in 10 mL Young's flask under argon without any hydrogen pressure.

Having **1‐EtOH** in hand, we studied its reactions with diethyl terephthalate (DETP) and ethyl 4‐(hydroxymethyl)benzoate (EHMB). Our study showed that complex **1‐EtOH** reacted with 1 equiv. of DETP (in toluene‐*d_8_*) at room temperature in 1 h and formed a mixture of **1‐EHMB** and **1‐BDM** in 86% and 8% yields, respectively (Scheme 1a). This reaction could be referred to as transfer hydrogenation under stoichiometric conditions, and it indeed generated a stoichiometric amount of ethyl acetate as a by‐product, as confirmed by ^1^H NMR spectroscopy. The experiment also confirms that **1‐EtOH** is capable of dehydrogenating ethanol to form a ruthenium dihydride complex, which reacts with DETP or EHMB even at room temperature. This is in line with Gusev's report, where complex **1‐EtOH** was found to convert to ***fac*‐1‐H_2_** upon mild heating.^[^ ^25^ ^]^

When **1‐EtOH** was mixed with EHMB in toluene‐*d8*, it predominantly led to the exchange of ligand forming **1‐EHMB** in 63% yield, and transfer hydrogenation product, **1‐BDM** in 31% yield (Scheme 1b). After two days at room temperature, yellow crystals suitable for X‐ray crystallography were isolated from the reaction mixture. Structural analysis revealed these crystals to be the bimetallic complex **1_2_‐BDM_2_**, a product of EHMB hydrogenation. These results indicate that EHMB undergoes hydrogenation notably slower than DETP, primarily because it preferentially binds to ruthenium via its alcohol group. We speculate that this binding preference is the key factor contributing to the observed selectivity for EHMB formation at low catalyst loadings (e.g., <0.05 mol%, also see, Table S11, Figures S107, S108).

Based on these, we propose a reaction pathway as outlined in Scheme 1c. We suggest that PET undergoes depolymerization via base‐catalyzed ethanolysis to form DETP. Simultaneously, complex **1** is transformed to a mixture of ***fac*‐1‐H_2_** and ***mer*‐1‐H_2_** through **1‐EtOH** in the presence of H_2_ and ethanol. Transfer of hydride from the ruthenium dihydride complex to DETP leads to the formation of complex **1‐EHMB** that can eliminate EHMB, regenerating the ruthenium dihydride species via metal‐ligand cooperation in the presence of base and H_2_.

**Figure 2:**
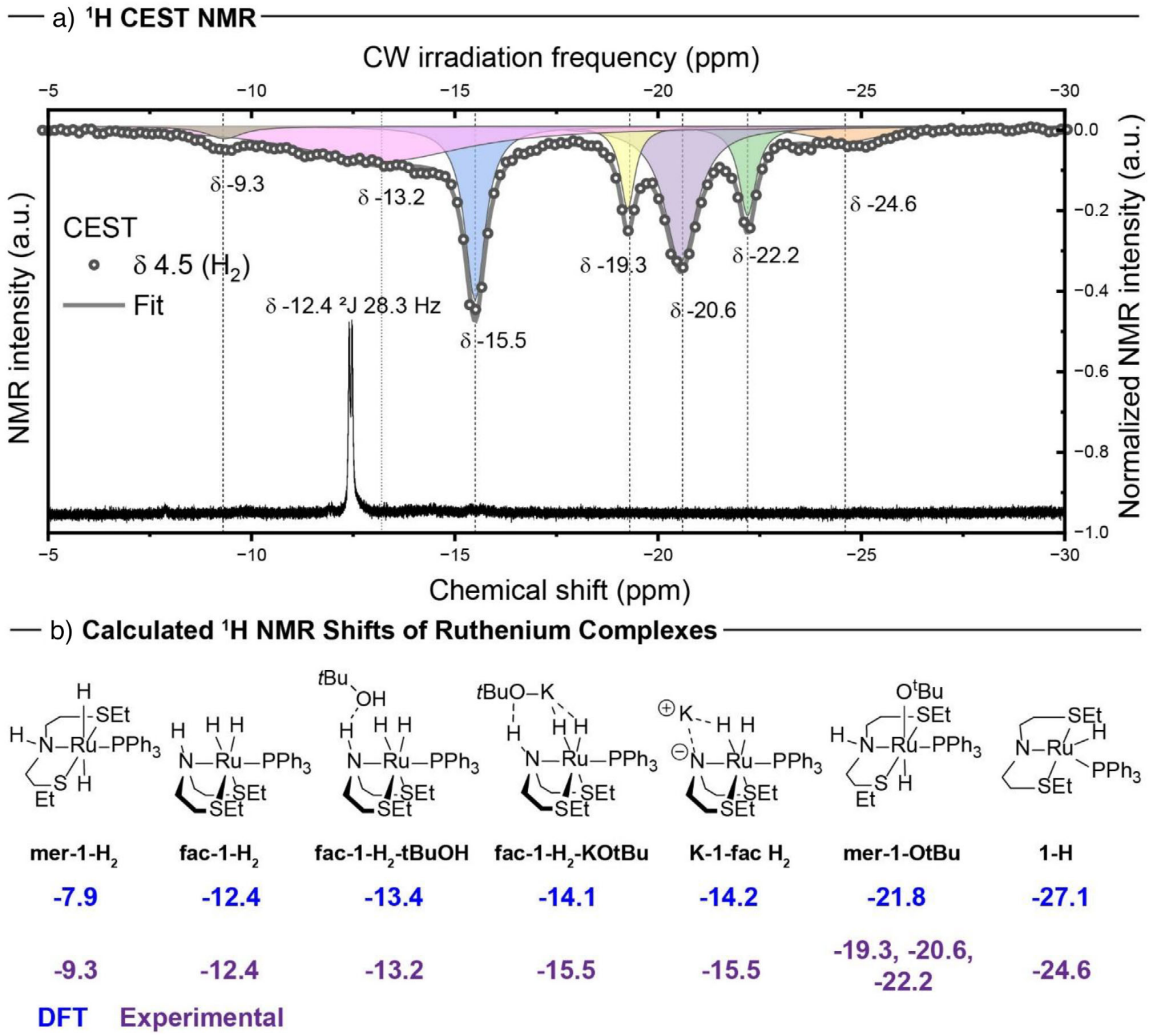
a) Representative ^1^H NMR (below) and ^1^H NMR CEST (above) spectra of solution from the reaction of complex **1** with 1.25 equiv. of KO*t*Bu and 5 bar H_2_ in THF‐*d_8_* at 298 K. b) Assignment of signals in ^1^H NMR CEST using DFT calculated chemical shifts from 4c‐DKS/PBE0/pcS‐2//PBE0‐D3(BJ)/def2‐TZVPP/SMD(THF) level of theory. Full details of DFT calculations are listed in Section 10 of the Supporting Information.

To get more insights into the nature of possible active species, we studied the reaction of complex **1** with KO*t*Bu in the presence of H_2_ at room temperature using chemical exchange saturation transfer (CEST) ^1^H NMR spectroscopy. This technique allows the observation of dynamic species inaccessible to a regular thermal ^1^H or ^31^P NMR measurements. The tool of CEST ^1^H NMR spectroscopy was recently utilized by Knecht et al.^[^ ^33^ ^]^ to observe short‐lived Ir‐NHC species via chemical hydride exchange of the species with free hydrogen gas. CEST NMR spectra in our case were recorded after the formation of dihyride complex ***fac*‐1‐H_2_** from the reaction of complex **1**, KO*t*Bu, and H_2_. The resulting CEST NMR spectra showed signals at –13.2, –15.5, –20.6 (together with –19.3 and –22.2 ppm), and –24.6 ppm that were assigned using computational modelling as the species ***fac*‐1‐H_2_‐*t*BuOH, *fac*‐1‐H_2_‐KO*t*Bu (or K‐1‐*fac*‐H_2_)**, ***mer*‐1‐O*t*Bu**, and **1‐H** (Figure 2). The result suggested that the dissolved hydrogen gas was observed to exchange mainly with the dynamic *fac*‐complexes interacting with the base or ***mer*‐1‐O*t*Bu**, and not the *fac*‐dihydride complex ***fac*‐1‐H_2_**. The absence of exchange might indicate lower reactivity and limited interconversion between more static **fac‐1‐H_2_** and the other species. This is consistent with the DFT study conducted by Chen et al. suggesting that ***fac*‐1‐H_2_** is likely to be a resting state whereas another isomer ***mer*‐1‐H_2,_** is the active species in the hydrogenation of esters.^[^ ^34^ ^]^ At the same time, CEST ^1^H NMR experiments are suggestive of the possibility of ***fac*‐1‐H_2_‐KO*t*Bu/K‐1‐*fac*‐H_2_** and ***mer*‐1‐O*t*Bu** (which is analogous to **1‐EtOH**) to participate in hydrogen splitting step, and that a base such as KO*t*Bu or KOEt could be important in the activation of H_2,_ as it was discussed previously.^[^ ^35^ ^]^

**Figure 3:**
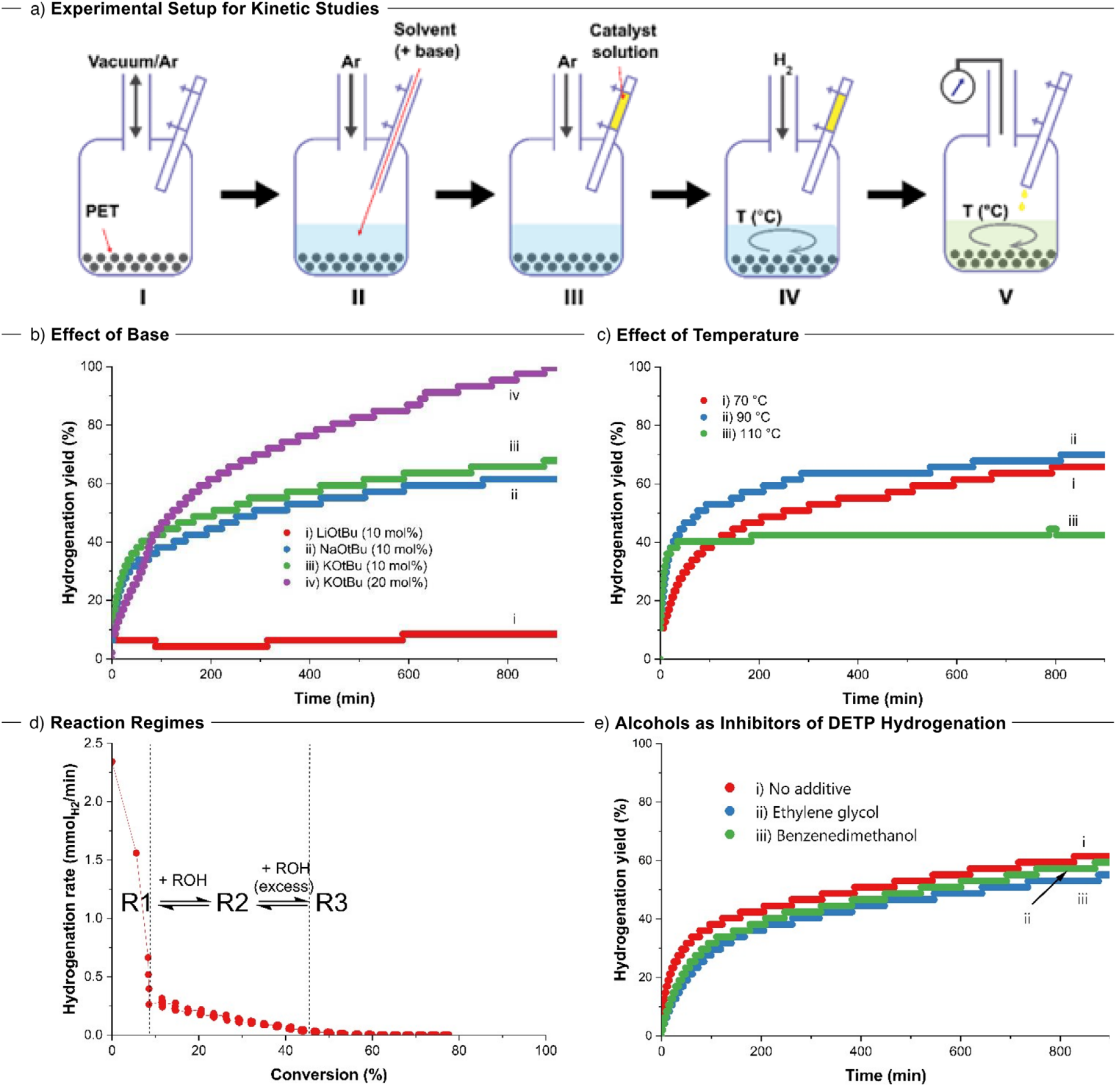
a) Experimental setup for kinetic studies, I) autoclave is vacuum cycled with argon, II) the desired solvent (and base) are added to the autoclave under the flow of argon, III) the catalyst solution is added to the chamber in the injection port under argon, IV) the chamber and reactor are both pressurized with H_2_ and thermostated, V) the autoclave is sealed, catalyst added, and the pressure monitored. b) Graph displaying the effect of the base on the hydrogenation of PET versus time (2 mmol PET, [0.05 mol% **1 **+ 0.063 mol% KO*t*Bu], 5 mL EtOH:2‐MeTHF (1:4), 80 °C, 50 bar H_2_). c) Graph displaying the effect of temperature on the hydrogenation of PET versus time (2 mmol PET, [0.05 mol% **1 **+ 0.063 mol% KO*t*Bu], 10 mol% KO*t*Bu, 5 mL EtOH:2‐MeTHF (1:4), 50 bar H_2_). d) Representative hydrogenation rate versus conversion graph of hydrogenation of PET displaying reaction regimes. e) Graph displaying hydrogenation of DETP (2 mmol DETP, [0.025 mol% **1 **+ 0.063 mol% KO*t*Bu], 5 mL EtOH:2‐MeTHF (1:4), 80 °C, and 50 bar H_2_) with no additive (red) or 30 mol% ethylene glycol (blue) or 30 mol% BDM (green).

Having gained insights into the nature of active species, we directed our efforts toward understanding the factors inhibiting or promoting the catalytic hydrogenation. To probe the effect of base, temperature, and alcohols on reaction kinetics, we performed a series of experiments by monitoring the consumption of pressure over time using the method shown in Figure 3a. The most notable observations occurred by altering the type of base and base concentration (Figure 3b). The use of LiO*t*Bu resulted in very low hydrogenation of ∼8%, almost all of which occurred in <30 min, followed by very little observable hydrogenation taking place. This contrasted with the results of both KO*t*Bu and NaO*t*Bu, which followed the expected kinetic profiles and afforded a moderate degree of hydrogenation (∼68%, ∼62%, respectively). Furthermore, doubling the concentration of KO*t*Bu (20 mol%) increased the degree of hydrogenation to 100% from 70% suggesting the positive influence of KO*t*Bu on hydrogenation. A similar trend was previously reported for the hydrogenation of small molecule esters using Firmenich's catalyst **2**.^[^ ^36^ ^]^ Varying the temperature revealed faster kinetics at 90 °C in comparison to that of 70 °C, although a similiar hydrogenation yield was obtained at the end of 15 h (70%, and 67%, respectively, Figure 3c). However, increasing the temperature to 110 °C shut down the catalysis in 35 min, suggesting that higher temperatures are detrimental to the active species.

The hydrogenation rate profiles of all activated catalyst experiments follow similar trends that can be separated into three regimes of differing reactivity (Figure 3d): **R1,** which has incredibly high activity but lasts only minutes; **R2**, which has much lower activity than **R1** and which steadily decreases lasting around 1–2 h; **R3** with extremely low activity but can last for days. We suggest that the reaction products, EHMB, EG, and BDM, being more acidic than ethanol and *tert*‐butyl alcohol, could inhibit catalyst activity, resulting in these various reaction regimes due to dynamic equilibria between the active/inactive catalyst species present in the reaction mixture. We propose that the predominant species in the **R1** regime are potassium complexes ***fac*‐1‐H_2_‐KO*t*Bu**, **K‐1‐*fac*‐H_2_**, ***mer*‐1‐*t*BuOH** (or ***mer*‐1‐EtOH**), and **1‐H** (Figure 2b). However, the concentration of these species, e.g., potassium complex, diminishes upon its interaction with the hydroxyl groups of reaction products (e.g., EHMB) present in the reaction mixture, resulting in **R2**. The ensuing slow decrease in reactivity observed culminating in **R3** is due to the increasing concentration of hydrogenation and alcoholysis products BDM, EHMB, and EG. It was also previously demonstrated that the buildup of alcohol product could inhibit the ester hydrogenation reaction in the case of the catalyst derived from complex **4,** which operates by a metal–ligand‐cooperation mechanism analogous to that proposed for Gusev's complex **1**.^[^ ^37^ ^]^ Indeed, the analysis of reaction mixture confirms that the regime **R3** (Figure 3d) starts when the concentration of EHMB reaches its maximum (Tables S11, Figure S107).

To test the hypothesis that EG (ethylene glycol), and BDM (1,4‐benzenedimethanol) can inhibit the catalytic hydrogenation of esters, kinetics of the hydrogenation of DETP (diethyl terephthalate) was conducted (as a simplified model of PET hydrogenation) in the presence of 30% ethylene glycol, 30% benzenedimethanol, and without using any additive (Figure 3e). The kinetic data revealed that the presence of ethylene glycol and benzendimethanol resulted in a lower overall yield and rate of hydrogenation than when no additive was present, confirming the inhibitory effect of these alcohols on the hydrogenation of esters (Figure 3e).

Having gained insights into factors promoting and inhibiting hydrogenation of PET, we performed further optimization studies aiming to increase the turnover number towards EHMB. First, we compared the performance of complexes **4**, **5**, and **6,** which had high selectivity toward EHMB (Table 1), with that of **1** at 0.01 mol% catalytic loading and found that **1** was still superior in terms of EHMB yield (Table S7). Increasing the time from 18 to 44 h in the case of 0.01 mol% **1** and 5 mol% KO*t*Bu did not lead to any change in conversion of PET or selectivity toward EHMB (Table 3, entries 1, and 2). Increasing the base loading to 10 mol% from 5 mol% showed a lower yield of EHMB (75%) but a slightly higher yield of BDM, suggesting higher overall hydrogenation of esters (entry 3). This is consistent with our kinetic studies, shown in Figure 3b, that an increase in the amount of KO*t*Bu can increase the overall hydrogenation yield. This suggested that using a higher amount of KO*t*Bu, we might be able to further push the turnover number of **1**. We therefore carried out some catalytic studies using lower catalytic loadings. Using 0.005 mol% **1** and 5 mol% KO*t*Bu led to 74% and 85% yields of EHMB in 18 and 45 h, respectively (entries 4 and 5). Increasing the base loading to 20 mol% increased the yield of EHMB to 82% in 18 h (entry 6). Further lowering the catalytic loading to 0.002 mol% and increasing the base loading to 40% and time to 72 h produced EHMB in 45% yield (entry 7, TON = 22 500). Further increasing the base loading to 60%, and 80% increased the yield of EHMB to 56% and 72%, respectively, making the TON 27 500 and 37 000 (entries 8 and 9).

**Table 3:** Hydrogenative depolymerization of PET with Complex 1.^a)^

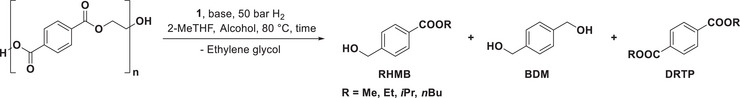								
Entry	KO*t*Bu, mol%	Alcohol	Precat, %	Time, h	RHMB, %	BDM, %	DRTP, %	TON^b)^
1	5	EtOH	0.01	18	84	11	3	10 600
2	5	EtOH	0.01	44	84	14	<1	11 200
3	10	EtOH	0.01	18	75	20	<1	11 500
4^c)^	5	EtOH	0.005	18	74	3	20	16 000
5^d)^	5	EtOH	0.005	45	85	8	5	20 200
6	20	EtOH	0.005	18	82	7	7	18 500
7^e)^	40	EtOH	0.002	72	45	<1	55	22 500
8^e)^	60	EtOH	0.002	72	55	<1	45	27 500
9^e)^	80	EtOH	0.002	72	74	<1	26	37 000
10	5	MeOH	0.01	18	79	3	17	8500
11	5	*i*PrOH	0.01	18	84	11	2	10 600
12	5	*n*BuOH	0.01	18	87	9	4	10 500
13	5	CF_3_CH_2_OH	0.01	18	<1	<1	4	–

^a)^ Polyethylene terephthalate powder (2 mmol of monomeric unit), 4.5 mL 2‐MeTHF and 0.5 mL alcohol solvent, 5, 10 or 20 mol% KO*t*Bu (1 M solution in THF), 0.005–0.01 mol% of **1**, and 50 bar of H_2_. The yields were estimated by ^1^H NMR spectroscopy using mesitylene as an internal standard. For the detailed information see Tables S9, S10.

^b)^ Estimated based on numbers of ester groups hydrogenated.

^c)^ Average results of 2 runs.

^d)^ Average results of 4 runs.

^e)^ Conditions: PET (2 mmol), 4 mL 2‐MeTHF, 1 mL EtOH, KO*t*Bu, 0.002 mol% of **1**, and 50 bar of H_2_.

Additionally, we envisioned that using our catalytic protocol, we could make other alkyl 4‐(hydroxymethyl)benzoates by using different alkyl alcohols instead of ethanol. Indeed, methanol, isopropanol, and *n*‐butanol led to the formation of corresponding alkyl 4‐(hydroxymethyl)benzoate in excellent yields (Table 3, entries 10–12). However, the use of trifluoroethanol did not lead to conversion of PET likely due to lower alcoholysis under these conditions (Table 3, entry 13).

Having optimized the synthesis of ethyl 4‐(hydroxymethyl)benzoate from polyethylene terephthalate, we studied the hydrogenative depolymerization of postconsumer plastic on a gram scale. Our optimization studies showed that KOEt is as effective as KO*t*Bu in the catalytic hydrogenation of PET under our conditions (see, Table S7 and S9). Indeed, using 0.005 mol% of complex **1**, and 5 mol% of KOEt or KO*t*Bu, 5 g of PET derived from colorless or green plastic bottles was hydrogenated to produce 84% or 80% yield of EHMB (TON = 20 000, or 20 800; Table 4, entries 1 and 2). Remarkably, decreasing catalytic loadings to 0.002 mol% (S/C = 50 000) and increasing the amount of base to 25 mol% led to the formation of EHMB in 60% yield, which corresponds to a TON of 32 000 (Table 4, entry 3), the highest ever achieved number for practical hydrogenation of postconsumer polyethylene terephthalate. PET textile derived from a white hairband and green ribbon was also hydrogenated to produce EHMB in yields >80% (TON = 19 800 and 23 600, entries 4 and 5). Blue fleece jacket required double the amount of base (10 mol% KO*t*Bu) and precatalyst (0.01 mol% **1**) loadings compared to our standard conditions and gave EHMB and BDM in 86% and 7% yield, respectively (TON = 10 000, entry 6).

**Table 4:** Gram scale hydrogenative depolymerization of PET using complex 1.^a)^

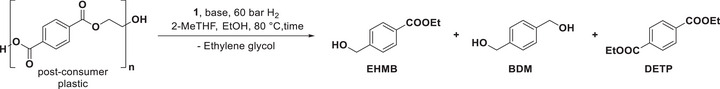											
Entry	PET source, mass		1, mol%	Base, mol%	2‐MeTHF, mL	EtOH, mL	Time, h	EHMB, %	BDM, %	DETP, %	TON^b)^
1	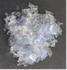 Colourless bottle	5 g	0.005	KOEt, 5	46.8	5.2	72	84 (72)^c)^	8	1	20 000
2	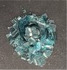 Green bottle	5 g	0.005	KO*t*Bu, 5	46.8	5.2	48	80	12	1	20 800
3	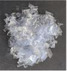 Colourless bottle	5 g	0.002	KO*t*Bu, 25	30	3.3	72	60	2	33	32 000
4	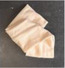 White hairband	1.6 g	0.005	KO*t*Bu, 5	45	1.5	48	83	8	4	19 800
5	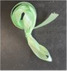 Green ribbon	5 g	0.005	KO*t*Bu, 5	45	5	48	82	18	<1	23 600
6	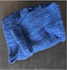 Blue fleece jacket	1.6 g	0.01	KO*t*Bu, 10	45	5	48	86	7	6	10 000

^a)^ Post‐consumer polyethylene terephthalate (considered as 100% polyester) was suspended in 2‐MeTHF and EtOH solvent. 5 or 25 mol% of base was added, and after 5 min of stirring, 0.002 or 0.01 mol% of **1** was added. The autoclave (150 mL volume) was purged with H_2_ and then pressure of 60 bar of H_2_ was applied. The reaction was heated at 80 °C at 750 rpm stirring with magnetic bar. The yields were estimated by ^1^H NMR spectroscopy using dichloromethane as an internal standard. For the detailed information see Table S14.

^b)^ Estimated based on numbers of ester groups hydrogenated.

^c)^ Isolated yield of 4‐(hydroxymethyl)benzoic acid of 96% purity with the other component being terephthalic acid. Along with HMBA, EG was also isolated with 67% yield.

### Application of Ethyl 4‐(hydroxymethyl)benzoate for Making Pharmaceuticals and Agrochemicals

**Scheme 2:**
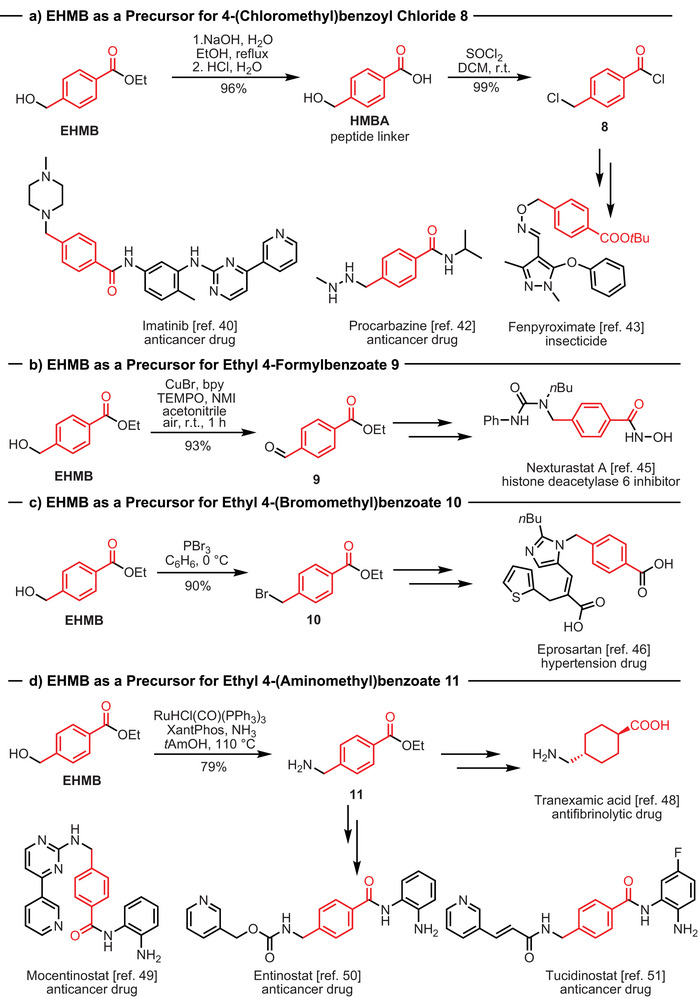
Application of EHMB for the synthesis of intermediates for making various known pharmaceuticals and agrochemicals. DCM – dichloromethane, bpy – 2,2′‐bipyridine, TEMPO – (2,2,6,6‐tetramethylpiperidin‐1‐yl)oxidanyl, NMI – N‐methylimidazole, XantPhos – (9,9‐Dimethyl‐9H‐xanthene‐4,5‐diyl)bis(diphenylphosphane).

We envisioned that ethyl 4‐(hydroxymethyl)benzoate (EHMB) could be used to make feedstock/precursors for therapeutics and agrochemicals. For example, several histone deacetylase inhibitors contain fragments that could be produced from EHMB, making it a valuable feedstock for various generic anticancer drugs.^[^ ^38^ ^]^ With this motivation, we studied further reactivity of EHMB to develop new routes to make molecules of commercial interest. Hydrolysis of EHMB led to the formation of 4‐(hydroxymethyl)benzoic acid (HMBA) in 96% yield (Scheme 2a) which is also a product of commercial interest and is sold for the application of a linker in automated peptide synthesis.^[^ ^39^ ^]^ HMBA can then be converted to 4‐(chloromethyl)benzoyl chloride **8** via reaction with thionyl chloride in a quantitative yield (Scheme 2a). It is noteworthy that compound **8** is a precursor for the synthesis of the blockbuster anticancer drug Imatinib^[^ ^40^ ^]^ (originally launched by Novartis, ∼$4.6 B sales in 2015),^[^ ^41^ ^]^ a chemotherapy drug procarbazine^[^ ^42^ ^]^ (1 month's cost ∼£450–750 in NHS UK, $82.36 million sales in 2023) and an insecticide fenpyroximate^[^ ^43^ ^]^ (estimated $500 million sales in 2023).

Furthermore, EHMB can be easily oxidized to ethyl 4‐formylbenzoate **9** ^[^ ^44^ ^]^ in 93% yield, which is used for the synthesis of the potent and selective histone deacetylase 6 inhibitor Nexturastat A (Scheme 2b).^[^ ^45^ ^]^ Substitution of the hydroxy group in EHMB to the bromide group can be achieved using PBr_3,_ producing ethyl 4‐(bromomethyl)benzoate **10** in 90% yield (Scheme 2c), which is used as a starting material for the synthesis of the antihypertension drug Eprosartan.^[^ ^46^ ^]^ Another useful modification of EHMB is its transformation to ethyl 4‐(aminomethyl)benzoate **11,** which was achieved in 79% yield using a ruthenium‐catalyzed amination reaction reported by Beller (Scheme 2d).^[^ ^47^ ^]^ Compound **11** is used as a precursor to make the antifibrinolytic drug tranexamic acid^[^ ^48^ ^]^ (estimated $1.01 B sales in 2024) as well as other anticancer drugs – mocetinostat,^[^ ^49^ ^]^ entinostat,^[^ ^50^ ^]^ and tucidinostat^[^ ^51^ ^]^ (∼$3000 for 0.5 g). These examples clearly demonstrate the diverse applications of EHMB in the production of high‐value products.

### Application of Ethyl 4‐(Hydroxymethyl)benzoate for Making a New Polyester

**Scheme 3:**
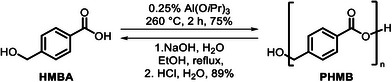
Synthesis and recycling of poly(4‐(hydroxymethyl)benzoate) (PHMB).

Although upcycling PET waste to make high‐value pharmaceuticals can be useful to the circular economy of the pharmaceutical industry, it is worth noting that the supply of PET waste is significantly higher than the demand for pharmaceuticals. We envisioned that 4‐(hydroxymethyl)benzoic acid (HMBA) derived from EHMB could also be used as a monomer for a new polyester, which could have a larger market size. To the best of our knowledge, there is no report on the synthesis of a polyester from HMBA. Interestingly, polycondensation of HMBA at 260 °C with 0.25 mol% aluminium isopropoxide as a catalyst without using any solvent under vacuum led to the formation of a yellow glassy material in 74% yield (Scheme 3). The obtained solid was found to be highly chemically resistant and not soluble in toluene, CHCl_3_, THF, ethanol, methanol, water, DMF, DMSO, which precluded the analysis of this material through gel permeation chromatography. Nevertheless, IR spectroscopy as well as ^1^H and ^13^C{^1^H} NMR spectra in deuterated trifluoroacetic acid showed the presence of ester, aromatic, and CH_2_ groups, confirming the polymer to be poly(4‐(hydroxymethyl)benzoate) (PHMB). End group analysis via ^1^H NMR spectrum gave an estimated degree of polymerization *n* = 14, which corresponds to *M*_n_ = 1,895 g mol^−1^. It is possible that this number could have been affected by polyester degradation in the acidic medium of deuterated trifluoroacetic acid, and the actual degree of polymerization and *M*_n_ could be higher. Thermogravimetric analysis (TGA) of PHMB showed 5% weight loss at 370 °C, which is close to that obtained for a virgin PET powder (390 °C, Table 5). Differential scanning calorimetry showed the glass transition temperature to be 49 °C, which is lower than that of a virgin PET powder (77 °C). The melting temperature (245 °C) and crystallization temperature (165 °C) of PHMB were also found to be slightly lower than those of the virgin PET (254 and 201 °C, respectively). To understand the mechanical properties, films of PHMB and PET were prepared using a Specac constant pressure film maker by applying a constant pressure of 2000 N at 220 °C (for PHMB), and 260 °C (for PET). Stress–strain curve (Figure S190) showed the Young's modulus of PHMB to be 0.95 GPa, which was found to be lower than that of a virgin PET sample measured under the same conditions. The lower Young's modulus of PHMB compared to virgin PET indicates reduced stiffness, suggesting that PHMB may exhibit greater flexibility under mechanical stress. Strain at break for PHMB (10.5%) was found to be similar to that of the virgin PET sample (12%), suggesting that the ductility of PHMB is similar to that of PET. Furthermore, the ultimate tensile stress of PHMB was estimated to be 24 MPa, which was found to be lower in comparison to the PET sample (42 MPa), suggesting that PHMB can bear less load in comparison to PET before failure. Young's modulus and ultimate stress values place PHMB between HDPE and PET, whereas the strain at break suggests that PHMB is less ductile than both PET and HDPE.^[^ ^52^ ^]^

**Table 5:** Properties of PHMB in comparison with virgin PET.

Entry	Polymer	M_n_,^a)^ g/mol	T_d_,^b)^ (5 wt%)	T_g_,^c)^ °С	T_m_,^c)^ °C	T_c_,^c)^ °C	*E*,^d)^ GPa	*F_tu,_* ^e)^ MPa	Strain at break*, %*
1	PHMB	1895	370	49	245	165	0.95	24	10.5
2	PET	2709	390	77	254	201	1.8	42	12

^a)^ Estimated using end group analysis by ^1^H NMR spectroscopy.

^b)^ Measured using thermogravimetric analysis.

^c)^ Measured using differential scanning calorimetry.

^d)^ Young's modulus measured via tensile testing.

^e)^ Ultimate tensile strength measured via tensile testing. *T*_d_ = decomposition temperature, *T*_g_ = glass transition temperature, *T*_m_ = melting temperature, *T*_c_ = crystallisation temperature, *E* = Young's modulus, *F*_tu_ = Ultimate tensile stress.

Having synthesized and characterized PHMB, we were interested in finding out if PHMB can be easily depolymerized to HMBA. Remarkably, we were able to convert PHMB back to virgin HMBA by saponification in 89% yield (Scheme 3).

### **Life Cycle** Assessment

**Scheme 4:**
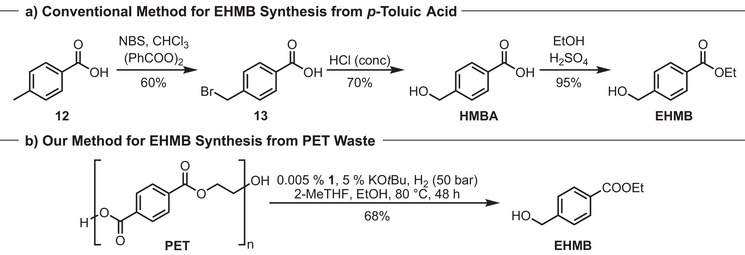
a) Conventional method for synthesis of EHMB from *p*‐toluic acid **12**. b) Method for **EHMB** synthesis using PET waste as a feedstock.

To assess the environmental impact of the newly developed method, we conducted a life cycle assessment of our process and compared it with a conventional method of EHMB production that involves bromination of *p*‐toluic acid **12** with *N*‐bromosuccinimide, followed by hydrolysis of the 4‐(bromomethyl)benzoic acid **13** to HMBA and its esterification catalyzed by sulfuric acid (Scheme 4).^[^ ^53^ ^]^ The life cycle assessment (LCA) in this study refers to a cradle‐to‐gate footprint evaluation, focusing on four environmental footprint impact categories: Global warming potential (GWP), acidification, eutrophication, and water consumption (see Supporting Information for more details). These impact categories are the key metrics provided by the ACS Green Chemistry Institute's streamlined PMI‐LCA tool^[^ ^54^ ^]^ to assess and compare environmental footprints for complex organic reactions.^[^ ^55^ ^]^

**Figure 4:**
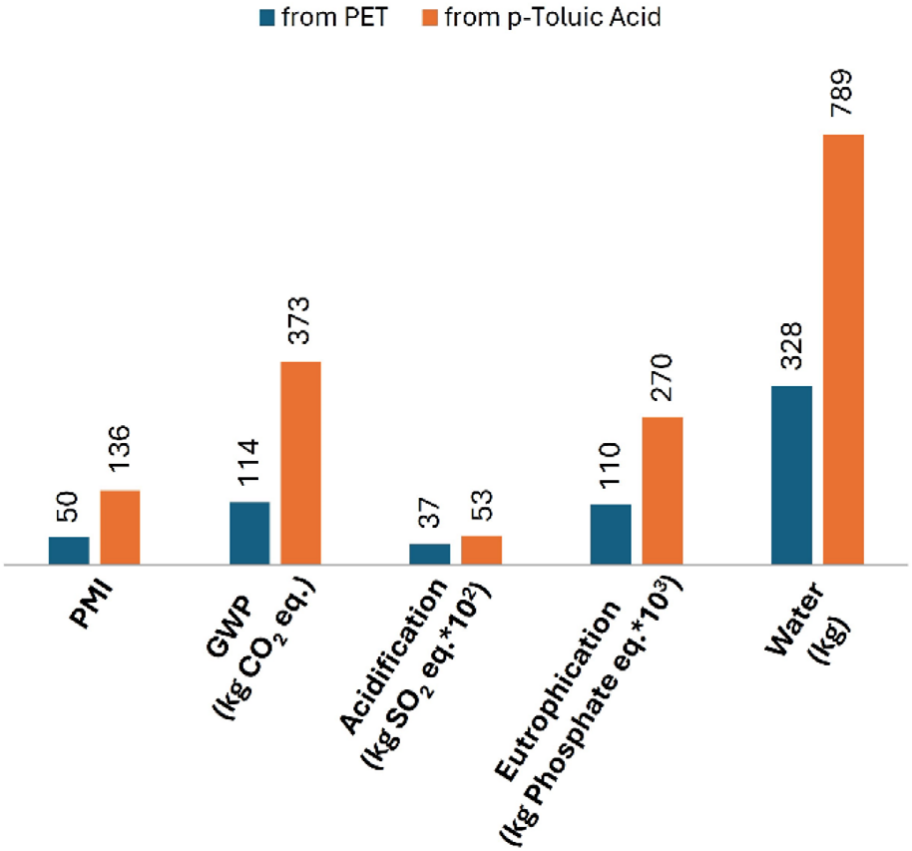
Comparison of the environmental impact of EHMB production from PET waste (dark blue) with that of – from *p*‐toluic acid (orange).

In all assessed dimensions, our route starting from PET waste showed a lower impact: the material efficiency (Process Mass Intensity, PMI), and thereby the generation of waste was reduced more than 2‐fold. This also translates into a reduced carbon footprint expressed through the global warming potential (GWP) of 114 kg CO_2_e kg^−1^ product VS 373 kg CO_2_e kg^−1^ for the conventional route. Likewise, acidification, eutrophication, and water use were all more than 50% lower when compared to the established method (Figure 4, Table S15). In addition, the conventional route starts with fossil feedstocks, while our approach adheres to circular economy principles and re‐uses carbon from the technosphere in the form of PET waste.

## Conclusion

In summary, we present a highly efficient process for the depolymerization of PET, achieving selective semi‐hydrogenation—rather than full hydrogenation—to produce ethyl 4‐(hydroxymethyl)benzoate (EHMB). Through a combination of analytical techniques, particularly ^1^H NMR CEST spectroscopy, we gained key insights into the hydrogenation kinetics and the factors that promote or inhibit the reaction. Guided by this mechanistic understanding, we achieved a record‐high turnover number exceeding 30 000 for the catalytic hydrogenative depolymerization of postconsumer PET waste under moderate conditions (80 °C, 50–60 bar) in bioderived solvents (2‐MeTHF and EtOH). Furthermore, we demonstrated the utility of EHMB as a versatile intermediate for the synthesis of known pharmaceutical compounds and a new, recyclable polyester. These findings offer a promising new strategy for the efficient upcycling of PET waste.

## Supporting Information

The Supporting Information contains details related to catalytic studies, mechanistic studies and synthesis/characterisation of drug feedstock and new polyester.

## Conflict of Interests

The authors declare no conflict of interest.

## Supporting information



Supporting Information

Supporting Information

## Data Availability

The data that support the findings of this study are available in the Supporting Information of this article. The research data supporting this publication can be accessed at https://doi.org/10.17630/32a82d0d‐e1f0‐4f02‐9758‐799e8b1ec826. CCDC 2464903 contains the supplementary crystallographic data for this paper. These data can be obtained free of charge from The Cambridge Crystallographic Data Centre via www.ccdc.cam.ac.uk/structures.
