# Supplementary material for: From Plastic Waste to Pharmaceutical Precursors: PET Upcycling Through Ruthenium Catalyzed Semi‐Hydrogenation
Source: Angew Chem Int Ed Engl. 2025 Dec 18;65(5):e21838. doi: 10.1002/anie.202521838 (PMC12850997; doi:10.1002/anie.202521838)
Supplement: Supplementary file 1 — Supporting Information [file ANIE-65-e21838-s002.docx]

**From Plastic Waste to Pharmaceutical Precursors: PET Upcycling through Ruthenium Catalysed Semi-Hydrogenation**

Pavel S. Kulyabin,^a^ James Luk,^a^ Evgeny A. Uslamin,^b^ Alexander Kolganov,^b^ Garima Saini,^a^ Raymundo Marcial-Hernandez,^a^ Ketan Pancholi,^c^ Benjamin Kühne,^d^ Alexander Dauth,^d^ Aidan P. McKay,^a^ David B. Cordes,^a^ Evgeny A. Pidko,^b^* Amit Kumar^a^*

^a^EaStCHEM, School of Chemistry, University of St. Andrews, North Haugh, St. Andrews, KY169ST, UK.

^b^Inorganic Systems Engineering group, Department of Chemical Engineering, Faculty of Applied Sciences, Delft University of Technology, Van der Maasweg 9, 2629 HZ Delft, The Netherlands.

^c^The Sir Ian Wood Building, Robert Gordon University, Garthdee Rd, Garthdee, Aberdeen, AB107GE, UK.

^d^Merck Group, Corporate Sustainability, Frankfurter Straße 250, 64293 Darmstadt, Germany.

Table of Contents

[1. General Procedure 3](#_Toc205476597)

[2. Optimization of hydrogenation reaction 4](#_Toc205476598)

[2.1 Hydrogenation after alcoholysis 4](#_Toc205476599)

[2.2 Hydrogenation of dimethyl terephthalate (DMTP) 9](#_Toc205476600)

[2.3 One pot alcoholysis and hydrogenation of PET. 16](#_Toc205476601)

[2.3.1 Stage I. 16](#_Toc205476602)

[2.3.2 Stage II. 23](#_Toc205476603)

[2.3.3 Stage III. 27](#_Toc205476604)

[2.3.4 Stage IV. 33](#_Toc205476605)

[2.3.5 Stage V. 38](#_Toc205476606)

[2.3.6 Stage VI. 48](#_Toc205476607)

[2.3.7 Stage VII. 53](#_Toc205476608)

[2.3.8 Stage VIII. 63](#_Toc205476609)

[3. Hydrogenation of PET with 1 in dynamics 71](#_Toc205476610)

[4. Reactions of Ruthenium Hydrides 73](#_Toc205476611)

[4.1 Synthesis of Ruthenium hydrides 1-EtOH and *fac-*1-H_2_ 73](#_Toc205476612)

[4.2 PET hydrogenation using 1, 1-EtOH, and *fac*-1-H_2_. 77](#_Toc205476613)

[4.3 Stoichiometric reactions of Ruthenium hydrides with esters. 78](#_Toc205476614)

[4.3.1 Reaction of complex 1-EtOH with DETP. 78](#_Toc205476615)

[4.3.2 Reaction of 1-EtOH with EHMB. 80](#_Toc205476616)

[5. Kinetic Studies 84](#_Toc205476617)

[5.1. General Procedure 84](#_Toc205476618)

[5.2. Results from maximised TON experiments: 84](#_Toc205476619)

[5.3. ^1^H NMR spectra corresponding to *fac*-1-H_2_ preparation 85](#_Toc205476620)

[5.4. Kinetic plots of PET and DET hydrogenation 87](#_Toc205476621)

[5.5. ^1^H NMR spectra corresponding to kinetic plots of PET and DET hydrogenation 89](#_Toc205476622)

[5.6. ^1^H NMR spectra corresponding to maximising TON experiments 97](#_Toc205476623)

[6. ^1^H NMR CEST 99](#_Toc205476624)

[6.1. General Procedure 99](#_Toc205476625)

[6.2. ^1^H NMR CEST spectra 99](#_Toc205476626)

[6.3. ^1^H NMR spectra of *fac*-1-H_2_ with ethylene glycol and used in CEST 99](#_Toc205476627)

[7. Multi-gram scale hydrogenation. 101](#_Toc205476628)

[8. Ethyl 4-(hydroxymethyl)benzoate as a feedstock. 113](#_Toc205476629)

[7.1 Synthesis of small organic molecules from ethyl 4-(hydroxymethyl)benzoate. 113](#_Toc205476630)

[7.1.1 Preparation of 4-(chloromethyl)benzoyl chloride 10^7^ 113](#_Toc205476631)

[7.1.2 Preparation of ethyl 4-formylbenzoate 11^8^ 113](#_Toc205476632)

[7.1.3 Preparation of ethyl (4-bromomethyl)benzoate 12^9^ 113](#_Toc205476633)

[7.1.4 Preparation of ethyl 4-(aminomethyl)benzoate 13^10^ 113](#_Toc205476634)

[7.2 Synthesis and depolymerization of polyester from 4-(hydroxymethyl)benzoic acid 113](#_Toc205476635)

[7.3 Mechanical properties of PHMB and PET 121](#_Toc205476636)

[9. Life cycle assessment of the production of EHMB. 123](#_Toc205476637)

[10. DFT calculations 125](#_Toc205476638)

[11. X-ray Crystallography 129](#_Toc205476639)

[12. References 130](#_Toc205476640)

# General Procedure

All reactions were performed using standard glovebox and Schlenk techniques. Hydrogen gas was supplied by BOC Gases. Dimethyl terephthalate and diethyl terephthalate were purchased from TCI and used as received. Polyethylene terephthalate samples (granules PET-g, 3-5 mm, crystallinity >50%, inherent viscosity 0.80 dl/g; powder PET-p, 300 micron, crystallinity >50%, inherent viscosity 0.8 dl/g) were purchased from GoodFellow and used as received. Post-consumer polyethylene terephthalate (PET-b) was taken from water bottles from various producers, bottles were chipped with scissors into small pieces (≈5x5 mm). The transition metal precursor dichlorotriphenylphosphine[bis(2-(ethylthio)ethyl)amine]ruthenium(II) (**1**), (dppf)(ampy)RuCl_2_ (**3**), dichlorotriphenylphosphine[2-(diphenylphosphino)-*N*-(2-pyridinylmethyl)ethanamine]-ruthenium(II) (**6**) were generously donated by Johnson Matthey and used as received. Ru-MACHO^®^ (**5**), chlorodihydrido[bis(2-(diisopropylphosphino)ethyl)amine]iridium(III) (**7**), [2-(di-tert-butylphosphinomethyl)-6-(diethylaminomethyl)pyridine]carbonylchlorohydrido-ruthenium(II) (**4**), dichlorobis[3-(diphenylphosphino]ethylamine]ruthenium(II) (**2**) were purchased from Strem and used as received. If not mentioned otherwise, all organic solvents (2-methyltetrahydrofuran, toluene, ethanol, butanol-1, isopropanol, tetrahydrofuran, *tert*-amyl alcohol) were purchased from Merck as anhydrous solvent. Afterwards, the solvents were degassed by flushing them with argon through an argon lance equipped with a frit. The solvents were dried and stored in glass Schlenk bottles over molecular sieves 3 Å and 4 Å.

All NMR spectra were recorded using BRUKER AV-400 and AV-500 spectrometers. The samples were measured with mesitylene as an internal standard and locked to the deuterium signal of the used solvents. The residual proton peak of the respective solvent was used as an internal reference. Chemical shifts δ are reported in ppm and coupling constants J in Hz. Multiplicity is reported as: s- singlet, d- doublet, t- triplet, m- multiplet, bs - broad signal.

Infrared spectra (ATR-FTIR) were collected using a Shimadzu IRAffinity-1.

Thermogravimetric Analysis (TGA) was performed using Stanton Redcroft STA-780 Series Thermal Analyser between 30–900 °C at a heating rate of 10 °C/min under a flow of nitrogen gas (25 mL/min). Decomposition temperature (T_d_, °C) was estimated as the temperature of 5% weight loss.

Differential Scanning Calorimetry (DSC) analyses were performed using a Netzsch DSC204 between - 40–300 °C at a heating rate of 10 °C/min under a flow of nitrogen gas (20 mL/min) after an initial heat/cool cycle (25–300 °C at 10 °C/min) to remove the thermal history of the sample.

GC-MS data were collected as solutions in HPLC grade DCM using an Agilent 8860 GC system coupled to an Agilent 5977B EI instrument. EI spectra were collected as solutions in acetonitrile using a Micromass LCT spectrometer.

# Optimization of hydrogenation reaction

## 2.1 Hydrogenation after alcoholysis

Inspired by Xie’s work^1^ we first tried to perform alcoholysis before ester hydrogenation (Table S1). Thus, polymer derived from a bottle of water (PET-b) was first subjected to alcoholysis using methanol or ethanol and various bases at 100 or 120 °C. Then the resulting solution was transferred to a vial with 1 mol% of the complex **1** and hydrogen pressure was applied at 80 °C or 100 °C presuming that there was some base left after alcoholysis that would be sufficient to activate the precatalyst **1** (entries 1–3, Table S1). Alcoholysis performed at a lower temperature of 100 °C or with polymer granules (PET-g) showed incomplete conversion of PET and lower hydrogenation yield (entries 4–9, Table S1). The hydrogenation worked better when an additional amount of base was added during hydrogenation, but the yields were still mediocre (entries 10–13, Table S1).

**General procedure A.**

Polyethylene terephthalate (192.2 mg, 1 mmol, 1 eq., taken from a plastic bottle or granules) and base (KO*t*Bu, NaO*t*Bu, K_2_CO_3_), were weighed in air, placed into a 50 mL glass ampule to which a stir bar was added, and the ampule was sealed with a JY cap. The ampule was backfilled with argon, and THF (3 mL) and alcohol (2 mL) were added via syringe. The ampule was heated in an oil bath at the specified temperature for the specified time. Next, the prepared solution was cannulated into an 8 mL vial fitted with a septum with 1 mol% of precatalyst **1** or **7** and (in selected cases) an additional amount of base under inert gas. The vials were placed inside a 150 mL autoclave with some metal beads to ensure thermal conductivity. The autoclave was purged with argon, then sealed, purged with H_2_, pressurized with H_2_ at 50 bar, and placed in a preheated oil bath. The reaction mixture was stirred for 24 h. After that, the autoclave was cooled down to room temperature first in air and then in an ice bath and carefully vented to the atmosphere. The reaction mixture was then diluted with 2 mL of MeOH and stirred for 10 min at room temperature, the vial was weighed. The yield of the product was estimated by NMR spectroscopy using mesitylene as an internal standard. Hence, 200–300 mg of the reaction solution and ≈10 mg mesitylene were weighed in an NMR tube, followed by the addition of methanol-d4 (0.3 mL).

| **Table S1. PET alcoholysis combined with hydrogenation.*^[a]^***   | | | | | | | | | | | | |
| --- | --- | --- | --- | --- | --- | --- | --- | --- | --- | --- | --- | --- |
| Entry | Start mat.*^b^* | alcohol | Alcoholysis | | | | Hydrogenation | | | Yield of BDM | Yield of (R)HMB | Yield of D(R)TP |
|  |  |  | base | T, ºC | Time, h | Conv.*^c^* | base | cat | T, ºC |  |  |  |
| 1 | PET-b | MeOH | 10% K_2_CO_3_ | 120 | 19 | 100% | none | 1% **1** | 80 | <1% | <1% | 81% |
| 2 | PET-b | MeOH | 10% KOtBu | 120 | 19 | 100% | none | 1% **1** | 80 | 18% | 15% | 41% |
| 3 | PET-b | MeOH | 10% NaOtBu | 120 | 19 | 100% | none | 1% **1** | 80 | <1% | <1% | 87% |
| 4 | PET-b | MeOH | 5% K_2_CO_3_ | 100 | 19 | 91% |  |  |  |  |  |  |
| 5 | PET-b | MeOH | 5% KOtBu | 100 | 19 | 49% |  |  |  |  |  |  |
| 6 | PET-g | EtOH | 10% KOtBu | 120 | 24 | 13% |  |  |  |  |  |  |
| 7 | PET-g | MeOH | 10% KOtBu | 120 | 24 | 35% |  |  |  |  |  |  |
| 8 | PET-g | EtOH | 10% KOtBu | 100 | 24 | 14% |  |  |  |  |  |  |
| 9 | PET-g | MeOH | 10% KOtBu | 100 | 24 | 53% |  |  |  |  |  |  |
| 10 | PET-b | EtOH | 10% KOtBu | 120 | 16 | 100% | 10% KOtBu | 1% **1** | 100 | <1% | <1% | 99% |
| 11 | PET-b | EtOH | 10% KOtBu | 100 | 16 | 98% | 10% KOtBu | 1% **6** | 100 | <1% | <1% | 98% |
| 12 | PET-b | MeOH | 10% KOtBu | 120 | 18 | 100% | 10% KOtBu | 1% **1** | 80 | 57% | 34% | <1% |
| 13 | PET-b | EtOH | 10% KOtBu | 120 | 18 | 100% | 10% KOtBu | 1% **1** | 80 | 66% | 14% | <1% |
| *[a]* General procedure A. *[b]* Two sources of PET were used: plastic bottle (PET-b) and granulate PET (PET-g). *[c]* Conversion of polymer was calculated as a difference between plastic loading and solid residue after the reaction. | | | | | | | | | | | | |


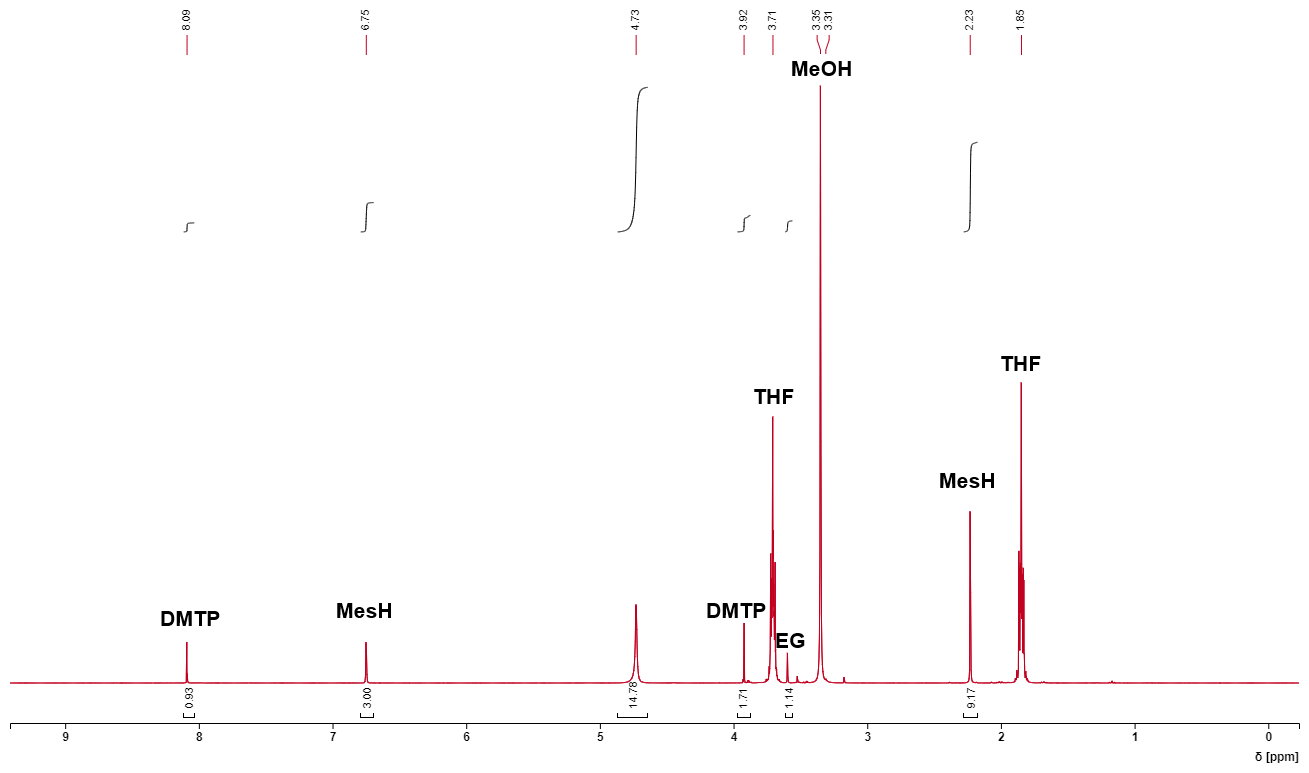


Figure S1. ^1^H NMR (400 MHz, methanol-d_4_) spectrum of reaction mixture from Entry 1, Table S1.


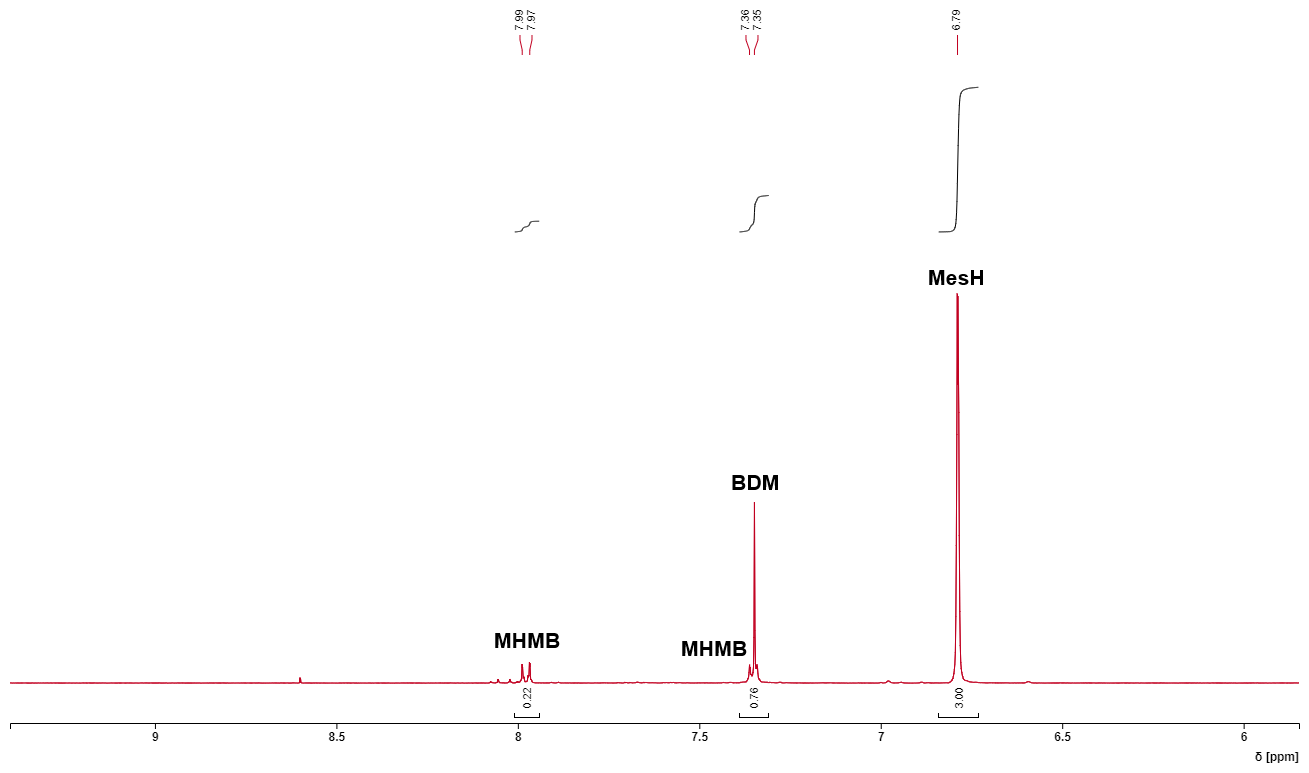


Figure S2. Fragment of ^1^H NMR (400 MHz, methanol-d4) spectrum of reaction mixture from Entry 2, Table S1.


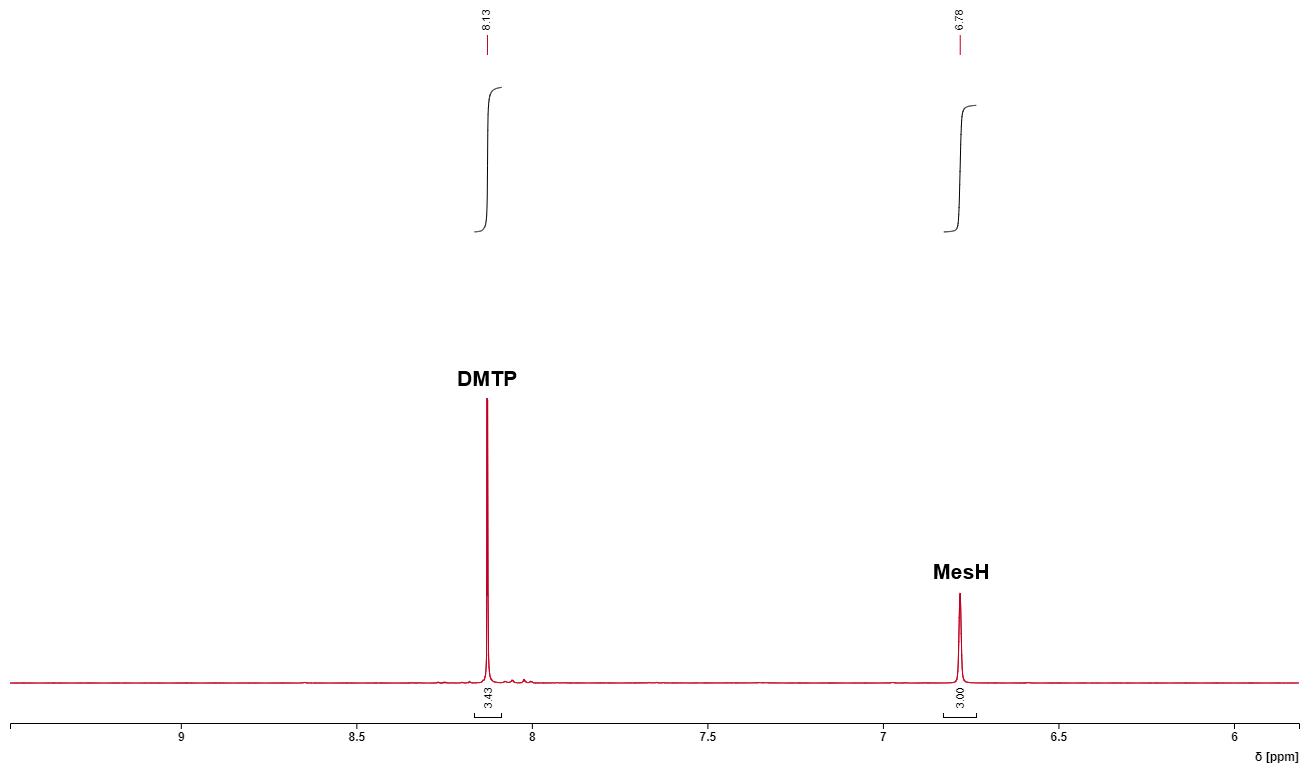


Figure S3. Fragment of ^1^H NMR (400 MHz, methanol-d4) spectrum of reaction mixture from Entry 3, Table S1.


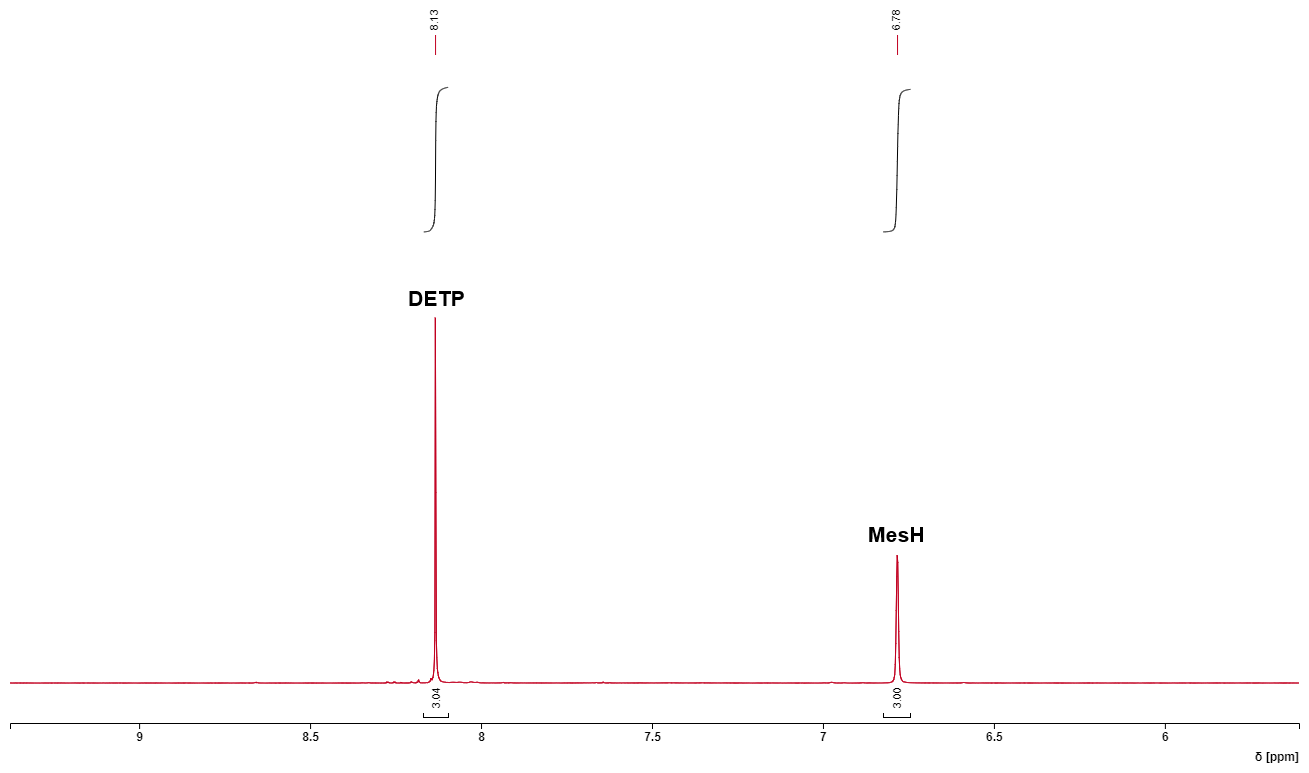


Figure S4. Fragment of ^1^H NMR (400 MHz, methanol-d4) spectrum of reaction mixture from Entry 10, Table S1.


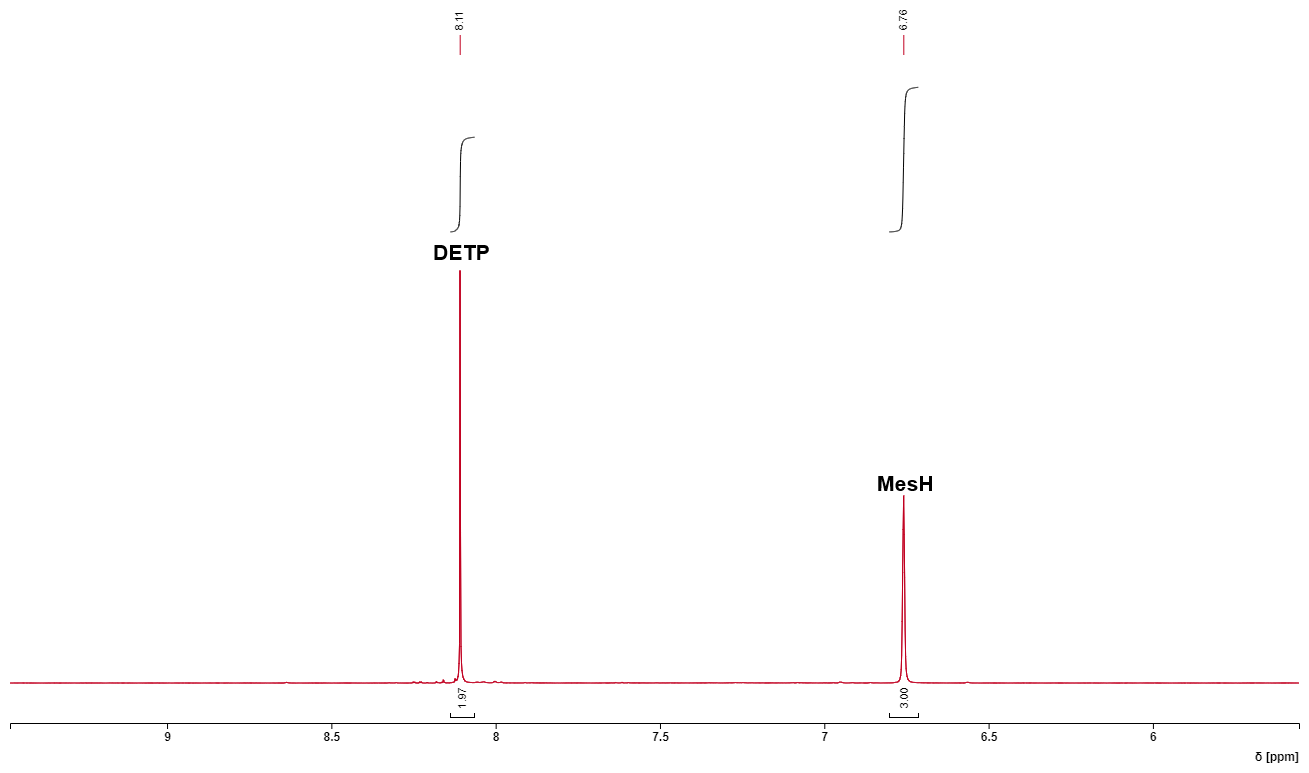


Figure S5. Fragment of ^1^H NMR (400 MHz, methanol-d4) spectrum of reaction mixture from Entry 11, Table S1.


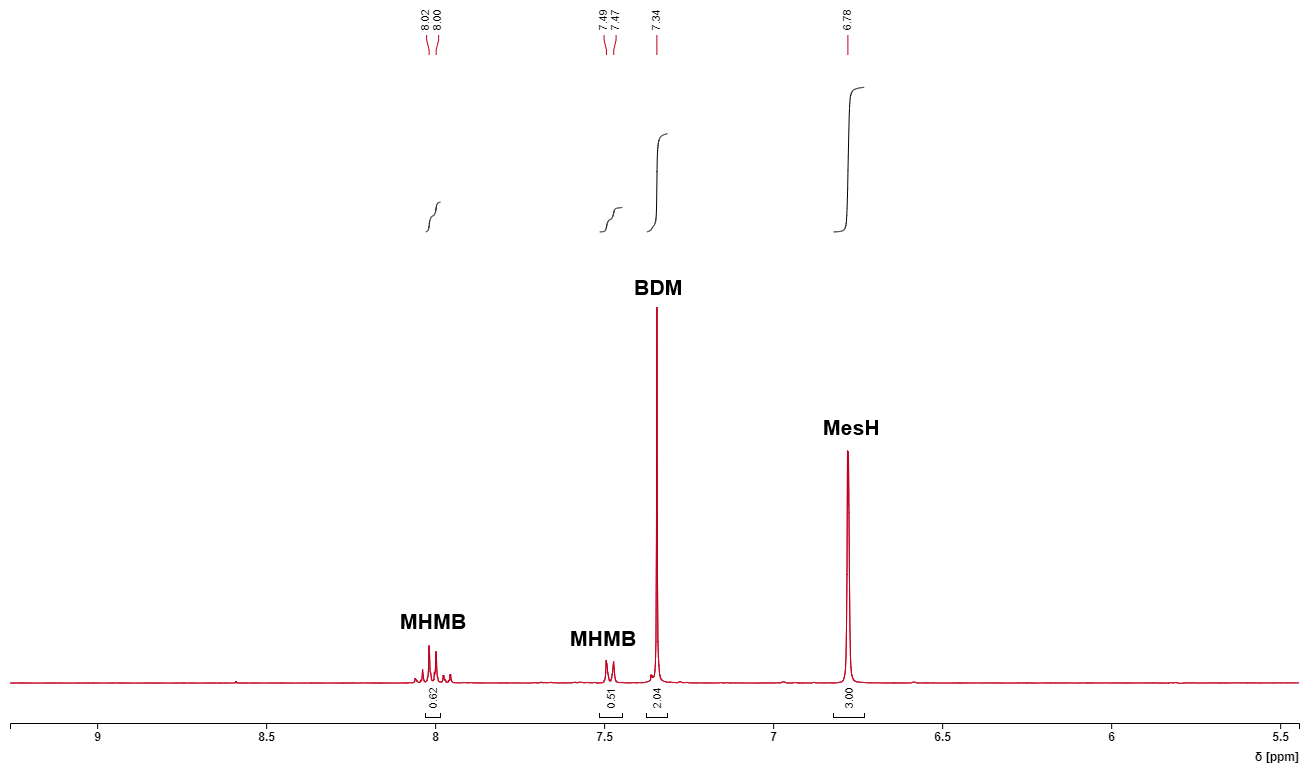


Figure S6. Fragment of ^1^H NMR (400 MHz, methanol-d4) spectrum of reaction mixture from Entry 12, Table S1.


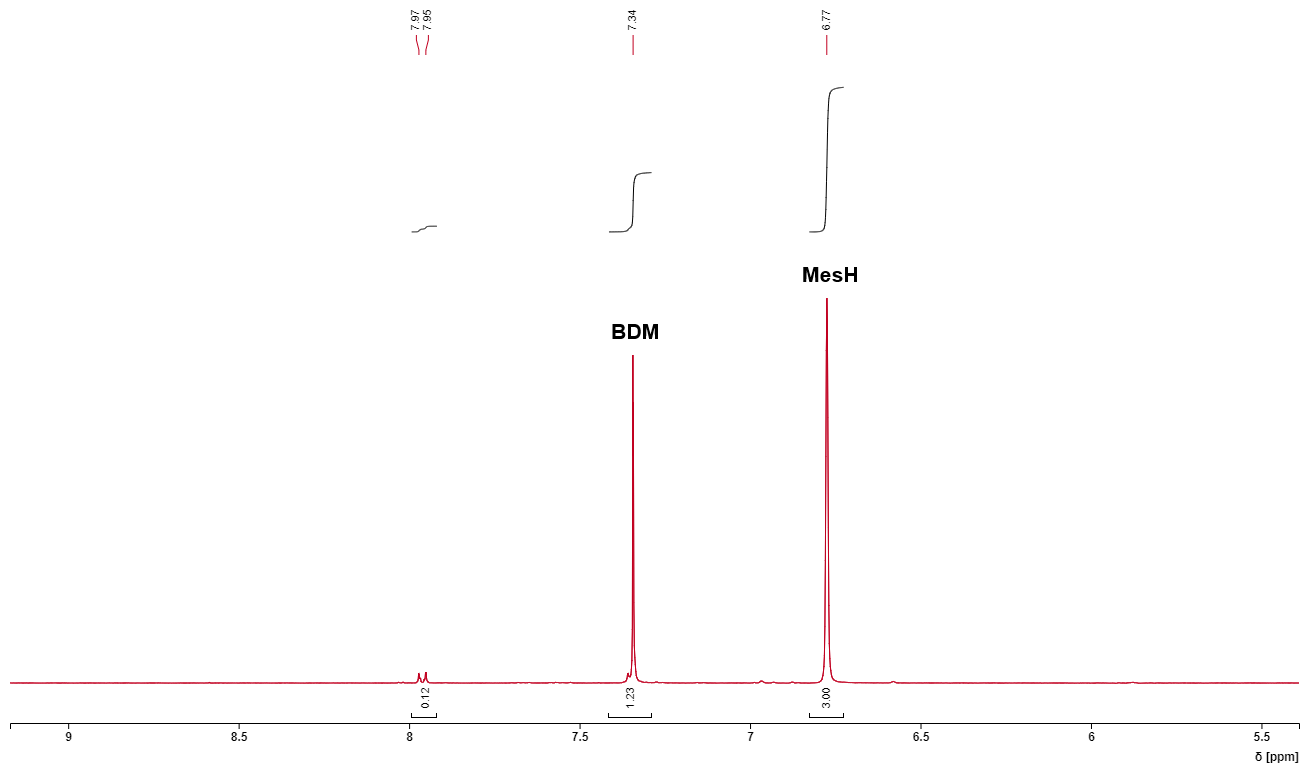


Figure S7. Fragment of ^1^H NMR (400 MHz, methanol-d4) spectrum of reaction mixture from Entry 13, Table S1.

## 2.2 Hydrogenation of dimethyl terephthalate (DMTP)

In contrast to the results in Table S1, precatalyst **1** successfully hydrogenated DMTP to 1,4-benzenedimethanol (BDM) in analogous reaction conditions in various solvents (Table S2) including MeOH, EtOH and THF/ethylene glycol (EG).

**General procedure B.**

Dimethyl terephthalate (**DMTP**, 194.2 mg, 1 mmol, 1 eq.), base (10 mol%), and precatalyst **1** (6.3 mg, 0.01 mmol, 1 mol%) were weighed in air, placed into an 8 mL glass vial to which a stir bar was added, and the vial was sealed with a septum. The vial was purged with argon, and solvent(s) were added via syringe. Two needles were added at the top of the vial, after which it was placed inside a 150 mL autoclave with some metal beads to ensure thermal conductivity. The autoclave was purged with argon, then sealed, purged with H_2_, pressurized with H_2_ at 50 bar, and placed in a preheated oil bath. The reaction was stirred at 80 °C for the specified time. After that, the autoclave was cooled down to room temperature in air and then in an ice bath and carefully vented to the atmosphere. After the reaction mixture was diluted with 2 mL of MeOH and stirred for 10 min at room temperature, the vial was weighed. The yield of the product was estimated by NMR spectroscopy using mesitylene as an internal standard. Hence, 200–300 mg of the reaction solution and ≈10 mg mesitylene were weighed in an NMR tube, followed by the addition of methanol-d4 (0.3 mL).

| **Table S2. Hydrogenation of dimethyl terephthalate with complex 1.***^[^****^a]^***   | | | | | |
| --- | --- | --- | --- | --- | --- |
| Entry | Solvent | Base | Time, h | Yield of BDM, % | Yield of MHMB, % |
| 1 | 5 mL THF | KO*t*Bu | 24 | 98 | <1 |
| 2 | 3 mL THF/ 2 mL MeOH | KO*t*Bu | 24 | 78 | 8 |
| 3 | 5 mL THF/ 1 eq EG | KO*t*Bu | 24 | 98 | <1 |
| 4 | 3 mL THF/ 2 mL EtOH | KO*t*Bu | 24 | 98 | <1 |
| 5 | 2 mL tAmOH/ 2 mL EtOH | KO*t*Bu | 24 | 95 | <1 |
| 6 | 4 mL EtOH | KO*t*Bu | 24 | 99 | <1 |
| 7 | 2 mL 2-MeTHF/ 2 mL EtOH | KO*t*Bu | 24 | 99 | <1 |
| 8 | 5 mL EtOH | K_2_CO_3_ | 24 | 90 | 9 |
| 9 | 5 mL EtOH | KO*t*Bu | 16 | 99 | <1 |
| 10 | 5 mL MeOH | KO*t*Bu | 16 | 14 | 83 |
| *[a]* General procedure B. | | | | | |


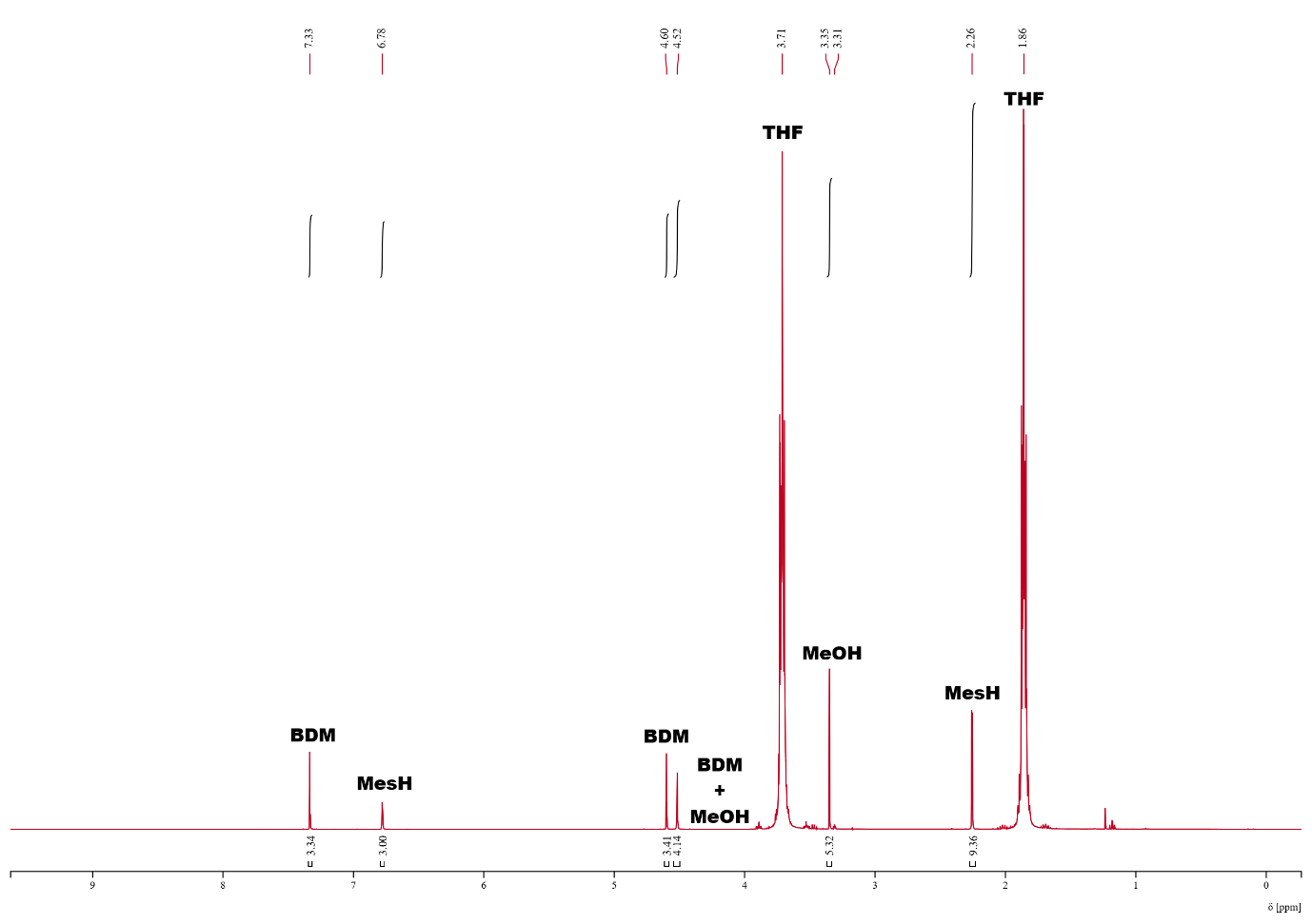


Figure S8. ^1^H NMR (400 MHz, methanol-d4) spectrum of reaction mixture from Entry 1, Table S2.


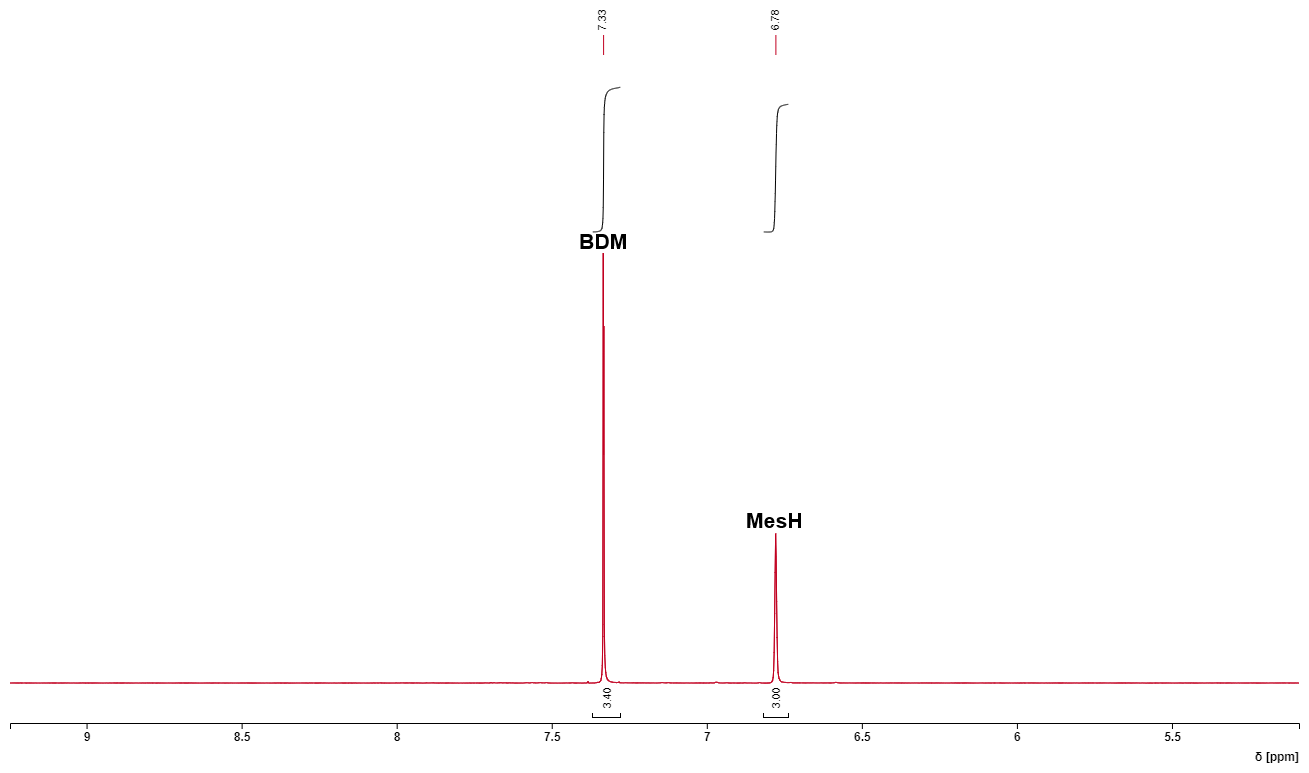


Figure S9. Fragment of ^1^H NMR (400 MHz, methanol-d4) spectrum of reaction mixture from Entry 1, Table S2.


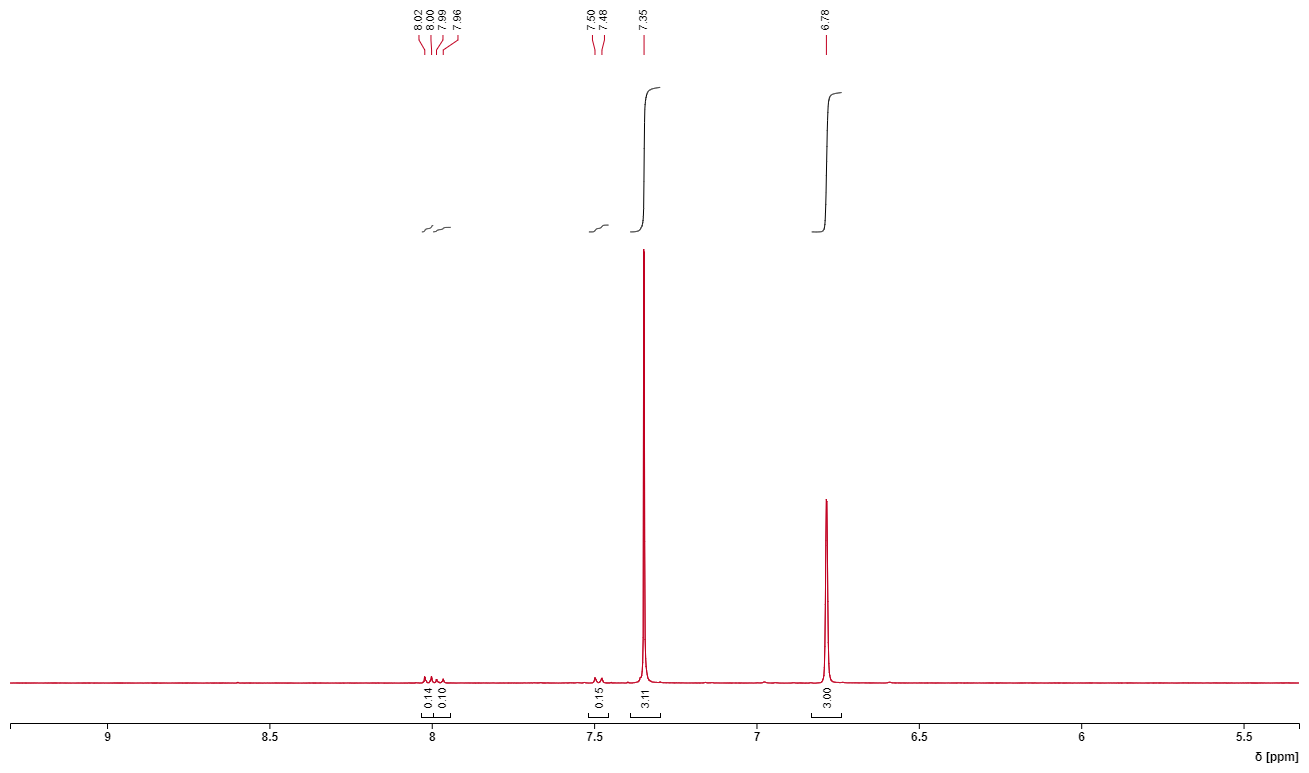


Figure S10. Fragment of ^1^H NMR (400 MHz, methanol-d4) spectrum of reaction mixture from Entry 2, Table S2.


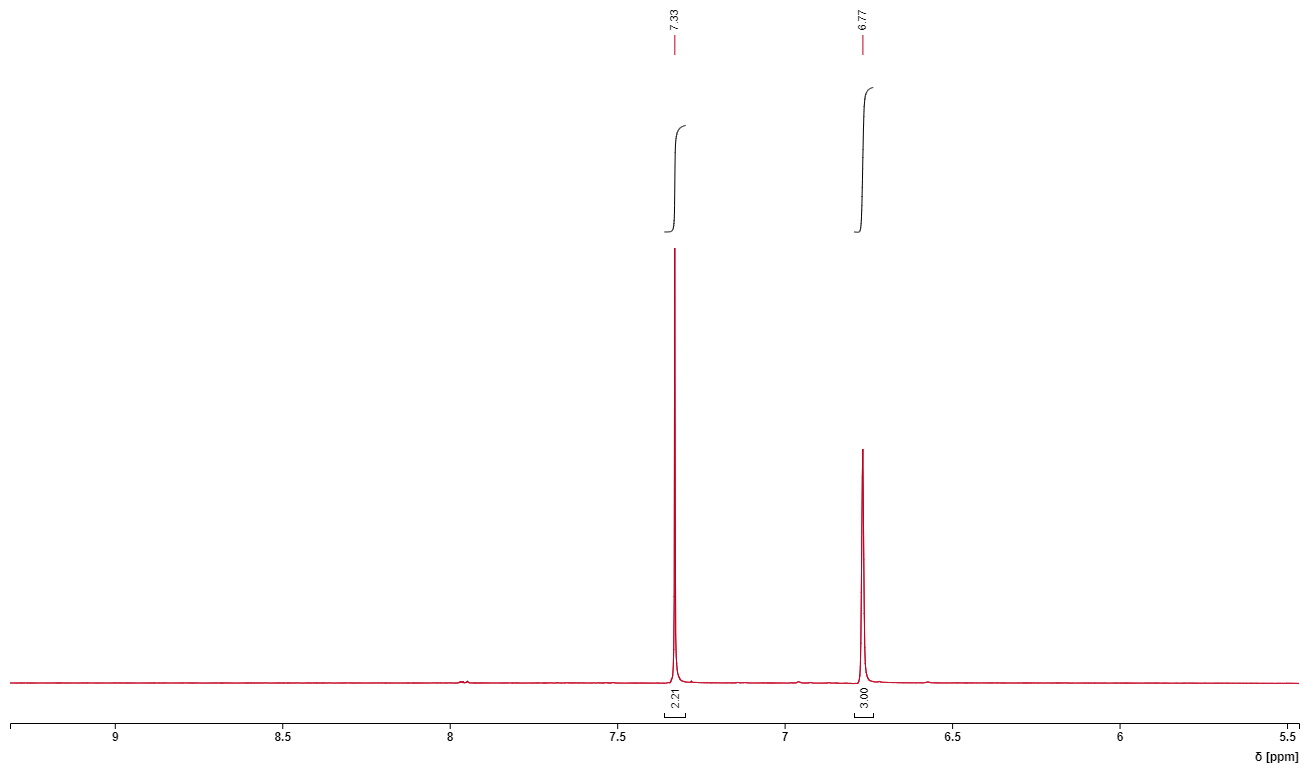


Figure S11. Fragment of ^1^H NMR (400 MHz, methanol-d4) spectrum of reaction mixture from Entry 3, Table S2.


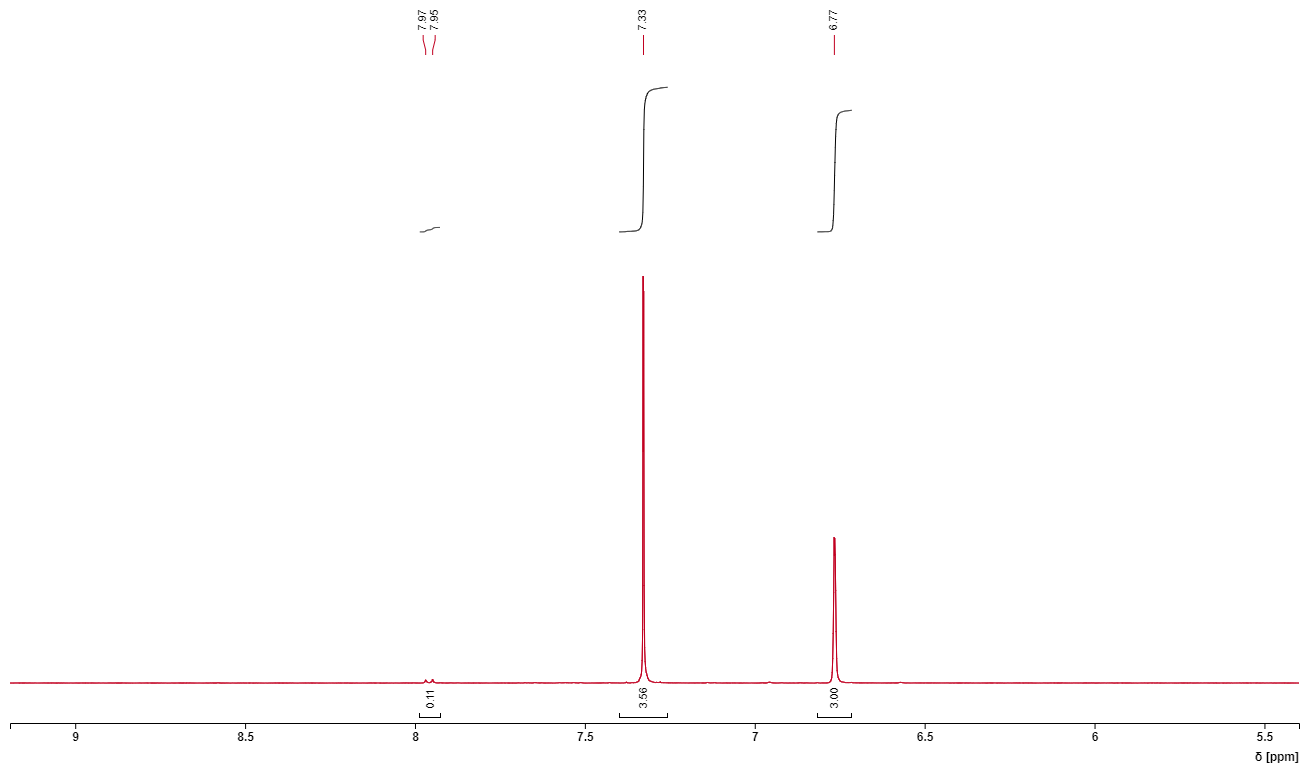


Figure S12. Fragment of ^1^H NMR (400 MHz, methanol-d4) spectrum of reaction mixture from Entry 4, Table S2.


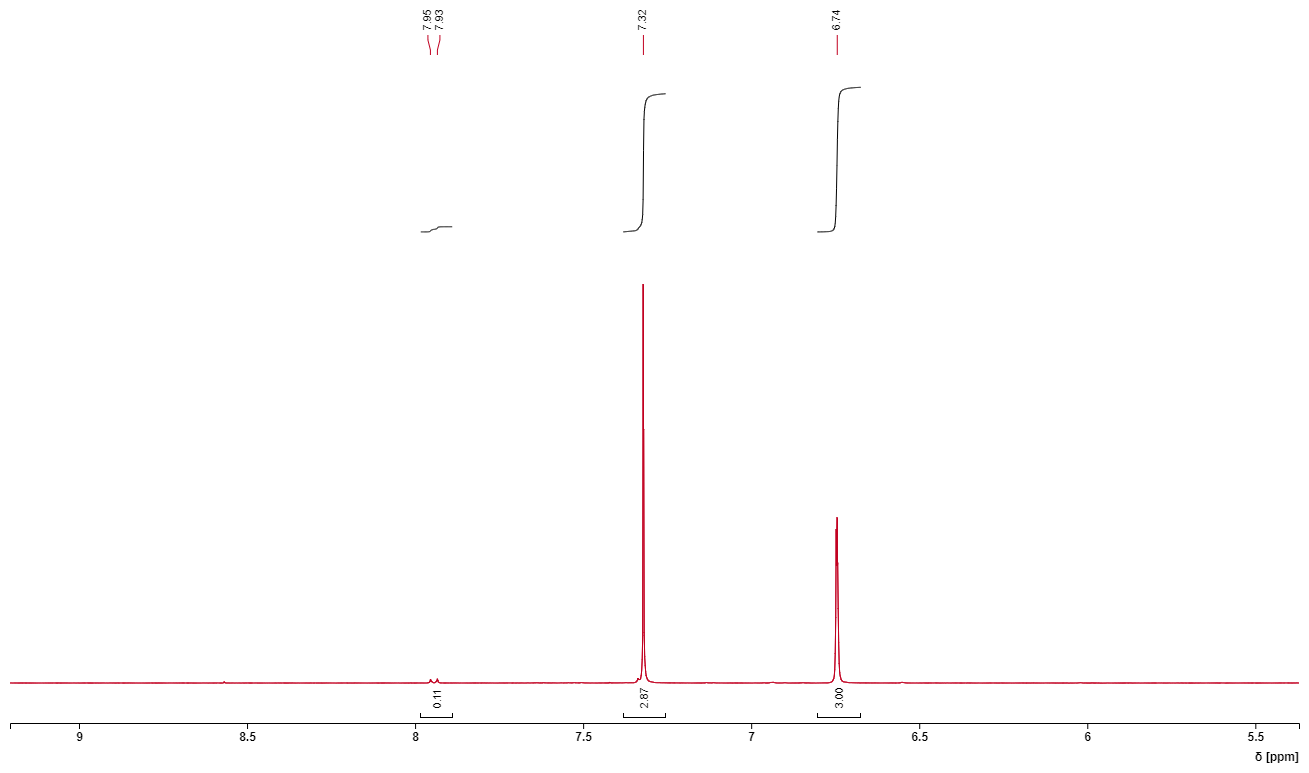


Figure S13. Fragment of ^1^H NMR (400 MHz, methanol-d4) spectrum of reaction mixture from Entry 5, Table S2.


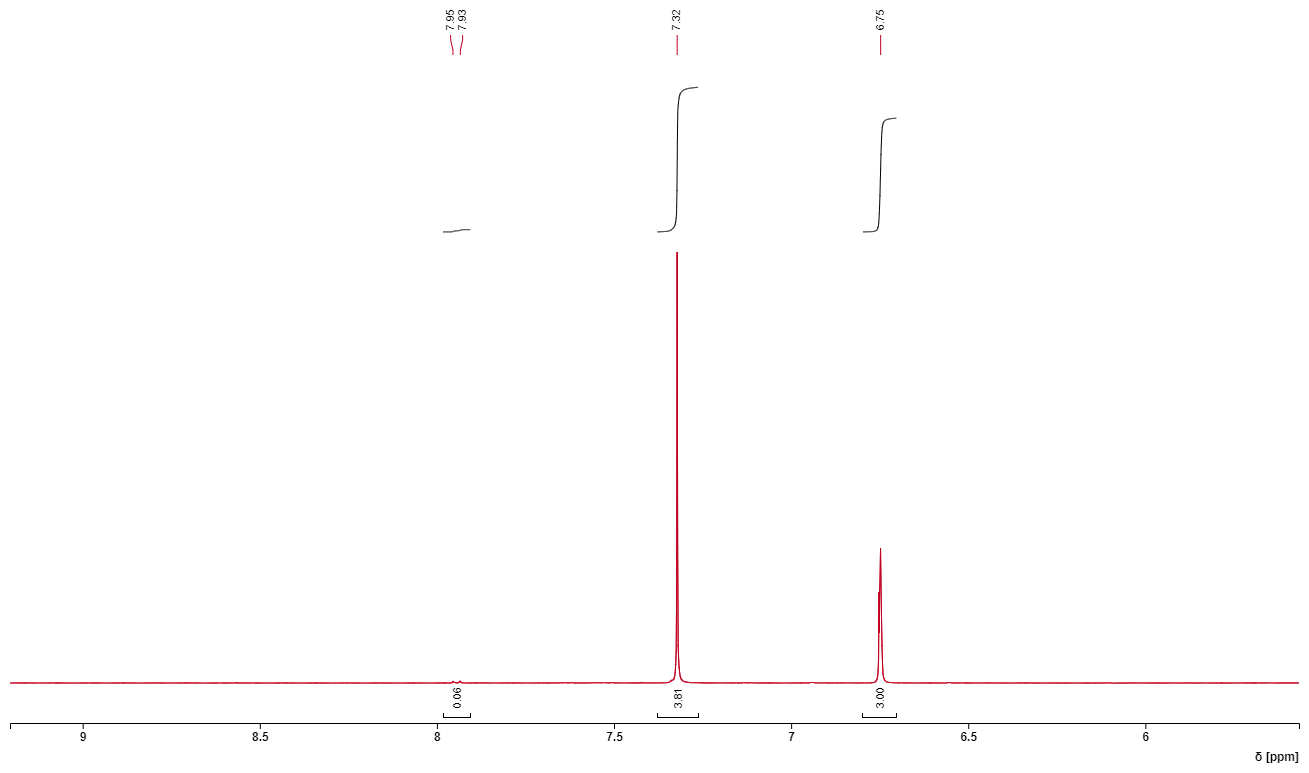


Figure S14. Fragment of ^1^H NMR (400 MHz, methanol-d4) spectrum of reaction mixture from Entry 6, Table S2.


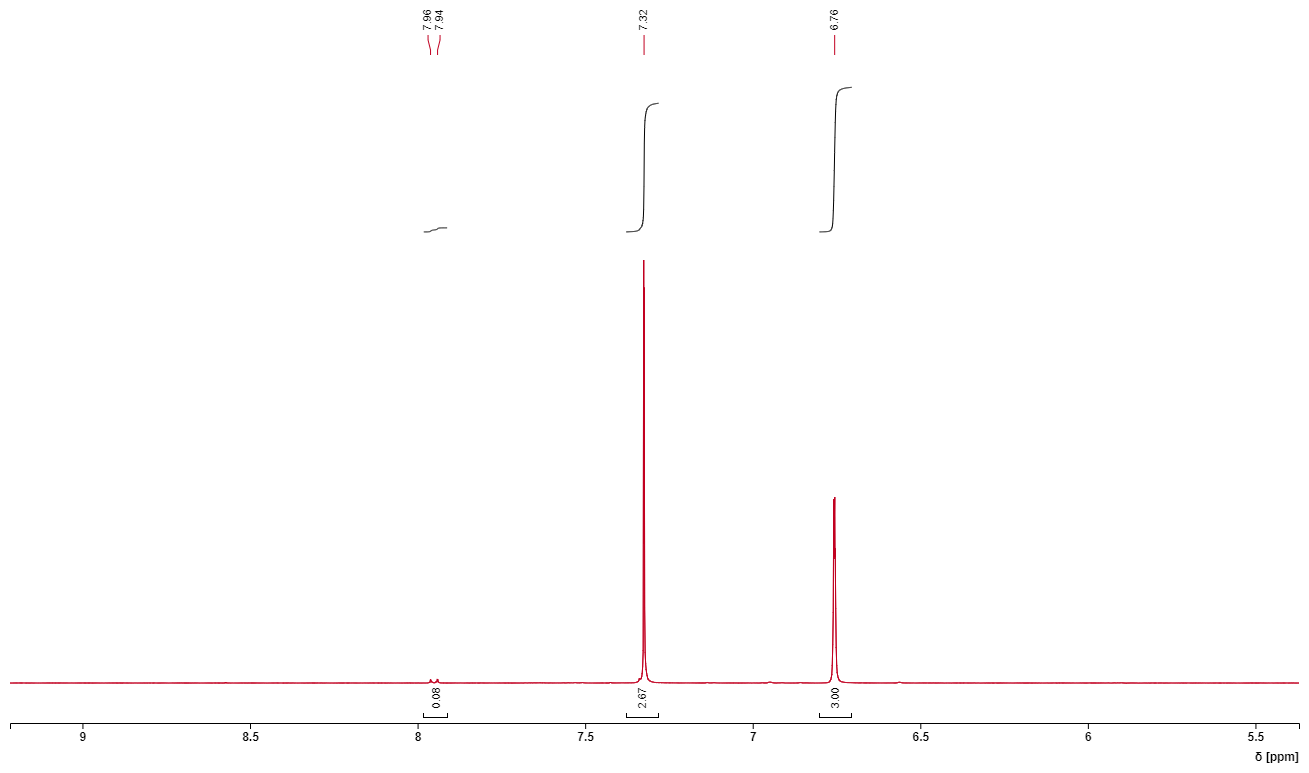


Figure S15. Fragment of ^1^H NMR (400 MHz, methanol-d4) spectrum of reaction mixture from Entry 7, Table S2.


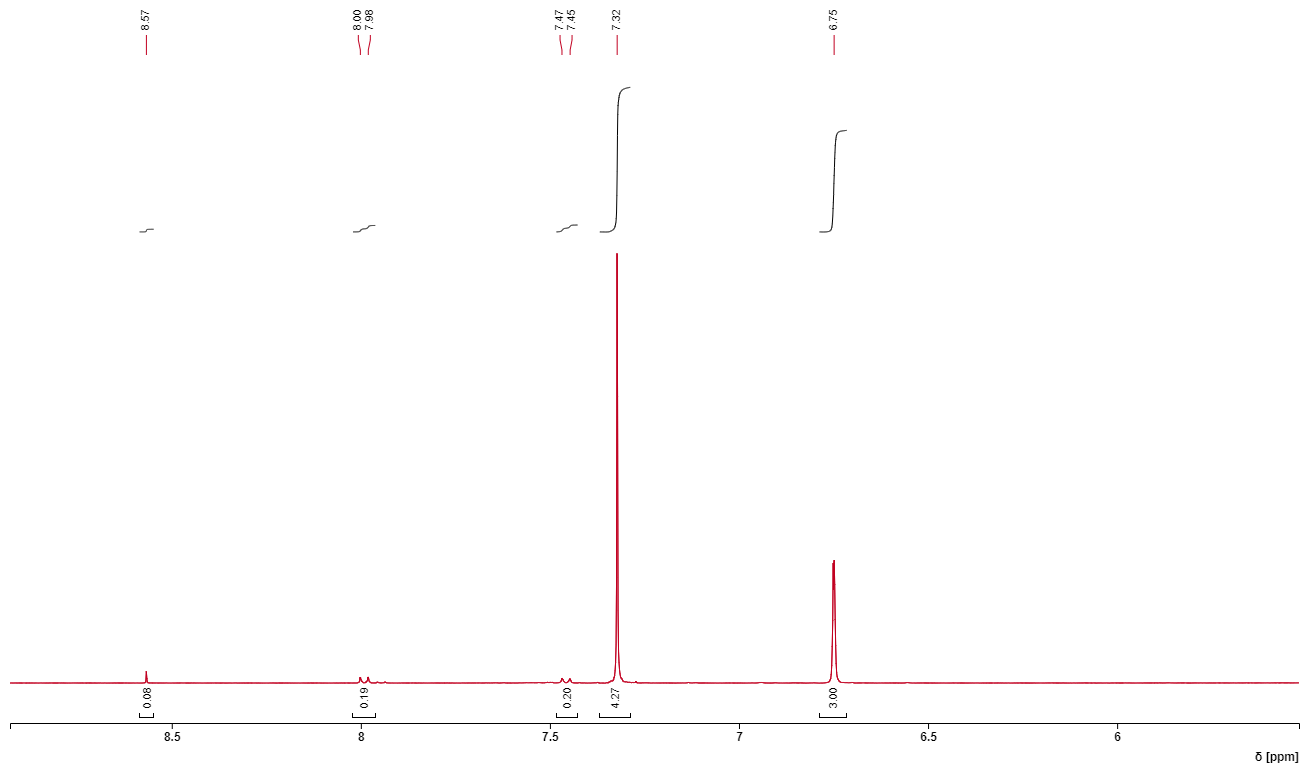


Figure S 16. Fragment of ^1^H NMR (400 MHz, methanol-d4) spectrum of reaction mixture from Entry 8, Table S2.


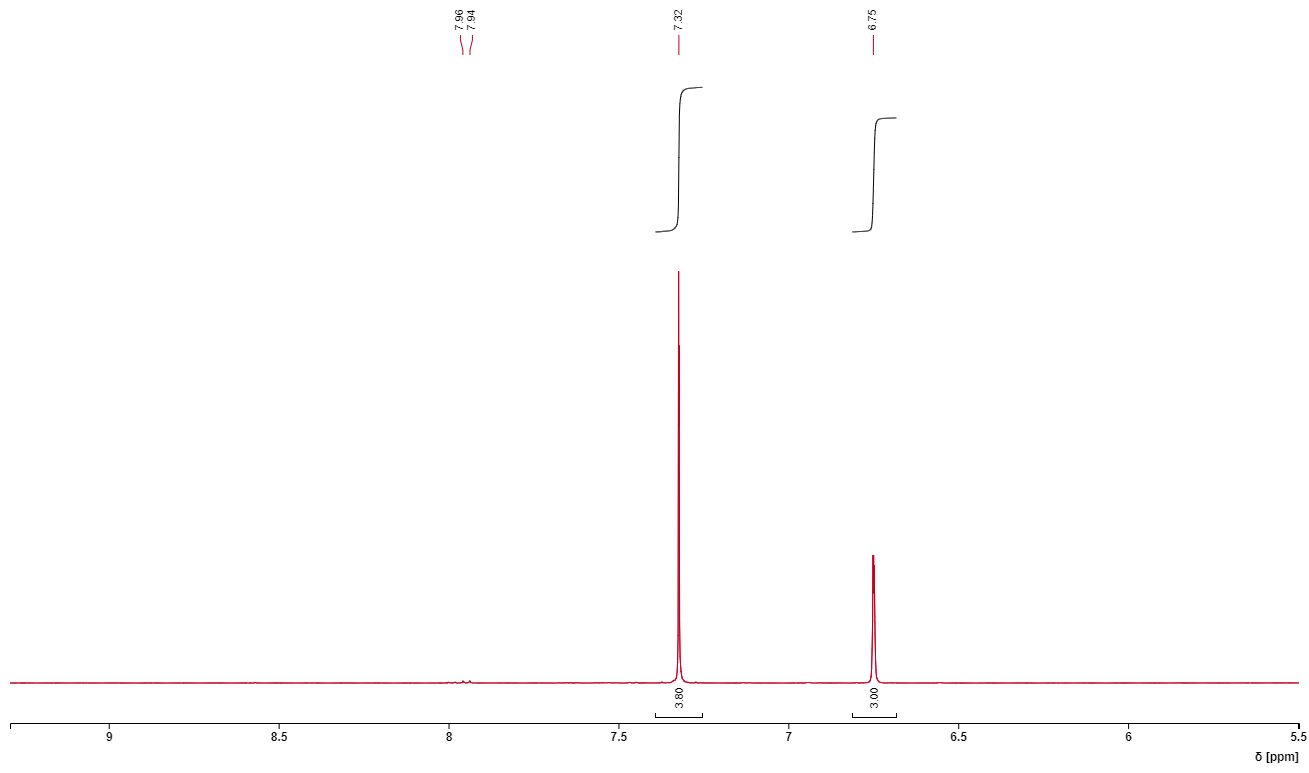


Figure S17. Fragment of ^1^H NMR (400 MHz, methanol-d4) spectrum of reaction mixture from Entry 9, Table S2.


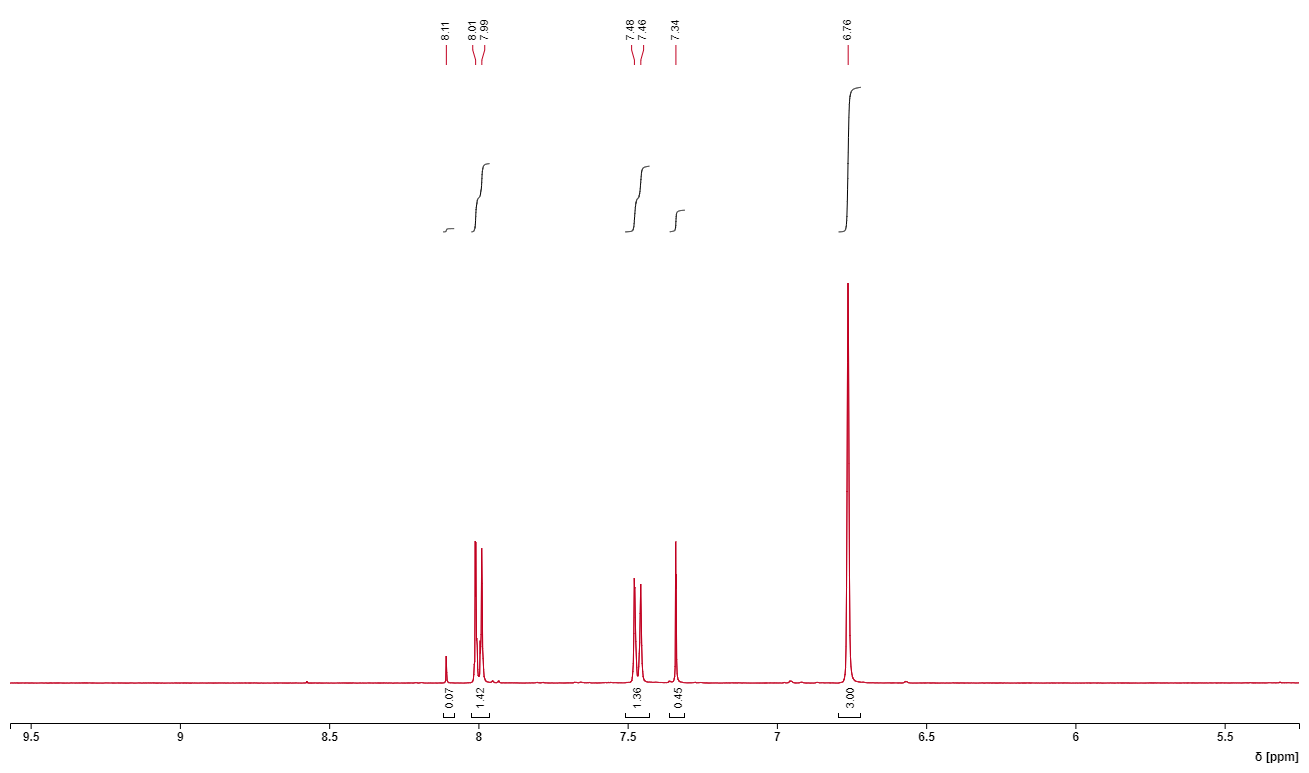


Figure S18. Fragment of ^1^H NMR (400 MHz, methanol-d4) spectrum of reaction mixture from Entry 10, Table S2.

## 2.3 One pot alcoholysis and hydrogenation of PET.

### 2.3.1 Stage I.

Next, one pot alcoholysis and hydrogenation with 1 mol% of **1** and 10% *t*BuOK was performed which gave 99% yield of 1,4-benzenedimethanol (Entry 1, Table S3). It became clear that there was no need to separate these two processes. These conditions were used as a starting point for further optimization. Next, hydrogenation of PET was performed in neat alcohols and a mixture of THF or 2-MeTHF and alcohols. It was demonstrated that the best result was obtained in a mixture of 2-MeTHF and ethanol, which allowed to obtain quantitative yield of BDM with 0.5 mol% of precatalyst **1** (Entry 8, Table S3). Interestingly, utilization of THF gave a lower yield than 2-MeTHF (Entries 7, Table S3). This observation was also made by Johnson Matthey for ester hydrogenation.^2^ However decrease in catalytic loadings led to a conversion drop and higher yield of (R)HMB products (Entries 11-13, Table S3).

**General procedure C.**

PET-b (192.2 mg, 1 mmol, 1 eq. taken from a bottle), base (5 or 10 mol%), and precatalyst **1** (0.63–6.3 mg, 0.001–0.01 mmol, 0.1–1 mol%) were weighed in air, placed into a 8 mL glass vial to which a stir bar was added, and the vial was sealed with a septum. The vial was purged with argon, and solvents were added via syringe. Two needles were added at the top of the vial, after which it was placed inside a 150 mL autoclave with some metal beads to ensure thermal conductivity. The autoclave was purged with argon, then sealed, purged with H_2_, pressurized with H_2_ at 50 bar, and placed in a preheated oil bath. The reaction was stirred at 80 °C for the specified time. After that, the autoclave was cooled down to room temperature in air and then in an ice bath and carefully vented to the atmosphere. After the reaction mixture was diluted with 2 mL of MeOH and stirred for 10 min at room temperature, the vial was weighed. The yield of the product was estimated by NMR spectroscopy using mesitylene as an internal standard. Hence, 200–300 mg of the reaction solution and ≈10 mg mesitylene were weighed in an NMR tube, followed by the addition of methanol-d4 (0.3 mL).

| **Table S3. Hydrogenation of PET with complex 1.***^[^****^a]^***   | | | | | | | | |
| --- | --- | --- | --- | --- | --- | --- | --- | --- |
| Entry | solvent | base | Cat, mol% | Time, h | Conv.^[b]^, % | Yield of BDM, % | Yield of (R)HMB, % | Yield of D(R)TP, % |
| 1 | 3 mL THF/2 mL EtOH | 10% KOtBu | 1 | 24 | 100 | 99 | <1 | <1 |
| 2 | 3 mL THF/2 mL EtOH | 10% KOtBu | 1 | 18 | 100 | 99 | <1 | <1 |
| 3 | 3 mL THF/2 mL EtOH | 10% K_2_CO_3_ | 1 | 18 | 5 | 3 | 2 | <1 |
| 4 | 3 mL THF/2 mL *n*BuOH | 10% KOtBu | 1 | 18 | 100 | 99 | <1 | <1 |
| 5 | 5 mL EtOH | 10% KOtBu | 1 | 24 | 83 | 71 | 10 | 1 |
| 6 | 5 mL *n*BuOH | 10% KOtBu | 1 | 18 | 19 | 15 | <1 | <1 |
| 7 | 4.5 mL THF/0.5 mL EtOH | 10% KOtBu | 0.5 | 18 | 77 | 13 | 55 | 3 |
| 8 | 4.5 mL 2-MeTHF/0.5 mL EtOH | 10% KOtBu | 0.5 | 18 | 100 | 98 | <1 | <1 |
| 9 | 4.5 mL 2-MeTHF/0.5 mL *n*BuOH | 10% KOtBu | 0.5 | 18 | 100 | 94 | <1 | <1 |
| 10 | 4.5 mL 2-MeTHF/0.5 mL EtOH | 5% KOtBu | 0.5 | 18 | 100 | 87 | 9 | <1 |
| 11 | 4.5 mL 2-MeTHF/0.5 mL EtOH | 5% KOtBu | 0.2 | 18 | 50 | 15 | 32 | 2 |
| 12 | 4.5 mL 2-MeTHF/0.5 mL *n*BuOH | 5% KOtBu | 0.2 | 18 | 17 | 3 | 13 | <1 |
| 13 | 4.5 mL 2-MeTHF/0.5 mL EtOH | 5% KOtBu | 0.1 | 18 | 39 | 3 | 32 | 4 |
| *[a]* General procedure C. *[b]* Conversion of polymer was calculated as a difference between plastic loading and solid residue after the reaction. | | | | | | | | |


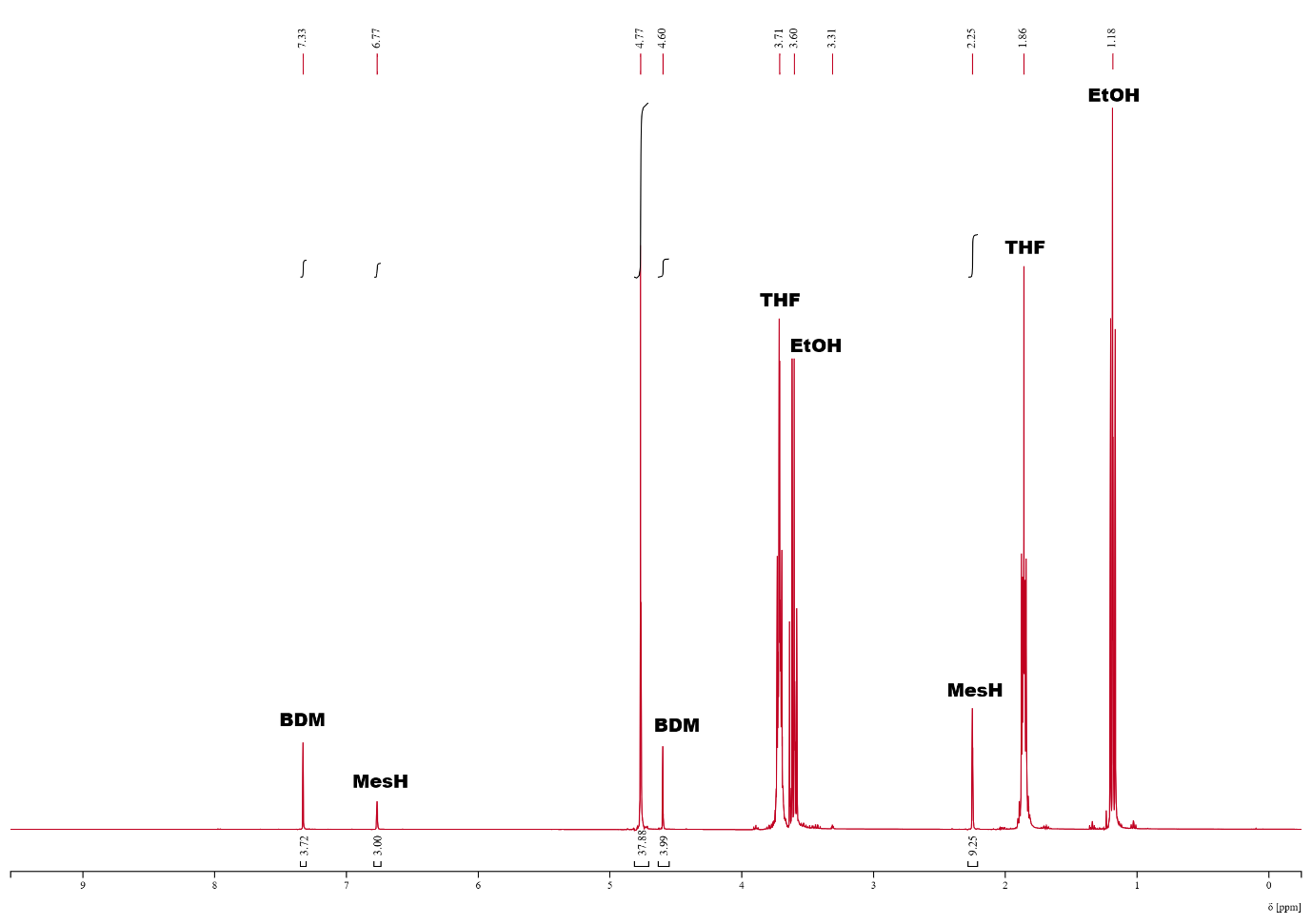


Figure S19. ^1^H NMR (400 MHz, methanol-d4) spectrum of reaction mixture from Entry 1, Table S3.


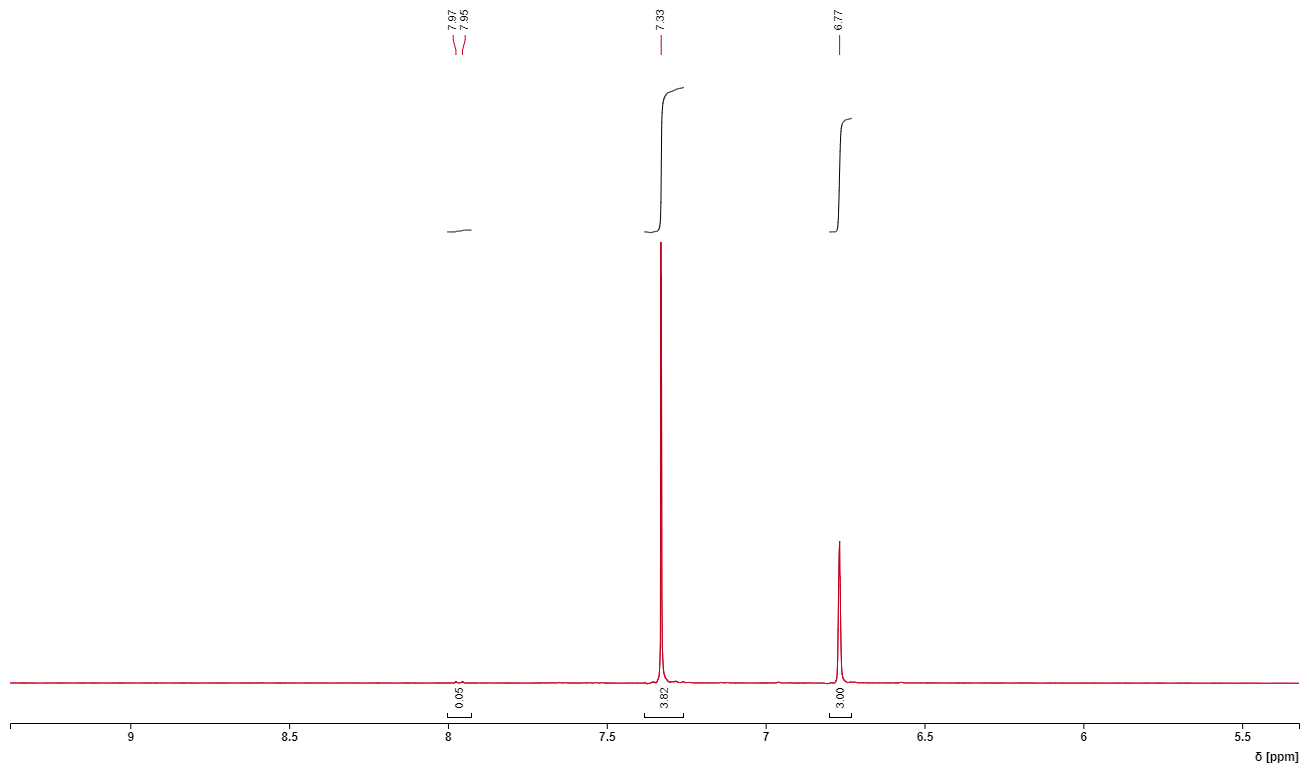


Figure S20. Fragment of ^1^H NMR (400 MHz, methanol-d4) spectrum of reaction mixture from Entry 1, Table S3.


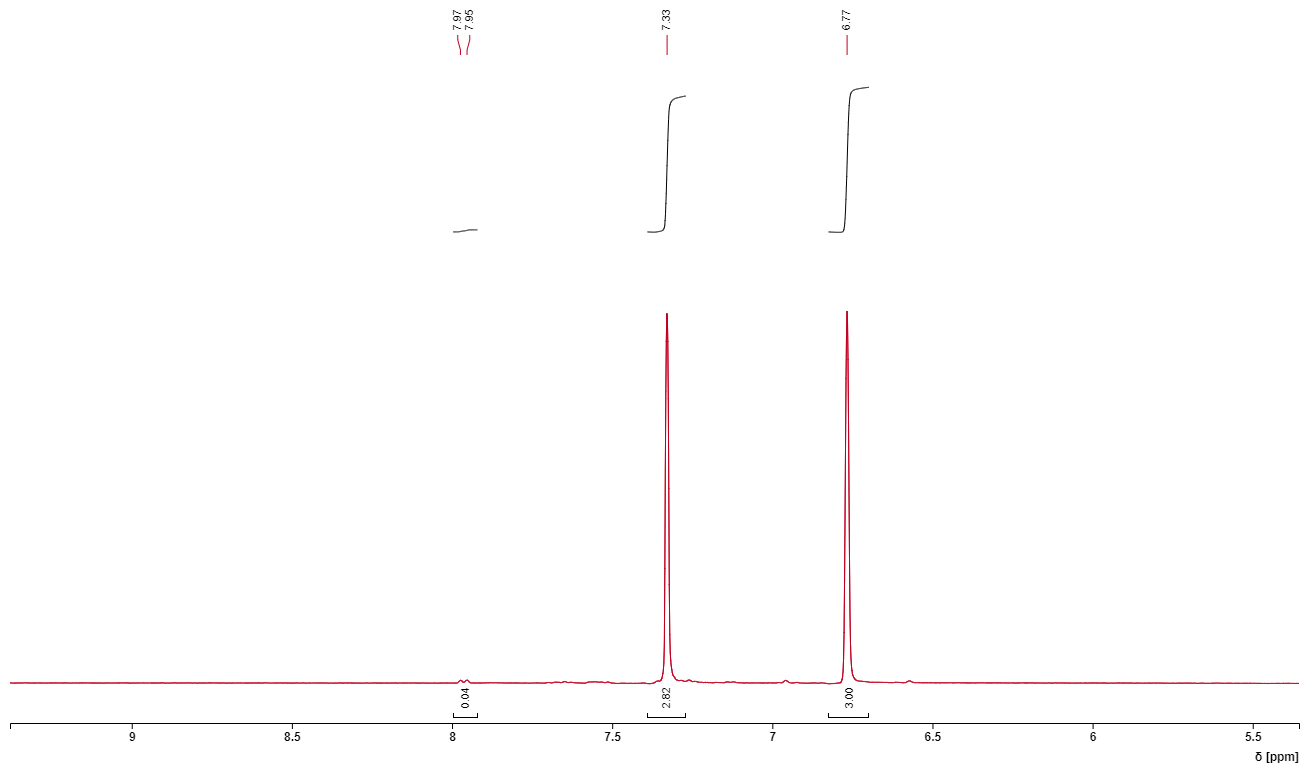


Figure S21. Fragment of ^1^H NMR (400 MHz, methanol-d4) spectrum of reaction mixture from Entry 2, Table S3.


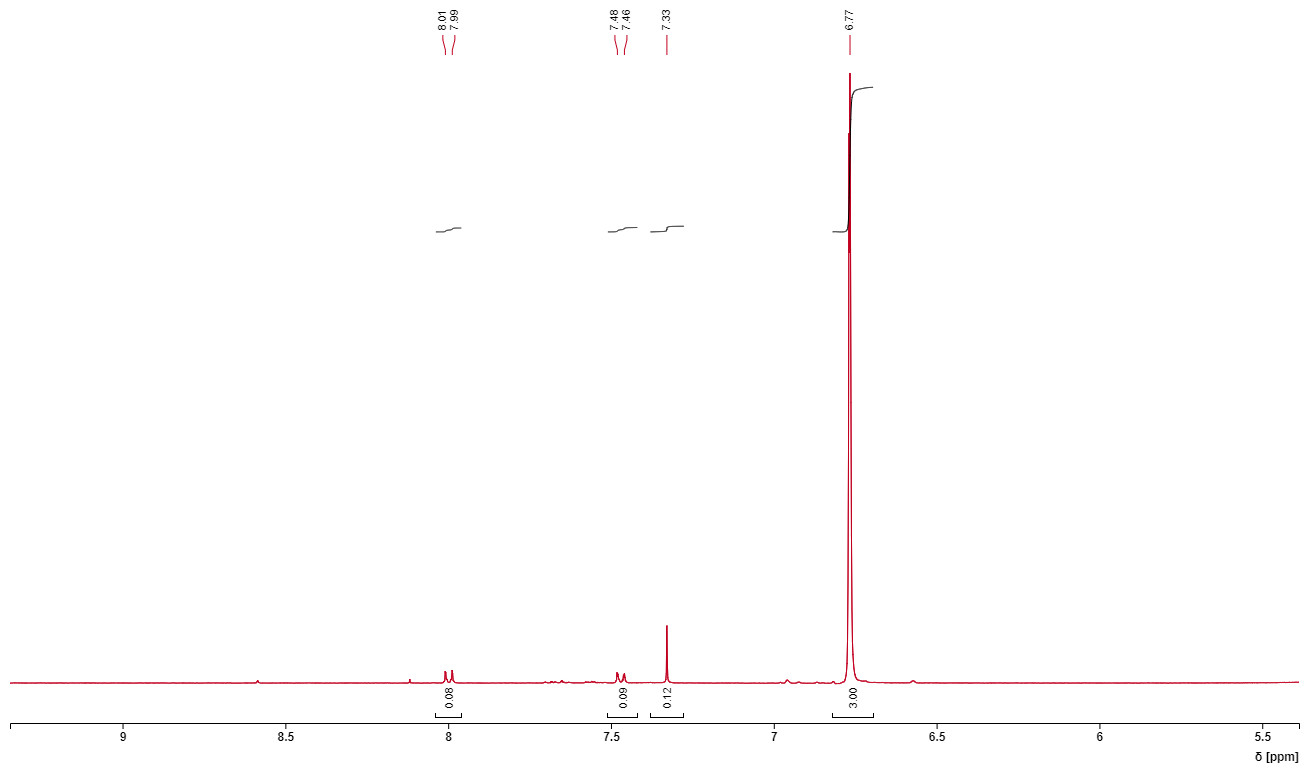


Figure S22. Fragment of ^1^H NMR (400 MHz, methanol-d4) spectrum of reaction mixture from Entry 3, Table S3.


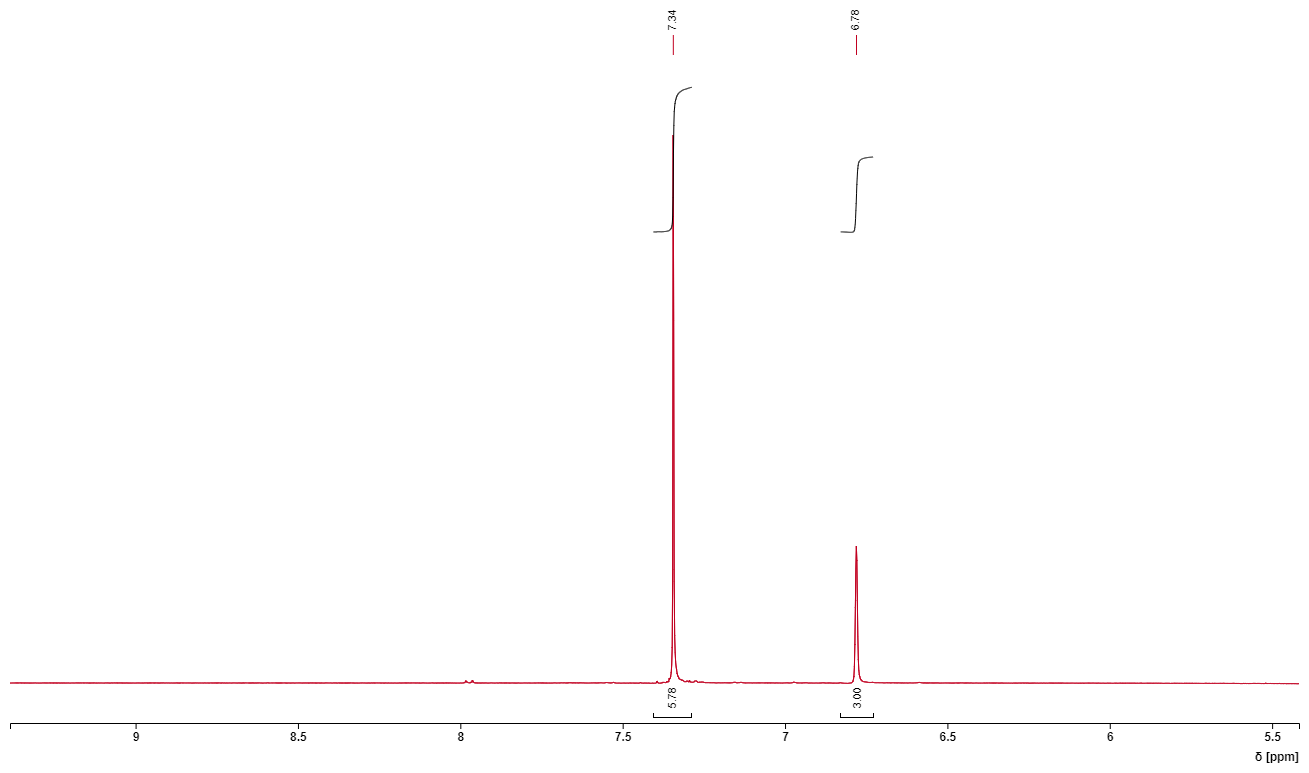


Figure S23. Fragment of ^1^H NMR (400 MHz, methanol-d4) spectrum of reaction mixture from Entry 4, Table S3.


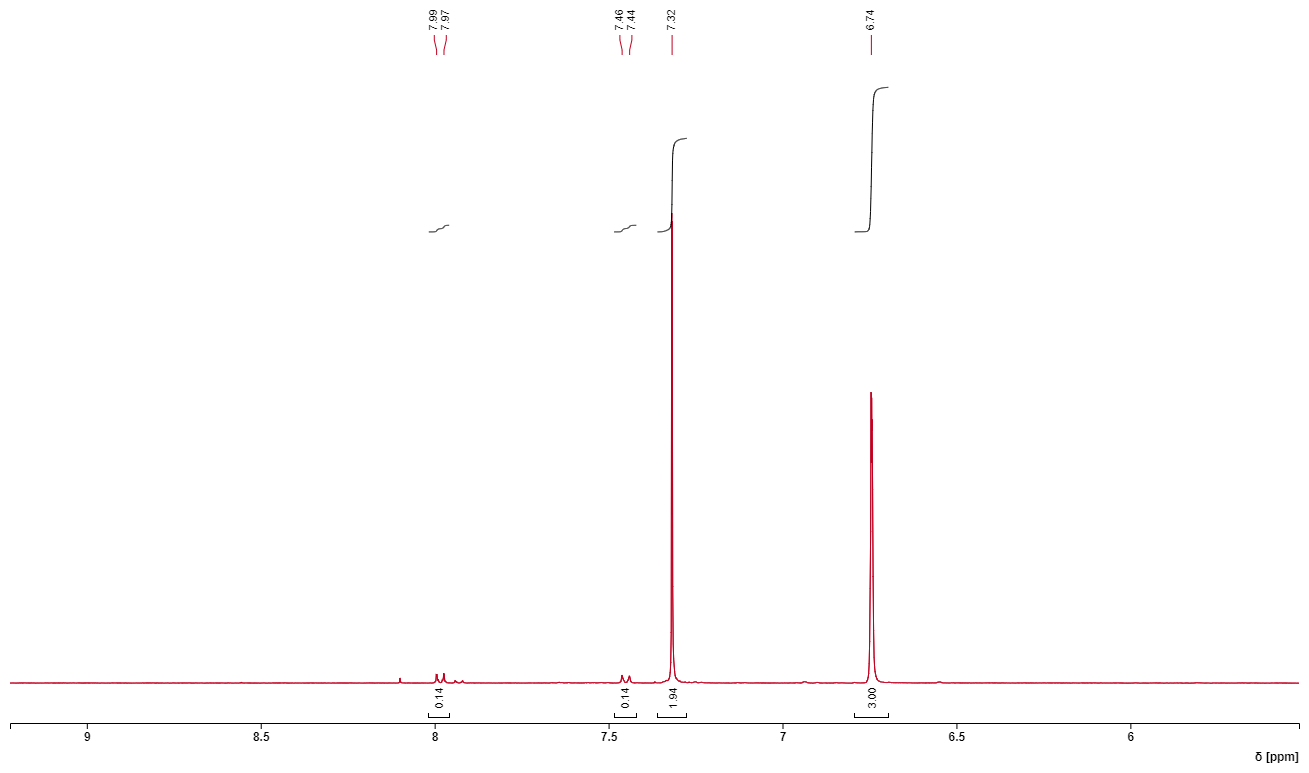


Figure S24. Fragment of ^1^H NMR (400 MHz, methanol-d4) spectrum of reaction mixture from Entry 5, Table S3.


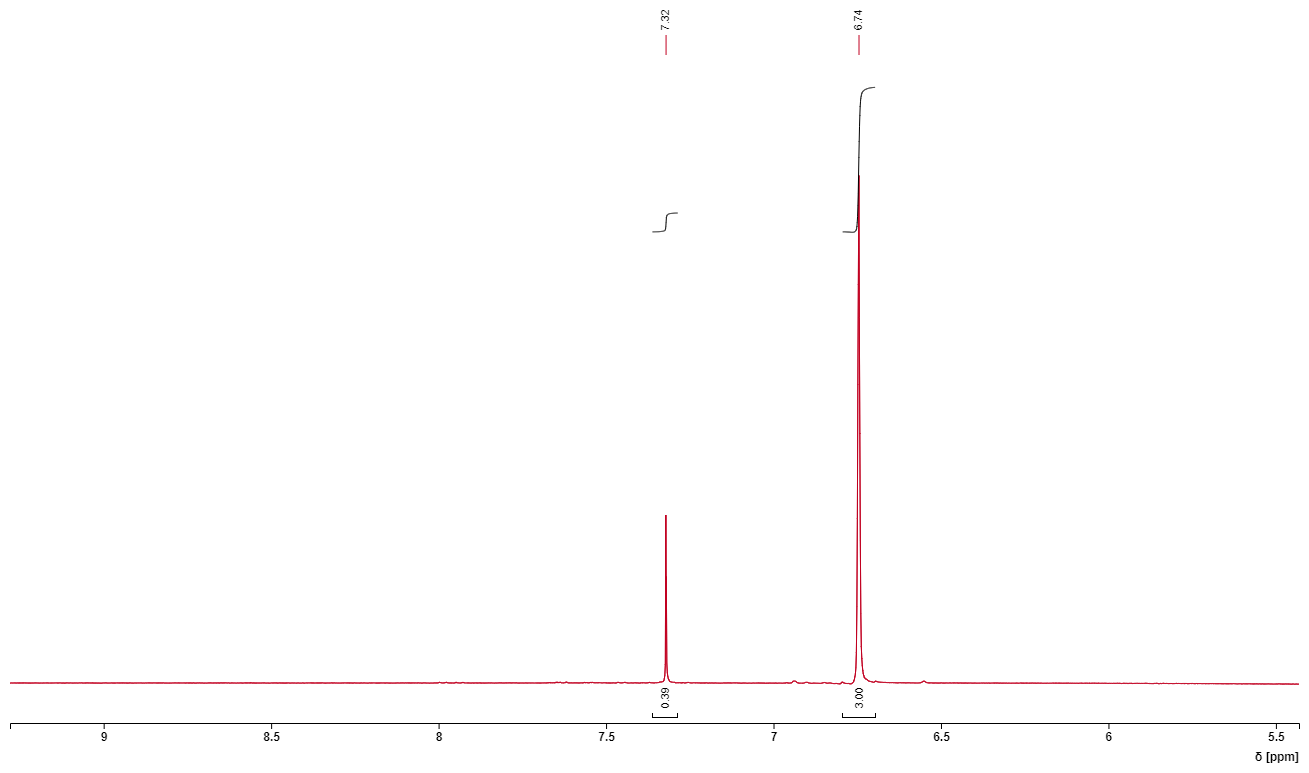


Figure S25. Fragment of ^1^H NMR (400 MHz, methanol-d4) spectrum of reaction mixture from Entry 6, Table S3.


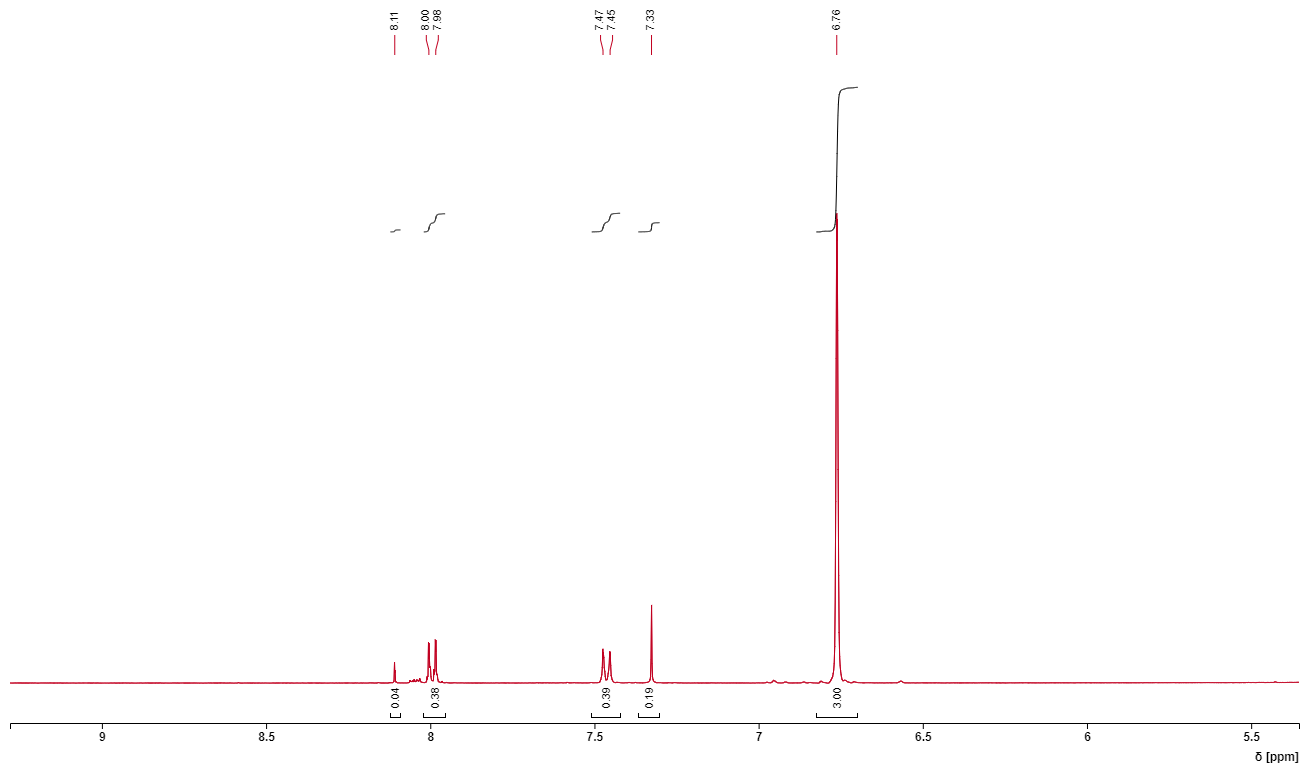


Figure S26. Fragment of ^1^H NMR (400 MHz, methanol-d4) spectrum of reaction mixture from Entry 7, Table S3.


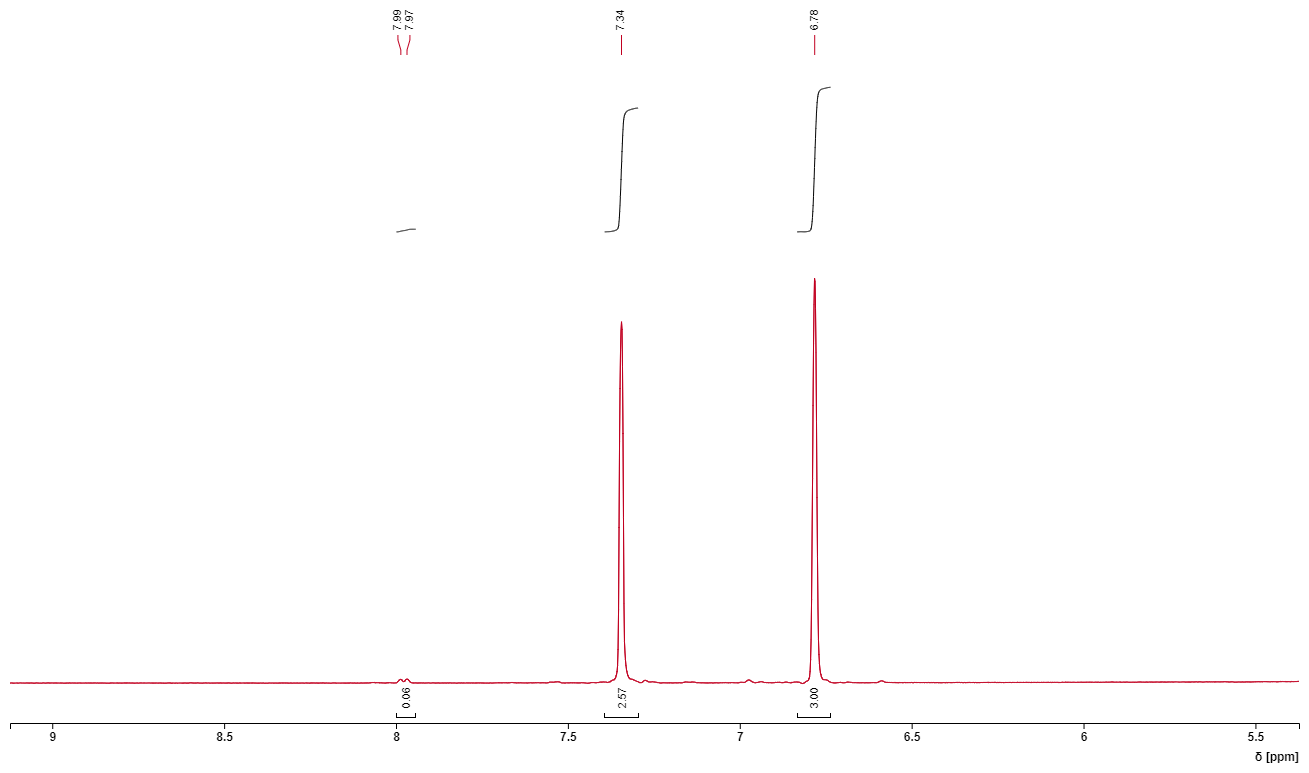


Figure S27. Fragment of ^1^H NMR (400 MHz, methanol-d4) spectrum of reaction mixture from Entry 8, Table S3.


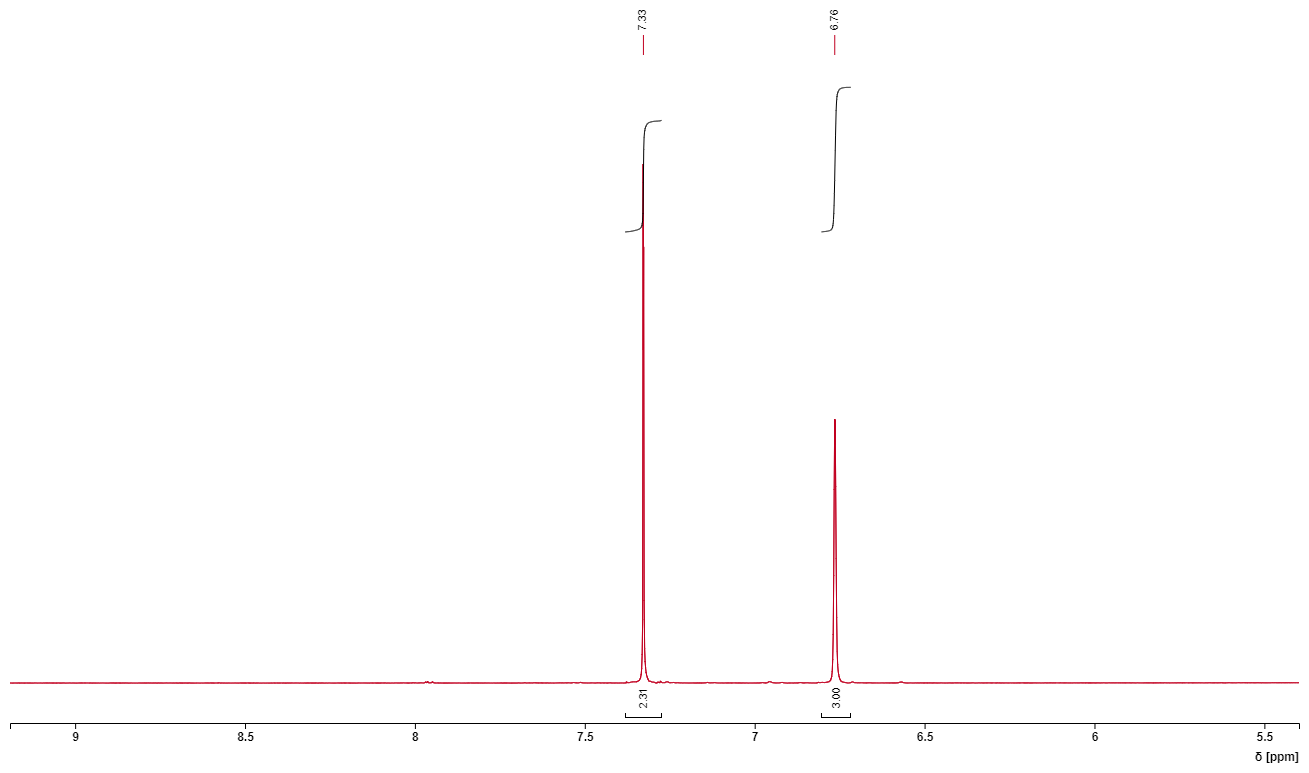


Figure S28. Fragment of ^1^H NMR (400 MHz, methanol-d4) spectrum of reaction mixture from Entry 9, Table S3.


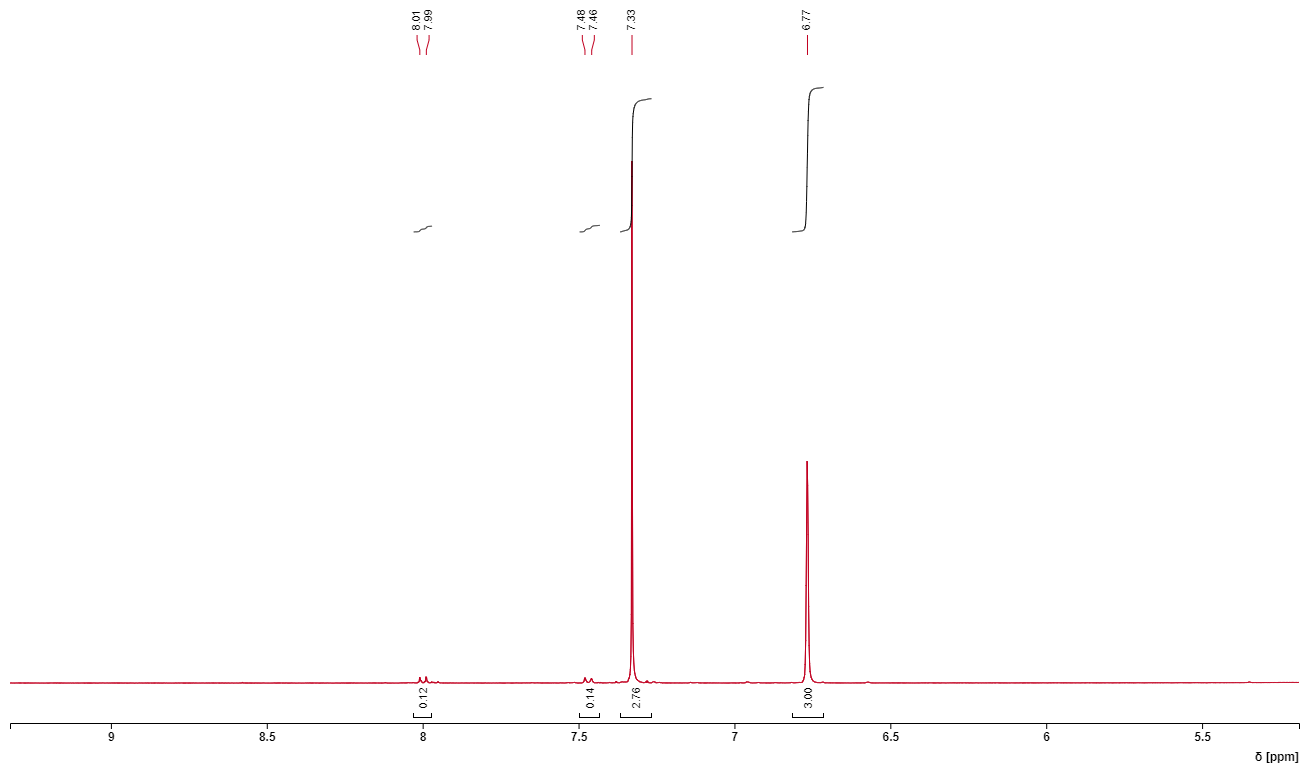


Figure S29. Fragment of ^1^H NMR (400 MHz, methanol-d4) spectrum of reaction mixture from Entry 10, Table S3.


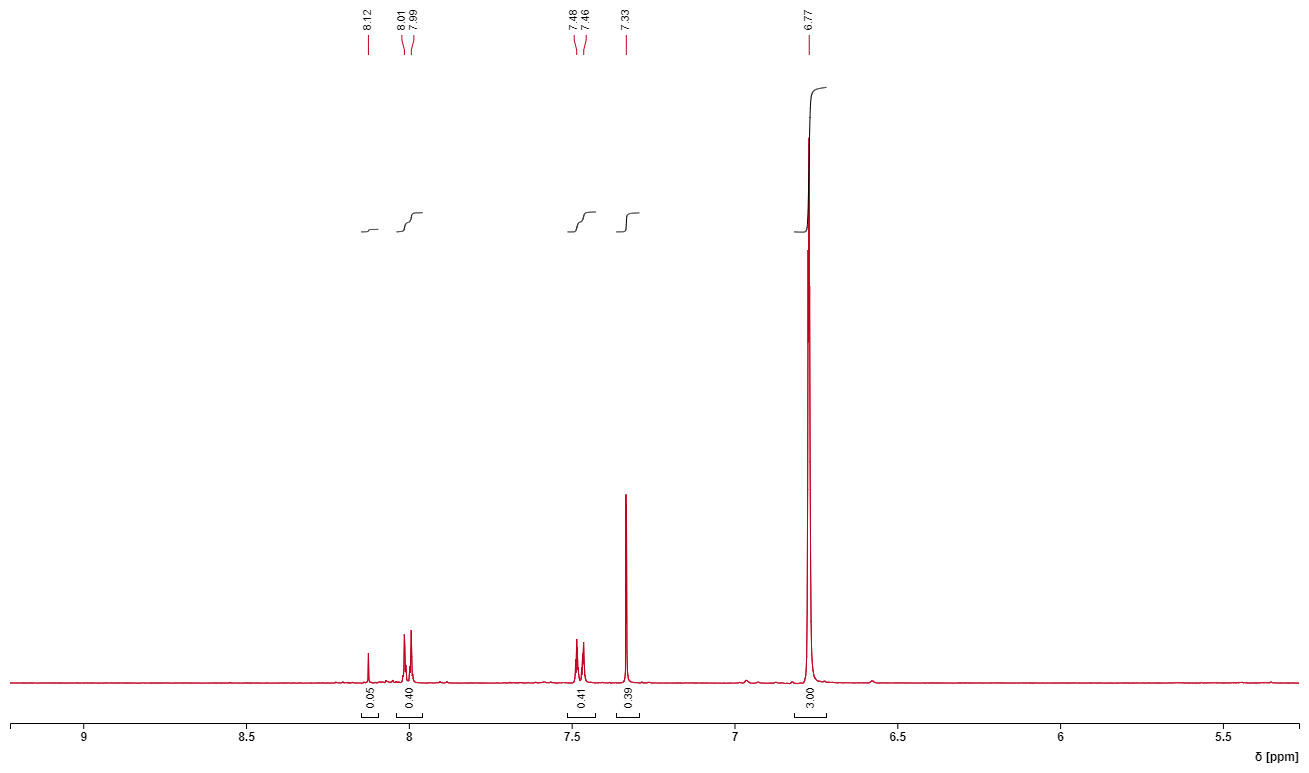


Figure S30. Fragment of ^1^H NMR (400 MHz, methanol-d4) spectrum of reaction mixture from Entry 11, Table S3.


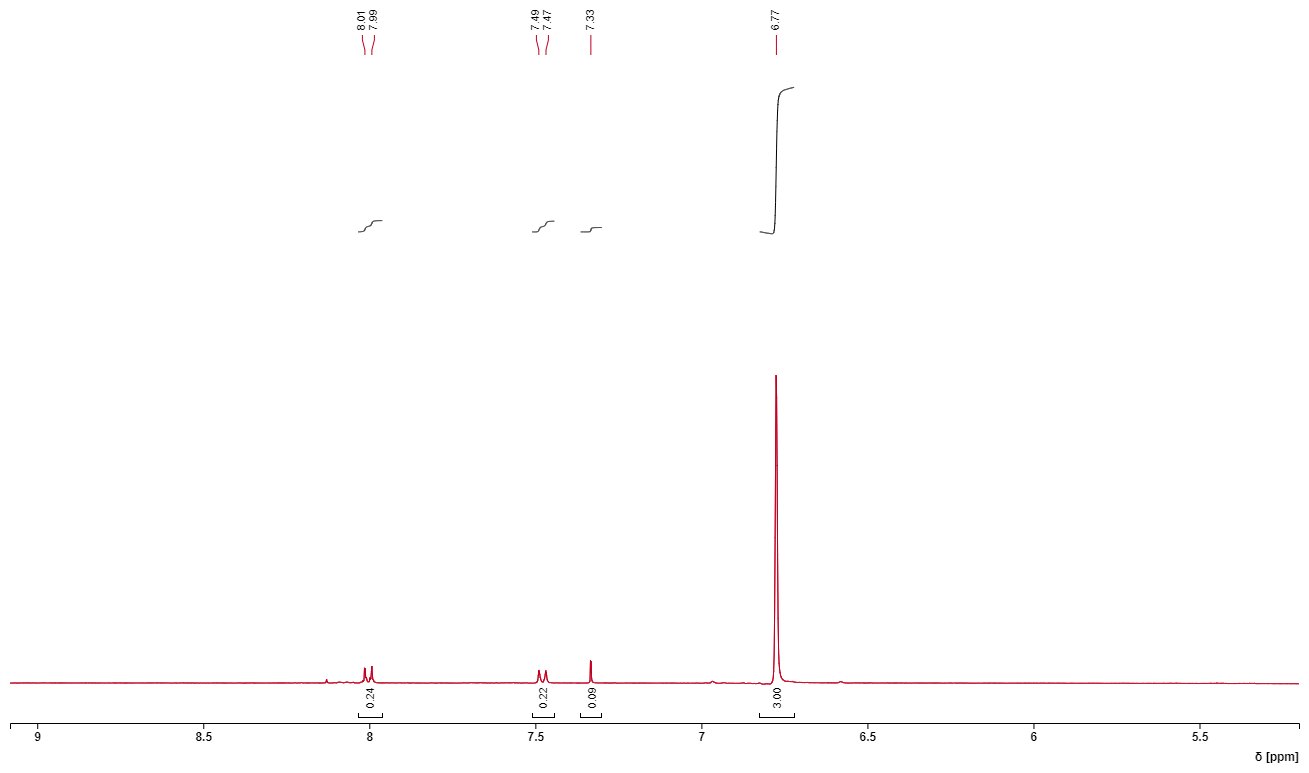


Figure S31. Fragment of ^1^H NMR (400 MHz, methanol-d4) spectrum of reaction mixture from Entry 12, Table S3.


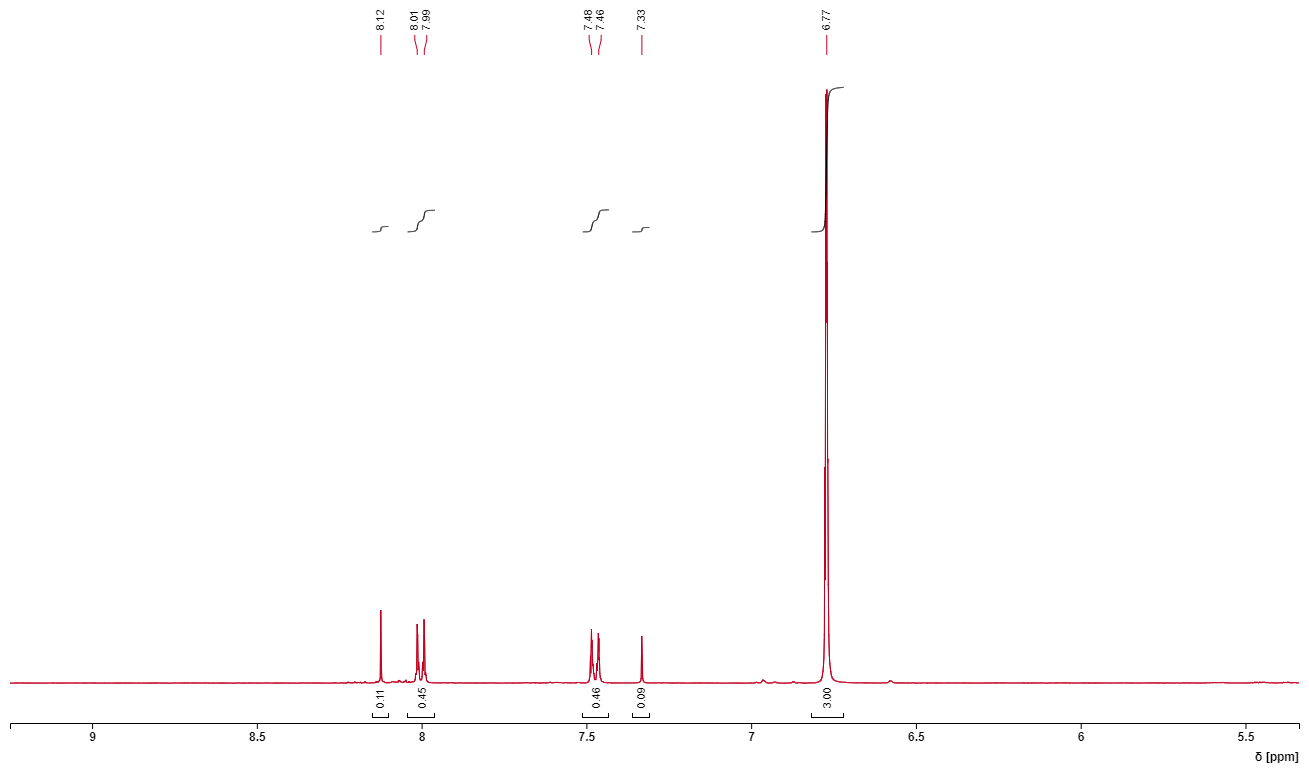


Figure S32. Fragment of ^1^H NMR (400 MHz, methanol-d4) spectrum of reaction mixture from Entry 13, Table S3.

### 2.3.2 Stage II.

From the results in Table S3, it was clear that the catalyst did not work at low catalytic loadings (0.1-0.2 mol%). We speculated it could be caused by moisture in the reaction since the base and precatalyst were weighed in air. Thus, further experiments with low catalyst loadings were repeated with base, polymer and catalyst weighted inside the Glovebox. Moreover, post-consumer PET sourced from plastic bottles (PET-b) was replaced by powdered PET (PET-p) purchased from GoodFellow to exclude any complications caused by additives that might be found in post-consumer PET. Expectedly, the yield improved, and the hydrogenation worked well with 0.1 mol% loadings (Table S4).

**General procedure D.**

PET-p (192.2 mg, 1 mmol, 1 eq. powder), KO*t*Bu (0.05 or 0.1 mmol, 5.6 or 11.2 mg, 5 or 10 mol%), and precatalyst **1** (1.3 or 0.6 mg, 0.2 or 0.1 mol%) were weighed in glovebox, placed into an 8 mL glass vial to which a stir bar was added, and the vial was sealed with a septum. The vial was taken out of the glovebox, connected to the Schlenk line, and solvents were added via syringe. Two needles were added at the top of the vial, after which it was placed inside a 150 mL autoclave with some metal beads to ensure thermal conductivity. The autoclave was purged with argon, then sealed, purged with H_2_, pressurized with H_2_ at 50 bar, and placed in an oil bath. The reaction was stirred at 80 °C for the specified time. After that, the autoclave was cooled down to room temperature in air and then in an ice bath and carefully vented to the atmosphere. After the reaction mixture was diluted with 2 mL of MeOH and stirred for 10 min at room temperature, the vial was weighed. The yield of the product was estimated by NMR spectroscopy using mesitylene as an internal standard. Hence, 200–300 mg of the reaction solution and ≈10 mg mesitylene were weighed in an NMR tube, followed by the addition of methanol-d4 (0.3 mL).

| **Table S4. Hydrogenation of PET with complex 1 in the glovebox.***^[^****^a]^***   | | | | | | | | | |
| --- | --- | --- | --- | --- | --- | --- | --- | --- | --- |
| № exp. | Start mat. | solvent | KOtBu, mol% | Cat. Mol% | Time, h | Conv.^[b]^ | Yield of BDM, % | Yield of EHMB, % | Yield of DETP, % |
| 1 | PET-p | 4.5 mL 2-MeTHF/0.5 mL EtOH | 5 | 0.2 | 18 | full | 93 | <1 | <1 |
| 2 | PET-p | 4.5 mL 2-MeTHF/0.5 mL EtOH | 5 | 0.1 | 18 | full | 80 | 10 | <1 |
| 3 | PET-p | 4.5 mL 2-MeTHF/0.5 mL EtOH | 10 | 0.1 | 18 | full | 92 | <1 | <1 |
| 4 | PET-p | 4.8 mL 2-MeTHF/0.2 mL EtOH | 10 | 0.1 | 18 | full | 95 | <1 | <1 |
| 5 | PET-p | 4.5 mL 2-MeTHF/0.5 mL EtOH | 5 | 0.1 | 43 | full | 93 | <1 | <1 |
| *[a]* General procedure D. *[b]* Conversion of polymer was calculated as a difference between plastic loading and solid residue after the reaction. | | | | | | | | | |


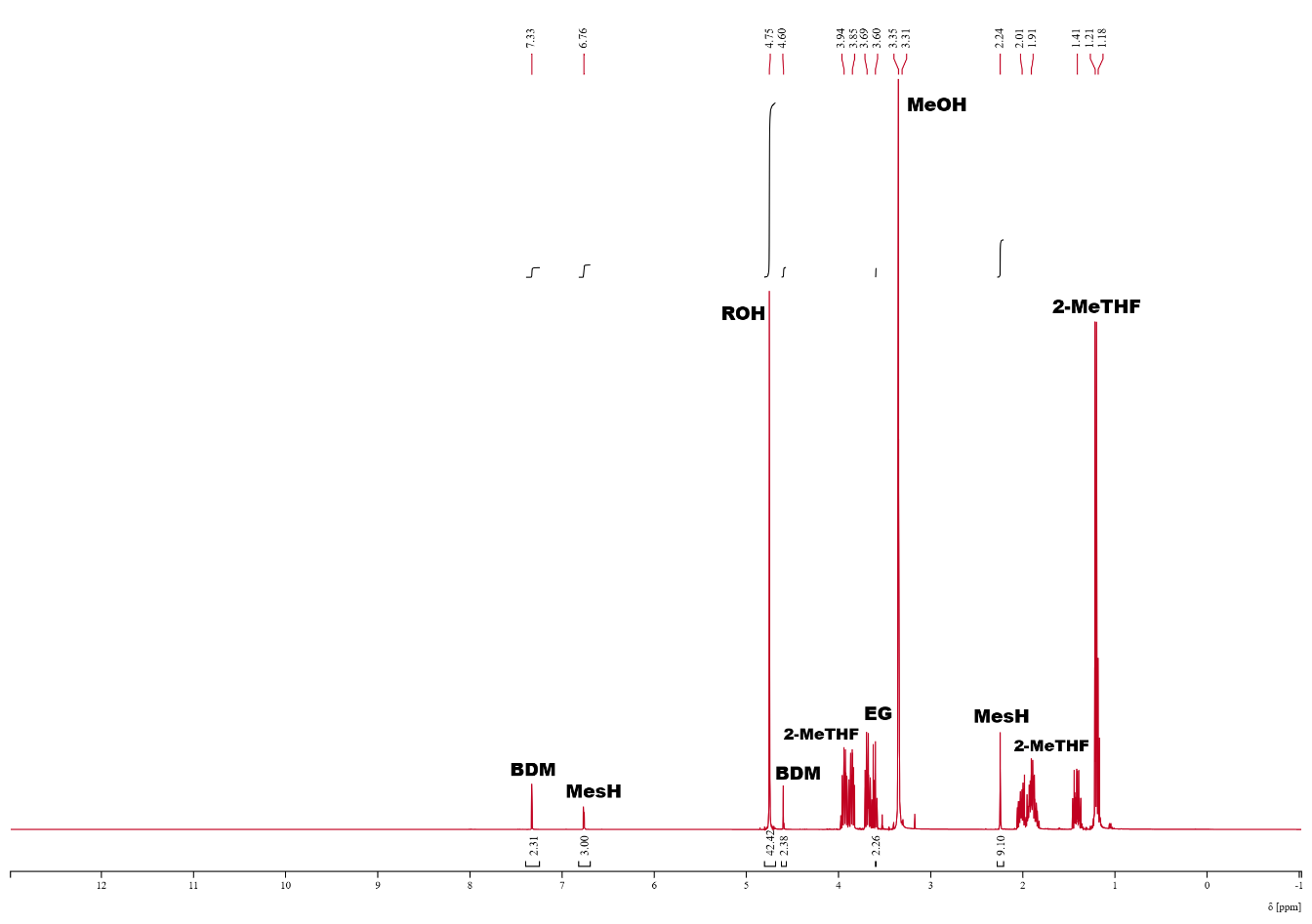


Figure S33. ^1^H NMR (400 MHz, methanol-d4) spectrum of reaction mixture from Entry 1, Table S4.


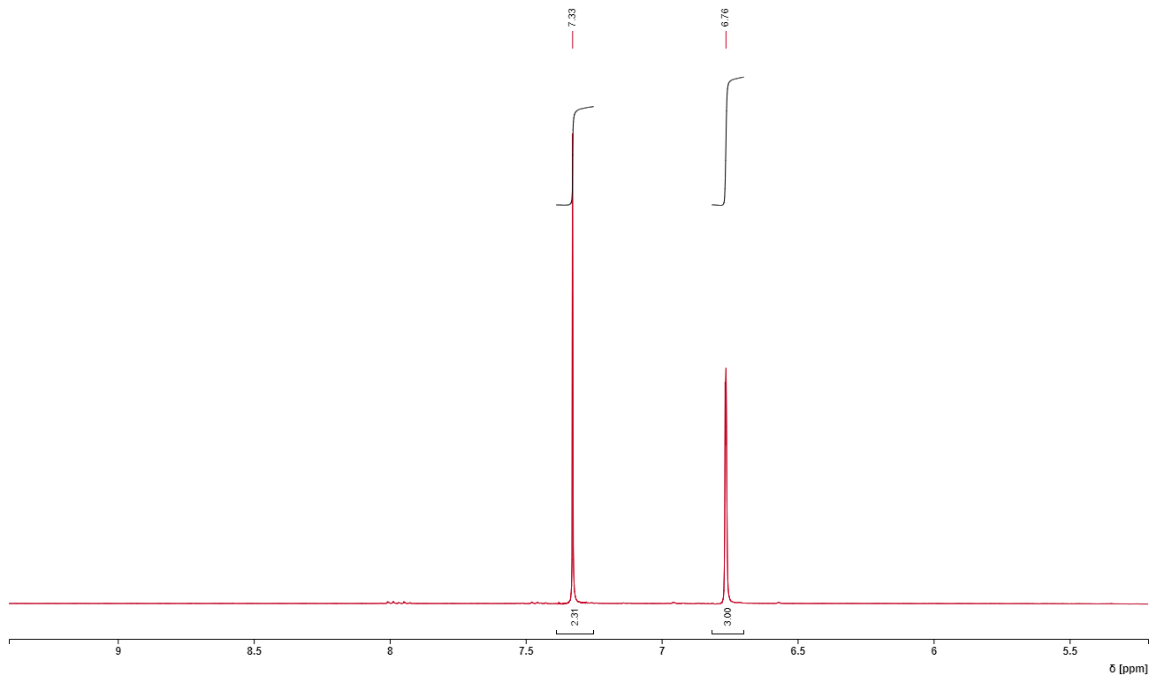


Figure S34. Fragment of ^1^H NMR (400 MHz, methanol-d4) spectrum of reaction mixture from Entry 1, Table S4.


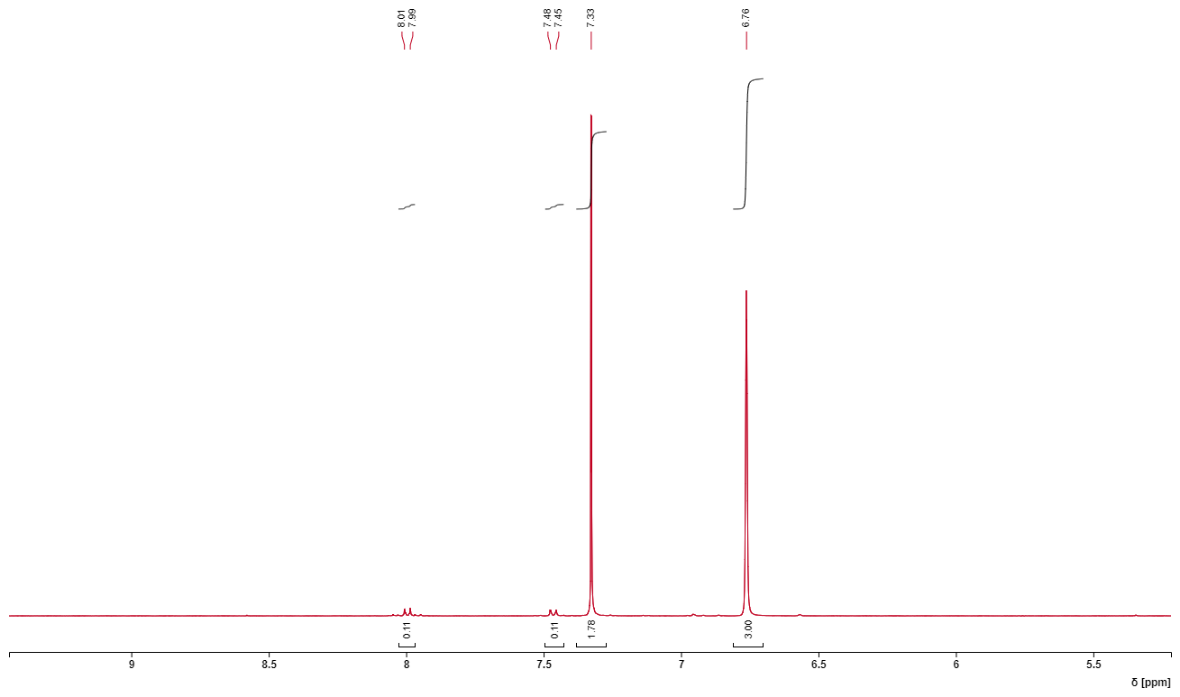


Figure S35. Fragment of ^1^H NMR (400 MHz, methanol-d4) spectrum of reaction mixture from Entry 2, Table S4.


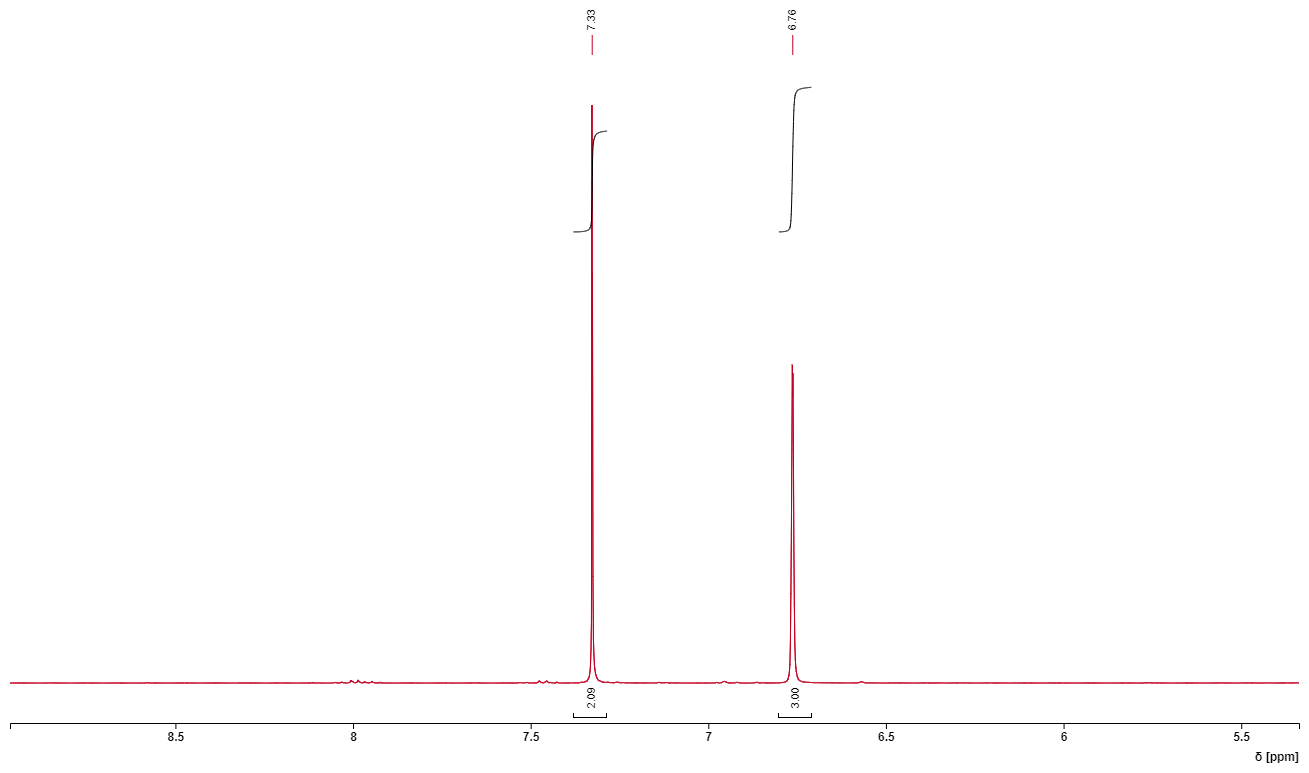


Figure S36. Fragment of ^1^H NMR (400 MHz, methanol-d4) spectrum of reaction mixture from Entry 3, Table S4.


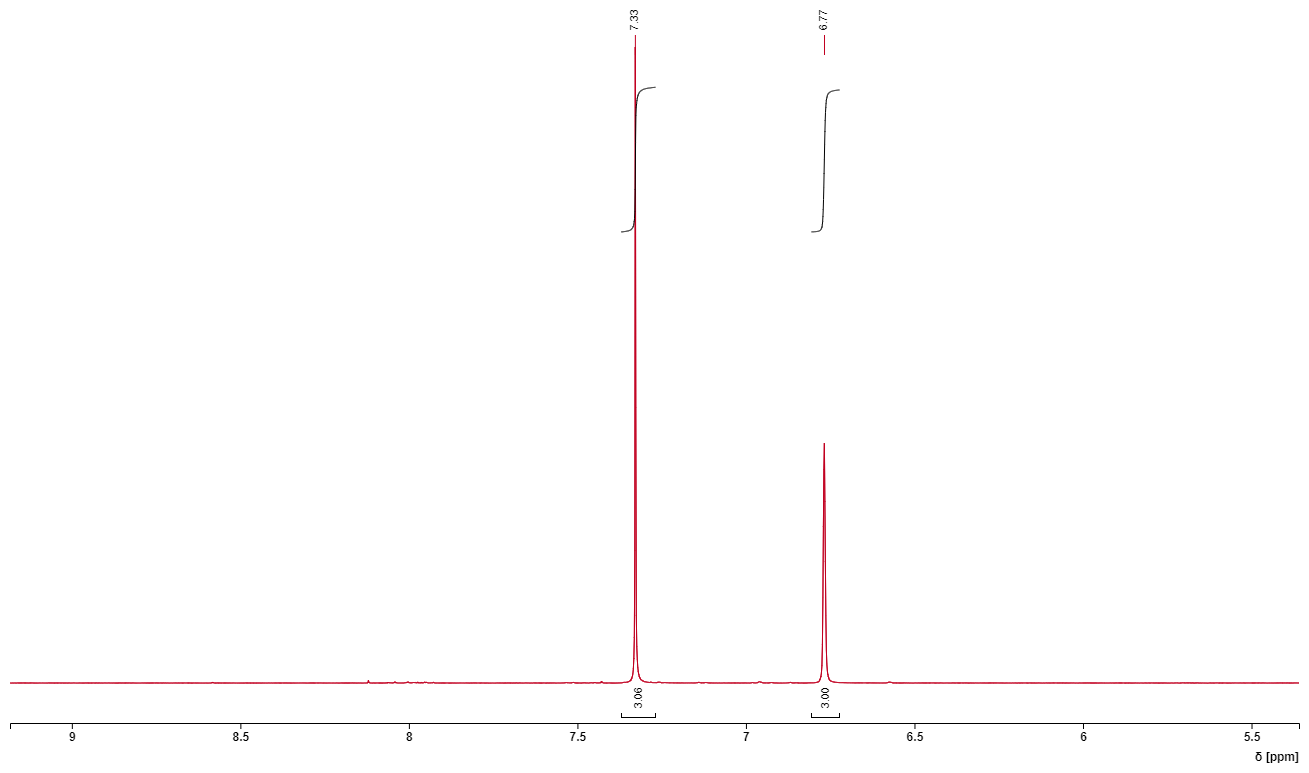


Figure S37. Fragment of ^1^H NMR (400 MHz, methanol-d4) spectrum of reaction mixture from Entry 4, Table S4.


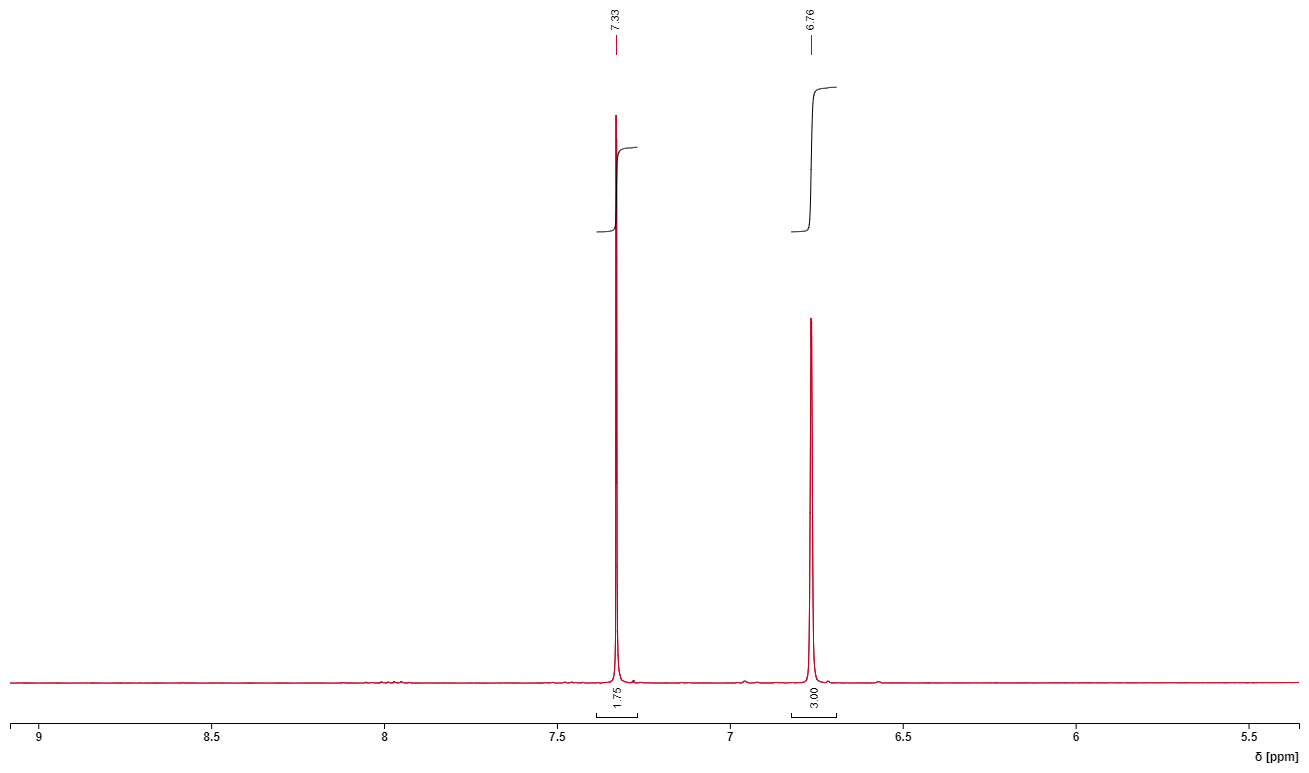


Figure S38. Fragment of ^1^H NMR (400 MHz, methanol-d4) spectrum of reaction mixture from Entry 5, Table S4.

### 2.3.3 Stage III.

To work with low catalytic loadings a stock solution of the precatalyst and base were prepared as shown in Scheme S1. First, precatalyst was dissolved in 2-MeTHF, giving a yellowish solution. Next, the solution of KO*t*Bu in THF (1M) was added via syringe. The resulting greenish solution was then transferred to reaction vial. Stock solutions were prepared fresh (always less than 1 h old) before each set of reactions.

Scheme S1. Preparation of stock solution of the catalyst and base.

A series of experiments demonstrated that different sources of PET may give a different yield (Table S5). The highest hydrogenation yield was obtained with polymer powder (PET-p), while polymer derived from a bottle (PET-b, post-consumer plastic) gave a lower hydrogenation yield. Polymer granules (PET-g) gave the lowest conversion which might be related to extremely dense material and low solubility.

Next, several experiments were carried out to see if the reaction works at lower pressure of hydrogen and lower catalytic loadings. Thus, the hydrogenation went slower at 20 bar of hydrogen which led to a higher content of monoester EHMB (Entry 4, Table S5). Despite full conversion of the polymer, hydrogenation at lower catalytic loadings at 50 bar of hydrogen gave low conversion of DETP to reduced products EHMB and BDM. It was suggested that the procedure for preparation of the stock catalyst solution was not optimal, and most of the active catalyst did not survive it. Various transformations with Ru-MACHO^®^ complex upon addition of base were previously reported by Schaub et al.^3^ Similar transformations could be possible in case of the Gusev’s complex **1**.

**General procedure E.**

Complex **1** (6.3 mg, 0.01 mmol) was weighed in air and placed into an 8 mL vial and sealed using a septum. The vial was purged with argon, and 2-MeTHF (0–4.5 mL) was added via syringe. To the resulting yellowish solution, KO*t*Bu (1 M solution, 0.5–5.0 mL, 0.5–5.0 mmol) was added. The greenish solution (0.002 M of the catalyst and 0.1–1.0 M of KO*t*Bu) was stirred at room temperature for 30 min. Polyethylene terephthalate (192.2 mg, 1 mmol, 1 eq.), was weighed under air, placed into an 8 mL glass vial containing a stir bar, and sealed using a septum. The vial was purged with argon, and 2-MeTHF (4.0–4.45 mL) and dry EtOH (0.5 mL) were added via syringe. Next, the prepared earlier solution of the catalyst and base (0.05–0.5 mL) was added into a sealed 8-mL vial via syringe under inert gas. Two needles were added at the top of the vial, after which it was placed inside a 150 mL autoclave with some metal beads to ensure thermal conductivity. The autoclave was purged with argon, then sealed, purged with H_2_ (20 bar), pressurized with H_2_ at 50 bar, and placed in an oil bath preheated to 80 °C. The reaction was stirred at 80 °C for the specified time. After that, the autoclave was cooled down to room temperature in air and then in an ice bath and carefully vented to the atmosphere. After the reaction mixture was diluted with 2 mL of MeOH and stirred for 10 min at room temperature, the vial was weighed. The yield of the product was estimated by NMR spectroscopy using mesitylene as an internal standard. Hence, 200-300 mg of the reaction solution and ≈10 mg mesitylene were weighed in an NMR tube, followed by the addition of methanol-d4 (0.3 mL).

| **Table S5. Hydrogenation of PET from various sources with complex 1.***^[^****^a]^***   | | | | | | | | | | |
| --- | --- | --- | --- | --- | --- | --- | --- | --- | --- | --- |
| № exp. | Start mat. | solvent | KOtBu, mol% | Cat **1**, mol% | Time | P(H_2_), bar | Conv.^[b]^ | Yield of BDM, % | Yield of EHMB, % | Yield of DETP, % |
| 1 | PET-p | 4.5 mL 2-MeTHF/0.5 mL EtOH | 5 | 0.1 | 18 h | 50 | full | 93 | 7 | <1 |
| 2 | PET-b | 4.5 mL 2-MeTHF/0.5 mL EtOH | 5 | 0.1 | 18 h | 50 | full | 75 | 21 | <1 |
| 3 | PET-g | 4.5 mL 2-MeTHF/0.5 mL EtOH | 5 | 0.1 | 18 h | 50 | 72% | 56 | 14 | <1 |
| 4 | PET-p | 4.5 mL 2-MeTHF/0.5 mL EtOH | 5 | 0.1 | 18 h | 20 | full | 69 | 29 | <1 |
| 5 | PET-p | 4.5 mL 2-MeTHF/0.5 mL EtOH | 5 | 0.05 | 40 h | 50 | full | <1 | 21 | 69 |
| 6 | PET-p | 4.5 mL 2-MeTHF/0.5 mL EtOH | 5 | 0.01 | 70 h | 50 | full | <1 | 12 | 62 |
| 7 | PET-p | 4.5 mL 2-MeTHF/0.5 mL EtOH | 5 | 0.01 | 7 days | 50 | full | <1 | 13 | 66 |
| *[a]* General Procedure E. *[b]* Conversion of polymer was calculated as a difference between plastic loading and solid residue after the reaction. | | | | | | | | | | |


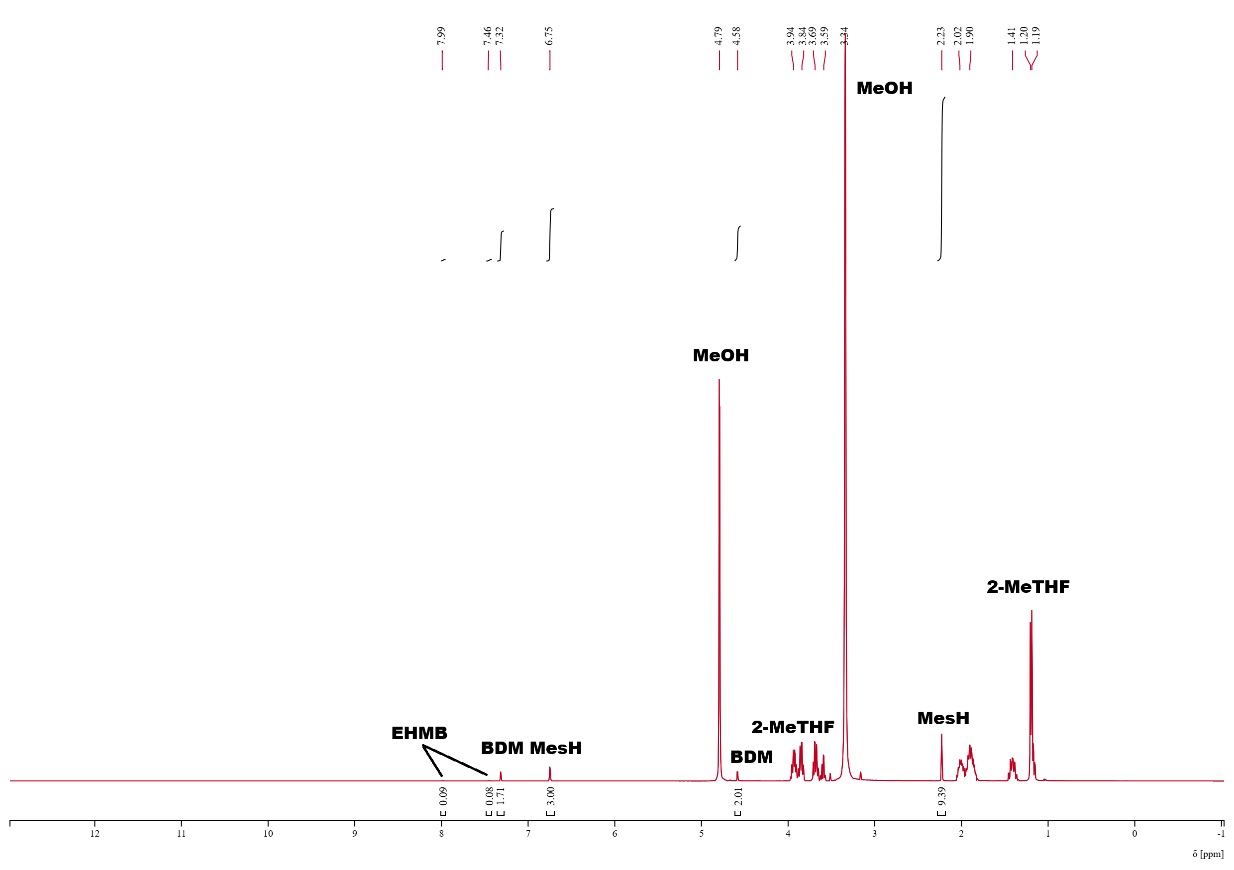


Figure S39. ^1^H NMR (400 MHz, methanol-d4) spectrum of reaction mixture from Entry 1, Table S5.


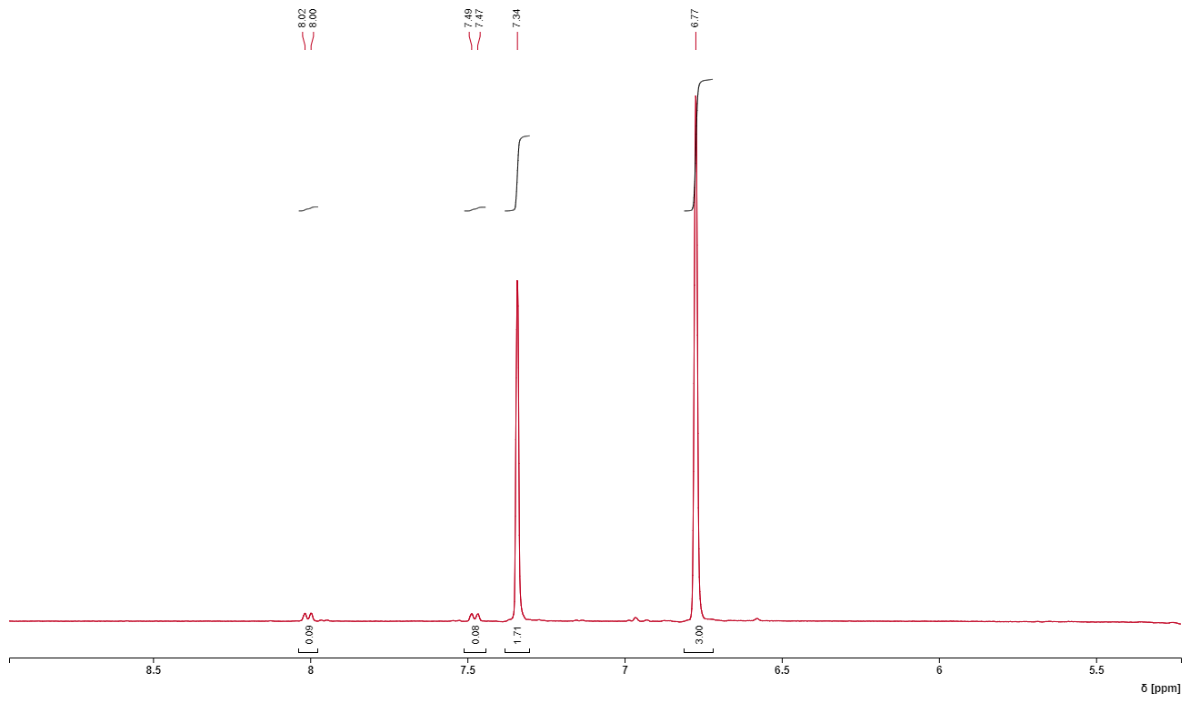


Figure S40. Fragment of ^1^H NMR (400 MHz, methanol-d4) spectrum of reaction mixture from Entry 1, Table S5.


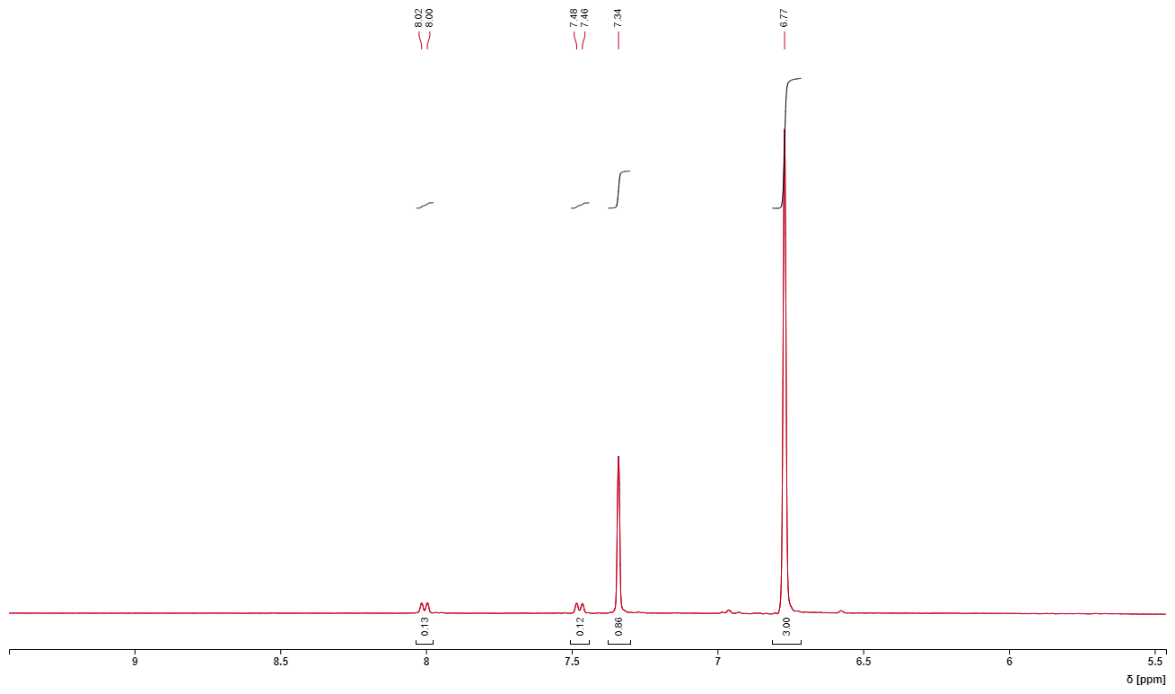


Figure S41. Fragment of ^1^H NMR (400 MHz, methanol-d4) spectrum of reaction mixture from Entry 2, Table S5.


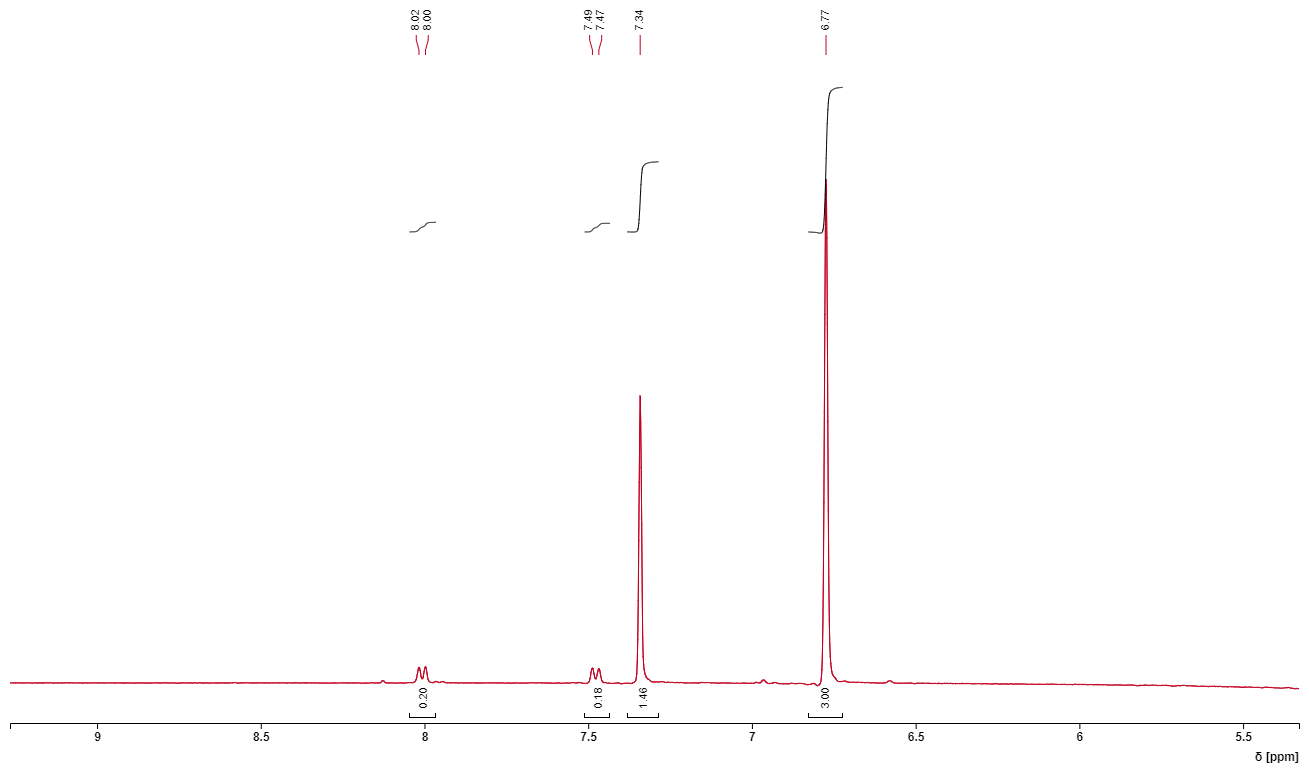


Figure S42. Fragment of ^1^H NMR (400 MHz, methanol-d4) spectrum of reaction mixture from Entry 3, Table S5.


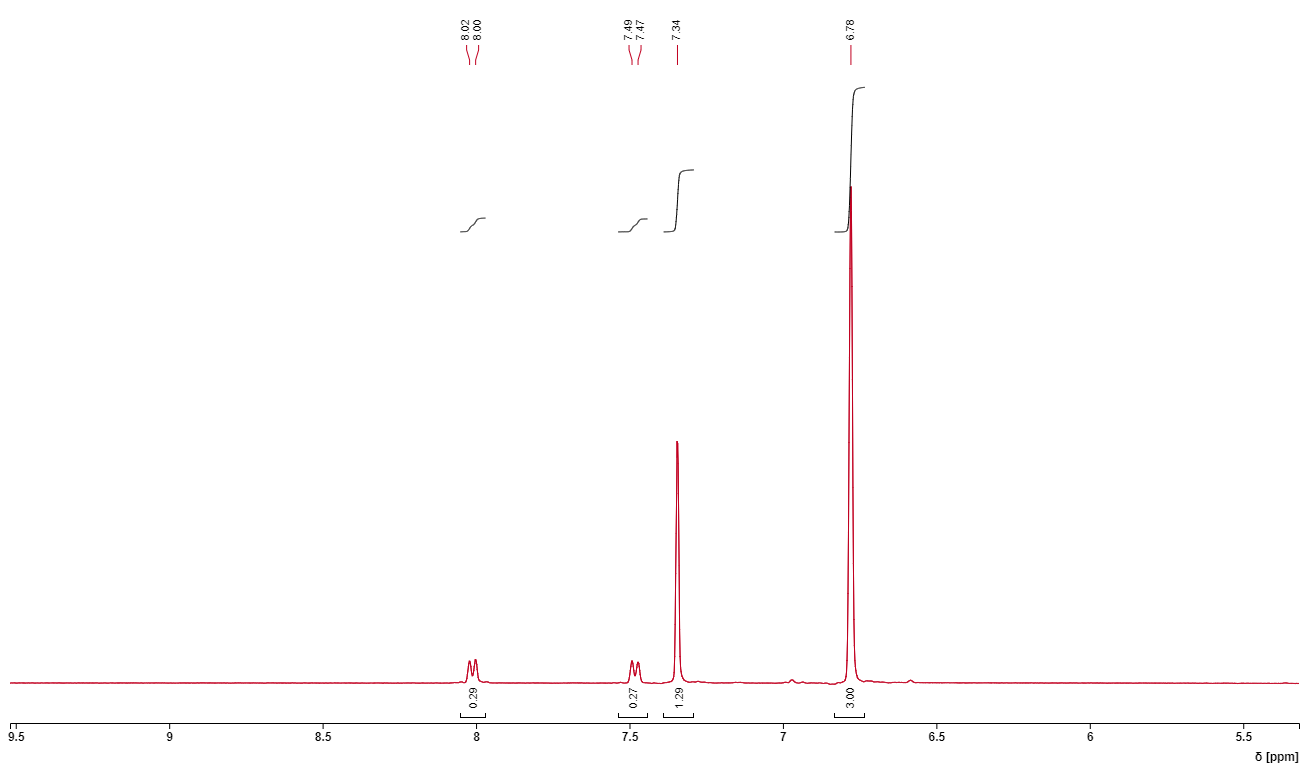


Figure S43. Fragment of ^1^H NMR (400 MHz, methanol-d4) spectrum of reaction mixture from Entry 4, Table S5.


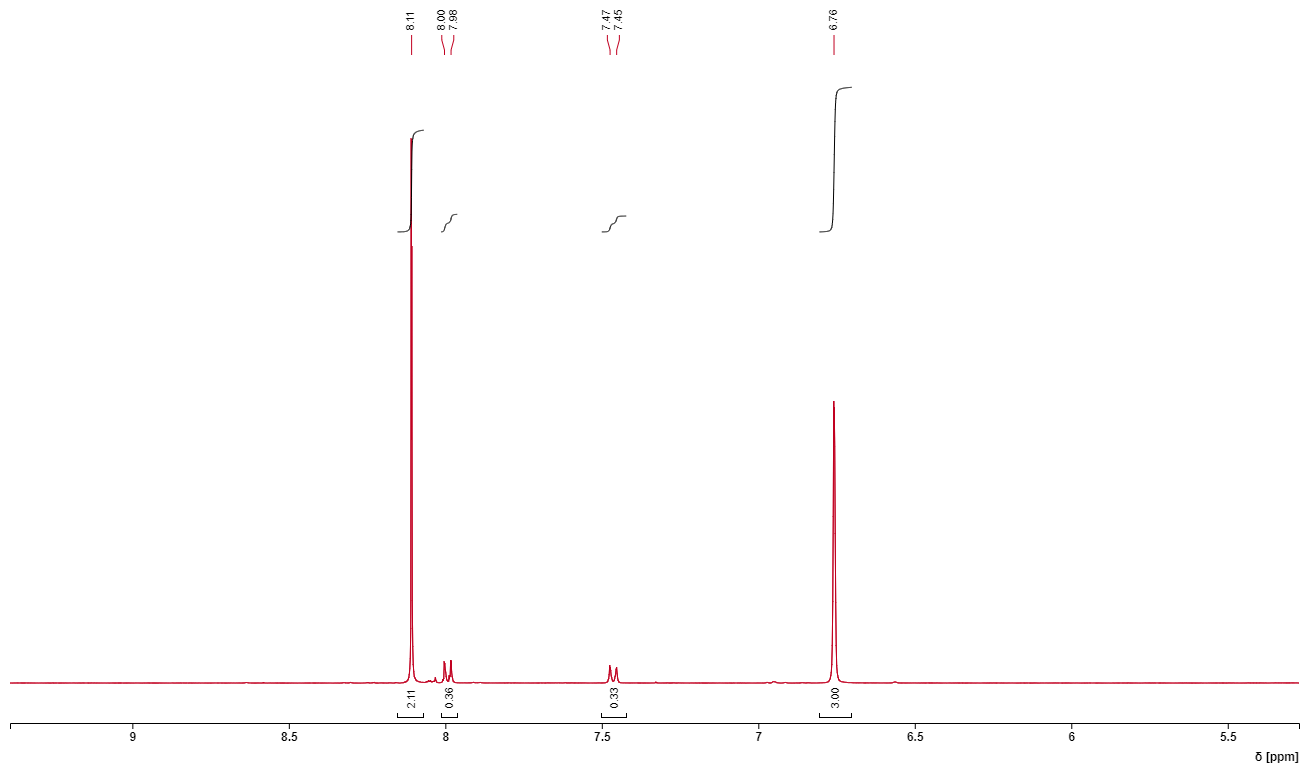


Figure S44. Fragment of ^1^H NMR (400 MHz, methanol-d4) spectrum of reaction mixture from Entry 5, Table S5.


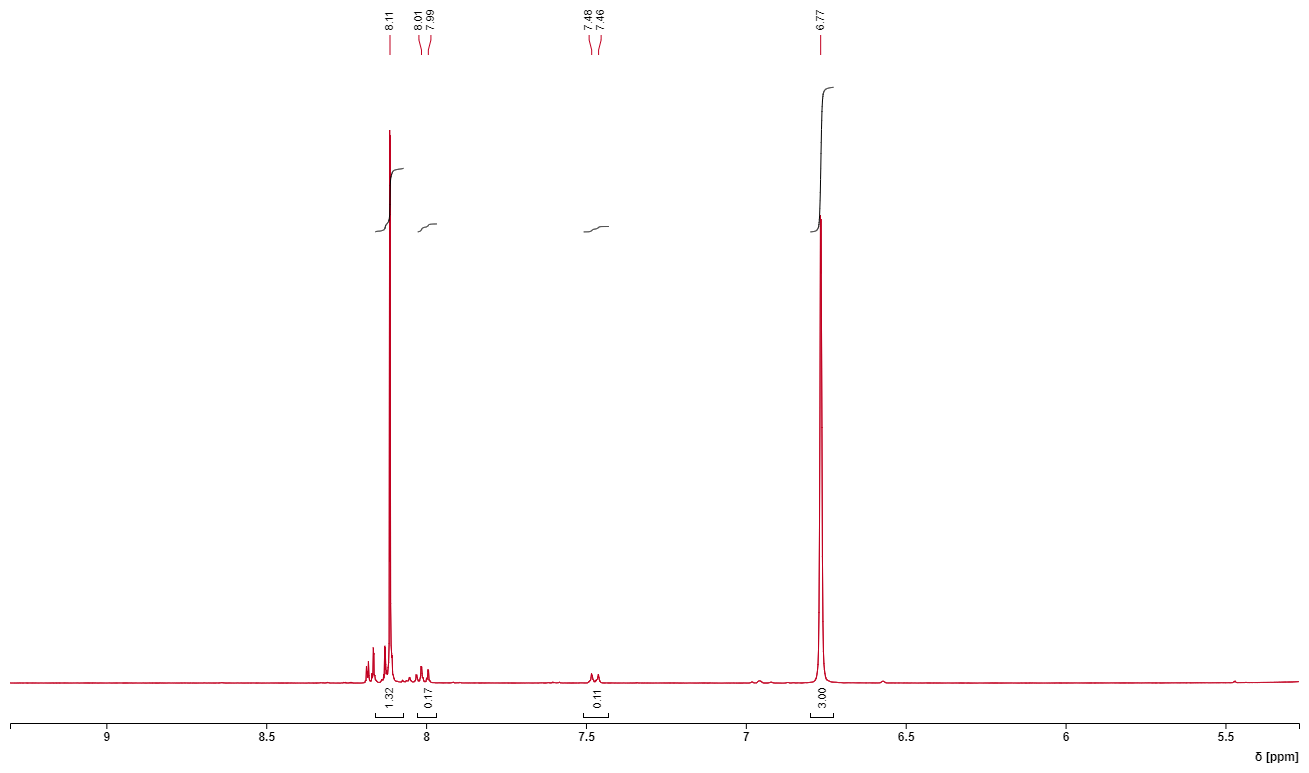


Figure S45. Fragment of ^1^H NMR (400 MHz, methanol-d4) spectrum of reaction mixture from Entry 6, Table S5.


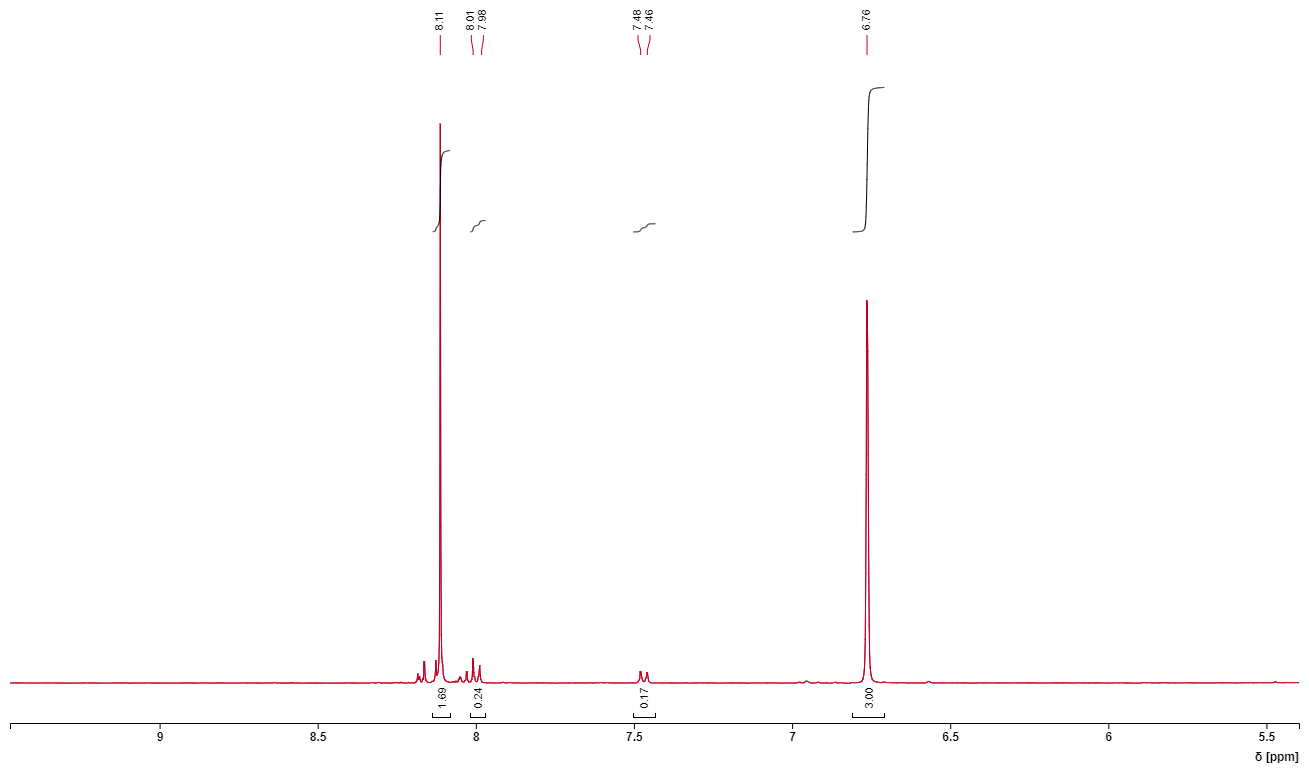


Figure S46. Fragment of ^1^H NMR (400 MHz, methanol-d4) spectrum of reaction mixture from Entry 7, Table S5.

### 2.3.4 Stage IV.

Gusev et al. reported that complex **1** gives a thermally stable ethoxide complex upon heating in basic ethanol^4^ hence preparation procedure of the stock solution was changed accordingly and carried out in the presence of ethanol (Scheme S2). First, precatalyst was dissolved in 2-MeTHF/EtOH mixture, giving a yellowish solution. Next, the solution of 100 eq. KO*t*Bu in THF (1 M) was added via syringe. The resulting yellowish solution was then transferred to the reaction vial. Stock solutions were prepared fresh (always less than 1 h old) before each set of reactions.

**

Scheme S2. Preparation of a stock solution of the catalyst and base.

After the new procedure had been applied, the catalyst became more active towards hydrogenation (Table S6). Thus, hydrogenation with 0.05% of **1** gave 57% yield of the BDM and 37% yield of the EHMB for 18 h (Entry 1, Table S6). Using post-consumer PET-b gave comparable result (Entry 2, Table S6) Lower catalyst and KO*t*Bu loadings (Entry 7, Table S6), increase of the ethanol concentration (Entries 3, 4, Table S6), and lower reaction temperature (Entry 6, Table S6) led to the higher selectivity towards EHMB but lower conversion of ester groups.

**General procedure F.**

Complex **1** (6.3 mg, 0.01 mmol) was weighed under air and placed into an 8 mL vial and sealed using septum. The vial was purged with argon, and 2-MeTHF (3.5 mL) and EtOH (0.5 mL) were added via syringe. To the resulting yellowish solution, KO*t*Bu (1M solution, 1 mL, 1 mmol) was added. The yellowish solution (0.002 M of the catalyst and 0.2 M of KOtBu) was stirred at room temperature for 30 min. Polyethylene terephthalate (192.2 mg, 1 mmol, 1 eq.), was weighed under air, placed into an 8 mL glass vial with a stir bar (vial and stir bar should be weighted), and sealed. The vial was purged with argon, and 2-MeTHF (4.25 mL) and EtOH (0.5 mL) were added via syringe. Next, the needed amount of the prepared earlier solution of the catalyst and base was added into the sealed 8 mL vial (containing PET) via syringe under inert gas. Two needles were added on top, and the vial was placed inside a 150 mL autoclave with some metal beads to ensure thermal conductivity. The autoclave was purged with argon, then sealed, purged with H_2_ (20 bar), pressurized with H_2_ at 50 bar, and placed in an oil bath heated to 80 °C. The reaction was stirred at 80 °C for the specified time. After that, the autoclave was cooled down to room temperature in air and then in an ice bath and carefully vented to atmosphere. After the reaction mixture was diluted with 2 mL of MeOH and stirred for 10 min at room temperature, the vial was weighed. The yield of the product was estimated by NMR spectroscopy using mesitylene as an internal standard. Hence, 200-300 mg of the reaction solution and ≈10 mg mesitylene were weighed in an NMR tube, followed by the addition of methanol-d4 (0.3 mL).

| **Table S6. Hydrogenation of PET with complex 1.***^[^****^a]^***   | | | | | | | | | |
| --- | --- | --- | --- | --- | --- | --- | --- | --- | --- |
| № exp. | Start mat. | solvent | KOtBu, mol% | Cat 1, mol% | T, ºC | Time, h | Yield of BDM, % | Yield of EHMB, % | Yield of DETP, % |
| 1 | PET-p | 4.5 mL 2-MeTHF/0.5 mL EtOH | 5 | 0.05 | 80 | 18 | 57 | 37 | <1 |
| 2 | PET-b | 4.5 mL 2-MeTHF/0.5 mL EtOH | 5 | 0.05 | 80 | 18 | 45 | 52 | <1 |
| 3 | PET-p | 3.5 mL 2-MeTHF/0.5 mL EtOH | 5 | 0.05 | 80 | 18 | 37 | 51 | <1 |
| 4 | PET-p | 3.0 mL 2-MeTHF/1.0 mL EtOH | 5 | 0.05 | 80 | 18 | 16 | 73 | 2 |
| 5 | PET-p | 4.5 mL 2-MeTHF/0.5 mL EtOH | 5 | 0.05 | 70 | 18 | 56 | 44 | <1 |
| 6 | PET-p | 4.5 mL 2-MeTHF/0.5 mL EtOH | 5 | 0.05 | 80 | 115 | 70 | 22 | <1 |
| 7 | PET-p | 4.5 mL 2-MeTHF/0.5 mL EtOH | 3 | 0.03 | 80 | 115 | 16 | 70 | 2 |
| *[a]* General procedure F. | | | | | | | | | |


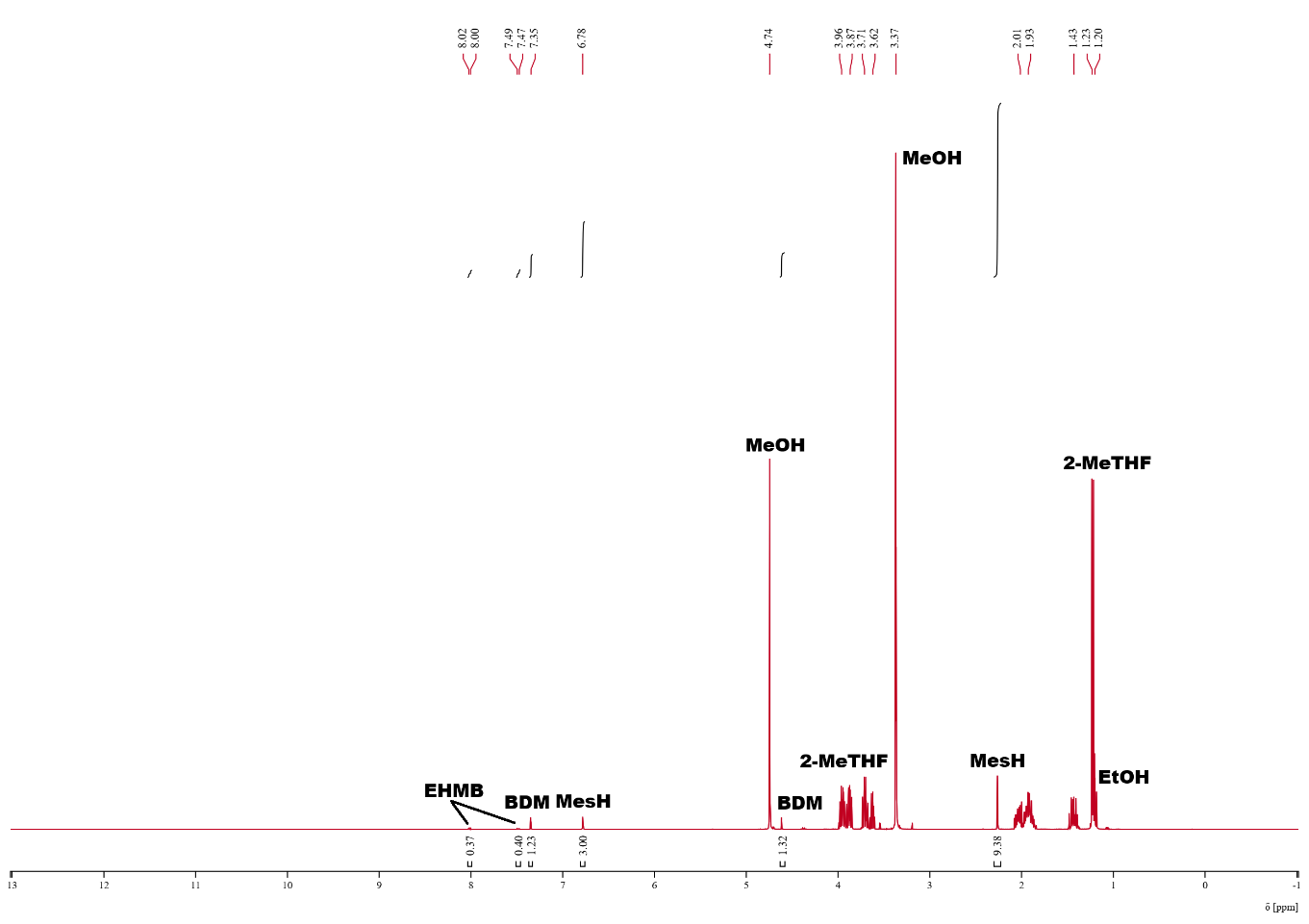


Figure S47. ^1^H NMR (400 MHz, methanol-d4) spectrum of reaction mixture from Entry 1, Table S6.


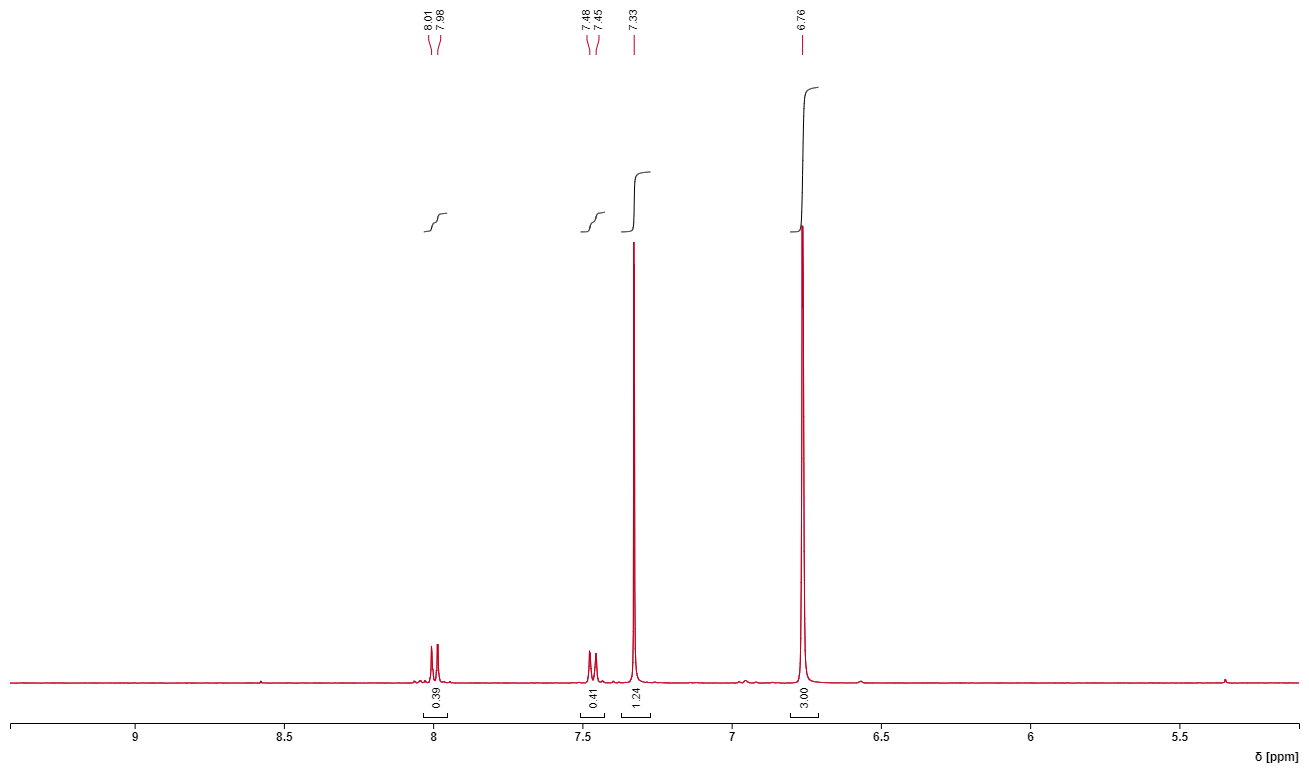


Figure S48. Fragment of ^1^H NMR (400 MHz, methanol-d4) spectrum of reaction mixture from Entry 1, Table S6.


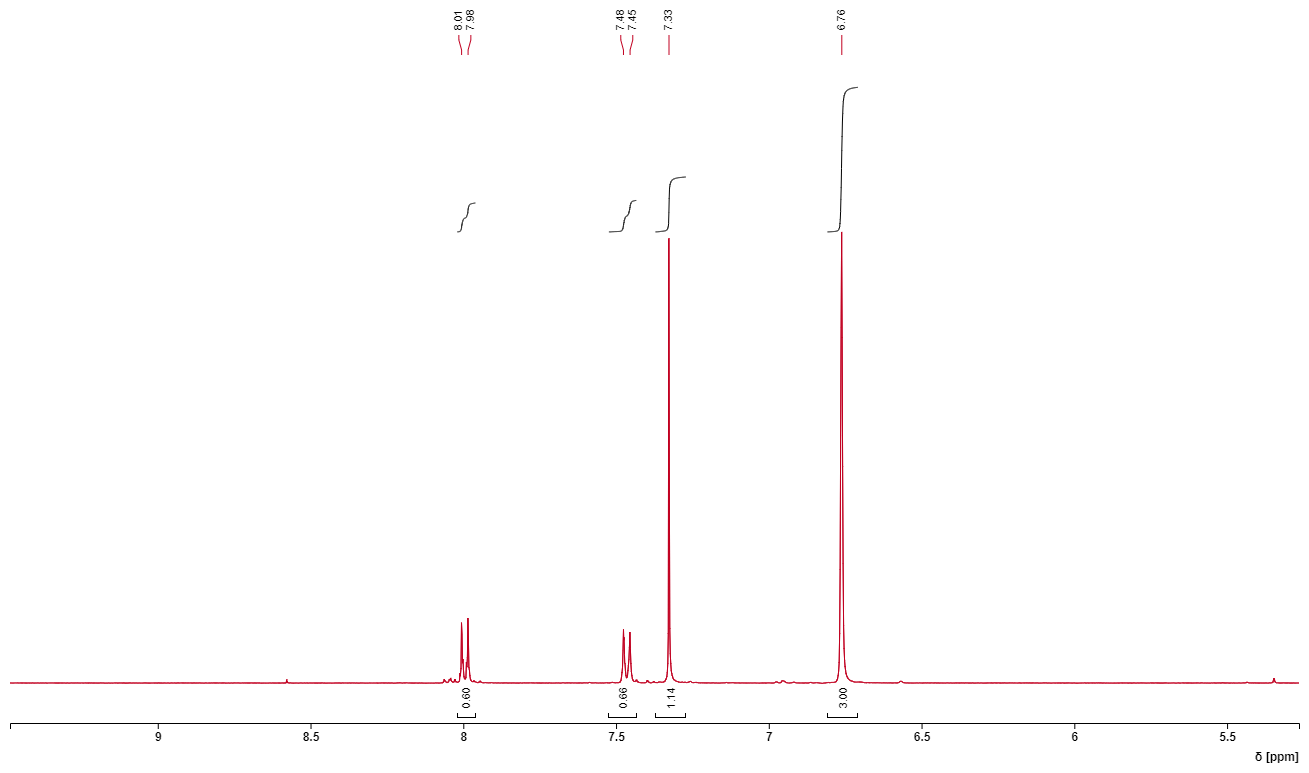


Figure S49. Fragment of ^1^H NMR (400 MHz, methanol-d4) spectrum of reaction mixture from Entry 2, Table S6.


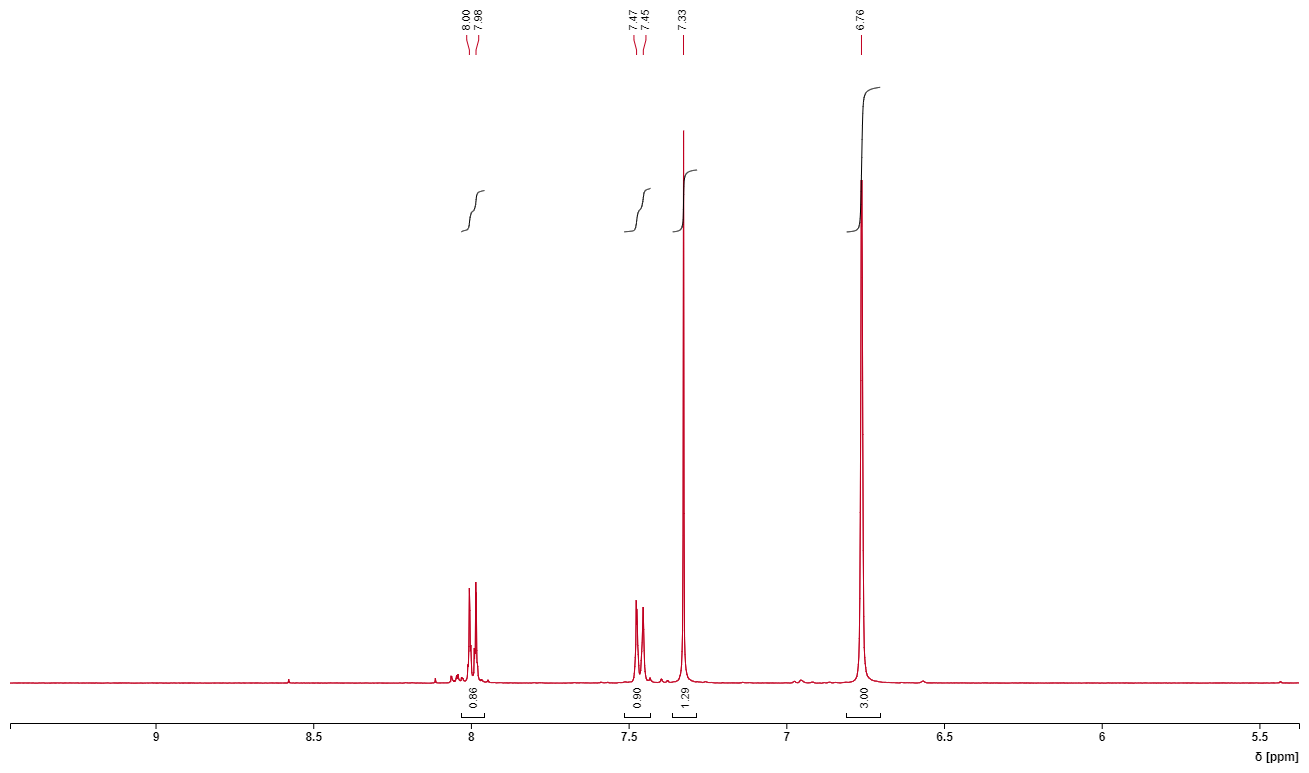


Figure S50. Fragment of ^1^H NMR (400 MHz, methanol-d4) spectrum of reaction mixture from Entry 3, Table S6.


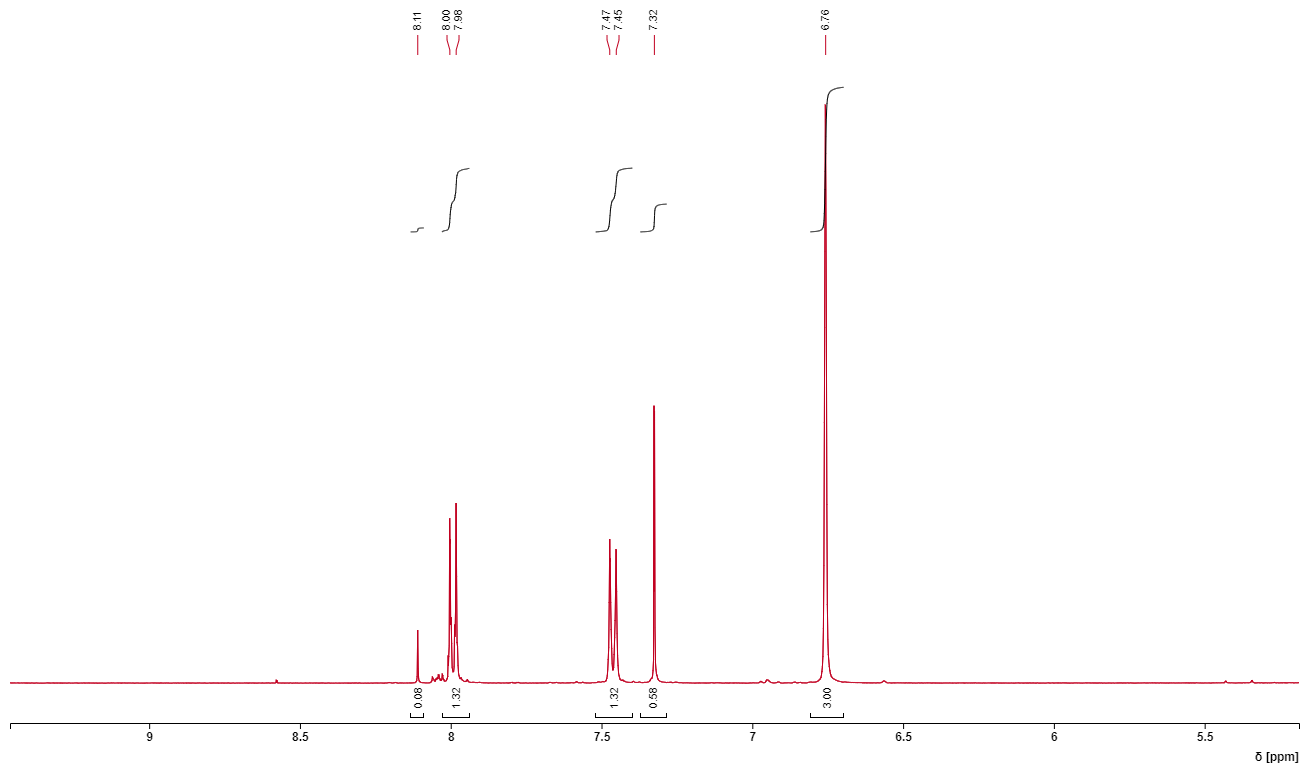


Figure S51. Fragment of ^1^H NMR (400 MHz, methanol-d4) spectrum of reaction mixture from Entry 4, Table S6.


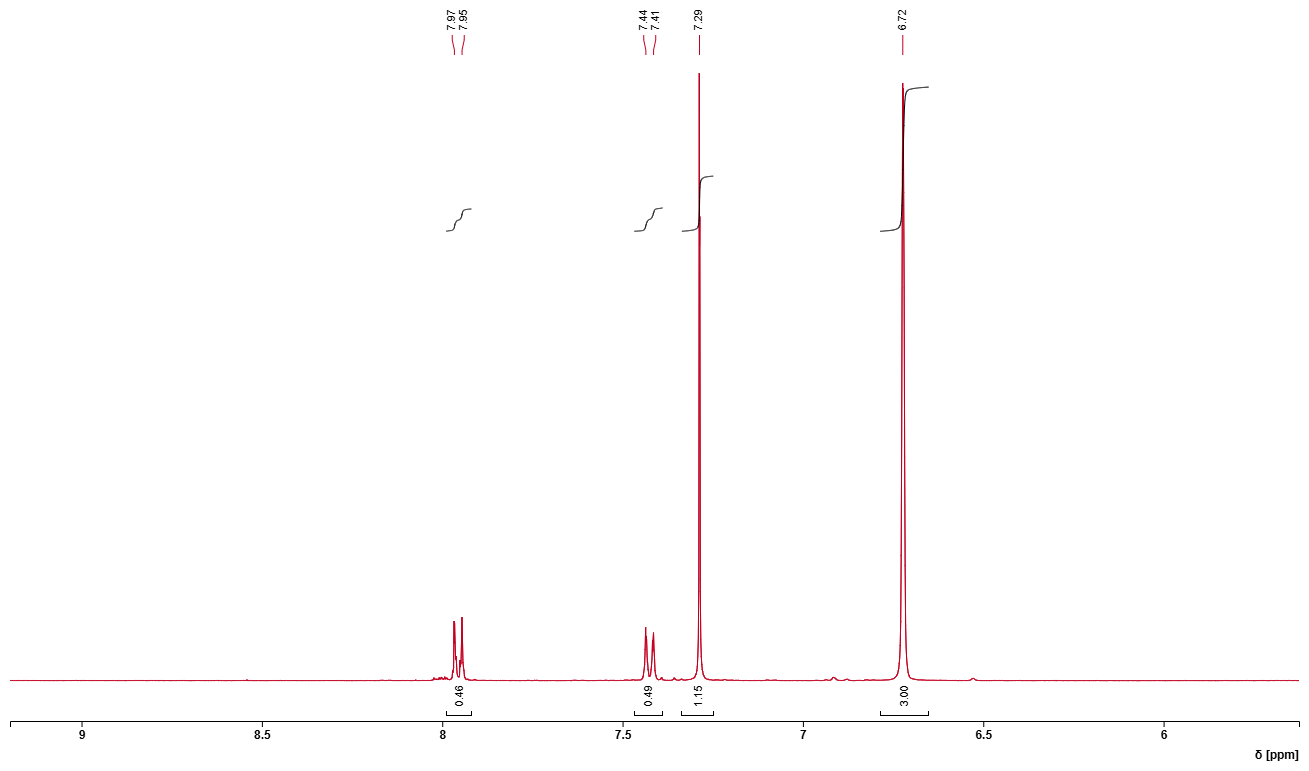


Figure S52. Fragment of ^1^H NMR (400 MHz, methanol-d4) spectrum of reaction mixture from Entry 5, Table S6.


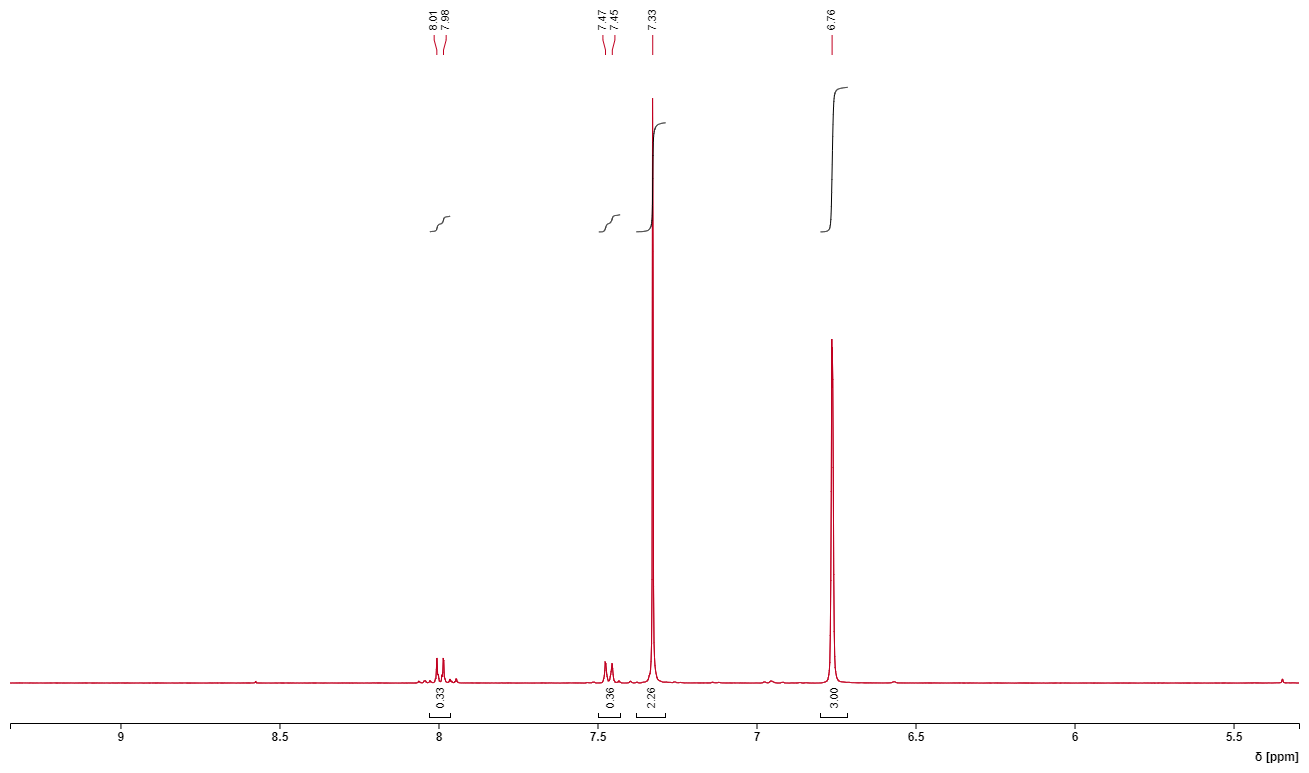


Figure S53. Fragment of ^1^H NMR (400 MHz, methanol-d4) spectrum of reaction mixture from Entry 6, Table S6.


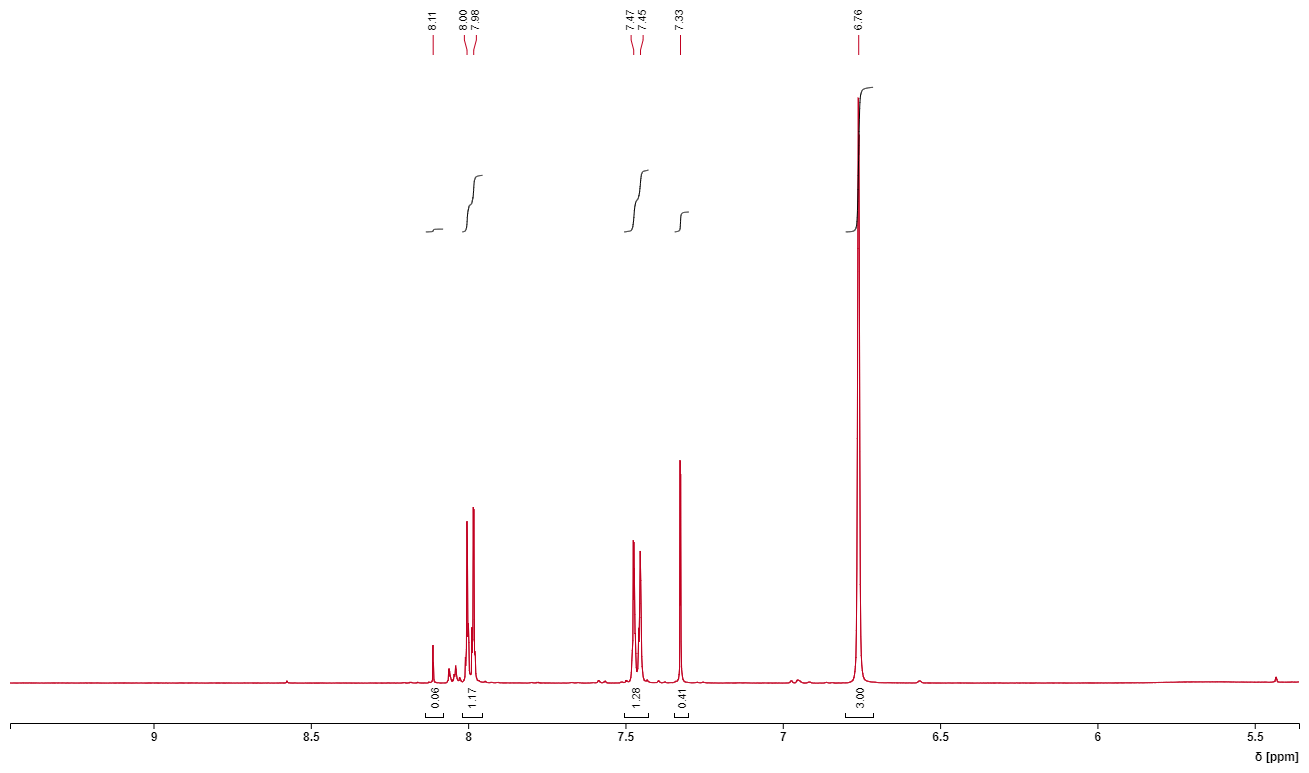


Figure S54. Fragment of ^1^H NMR (400 MHz, methanol-d4) spectrum of reaction mixture from Entry 7, Table S6.

### 2.3.5 Stage V.

Having in hand reliable conditions for hydrogenation reaction with 0.05 mol% of the catalyst **1**, we decided to screen other catalysts available to us (Table S7). It was found that in all the cases the yield of BDM was higher when complex **1** was used as a catalyst. The only comparable activity was obtained with another Gusev’s catalyst **7** (Entry 2, Table S7). This approach also allowed us to gather some information about reproducibility of the method and confidence interval for reaction yield was calculated (95% probability, Entry 1, Table S7).

Later, it was decided to try running reactions with 0.01 mol% of precatalysts **1**, **4**, **5**, **7** that worked the best at 0.05 mol% catalytic loadings. The four catalysts were premixed with 500 eq. KO*t*Bu in 2-MeTHF/EtOH or 500 eq. KOEt in EtOH, and it turned out that premixing precatalysts with the former in 2-MeTHF/EtOH resulted in highly active catalysts and the yields almost matched experiments with 0.05 mol% catalytic loadings (Entries 8–15, Table S7). Nevertheless, in all sets of experiments it was confirmed that complex **1** was the most promising precatalyst for PET hydrogenation with high TON.

**General procedure G.**

Complex (0.01 mmol) was weighed under air and placed into an 8 mL vial and sealed using a septum. The vial was purged with argon and 2-MeTHF (3.5 mL) and EtOH (0.5 mL) were added via syringe. To the resulting solution, KO*t*Bu (1 M solution, 1 mL, 1 mmol) was added. The yellowish solution (0.002 M of the catalyst and 0.2 M of KO*t*Bu) was stirred at room temperature for 30 min. Polyethylene terephthalate (192.2 mg, 1 mmol, 1 eq.), was weighed under air, placed into an 8 mL glass vial with a stir bar (vial and stir bar should be weighted), and sealed. The vial was purged with argon and 2-MeTHF (4.25 mL) and EtOH (0.5 mL) were added via syringe. Next, 0.25 mL of the prepared earlier solution of the catalyst and base was added into the sealed 8-mL vial (containing PET) via syringe under inert gas. Two needles were added at the top of the vial, and then it was placed inside a 150 mL autoclave with some metal beads to ensure thermal conductivity. The autoclave was purged with argon, then sealed, purged with H_2_ (20 bar), pressurized with H_2_ at 50 bars, and placed in an oil bath preheated to 80 °C. The reaction was stirred at 80 °C for 18 h. After that, the autoclave was cooled down to room temperature in air and then in an ice bath and carefully vented to atmosphere. After the reaction mixture was diluted with 2 mL of MeOH and stirred for 10 min at room temperature, the vial was weighed. Then ≈200 µL of mesitylene was added to the vial with the reaction mixture, and the solution was stirred for 10 min at room temperature. Next, 200–300 µL of the reaction mixture was added to NMR tube followed by the addition of MeOH-d4 (0.3 mL). Then, the mixture was analysed with ^1^H NMR.

**General procedure H.**

In the Glovebox under argon KO*t*Bu (561 mg, 5 mmol) was placed into an 8 mL vial and sealed. Dry 2-MeTHF (4.0 mL) and EtOH (1.0 mL) were added via syringe. To the resulting yellowish solution, precatalyst (0.01 mmol) was added. The solution (≈0.002 M of the catalyst and ≈1.0 M of KO*t*Bu) was stirred at room temperature for 30 min in a glovebox. Polyethylene terephthalate (384.4 mg, 2 mmol, 1 eq.), was weighed in a glovebox, placed into an 8 mL glass vial with a stir bar and sealed. 2-MeTHF (4.4 mL) and EtOH (0.5 mL) were added via syringe. Next, 0.1 mL of the prepared earlier solution of the precatalyst and base (suspension in case of **7**) was added into the sealed 8-mL vial (containing PET) via syringe under inert gas. Two needles were placed on top of the via and then it was placed inside a 150 mL autoclave with some metal beads to ensure thermal conductivity. The autoclave was purged with argon, then sealed, purged with H_2_ (20 bar), pressurized with H_2_ at 50 bar, and placed in an oil bath preheated to 80 °C. The reaction was stirred at 80 °C for 18 h. After that, the autoclave was cooled down to room temperature in air and then in an ice bath and carefully vented to atmosphere. After the reaction mixture was diluted with 2 mL of MeOH and stirred for 10 min at room temperature, the vial was weighed. Then ≈200 µL of mesitylene was added to the vial with the reaction mixture, and the solution was stirred for 10 min at room temperature. Next, 200–300 µL of the reaction mixture was added to an NMR tube followed by the addition of MeOH-d4 (0.3 mL). Then, the mixture was analysed by ^1^H NMR spectroscopy.

**General procedure I.**

In Glovebox under Argon precatalyst (0.01 mmol) was placed into 8 mL MW vial and sealed. EtOH (3.0 mL) was added via syringe followed by solution of KOEt in EtOH (2 mL, 2.5 M. 5 mmol). The yellowish solution (0.002 M of the catalyst and 1 M of KOEt) was stirred at room temperature for 15 min. Polyethylene terephthalate (384.4 mg, 2 mmol, 1 eq.), was weighed under air, placed into a 8 mL glass MW vial with a stir bar (vial and stir bar should be weighted) and sealed. 2-MeTHF (4.4 mL) and EtOH (0.5 mL) were added via syringe. Next, 0.1 mL of prepared earlier solution of the catalyst and base was added. The MW vial with two needles on top were placed inside a 150 mL autoclave with some metal beans to ensure good thermal conductivity. The autoclave was purged with H_2_ (20 bar), pressurized with H_2_ at 50 bar, and placed in an oil bath preheated to 80 °C. The reaction was stirred at 80 °C for 18 h. After that, the autoclave was cooled down to room temperature in an ice bath and carefully vented to atmosphere. After the reaction mixture was diluted with 2 mL of MeOH and stirred for 10 min at room temperature, the vial was weighted. Then ≈200 µL of mesitylene were added to the vial with the reaction mixture and the solution was stirred for 10 min at room temperature. Next, 200–300 µL of the reaction mixture was added to NMR tube followed by the addition of MeOH-d4 (0.3 mL). Then, the mixture was analysed by ^1^H NMR spectroscopy.

| **Table S7. Hydrogenation of PET with various catalysts.**     | | | | | | | |
| --- | --- | --- | --- | --- | --- | --- | --- |
| Entry | Start mat. | solvent | base | precat | Yield of BDM, % | Yield of EHMB, % | Yield of DETP, % |
| 1^[a]^ | PET-p (1 mmol) | 4.5 mL 2-MeTHF/0.5 mL EtOH | KOtBu | 0.05% **1** | 51±19^[b]^ | 46±20^[b]^ | <1 |
| 2^[a]^ | PET-p (1 mmol) | 4.5 mL 2-MeTHF/0.5 mL EtOH | KOtBu | 0.05% **2** | <1 | 24 | 66 |
| 3^[a]^ | PET-p (1 mmol) | 4.5 mL 2-MeTHF/0.5 mL EtOH | KOtBu | 0.05% **3** | <1 | 26 | 66 |
| 4^[a]^ | PET-p (1 mmol) | 4.5 mL 2-MeTHF/0.5 mL EtOH | KOtBu | 0.05% **4** | 2 | 63 | 23 |
| 5^[a]^ | PET-p (1 mmol) | 4.5 mL 2-MeTHF/0.5 mL EtOH | KOtBu | 0.05% **5** | 2 | 64 | 25 |
| 6^[a]^ | PET-p (1 mmol) | 4.5 mL 2-MeTHF/0.5 mL EtOH | KOtBu | 0.05% **7** | <1 | <1 | 95 |
| 7^[a]^ | PET-p (1 mmol) | 4.5 mL 2-MeTHF/0.5 mL EtOH | KOtBu | 0.05% **6** | 14 | 76 | 1 |
| 8^[c]^ | PET-p (2 mmol) | 4.5 mL 2-MeTHF/0.5 mL EtOH | KOtBu | 0.01% **1** | 21 | 79 | <1 |
| 9^[c]^ | PET-p (2 mmol) | 4.5 mL 2-MeTHF/0.5 mL EtOH | KOtBu | 0.01% **4** | 5 | 80 | 15 |
| 10^[c]^ | PET-p (2 mmol) | 4.5 mL 2-MeTHF/0.5 mL EtOH | KOtBu | 0.01% **5** | 1 | 49 | 50 |
| 11^[c]^ | PET-p (2 mmol) | 4.5 mL 2-MeTHF/0.5 mL EtOH | KOtBu | 0.01% **6** | 9 | 88 | 3 |
| 12^[d]^ | PET-p (2 mmol) | 4.5 mL 2-MeTHF/0.5 mL EtOH | KOEt | 0.01% **1** | 1 | 56 | 39 |
| 13^[d]^ | PET-p (2 mmol) | 4.5 mL 2-MeTHF/0.5 mL EtOH | KOEt | 0.01% **4** | <1 | 25 | 73 |
| 14^[d]^ | PET-p (2 mmol) | 4.5 mL 2-MeTHF/0.5 mL EtOH | KOEt | 0.01% **5** | <1 | 40 | 56 |
| 15^[d]^ | PET-p (2 mmol) | 4.5 mL 2-MeTHF/0.5 mL EtOH | KOEt | 0.01% **6** | <1 | 2 | 98 |
| *[a]* General Procedure G. *[b]* Confidence interval of 95% for 4 experiments was calculated *[c]* General Procedure H. *[c]* General Procedure I. | | | | | | | |


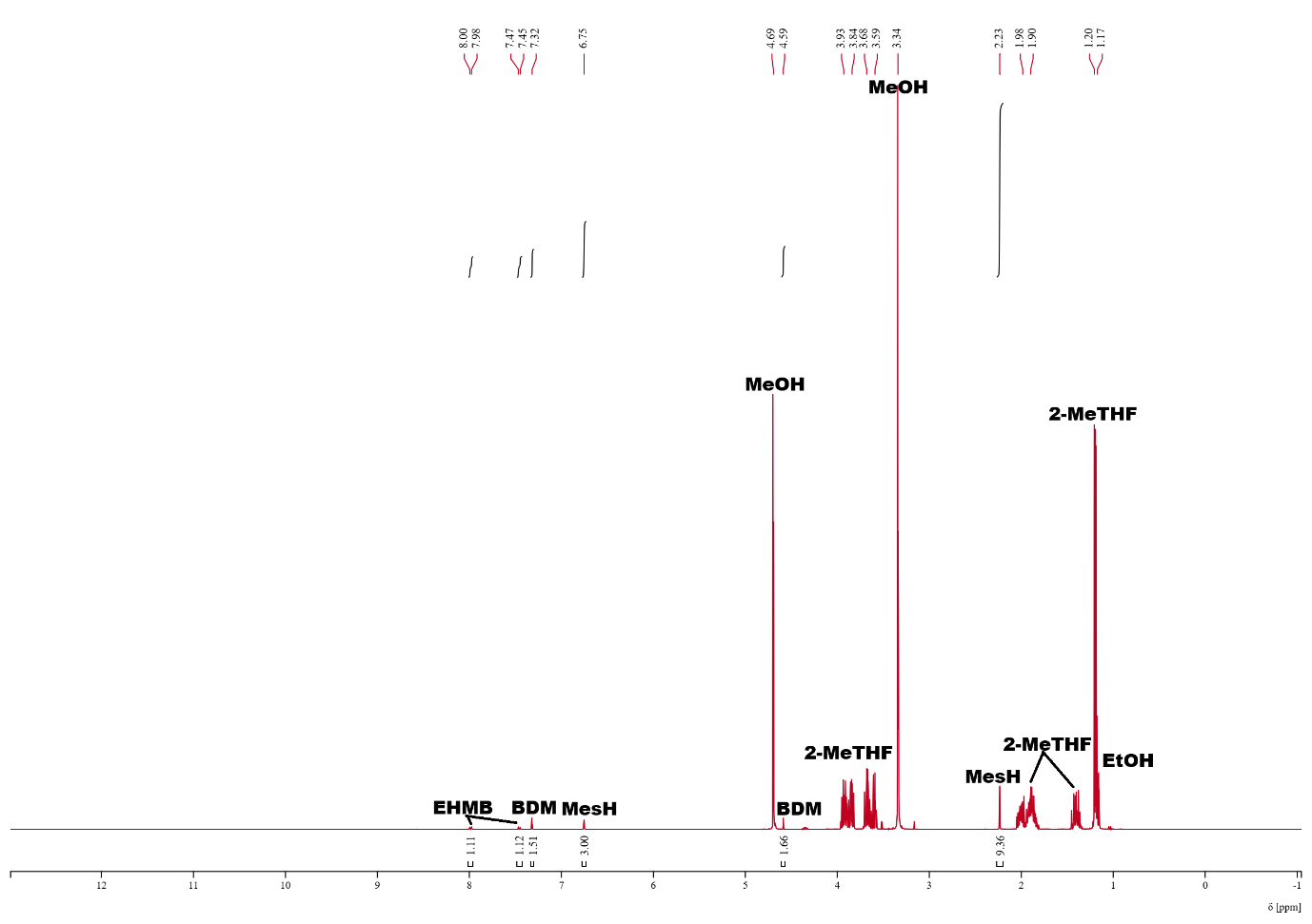


Figure S55. ^1^H NMR (400 MHz, methanol-d4) spectrum of reaction mixture from Entry 2, Table S7.


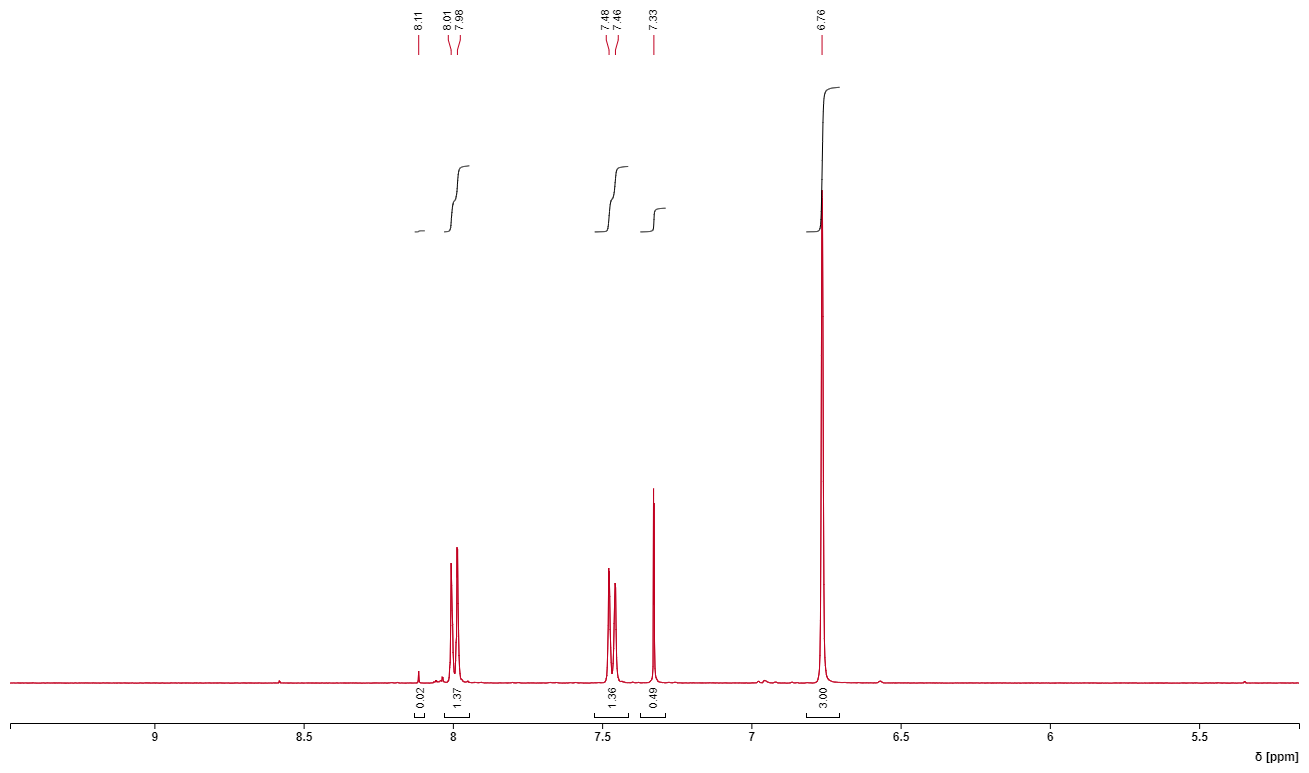


Figure S56. Fragment of ^1^H NMR (400 MHz, methanol-d4) spectrum of reaction mixture from Entry 2, Table S7.


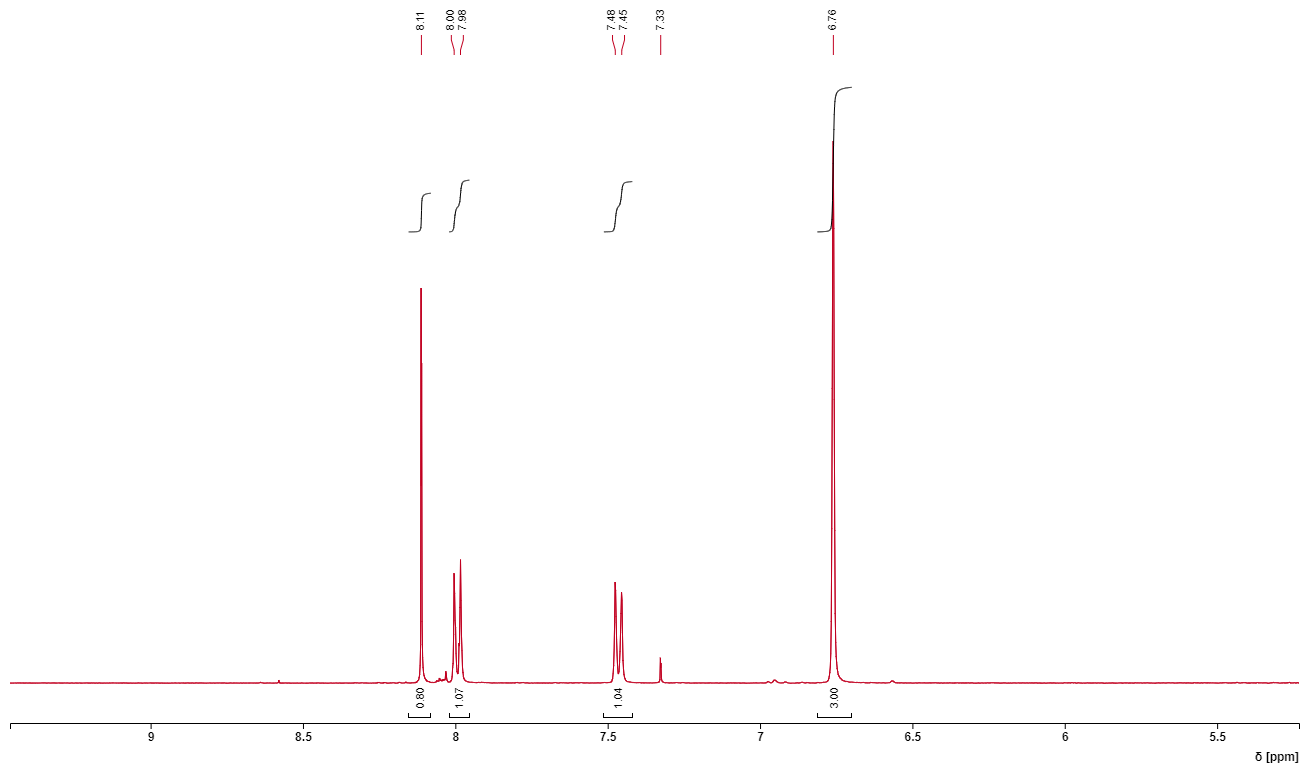


Figure S57. Fragment of ^1^H NMR (400 MHz, methanol-d4) spectrum of reaction mixture from Entry 3, Table S7.


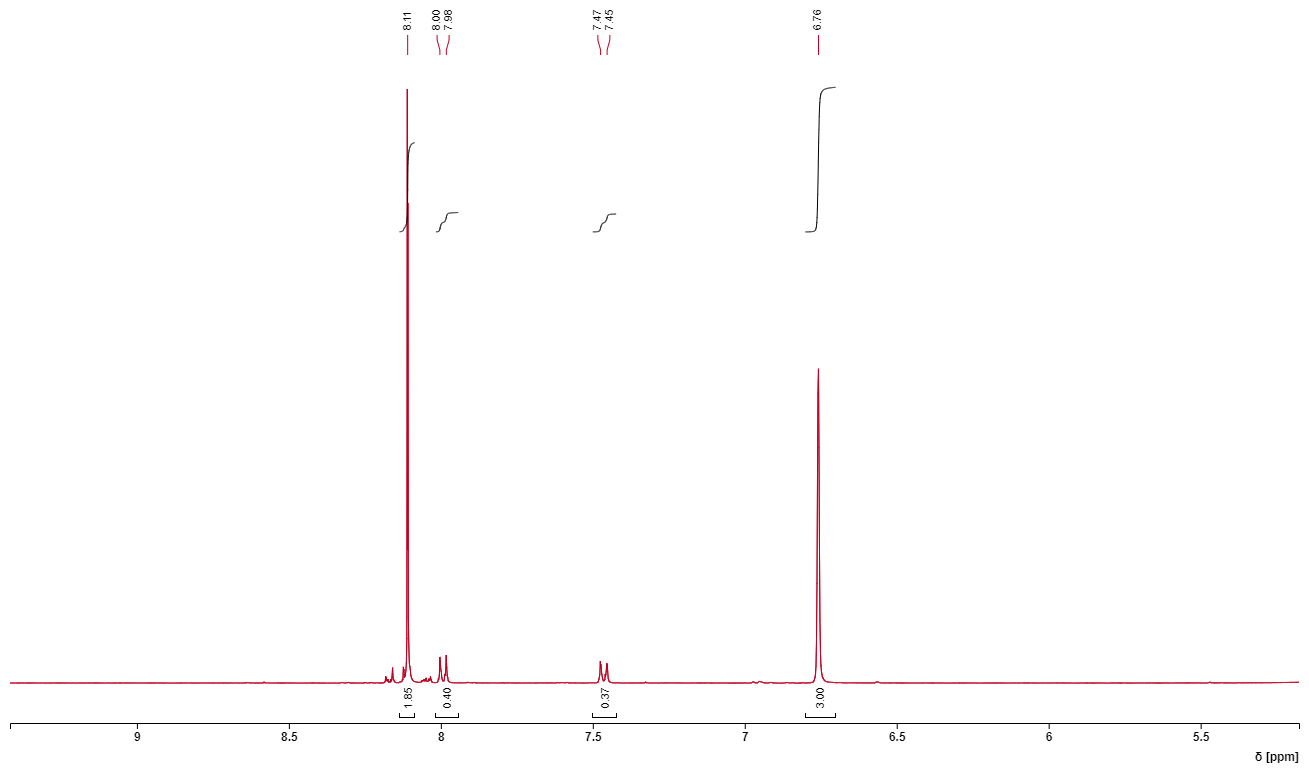


Figure S58. Fragment of ^1^H NMR (400 MHz, methanol-d4) spectrum of reaction mixture from Entry 4, Table S7.


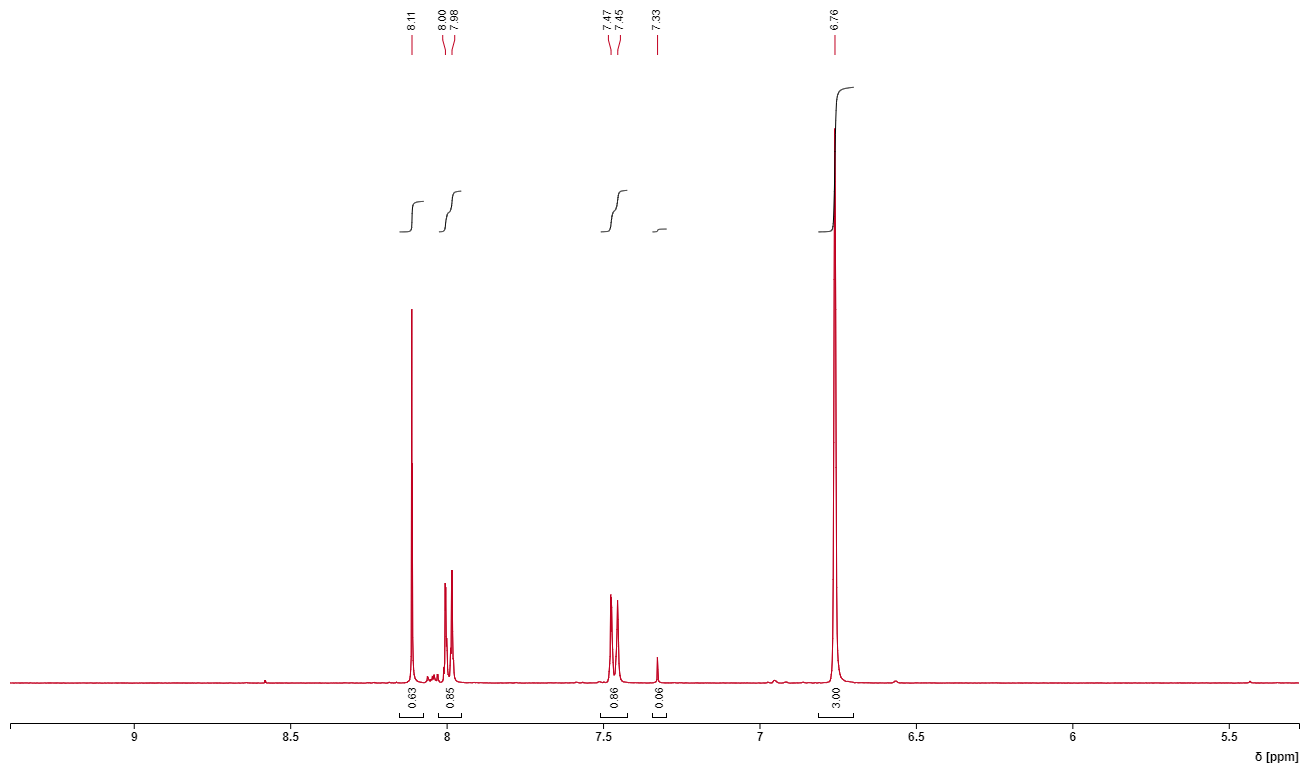


Figure S59. Fragment of ^1^H NMR (400 MHz, methanol-d4) spectrum of reaction mixture from Entry 5, Table S7.


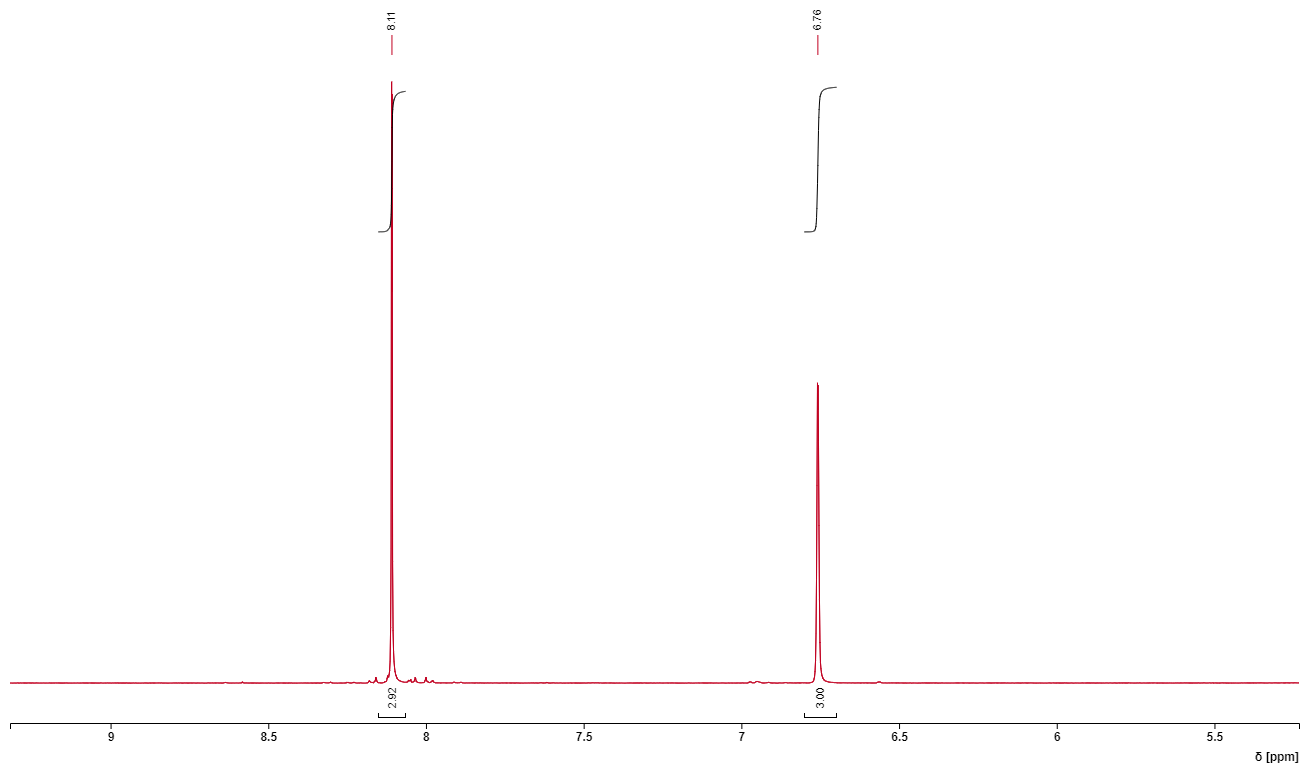


Figure S60. Fragment of ^1^H NMR (400 MHz, methanol-d4) spectrum of reaction mixture from Entry 6, Table S7.


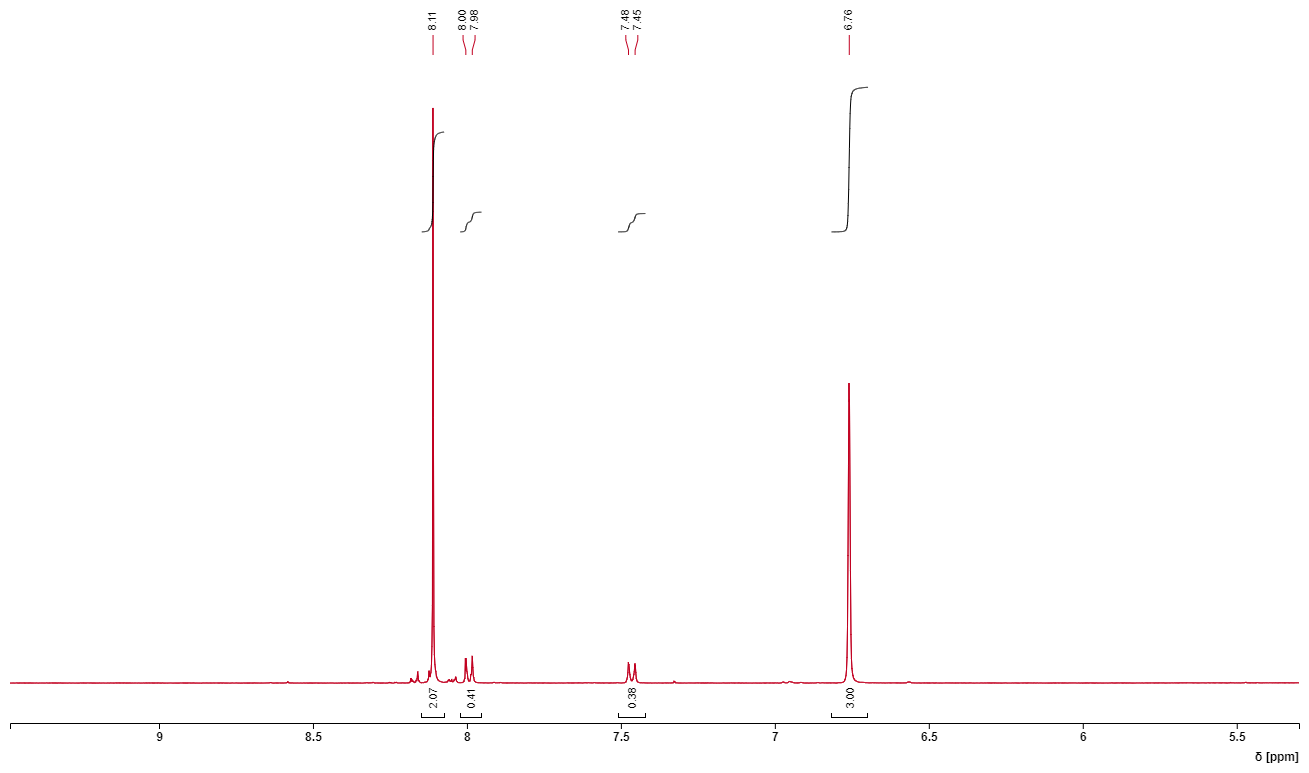


Figure S61. Fragment of ^1^H NMR (400 MHz, methanol-d4) spectrum of reaction mixture from Entry 7, Table S7.


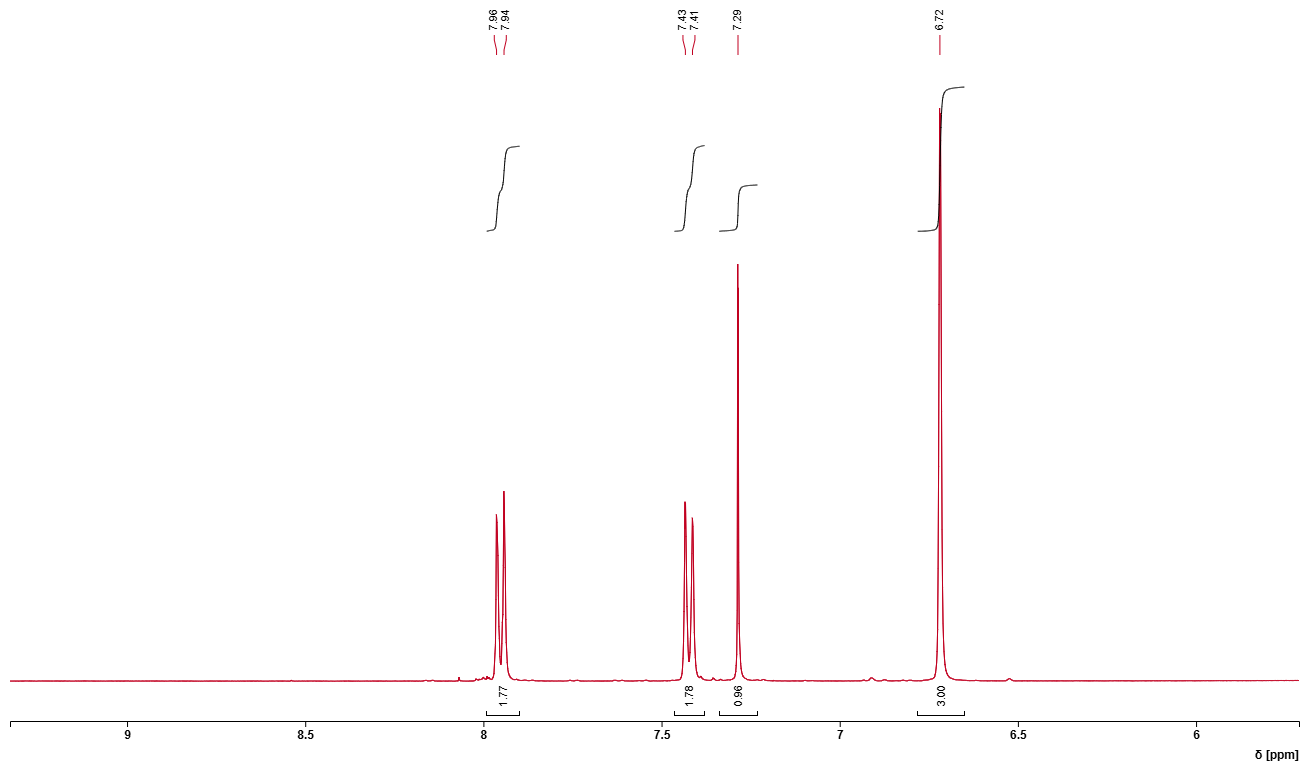


Figure S62. Fragment of ^1^H NMR (400 MHz, methanol-d4) spectrum of reaction mixture from Entry 8, Table S7.


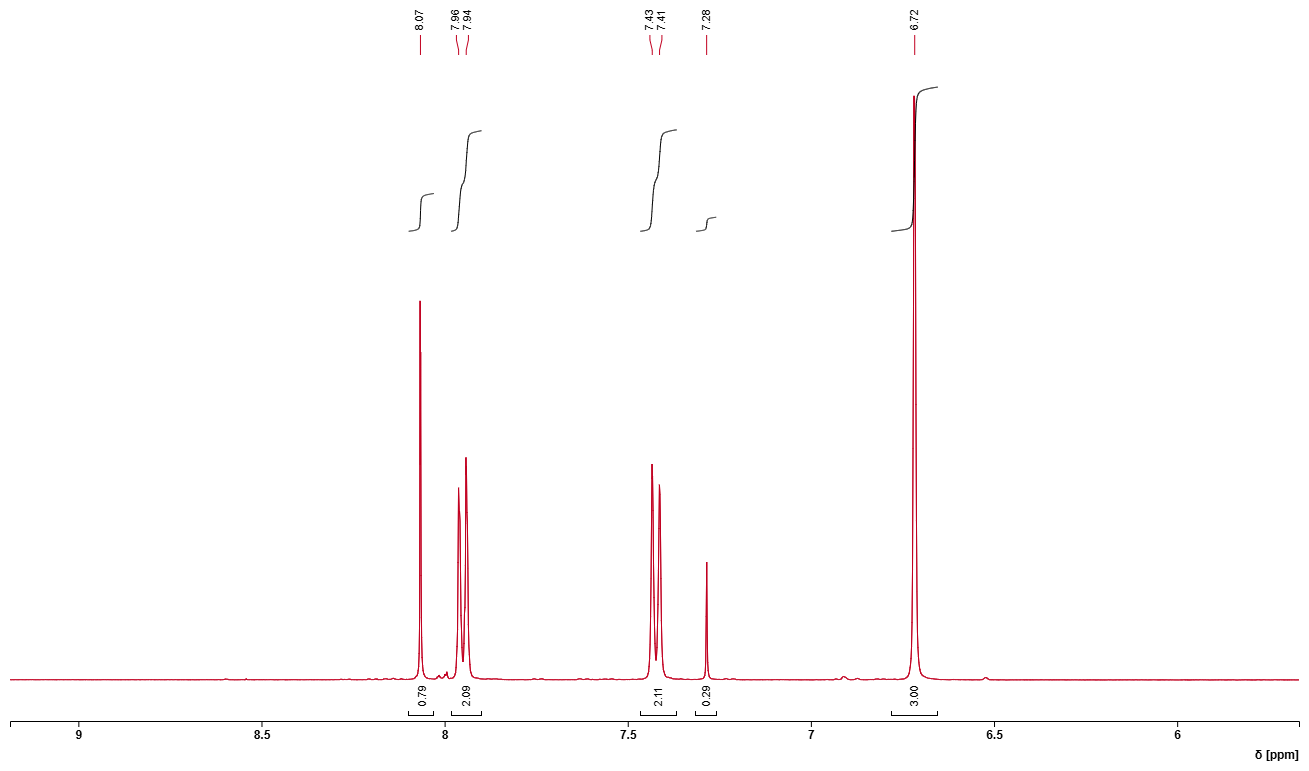


Figure S63. Fragment of ^1^H NMR (400 MHz, methanol-d4) spectrum of reaction mixture from Entry 9, Table S7.


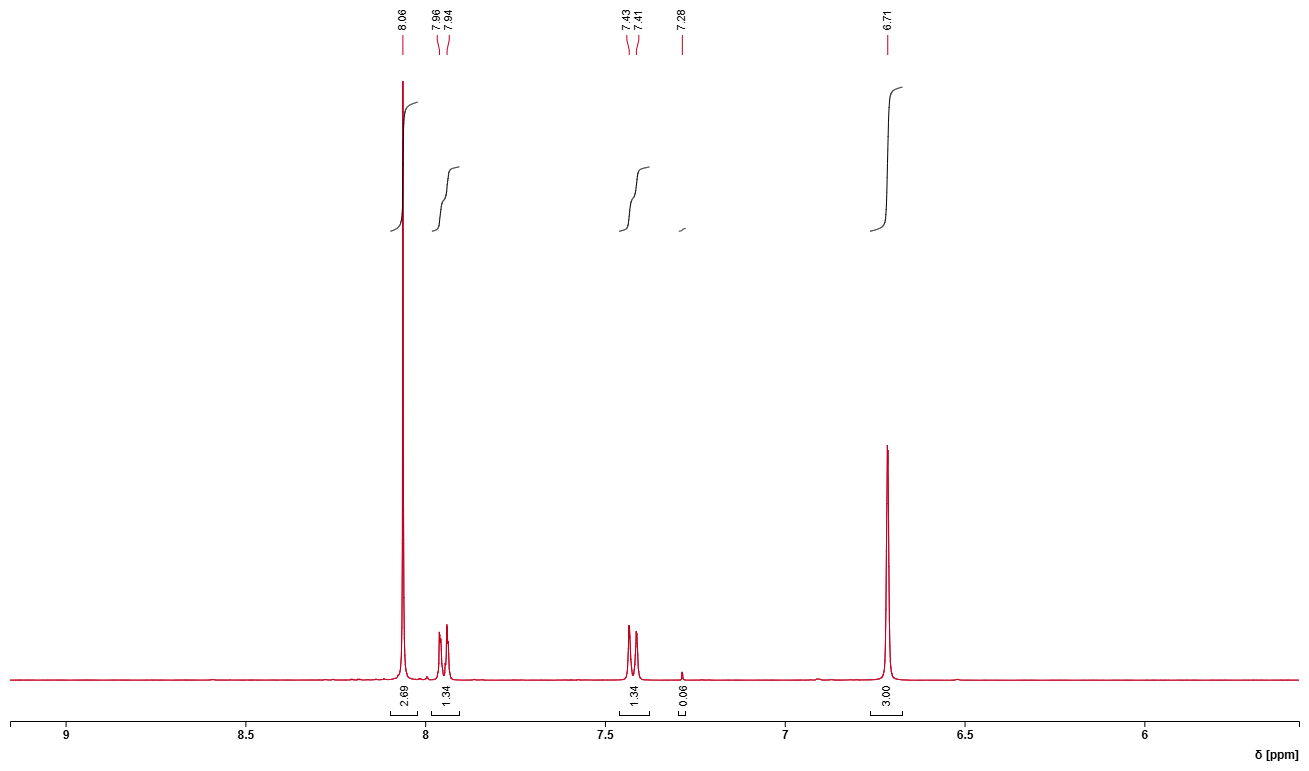


Figure S64. Fragment of ^1^H NMR (400 MHz, methanol-d4) spectrum of reaction mixture from Entry 10, Table S7.


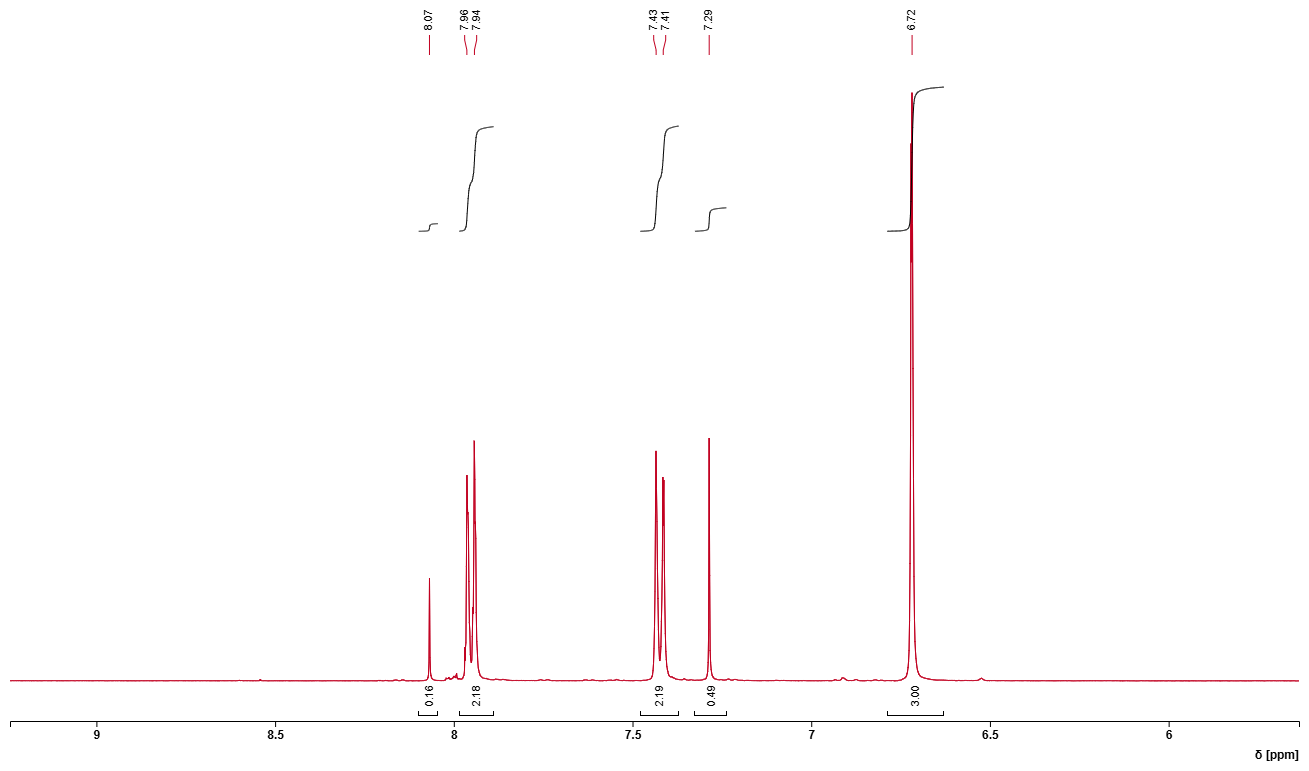


Figure S65. Fragment of ^1^H NMR (400 MHz, methanol-d4) spectrum of reaction mixture from Entry 11, Table S7.


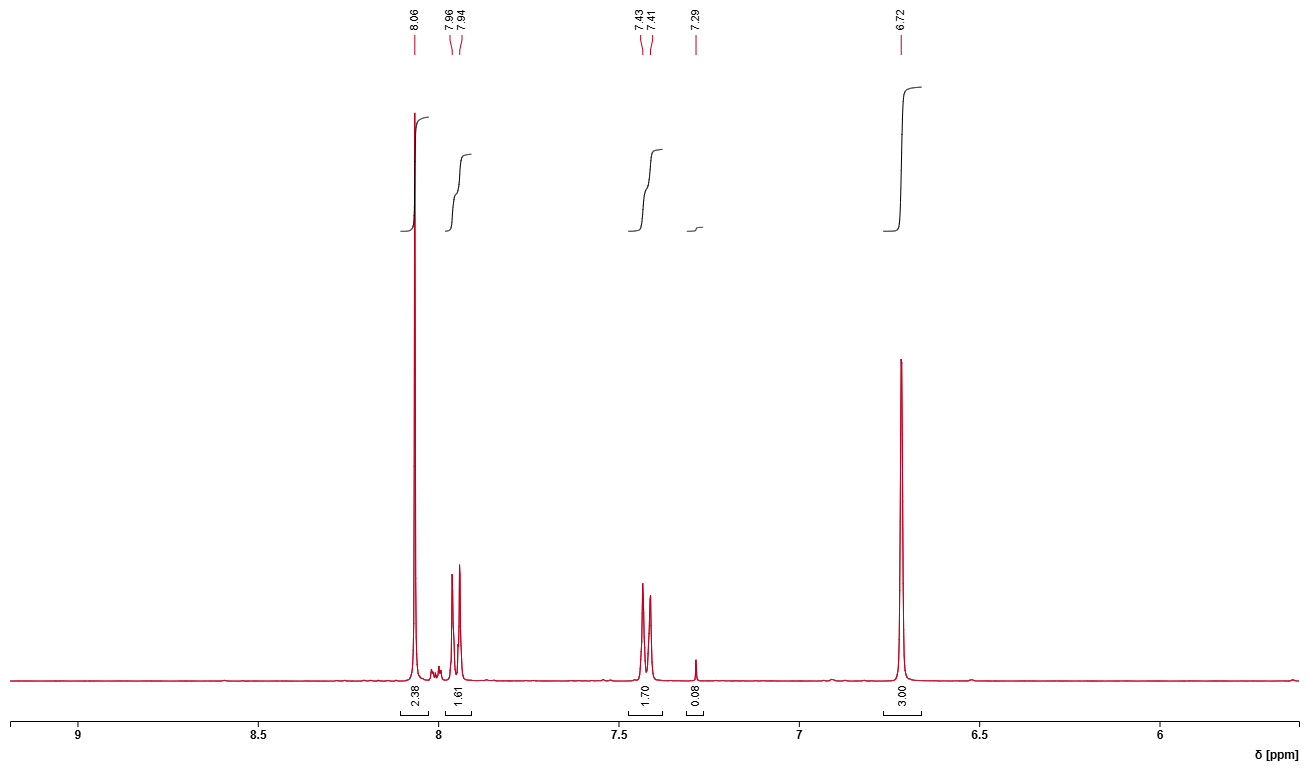


Figure S66. Fragment of ^1^H NMR (400 MHz, methanol-d4) spectrum of reaction mixture from Entry 12, Table S7.


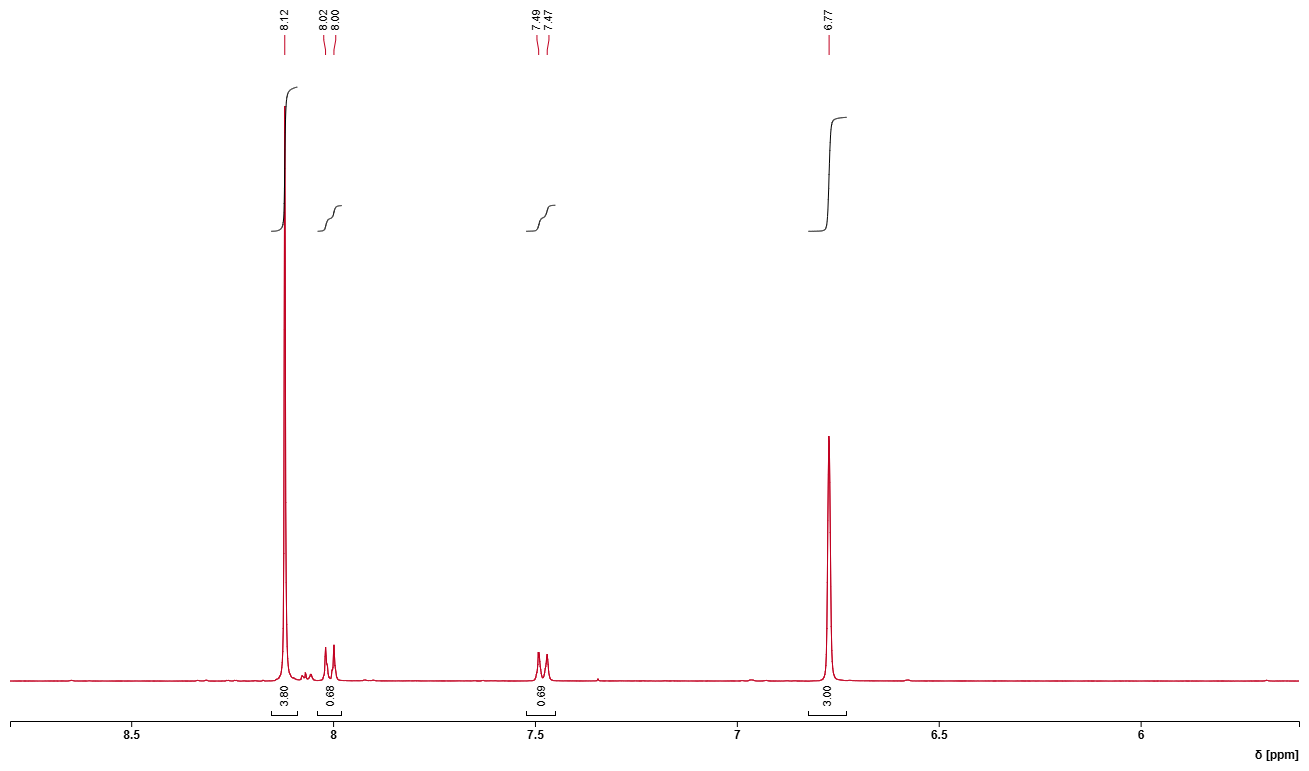


Figure S67. Fragment of ^1^H NMR (400 MHz, methanol-d4) spectrum of reaction mixture from Entry 13, Table S7.


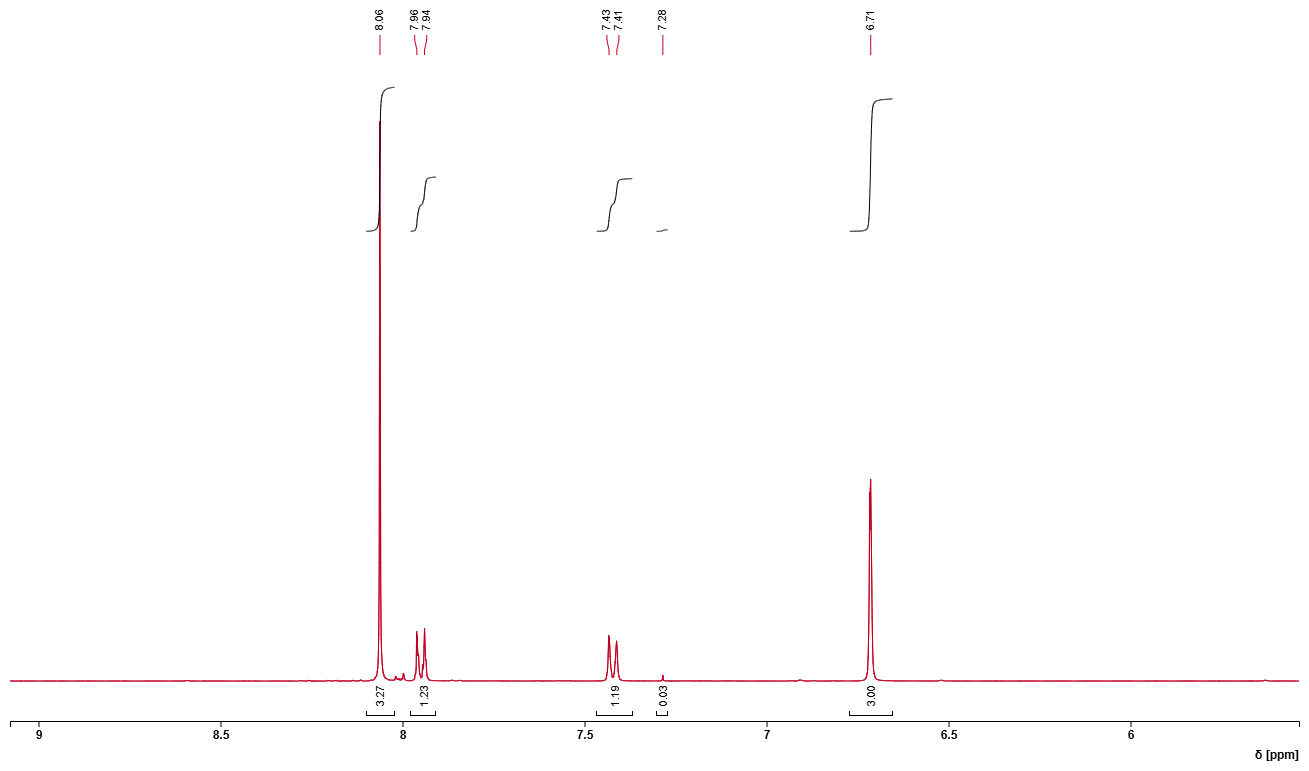


Figure S68. Fragment of ^1^H NMR (400 MHz, methanol-d4) spectrum of reaction mixture from Entry 14, Table S7.


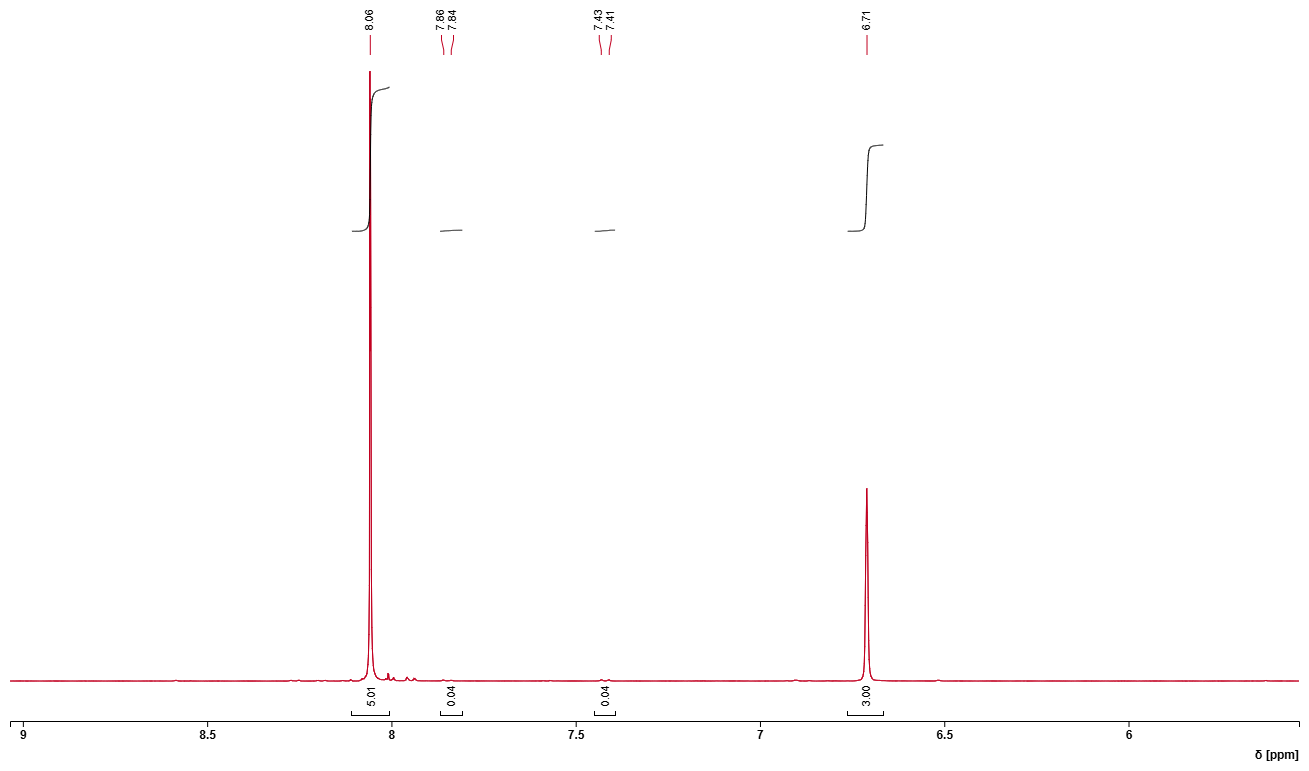


Figure S69. Fragment of ^1^H NMR (400 MHz, methanol-d4) spectrum of reaction mixture from Entry 15, Table S7.

### 2.3.6 Stage VI.

For the next series of experiments, 2 mmol of the polymer were hydrogenated in the same amount of solvent and same catalytic loadings (it means that concentrations of the catalyst and base were also increased twice). The increase of substrate, base and catalyst concentration led to improved hydrogenation yields (Table S8). Besides, post-consumer PET-b gave higher hydrogenation yields (Entries 4 and 5, Table S8). Lower catalytic loadings led to higher selectivity towards half-hydrogenated product EHMB (Entry 6, Table S8). As a result, the best yields of monoester EHMB were achieved when 0.01% of complex **1** were used in combination with 5% of KO*t*Bu (Entries 7 and 8, Table S8).

**General procedure J.**

Complex **1** (0.01 mmol) was weighted under air and placed into 8 mL MW vial and sealed. The vial was backfilled with argon and absolute 2-MeTHF (3.5 mL) and EtOH (0.5 mL) was added via syringe. To the resulting yellowish solution, KO*t*Bu (1M solution, 1 mL, 1 mmol) was added. The yellowish solution (0.002 M of the catalyst and 0.2 M of KO*t*Bu) was stirred at room temperature for 30 min. Polyethylene terephthalate (384.4 mg, 2 mmol, 1 eq.), was weighed under air, placed into a 8 mL glass MW vial with a stir bar (vial and stir bar should be weighted) and sealed. The vial was backfilled with argon and 2-MeTHF (4.0–4.25 mL) and absolute EtOH (0.5 mL) were added via syringe. Next, if needed 18-crown-6 was added and 0.25–0.5 mL of prepared earlier solution of the catalyst and base was added into sealed MW 8-mL vial via syringe under inert gas. The MW vial with two needles on top were placed inside a 150 mL autoclave with some metal beans to ensure thermal conductivity. The autoclave was purged with argon, then sealed, purged with H_2_ (20 bar), pressurized with H_2_ at 50 bar, and placed in an oil bath preheated to 80 °C. The reaction was stirred at 80 °C or 70 °C for 18–67 h. After that, the autoclave was cooled down to room temperature in an ice bath and carefully vented to atmosphere. After the reaction mixture was diluted with 2 mL of MeOH and stirred for 10 min at room temperature, the vial was weighted. Then ≈200 µL of mesitylene were added to the vial with the reaction mixture and the solution was stirred for 10 min at room temperature. Next, 200–300 µL of the reaction mixture was added to NMR tube followed by the addition of MeOH-d4 (0.3 mL). Then, the mixture was analysed with ^1^H NMR.

| **Table S8. Hydrogenation of PET with 1 at higher concentrations of PET.***^[^****^a]^***   | | | | | | | | |
| --- | --- | --- | --- | --- | --- | --- | --- | --- |
| Entry | Start mat. | solvent | KO*t*Bu, mol% | Cat. **1**, mol% | Time, h | Yield of BDM, % | Yield of EHMB, % | Yield of DETP, % |
| 1 | PET-p (2 mmol) | 4.5 mL 2-MeTHF/0.5 mL EtOH | 5 | 0.05 | 18 | 72±12^[b]^ | 26±12^[b]^ | <1 |
| 2 | PET-b (2 mmol) | 4.5 mL 2-MeTHF/0.5 mL EtOH | 5 | 0.05 | 24 | 80 | 19 | <1 |
| 3 | PET-p (2 mmol) | 4.5 mL 2-MeTHF/0.5 mL EtOH | 5 | 0.05 | 67 | 88 | 11 | <1 |
| 4 | PET-b (2 mmol) | 4.5 mL 2-MeTHF/0.5 mL EtOH | 5 | 0.05 | 67 | 95 | 2 | <1 |
| 5^[c]^ | PET-p (2 mmol) | 4.5 mL 2-MeTHF/0.5 mL EtOH | 5 | 0.05 | 18 | 39 | 54 | <1 |
| 6^[d]^ | PET-p (2 mmol) | 4.5 mL 2-MeTHF/0.5 mL EtOH | 5 | 0.05 | 18 | 33 | 64 | <1 |
| 7 | PET-p (2 mmol) | 4.5 mL 2-MeTHF/0.5 mL EtOH | 2.5 | 0.025 | 24 | 22 | 74 | <1 |
| *[a]* General procedure J. *[b]* Confidence interval of 95% for 4 experiments was calculated *[c]* Reaction was run at 70 °C. *[d]* 5 mol% of 18-crown-6 was added to the reaction mixture. | | | | | | | | |


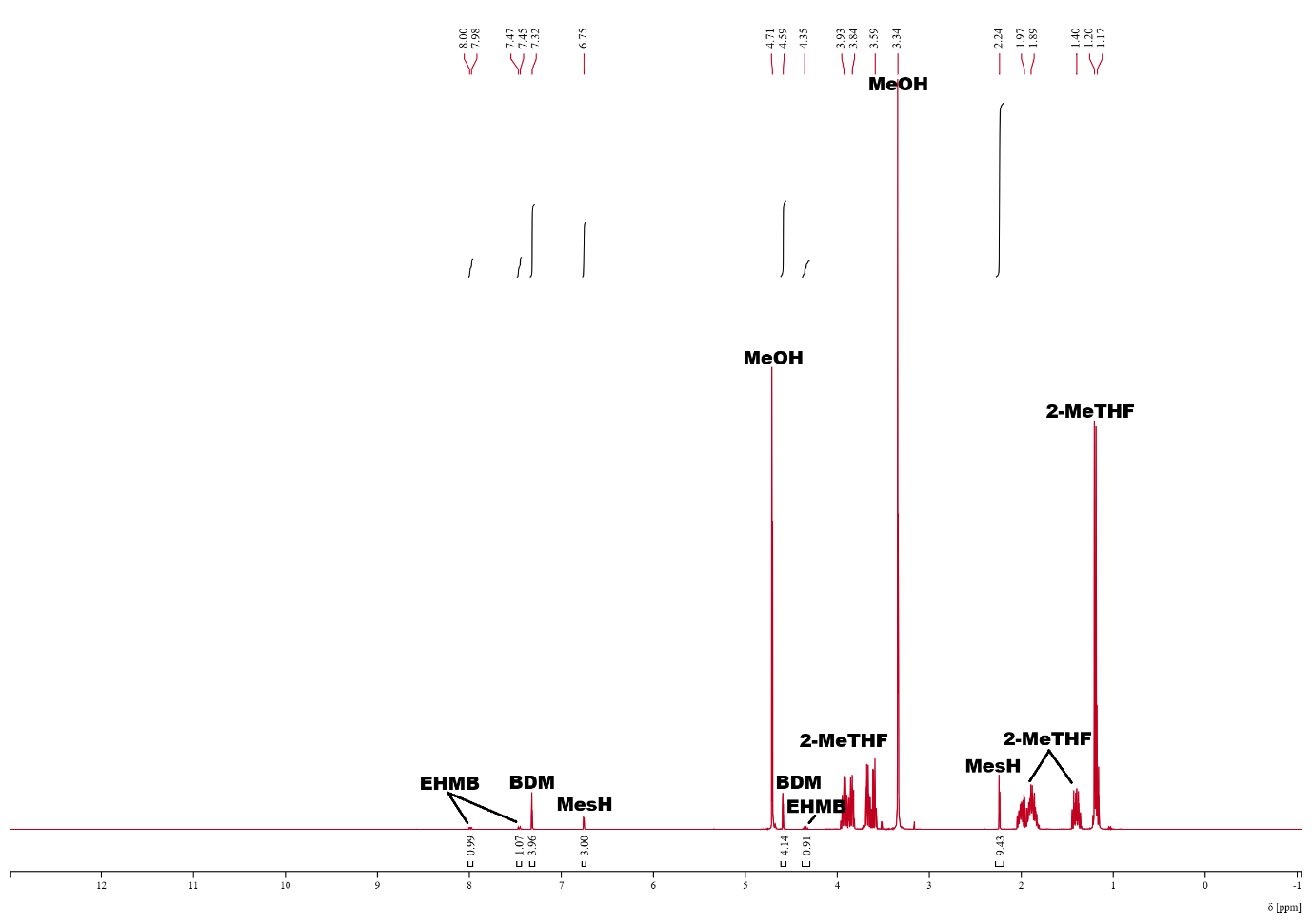


Figure S70. ^1^H NMR (400 MHz, methanol-d4) spectrum of reaction mixture from Entry 1, Table S8.


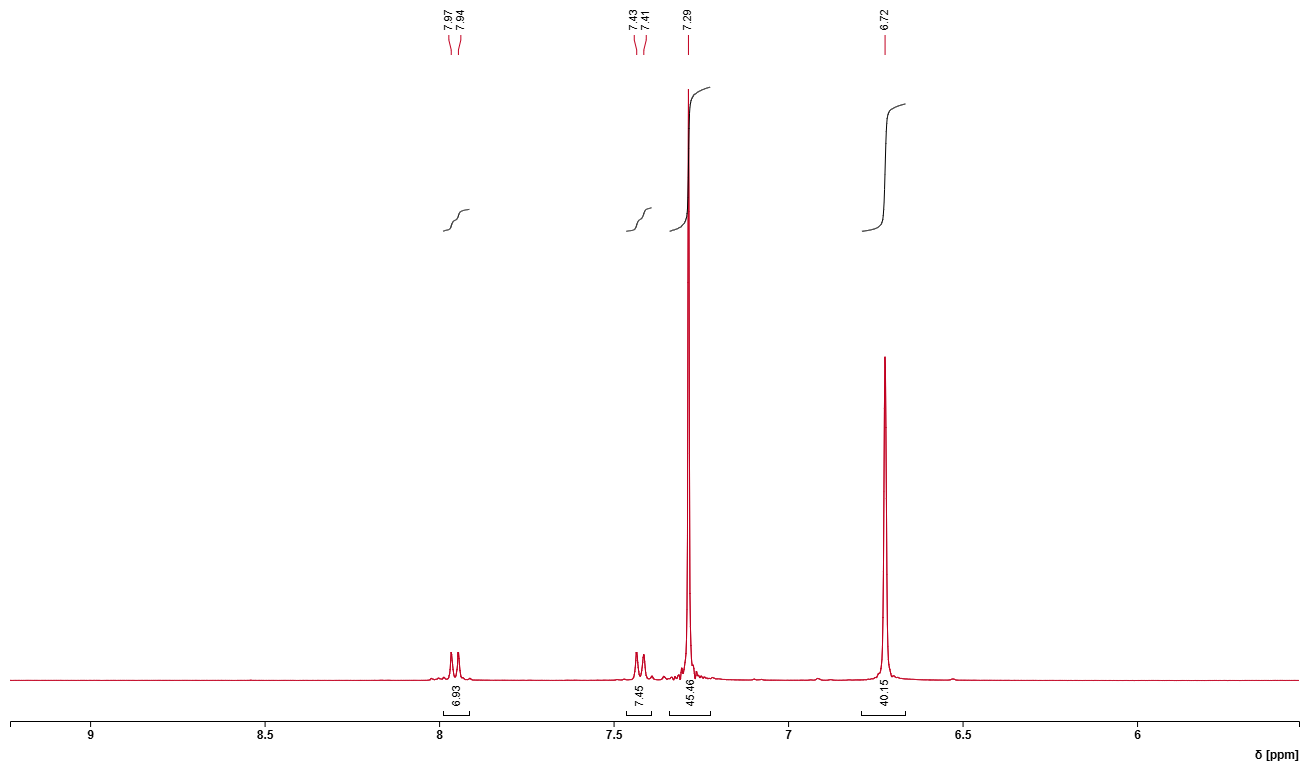


Figure S71. Fragment of ^1^H NMR (400 MHz, methanol-d4) spectrum of reaction mixture from Entry 1, Table S8.


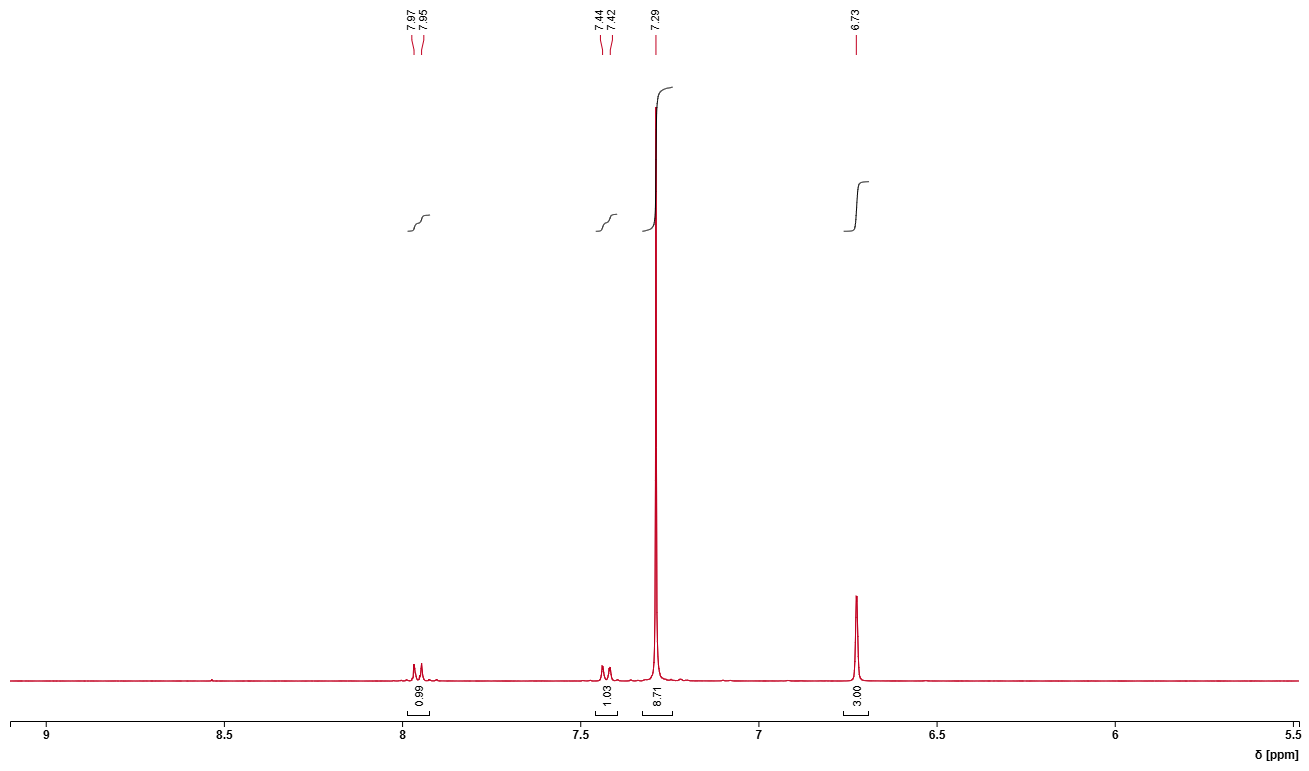


Figure S72. Fragment of ^1^H NMR (400 MHz, methanol-d4) spectrum of reaction mixture from Entry 2, Table S8.


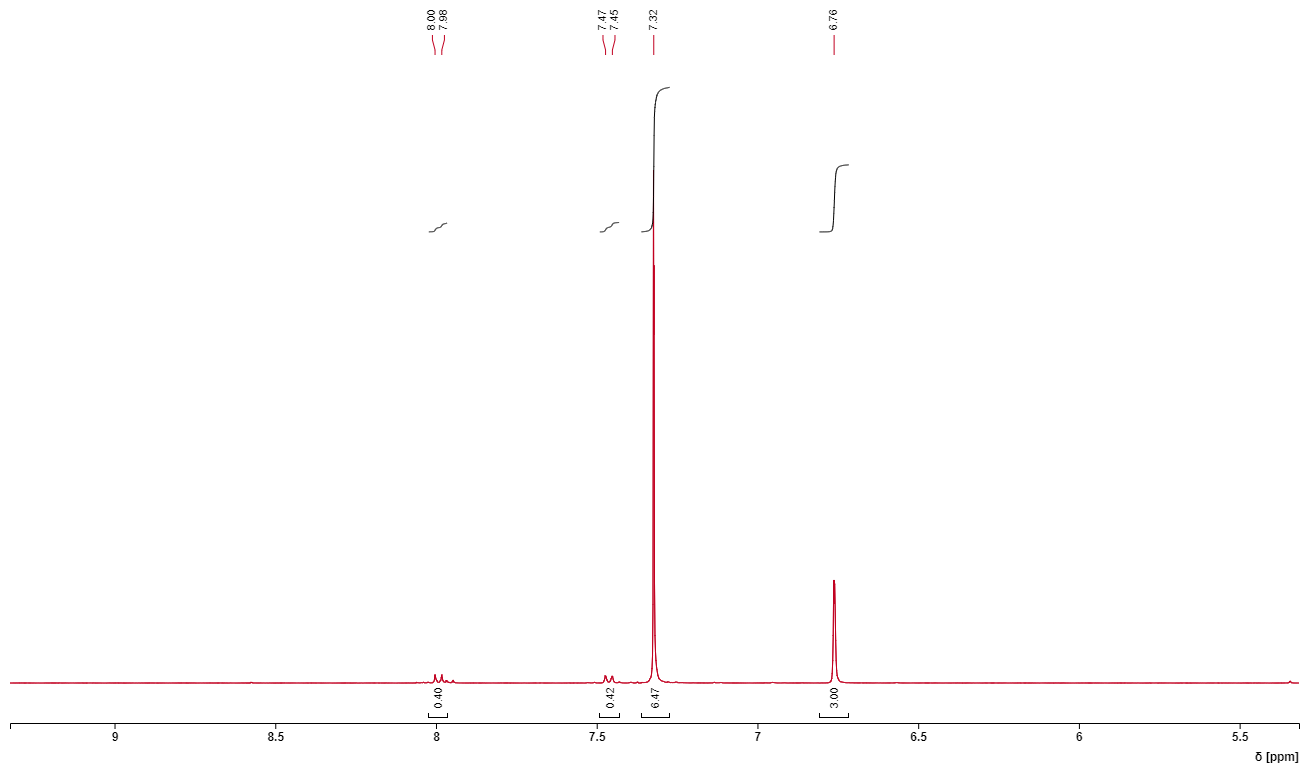


Figure S73. Fragment of ^1^H NMR (400 MHz, methanol-d4) spectrum of reaction mixture from Entry 3, Table S8.


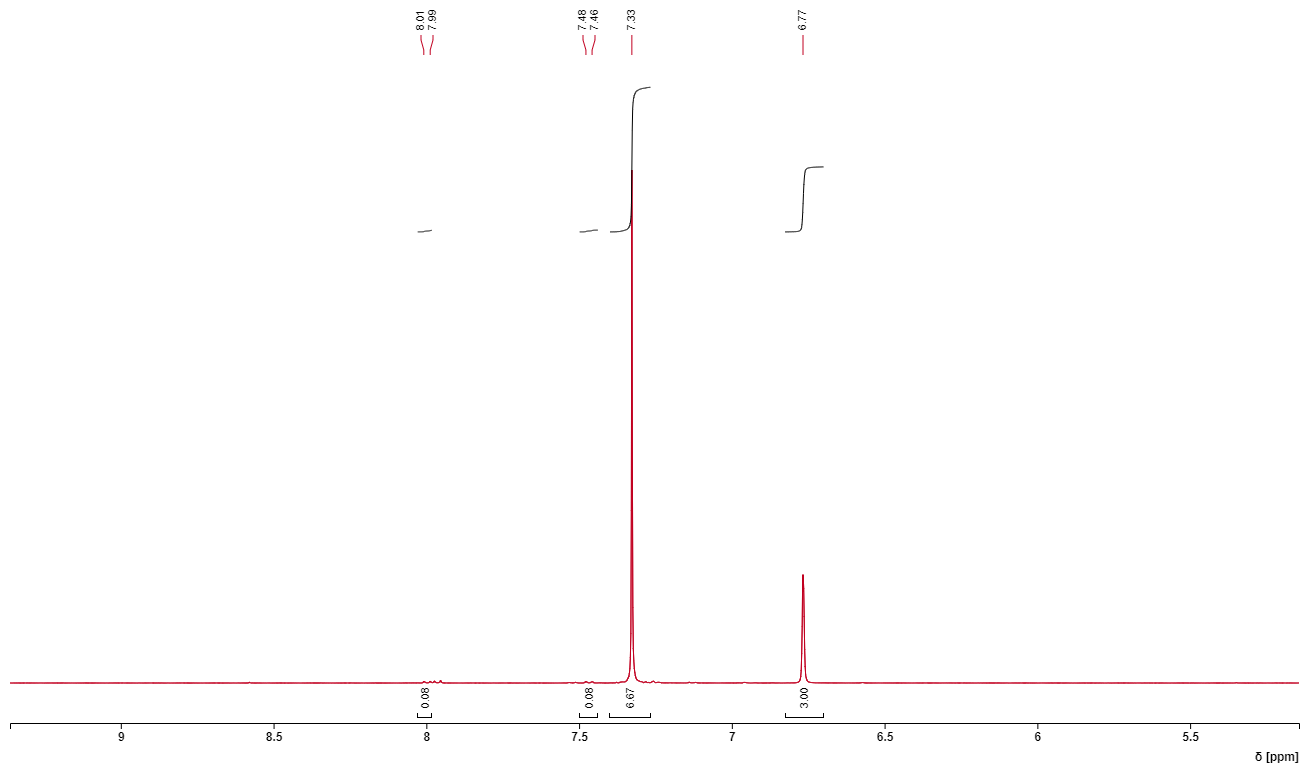


Figure S74. Fragment of ^1^H NMR (400 MHz, methanol-d4) spectrum of reaction mixture from Entry 4, Table S8.


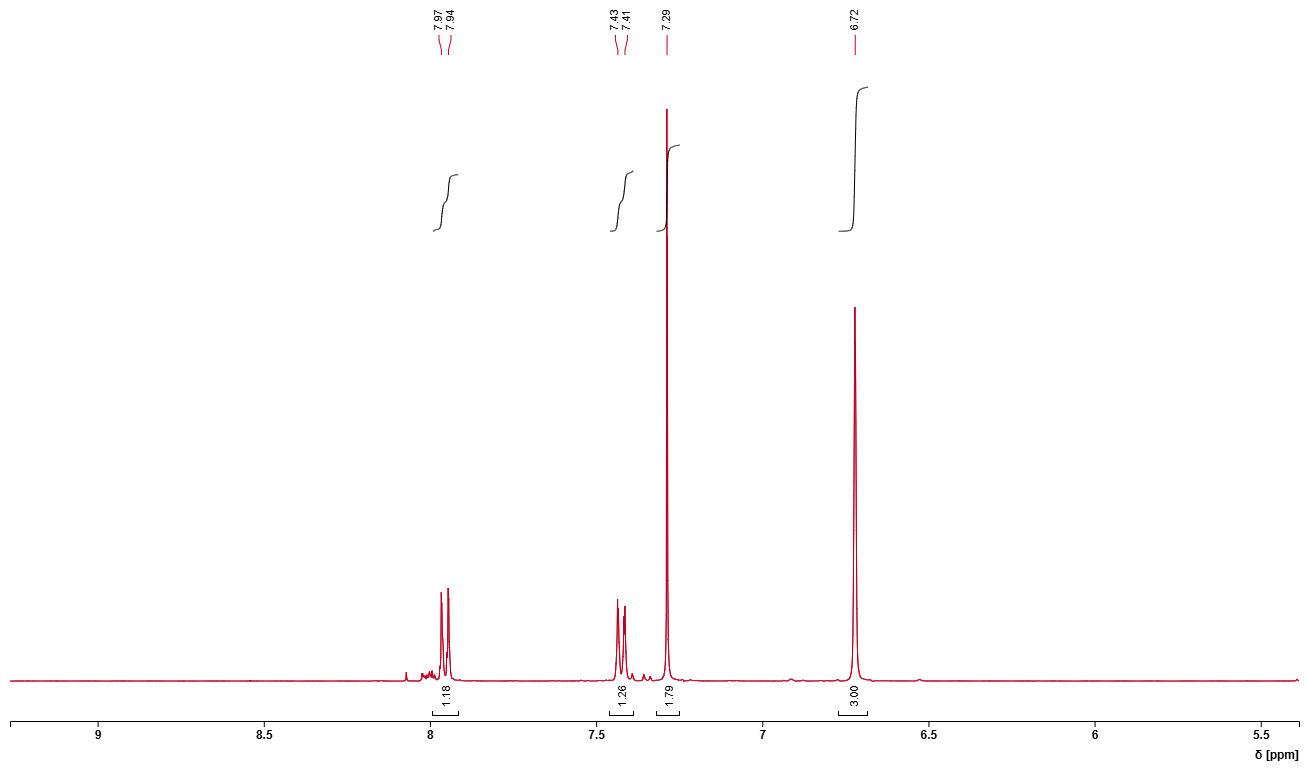


Figure S75. Fragment of ^1^H NMR (400 MHz, methanol-d4) spectrum of reaction mixture from Entry 5, Table S8.


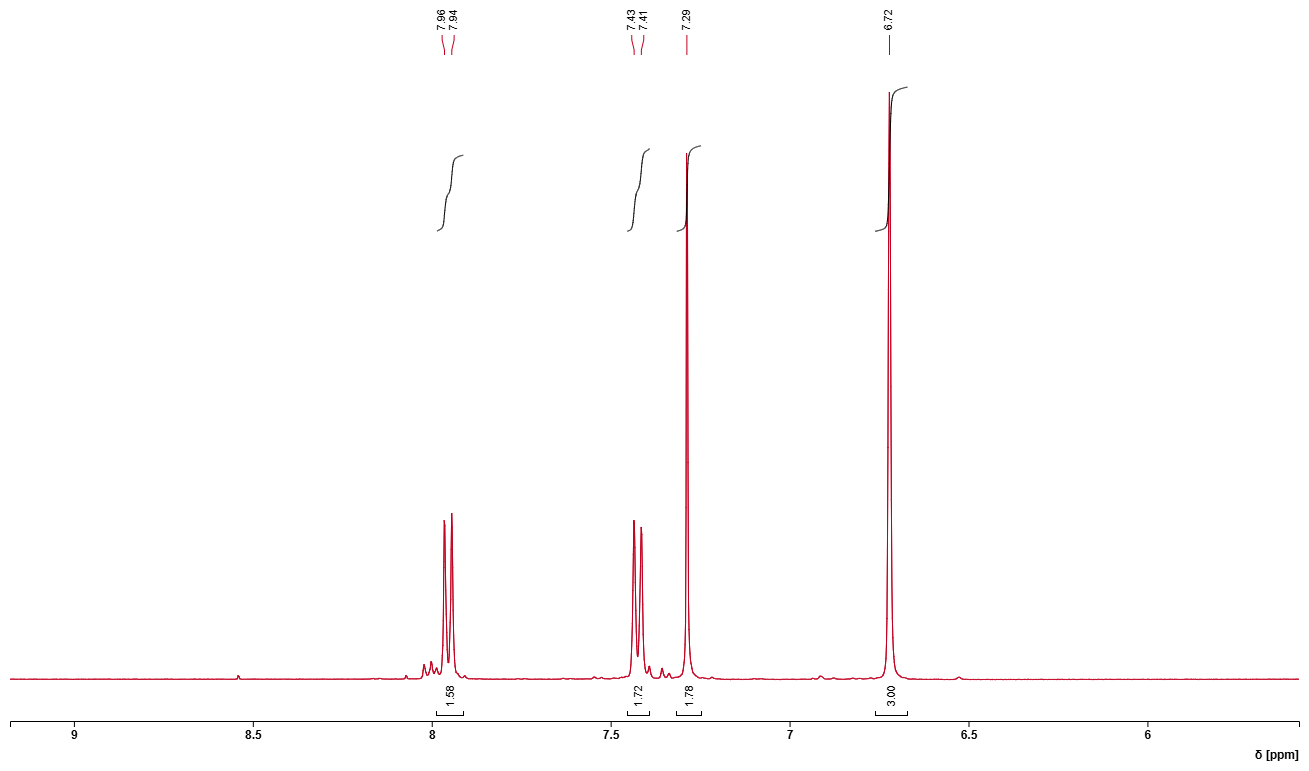


Figure S76. Fragment of ^1^H NMR (400 MHz, methanol-d4) spectrum of reaction mixture from Entry 6, Table S8.


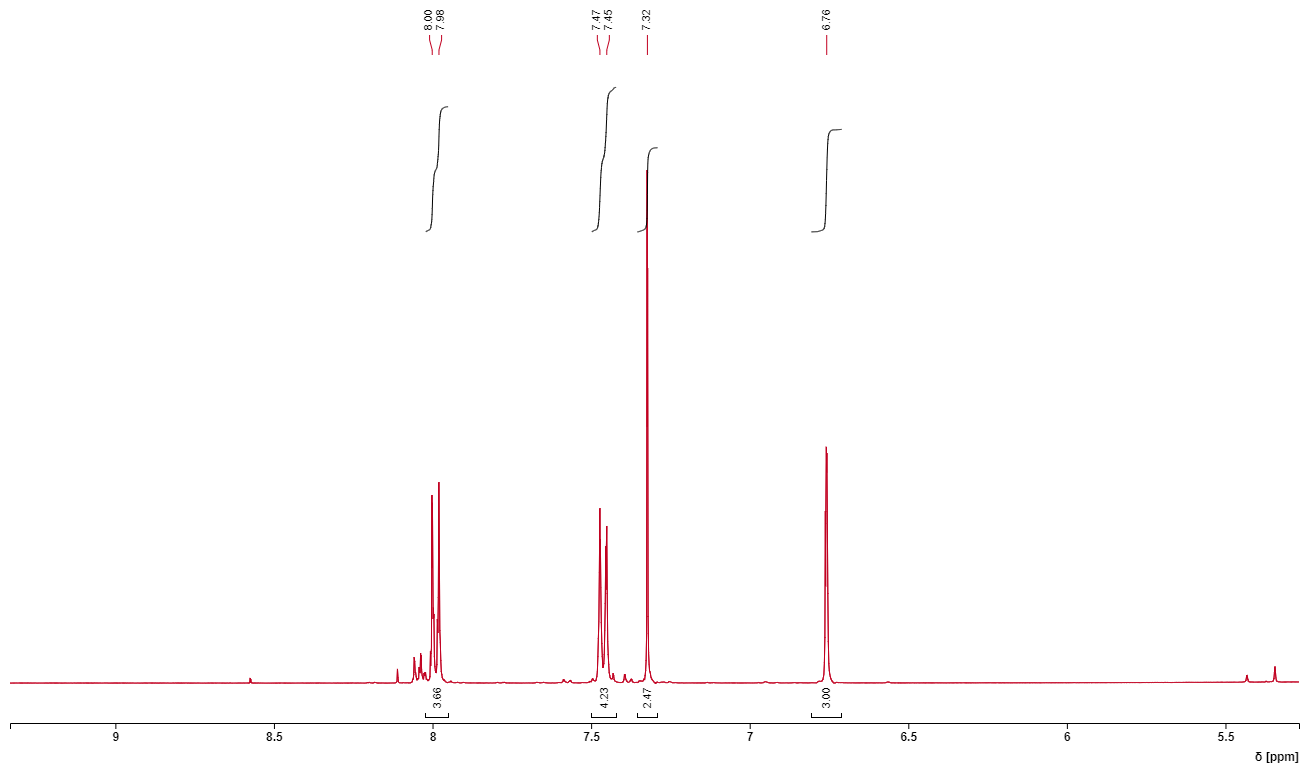


Figure S77. Fragment of ^1^H NMR (400 MHz, methanol-d4) spectrum of reaction mixture from Entry 7, Table S8.

### 2.3.7 Stage VII.

On the next stage of optimization we tried various additives to improve performance of the catalyst.

**General procedure K.**

Complex **1** (6.3 mg, 0.01 mmol) was weighted under air and placed into 8 mL MW vial and sealed. The vial was backfilled with argon and absolute 2-MeTHF (5 mL) was added via syringe. Polyethylene terephthalate (384.4 mg, 2 mmol, 1 eq.), was weighed under air, placed into a 8 mL glass MW vial with a stir bar and sealed. The vial was backfilled with argon and 2-MeTHF (3.5–4.4 mL) and dry EtOH (0.4–0.5 mL) or other alcohol (0.5 mL, Entries 10–13, Table S9) were added via syringe followed by additive (entries 7–9, 14, 15, Table S9). Next, 0.1 mL of prepared earlier solution of the catalyst and 0.1–0.2 mL of base solution (5–10 mol%, in THF or EtOH) was added into sealed MW 8-mL vial via syringe under inert gas. The MW vial with two needles on top were placed inside a 150 mL autoclave with some metal beans to ensure thermoconductivity. The autoclave was purged with argon, then sealed, purged with H_2_ (20 bar), pressurized with H_2_ at 50 bar, and placed in an oil bath preheated to 80 °C. The reaction was stirred at 70 or 80 °C for 18–44 h. After that, the autoclave was cooled down to room temperature in an ice bath and carefully vented to atmosphere. After the reaction mixture was diluted with 2 mL of MeOH and stirred for 10 min at room temperature, the vial was weighted. Then ≈200 µL of mesitylene were added to the vial with the reaction mixture and the solution was stirred for 10 min at room temperature. Next, 200–300 µL of the reaction mixture was added to NMR tube followed by the addition of MeOH-d4 (0.3 mL). Then, the mixture was analysed with ^1^H NMR.

| **Table S9. Hydrogenation of PET with 0.01 mol% 1 and various additives.***^[^****^a]^***   | | | | | | | | |
| --- | --- | --- | --- | --- | --- | --- | --- | --- |
| Entry | solvent | Additive | base | T, ºC | Time, h | Yield of BDM, % | Yield of EHMB, % | Yield of DETP, % |
| 1 | 4.5 mL 2-MeTHF/0.5 mL EtOH | - | 5% KO*t*Bu | 80 | 18 | 11±2^[b]^ | 84±3^[b]^ | 3±2^[b]^ |
| 2 | 4.5 mL 2-MeTHF/0.5 mL EtOH | - | 5% KOEt | 80 | 18 | 13 | 84 | 1 |
| 3 | 4.5 mL 2-MeTHF/0.5 mL EtOH | - | 5% KOtBu | 80 | 44 | 14 | 84 | <1 |
| 4 | 4.5 mL 2-MeTHF/0.5 mL EtOH | - | 5% KOtBu | 70 | 18 | 1 | 50 | 46 |
| 5 | 4.5 mL 2-MeTHF/0.5 mL EtOH | - | 10, KOtBu | 80 | 18 | 20 | 75 | <1 |
| 6 | 4.5 mL 2-MeTHF/0.5 mL EtOH | - | 10, KOtBu | 70 | 18 | 21 | 77 | <1 |
| 7 | 4.4 mL 2-MeTHF/0.5 mL EtOH | 0.1 mL Et_3_N | 5% KO*t*Bu | 80 | 18 | 7^[c]^ | 83^[c]^ | 8^[c]^ |
| 8 | 4.2 mL 2-MeTHF/0.5 mL EtOH | 0.3 mL Et_3_N | 5% KOtBu | 80 | 18 | 9 | 85 | 3 |
| 9 | 4.0 mL 2-MeTHF/0.5 mL EtOH | 0.5 mL Et_3_N | 5% KOtBu | 80 | 18 | 9 | 87 | 4 |
| 10 | 4.5 mL 2-MeTHF | 0.5 mL MeOH | 5% KO*t*Bu | 80 | 18 | 3 | 79 | 17 |
| 11 | 4.5 mL 2-MeTHF | 0.5 mL *i*PrOH | 5% KO*t*Bu | 80 | 18 | 11 | 84 | 2 |
| 12 | 4.5 mL 2-MeTHF | 0.5 mL *n*BuOH | 5% KO*t*Bu | 80 | 18 | 9 | 87 | 4 |
| 13 | 4.5 mL 2-MeTHF | 0.5 mL TFE^[d]^ | 5% KOtBu | 80 | 24 | <1 | <1 | 4 |
| 14 | 3.5 mL 2-MeTHF/0.5 mL EtOH | 1 mL tAmOH | 5% KOtBu | 80 | 24 | 10 | 86 | 3 |
| 15 | 4.5 mL 2-MeTHF/0.5 mL EtOH | 1.4 eq EG^[e]^ | 5% KOtBu | 80 | 24 | 5 | 77 | 14 |
| *[a]* General procedure K. *[b]* Confidence interval of 95% for 4 experiments was calculated. *[c]* Average of 2 experiments. *[d]* TFE = 2,2,2-trifluoroethanol. *[e]* EG = ethylene glycol | | | | | | | | |


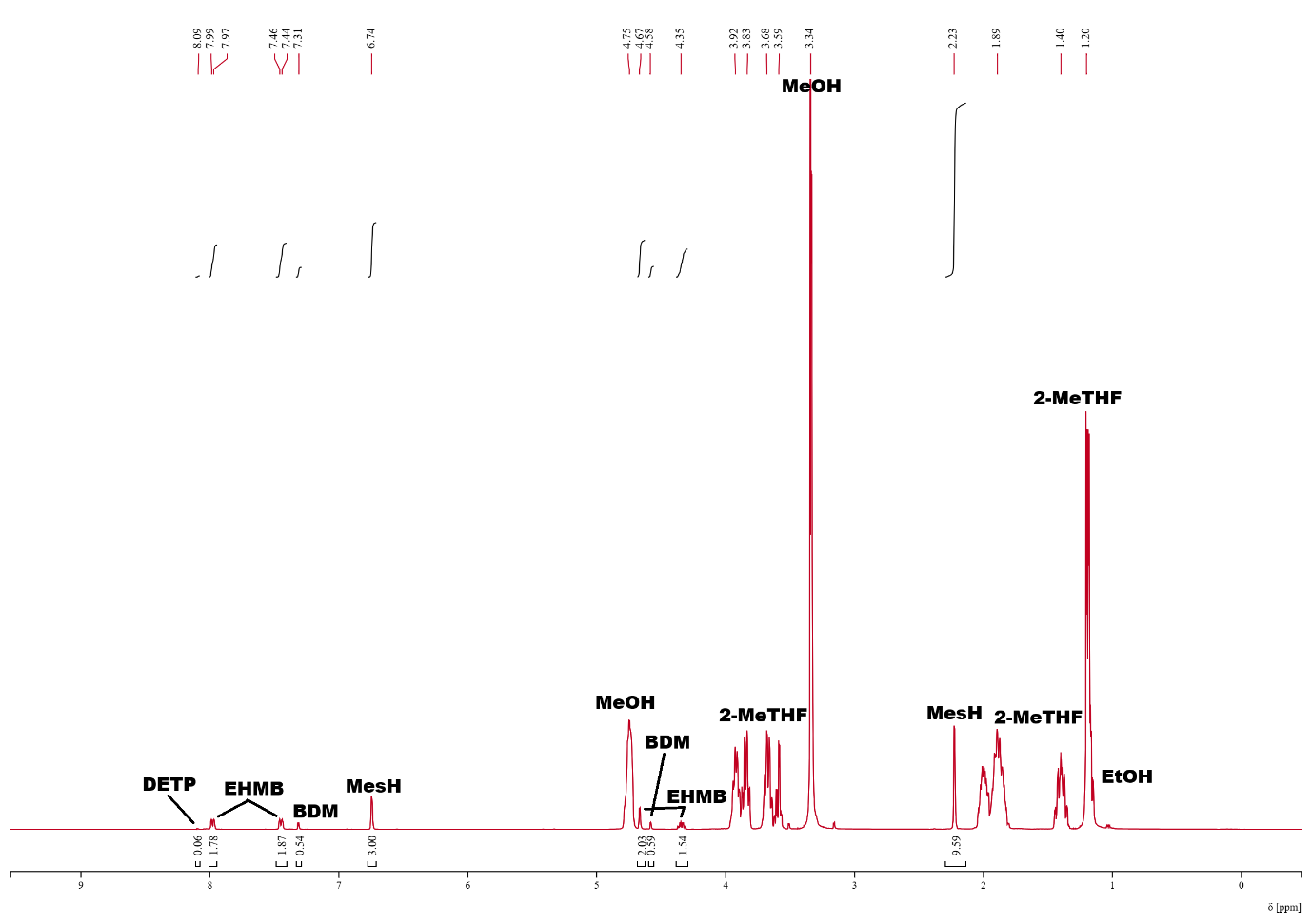


Figure S78. ^1^H NMR (400 MHz, methanol-d4) spectrum of one of the reaction mixtures from Entry 1, Table S9.


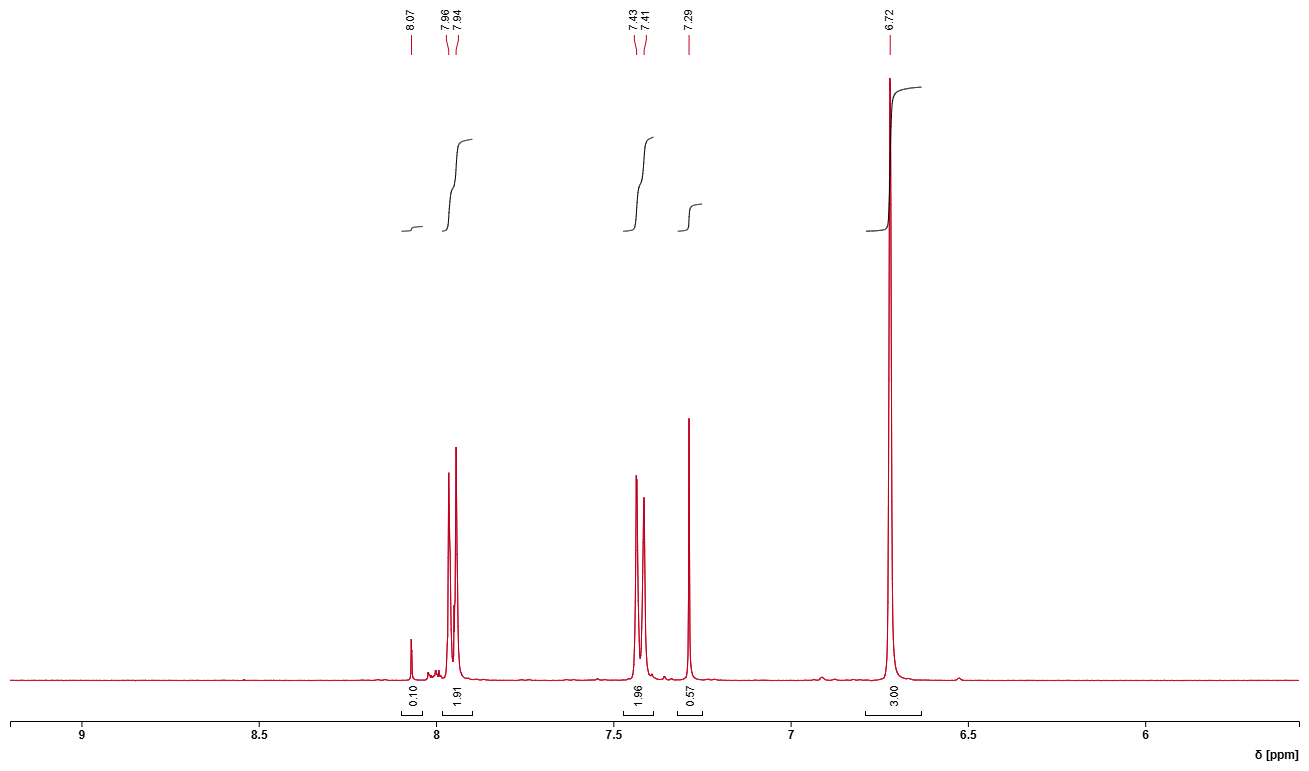


Figure S79. Fragment of ^1^H NMR (400 MHz, methanol-d4) spectrum of one of the reaction mixtures from Entry 1, Table S9.


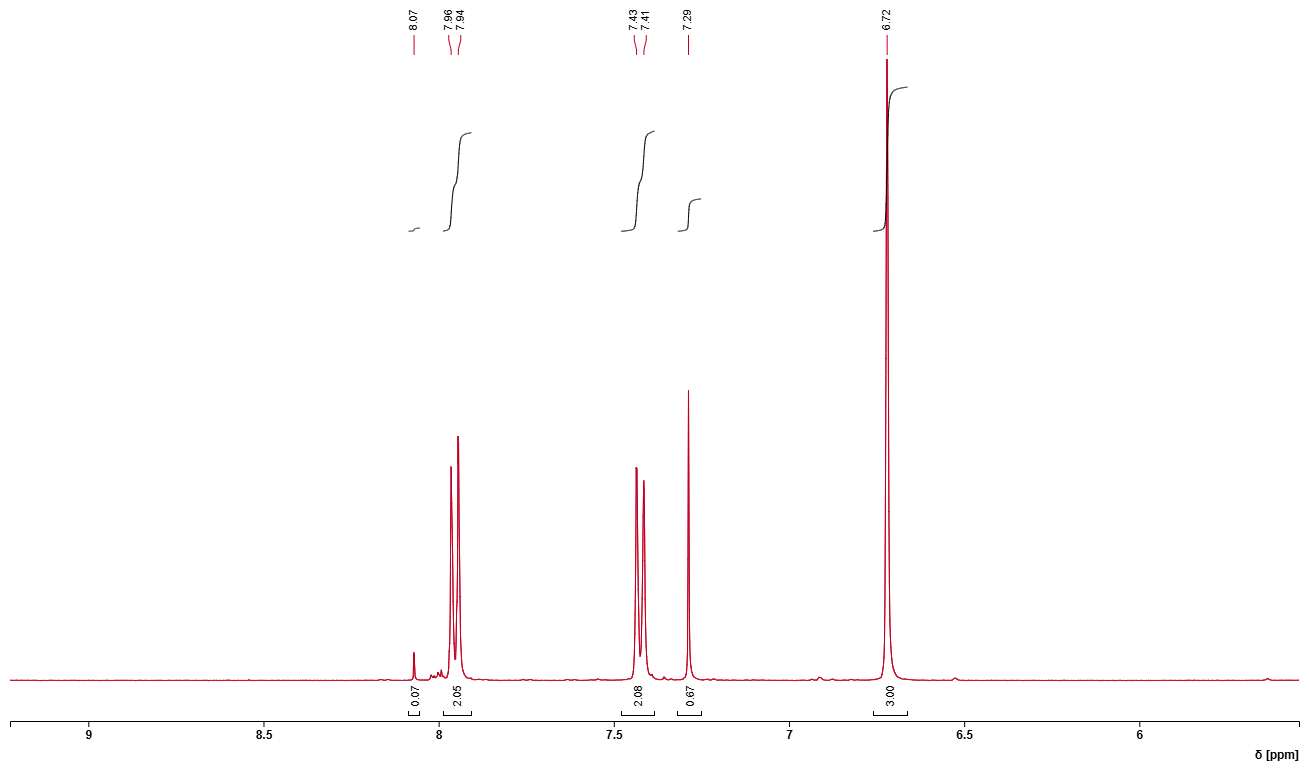


Figure S80. Fragment of ^1^H NMR (400 MHz, methanol-d4) spectrum of reaction mixture from Entry 2, Table S9.


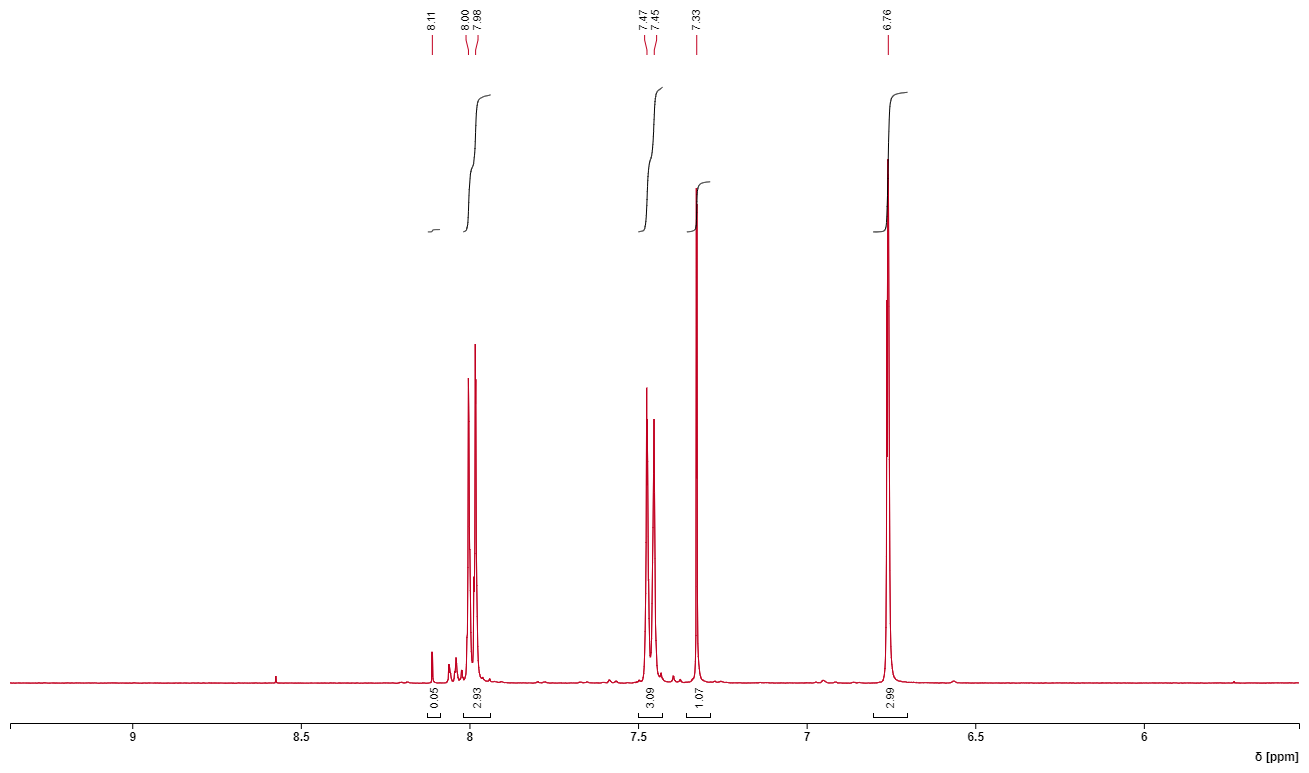


Figure S81. Fragment of ^1^H NMR (400 MHz, methanol-d4) spectrum of reaction mixture from Entry 3, Table S9.


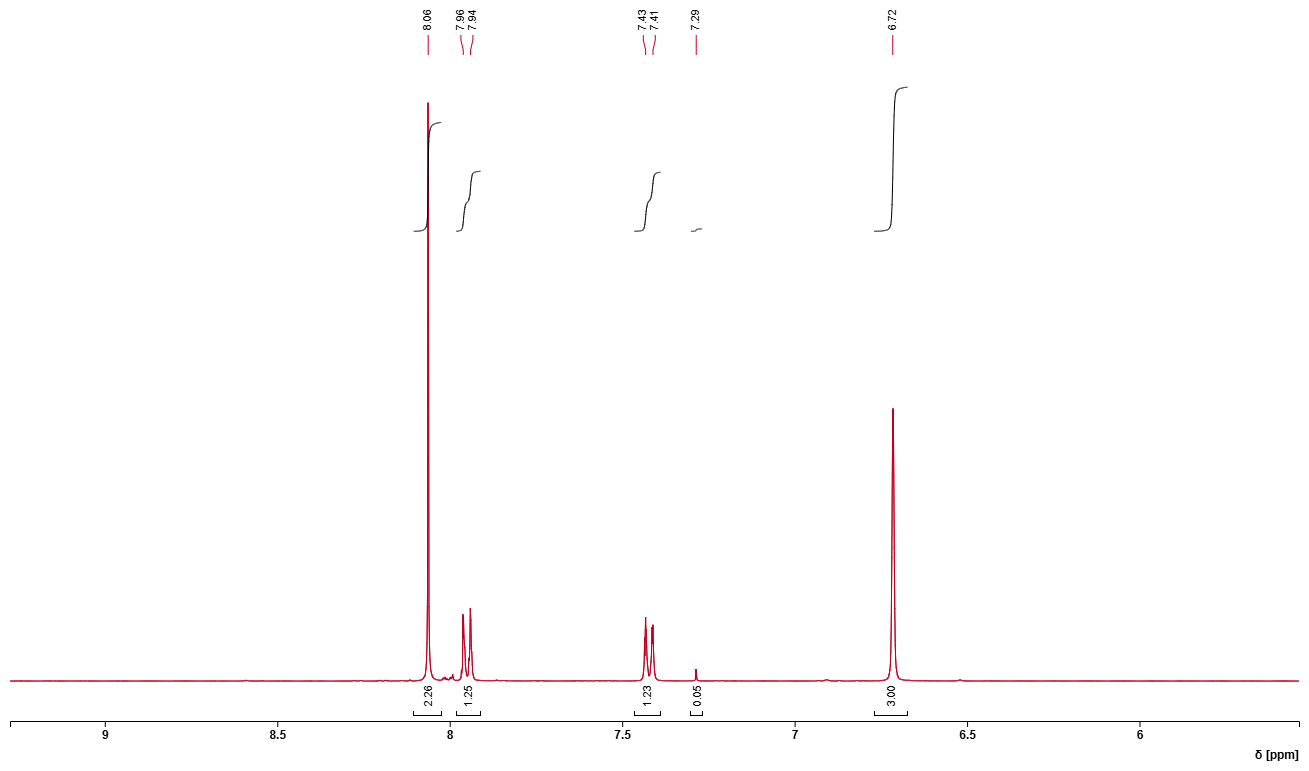


Figure S82. Fragment of ^1^H NMR (400 MHz, methanol-d4) spectrum of reaction mixture from Entry 4, Table S9.


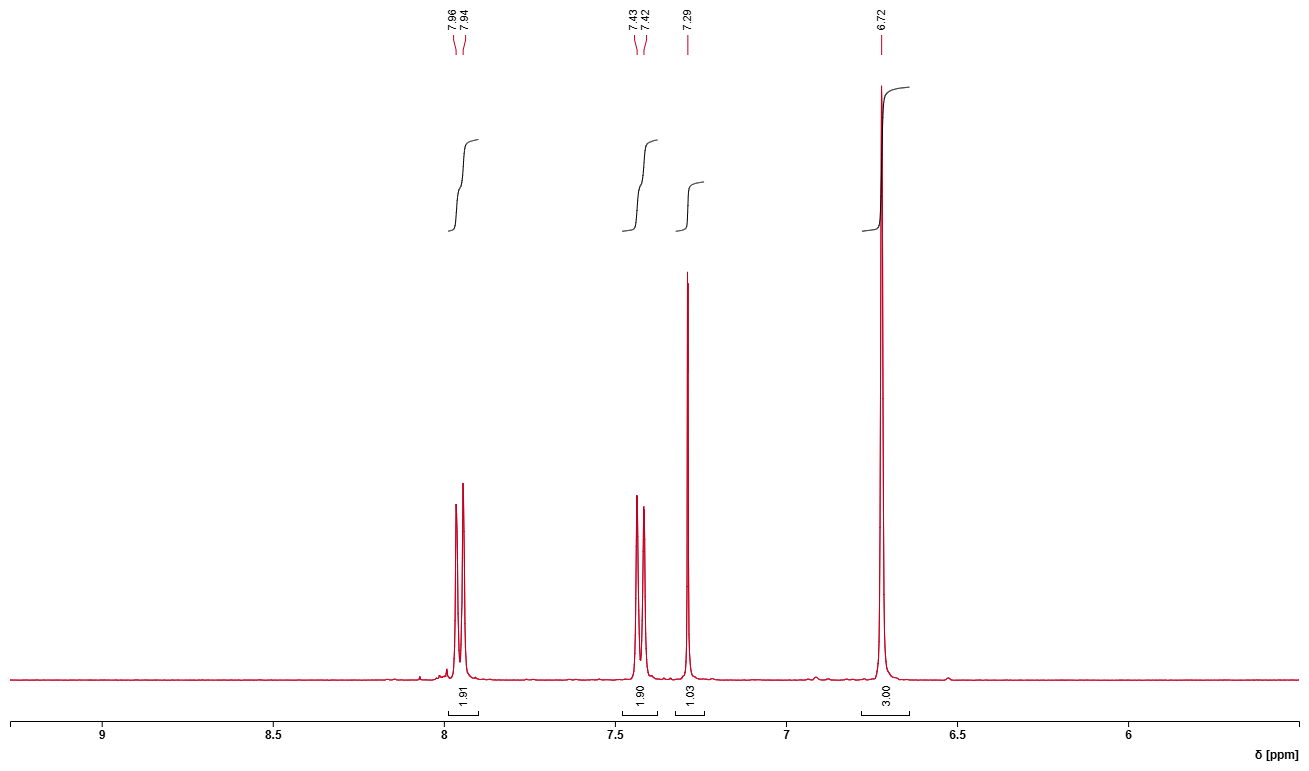


Figure S83. Fragment of ^1^H NMR (400 MHz, methanol-d4) spectrum of reaction mixture from Entry 5, Table S9.


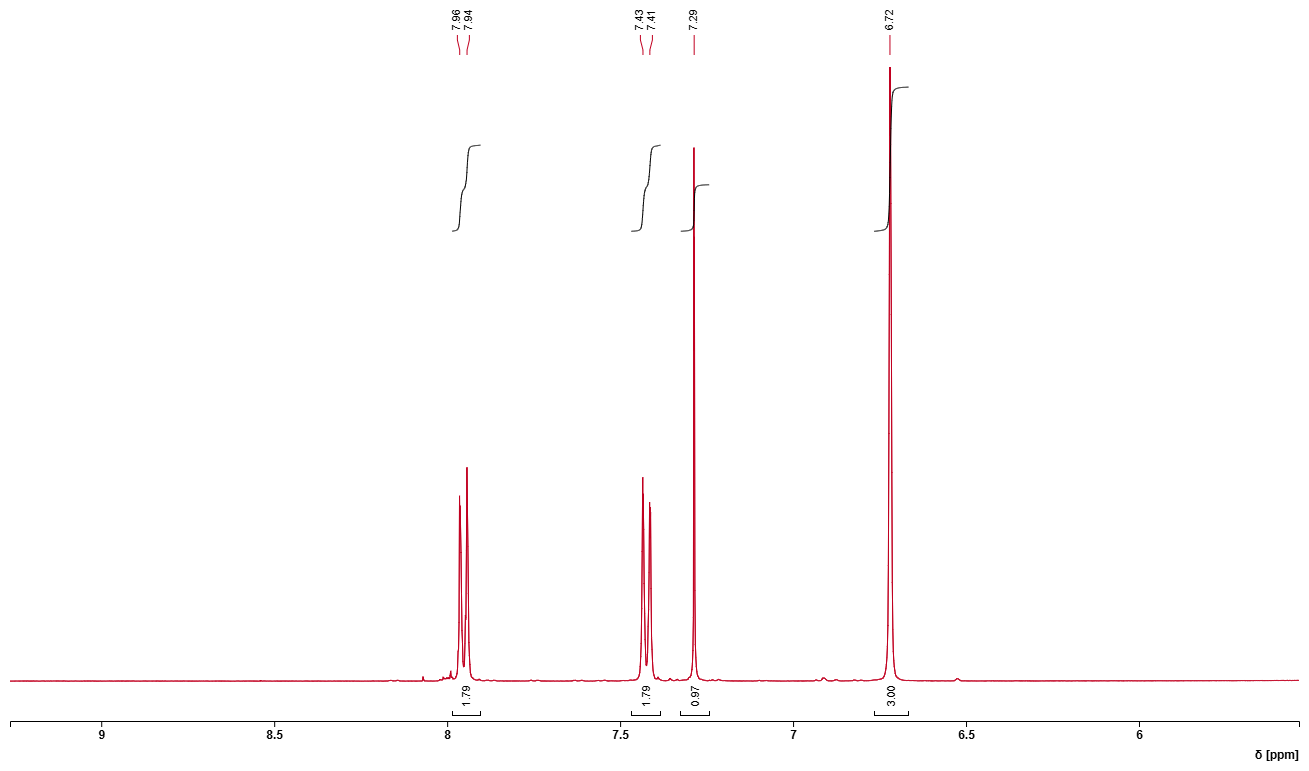


Figure S84. Fragment of ^1^H NMR (400 MHz, methanol-d4) spectrum of reaction mixture from Entry 6, Table S9.


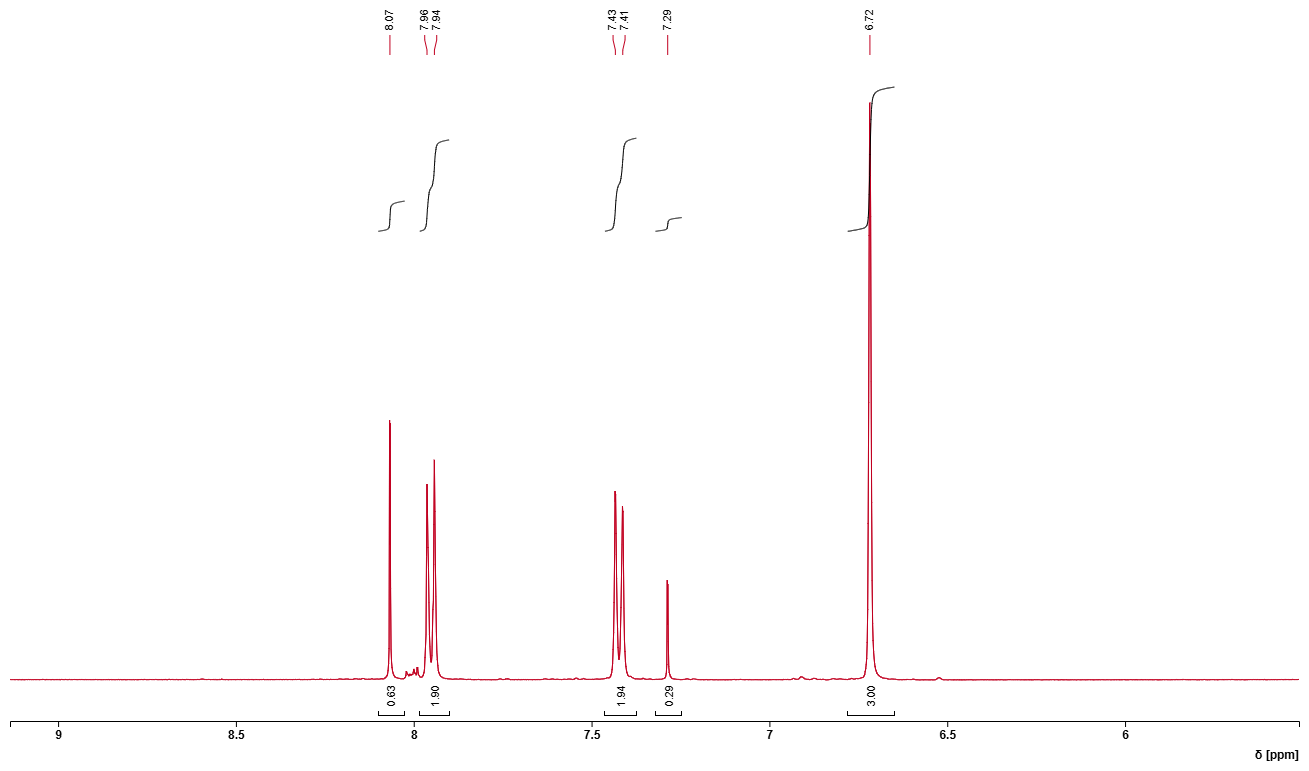


Figure S85. Fragment of ^1^H NMR (400 MHz, methanol-d4) spectrum of reaction mixture from Entry 7, Table S9.


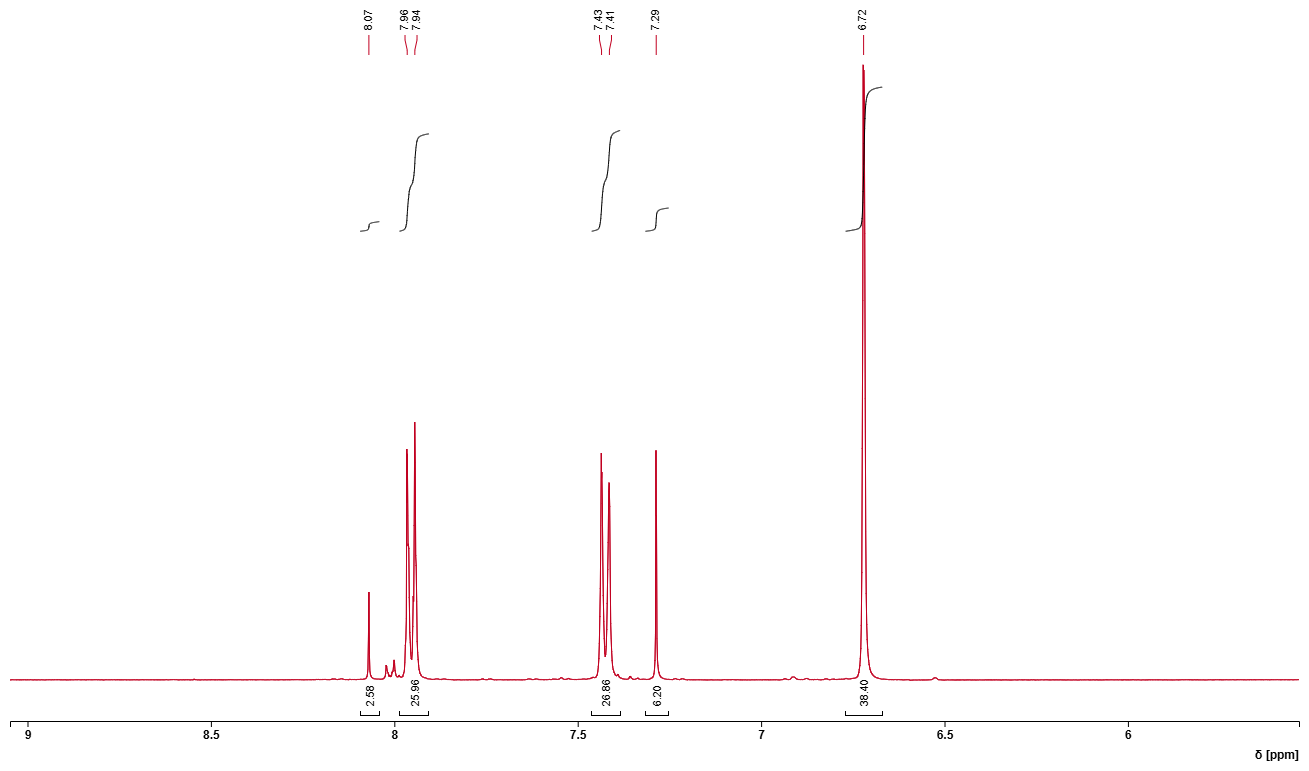


Figure S86. Fragment of ^1^H NMR (400 MHz, methanol-d4) spectrum of reaction mixture from Entry 8, Table S9.


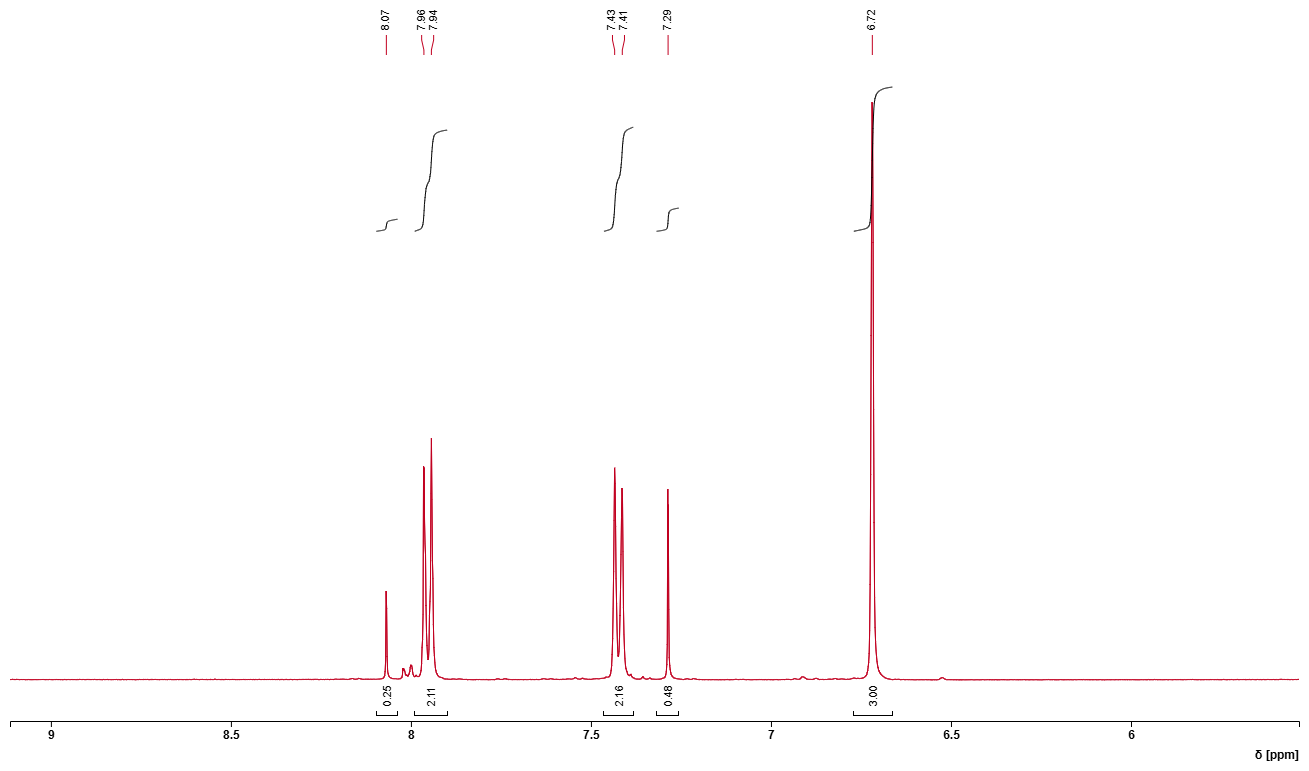


Figure S87. Fragment of ^1^H NMR (400 MHz, methanol-d4) spectrum of reaction mixture from Entry 9, Table S9.


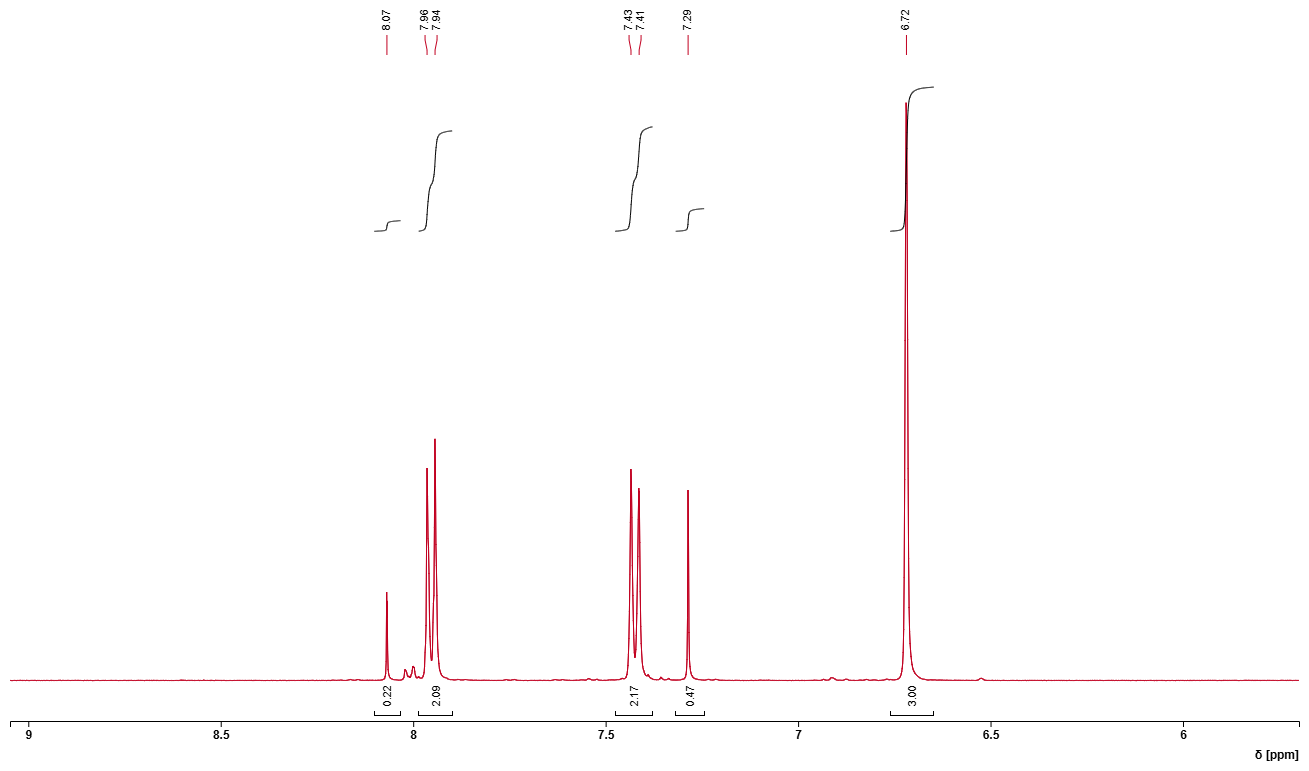


Figure S88. Fragment of ^1^H NMR (400 MHz, methanol-d4) spectrum of reaction mixture from Entry 10, Table S9.


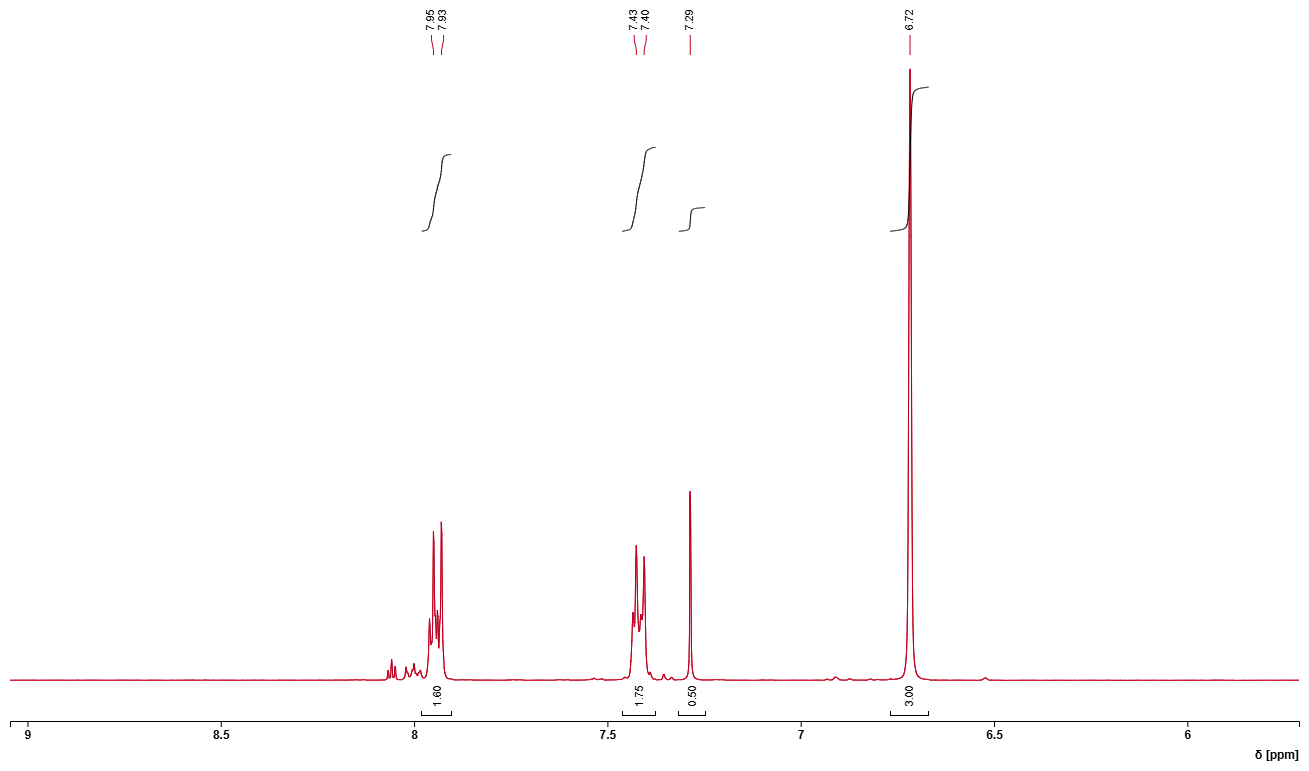


Figure S89. Fragment of ^1^H NMR (400 MHz, methanol-d4) spectrum of reaction mixture from Entry 11, Table S9.


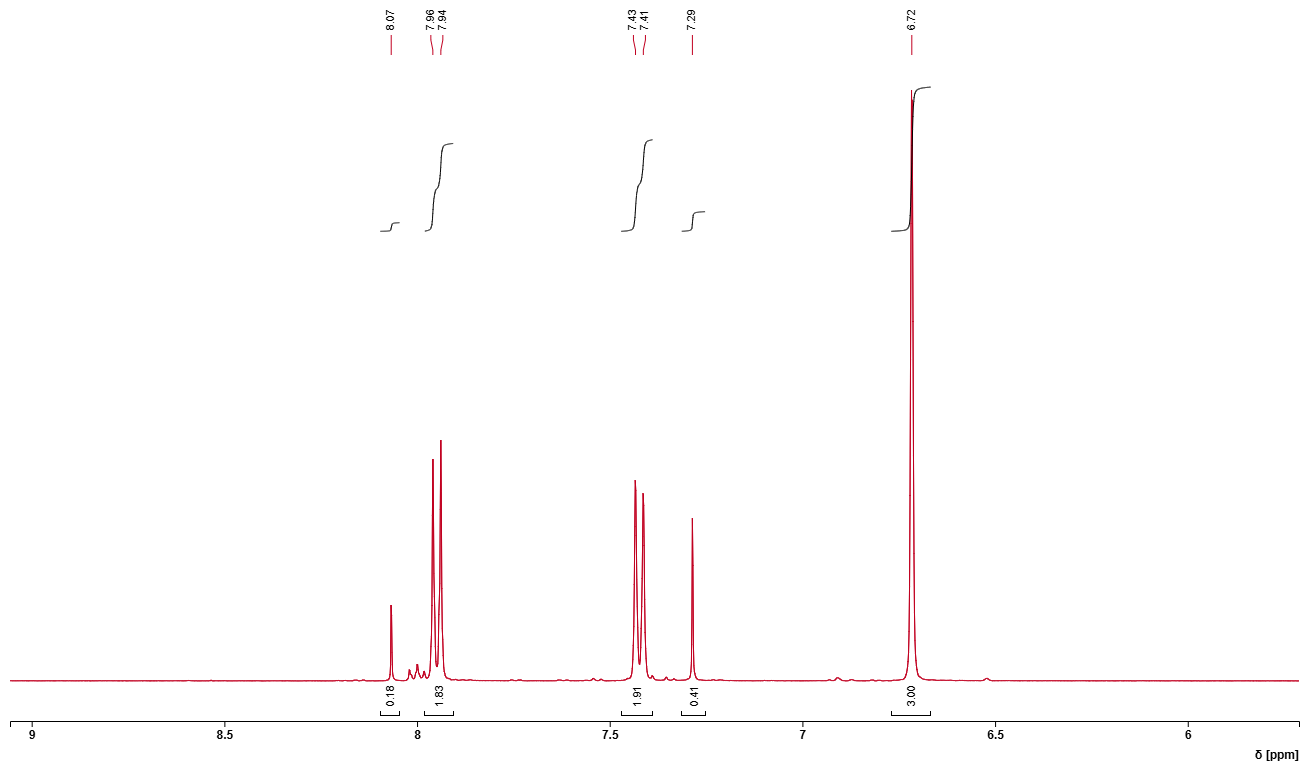


Figure S90. Fragment of ^1^H NMR (400 MHz, methanol-d4) spectrum of reaction mixture from Entry 12, Table S9.


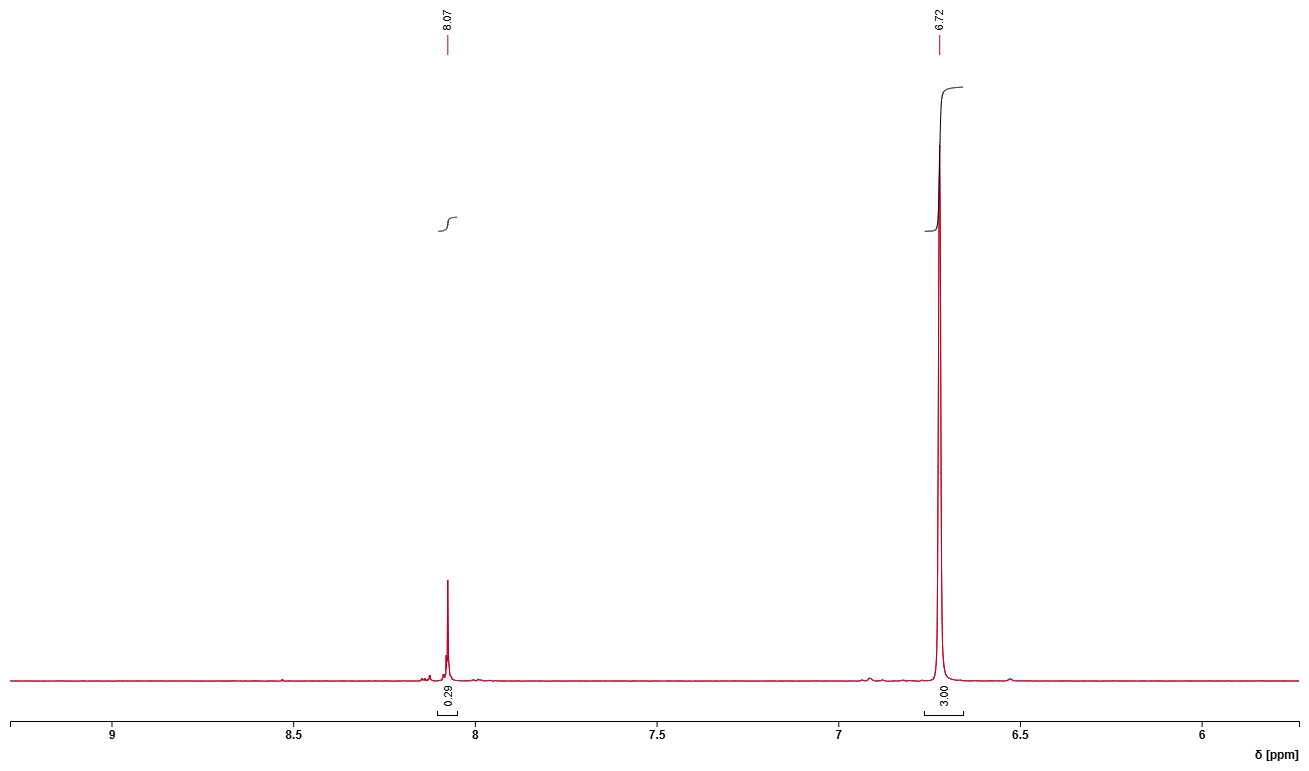


Figure S91. Fragment of ^1^H NMR (400 MHz, methanol-d4) spectrum of reaction mixture from Entry 13, Table S9.


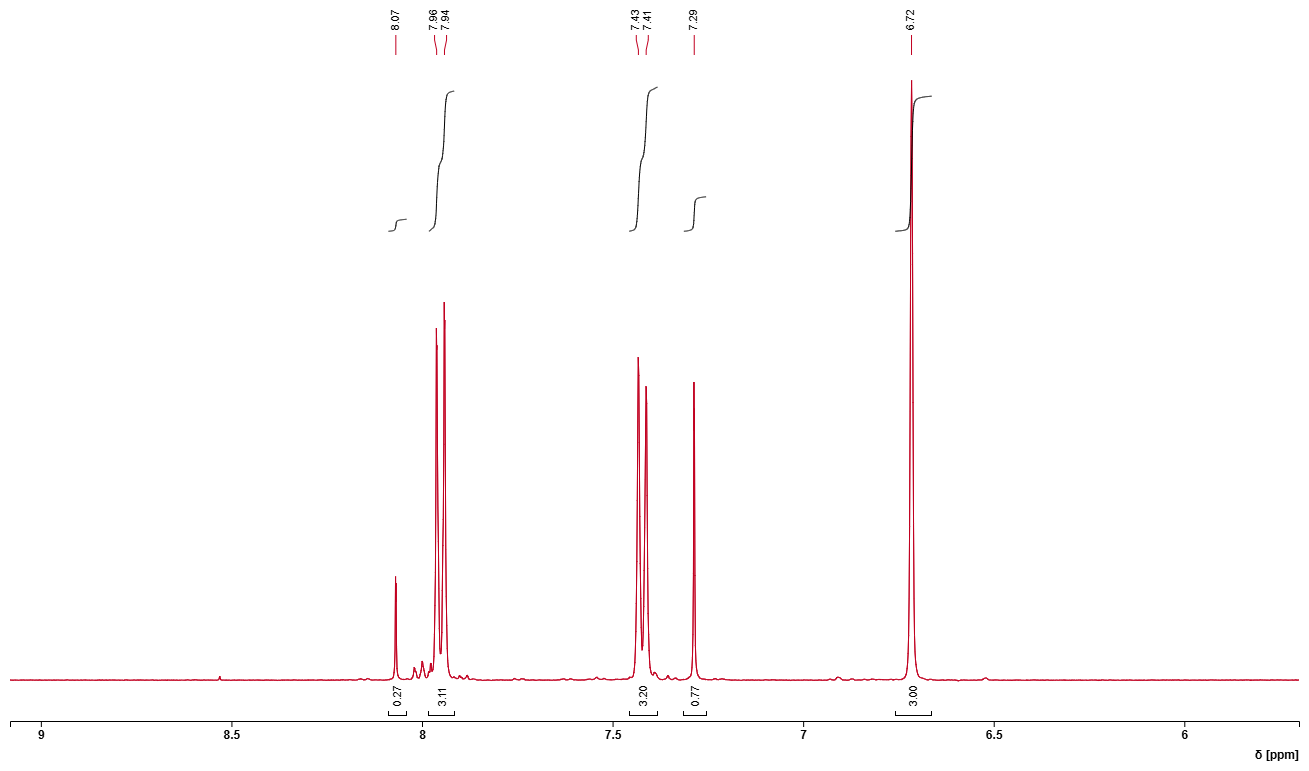


Figure S92. Fragment of ^1^H NMR (400 MHz, methanol-d4) spectrum of reaction mixture from Entry 14, Table S9.


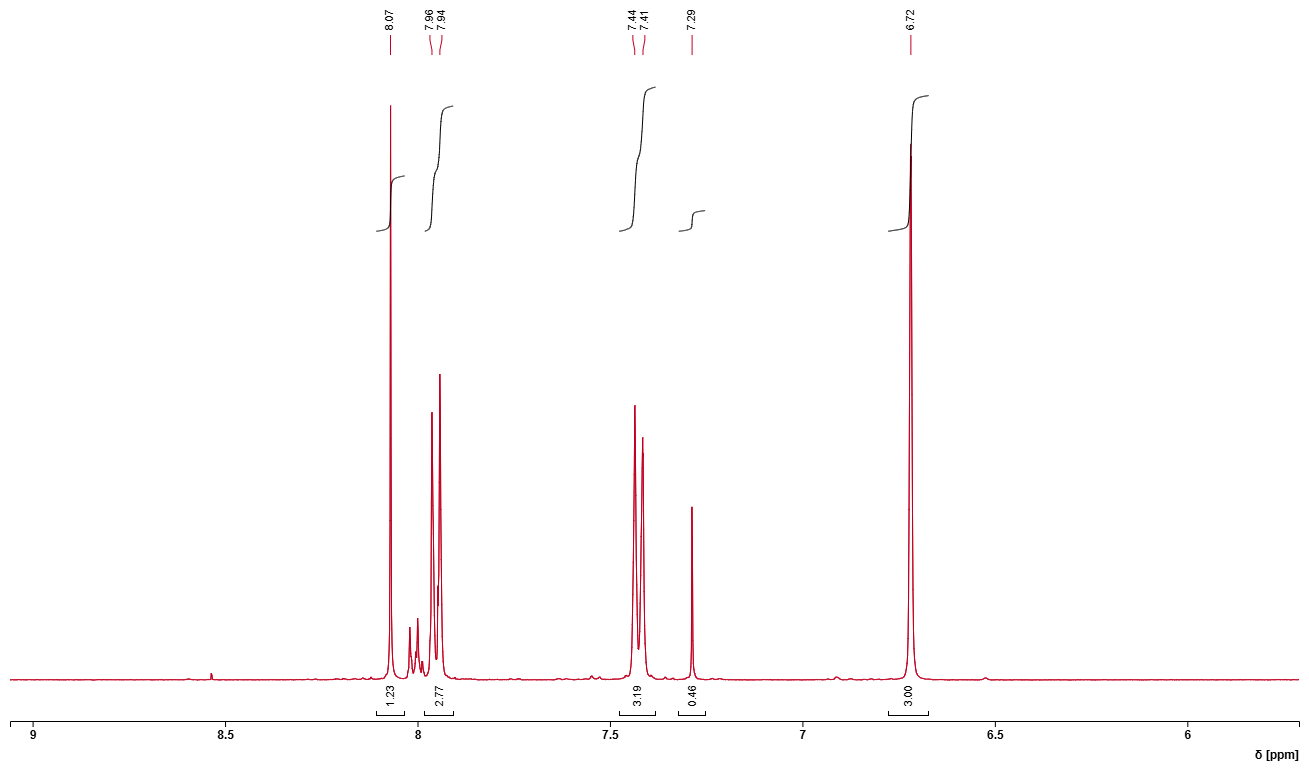


Figure S93. Fragment of ^1^H NMR (400 MHz, methanol-d4) spectrum of reaction mixture from Entry 15, Table S9.

### 2.3.8 Stage VIII.

On the next stage of optimization we reduced catalytic loadings to 0.005 mol% of **1** and tried various additives to improve the performance of the catalyst (Table S10). It was demonstrated that among all additives, only the additional amount of base helps to improve the yields of alcohols.

**General procedure L.**

Complex **1** (6.3 mg, 0.01 mmol) was weighed under air and placed into an 8 mL glass vial and sealed using a septum. The vial was purged with argon, and 2-MeTHF (5 mL) was added via syringe. Polyethylene terephthalate (384.4 mg, 2 mmol, 1 eq.) was weighed under air, placed into an 8 mL glass vial with a stir bar, and sealed. The vial was purged with argon and 2-MeTHF (3.5–4.4 mL) and EtOH (0.4–0.5 mL) or other alcohol (0.5 mL, Entries 10–13, Table S9) were added via syringe, followed by the additive (entries 7–9, 14, 15, Table S9). Next, 50 µL of prepared earlier solution of the catalyst and 0.1–0.4 mL of base solution (5–20 mol%, in THF or EtOH) were added into the sealed 8-mL vial (containing PET) via syringe under inert gas. Two needles were placed at the top of the vial, and it was then placed inside a 150 mL autoclave with some metal beads to ensure thermal conductivity. The autoclave was purged with argon, then sealed, purged with H_2_ (20 bar), pressurized with H_2_ at 50 bar, and placed in an oil bath preheated to 80 °C. The reaction was stirred at 70 or 80 °C for 18–44 h. After that, the autoclave was cooled down to room temperature in air and then in an ice bath and carefully vented to atmosphere. After the reaction mixture was diluted with 2 mL of MeOH and stirred for 10 min at room temperature, the vial was weighed. Then ≈200 µL of mesitylene was added to the vial with the reaction mixture, and the solution was stirred for 10 min at room temperature. Next, 200–300 µL of the reaction mixture was added to an NMR tube followed by the addition of MeOH-d4 (0.3 mL). The resulting mixture was analysed by ^1^H NMR spectroscopy.

| **Table S10. Hydrogenation of PET with 0.005 mol% 1 and various additives.***^[^****^a]^***   | | | | | | | | |
| --- | --- | --- | --- | --- | --- | --- | --- | --- |
| Entry | PET-p, mmol | solvent | Additive | KOtBu, mol% | Time, h | Yield of BDM, % | Yield of EHMB, % | Yield of DETP, % |
| 1 | 2 | 4.5 mL 2-MeTHF/0.5 mL EtOH | - | 5 | 18 | 3 | 74 | 20 |
| 2 | 2 | 4.5 mL 2-MeTHF/0.5 mL EtOH | - | 10 | 18 | 2 | 67 | 23 |
| 3 | 2 | 4.5 mL 2-MeTHF/0.5 mL EtOH | - | 20 | 18 | 7 | 82 | 7 |
| 4 | 2 | 4.5 mL 2-MeTHF/0.5 mL EtOH | - | 5 | 45 | 8±2^[b]^ | 85±3^[b]^ | 5±3^[b]^ |
| 5 | 4 | 4.5 mL 2-MeTHF/0.5 mL EtOH | - | 5 | 66 | 3 | 43 | 48 |
| 6 | 6 | 4.5 mL 2-MeTHF/0.5 mL EtOH | - | 5 | 66 | 13 | 45 | 32 |
| 7 | 2 | 4.4 mL 2-MeTHF/0.5 mL EtOH | 0.1 mL Et_3_N | 5 | 18 | 2 | 63 | 35 |
| 8 | 2 | 4.4 mL 2-MeTHF/0.5 mL EtOH | 0.1 mL Et_3_N | 5 | 62 | 12 | 87 | 1 |
| 9 | 4 | 4.4 mL 2-MeTHF/0.5 mL EtOH | 0.1 mL *n*HexSH | 5 | 24 | <1 | <1 | 99 |
| 10 | 4 | 4.4 mL 2-MeTHF/0.5 mL EtOH | 0.1 mL (EtO)_4_Si | 5 | 24 | <1 | <1 | 5 |
| 11 | 2 | 4.5 mL 2-MeTHF/0.5 mL EtOH | 20 mol% 18-crown-6 | 5 | 39 | <1 | <1 | 99 |
| 12 | 2 | 4.2 mL 2-MeTHF/0.8 mL EtOH | - | 5 | 39 | 1 | 52 | 42 |
| *[a]* General procedure L. *[b]* Confidence interval of 95% for 4 experiments was calculated. | | | | | | | | |


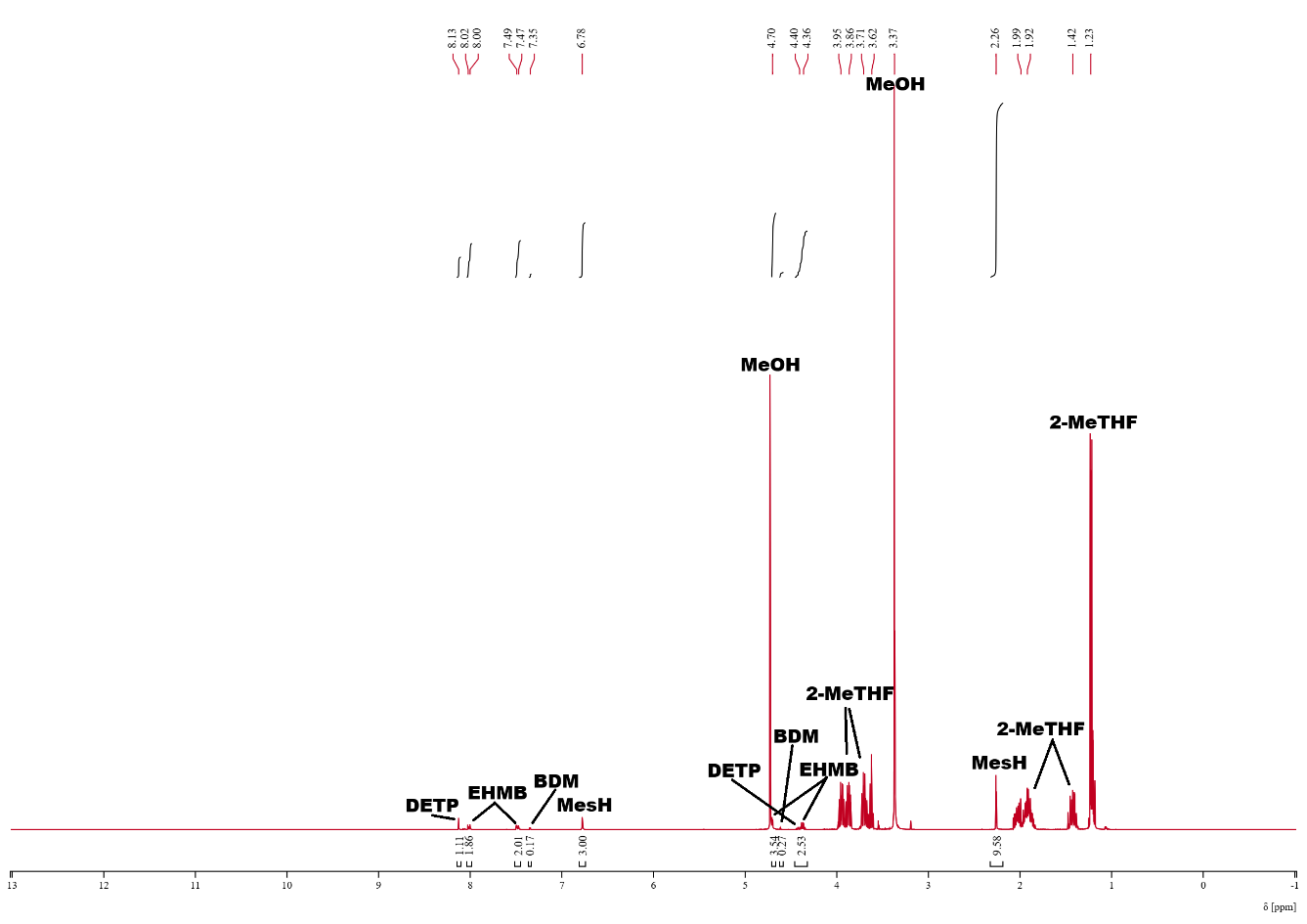


Figure S94. ^1^H NMR (400 MHz, methanol-d4) spectrum of reaction mixture from Entry 1, Table S10.


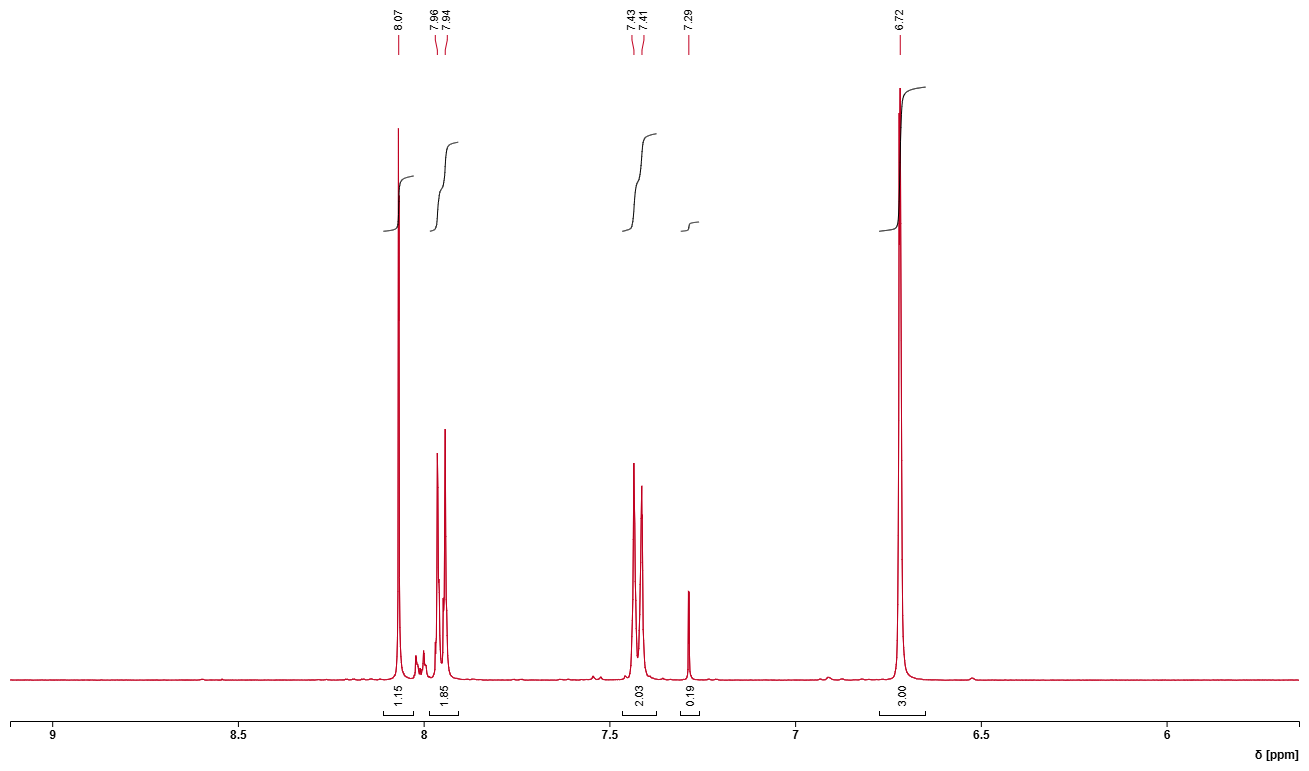


Figure S95. Fragment of ^1^H NMR (400 MHz, methanol-d4) spectrum of reaction mixture from Entry 1, Table S10.


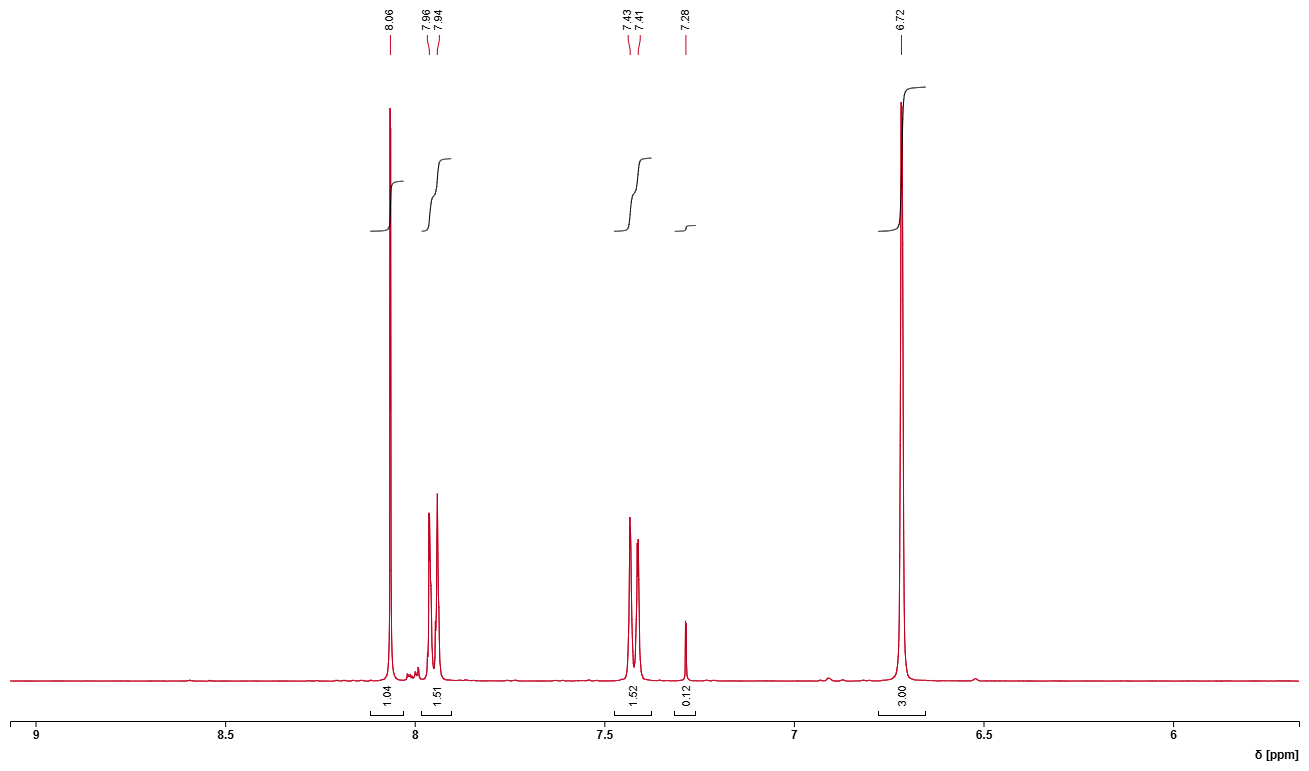


Figure S96. Fragment of ^1^H NMR (400 MHz, methanol-d4) spectrum of reaction mixture from Entry 2, Table S10.


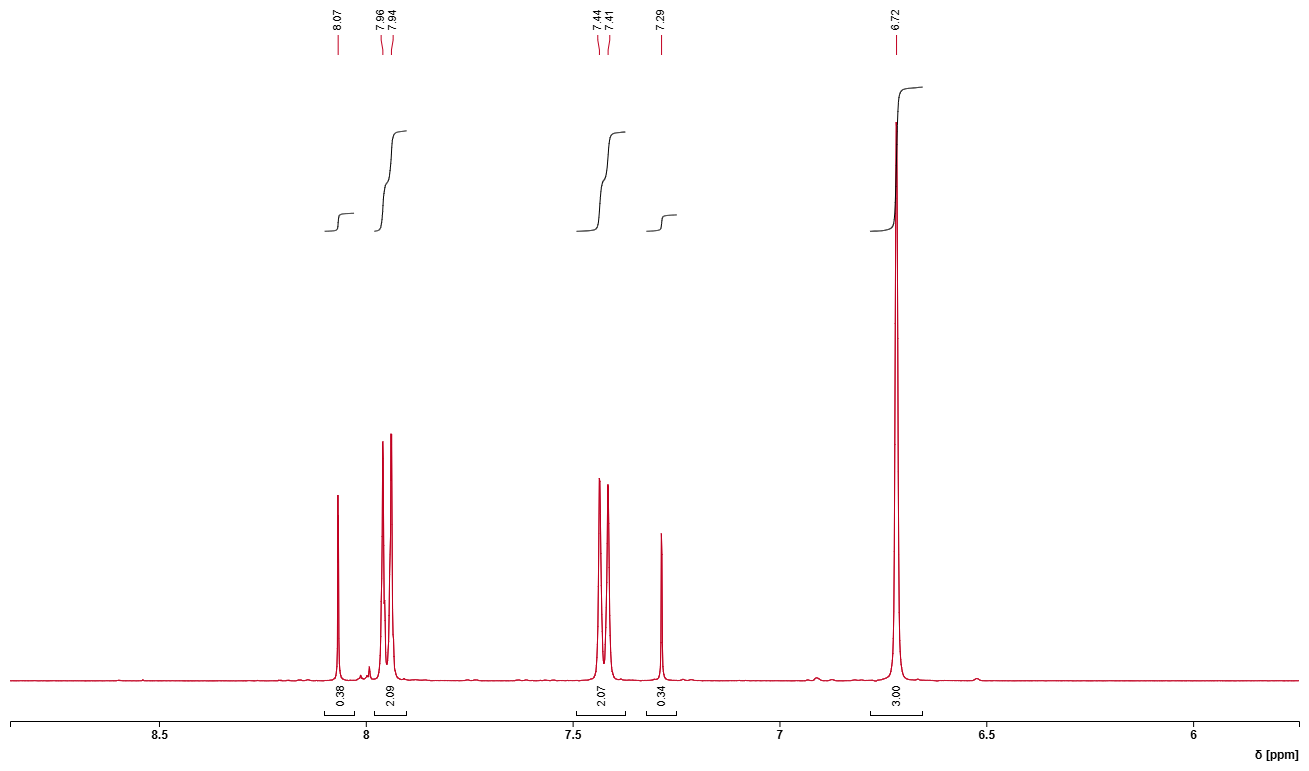


Figure S97. Fragment of ^1^H NMR (400 MHz, methanol-d4) spectrum of reaction mixture from Entry 3, Table S10.


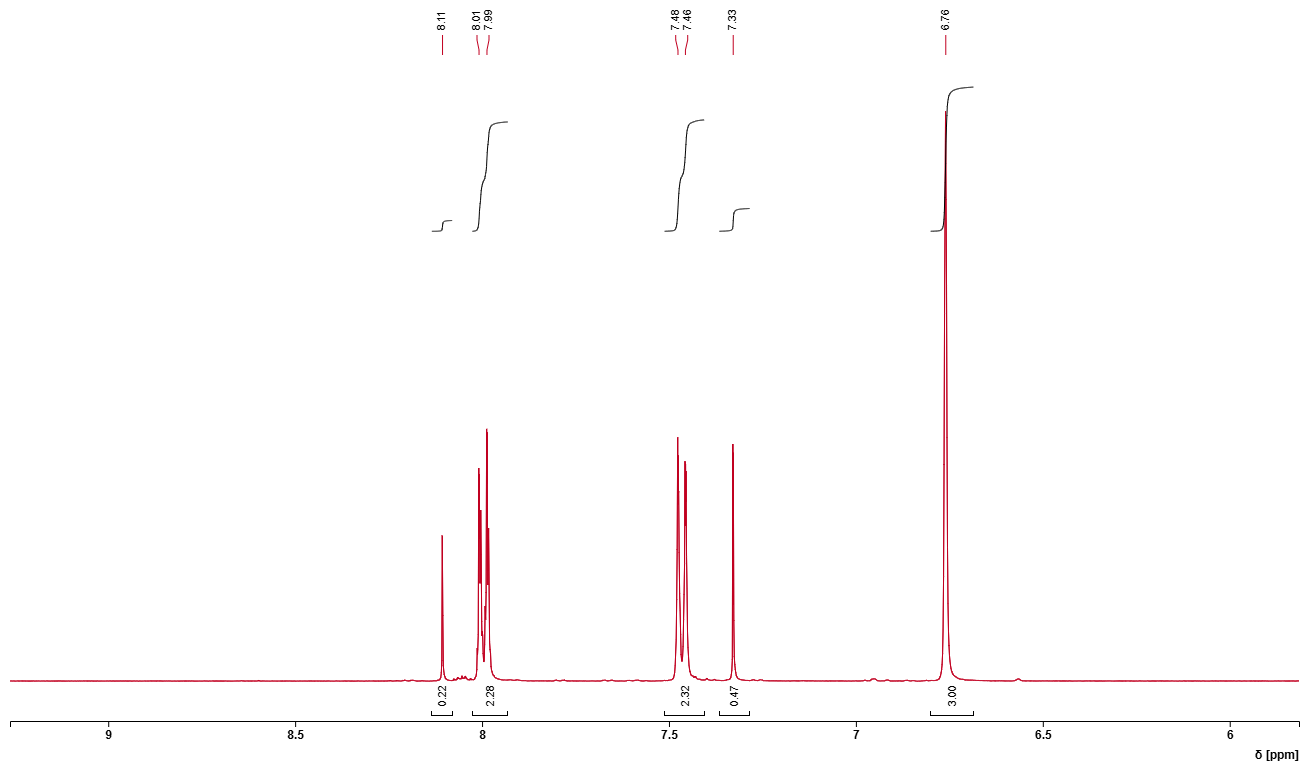


Figure S98. Fragment of ^1^H NMR (400 MHz, methanol-d4) spectrum of reaction mixture from Entry 4, Table S10.


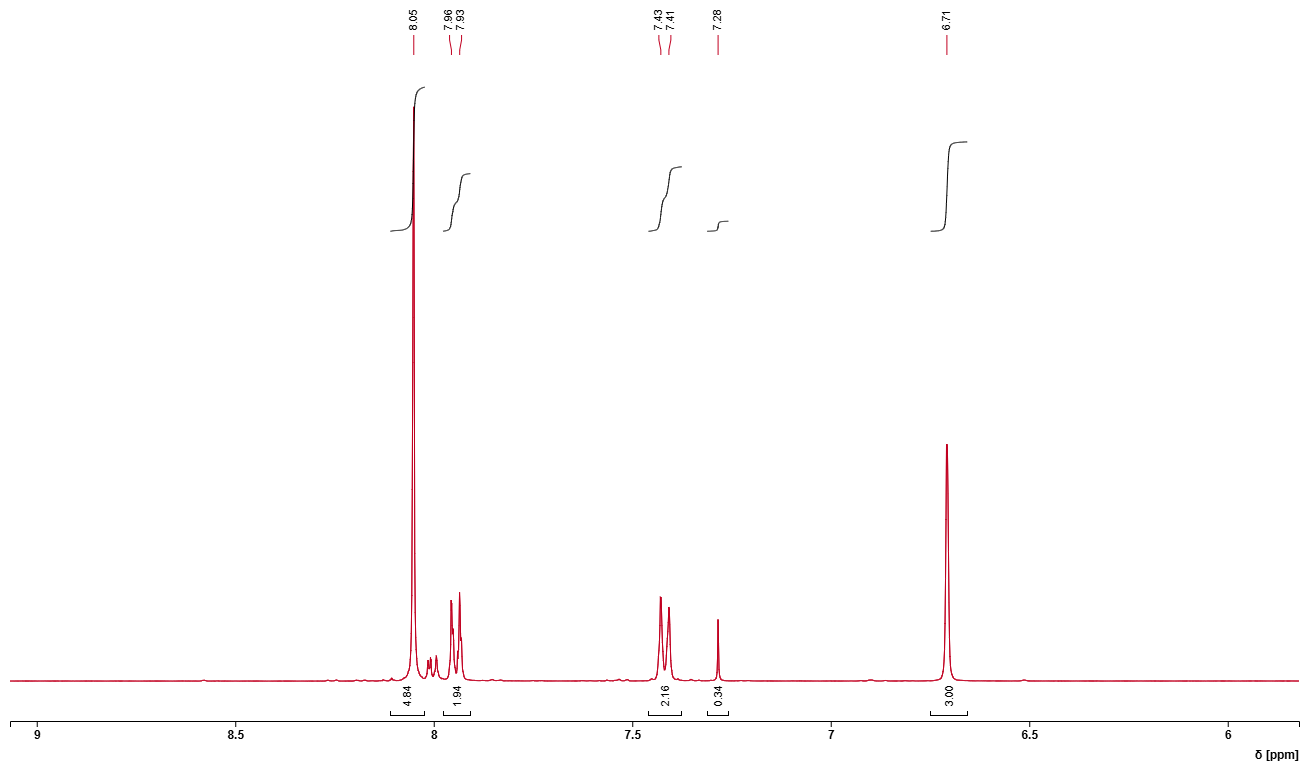


Figure S99. Fragment of ^1^H NMR (400 MHz, methanol-d4) spectrum of reaction mixture from Entry 5, Table S10.


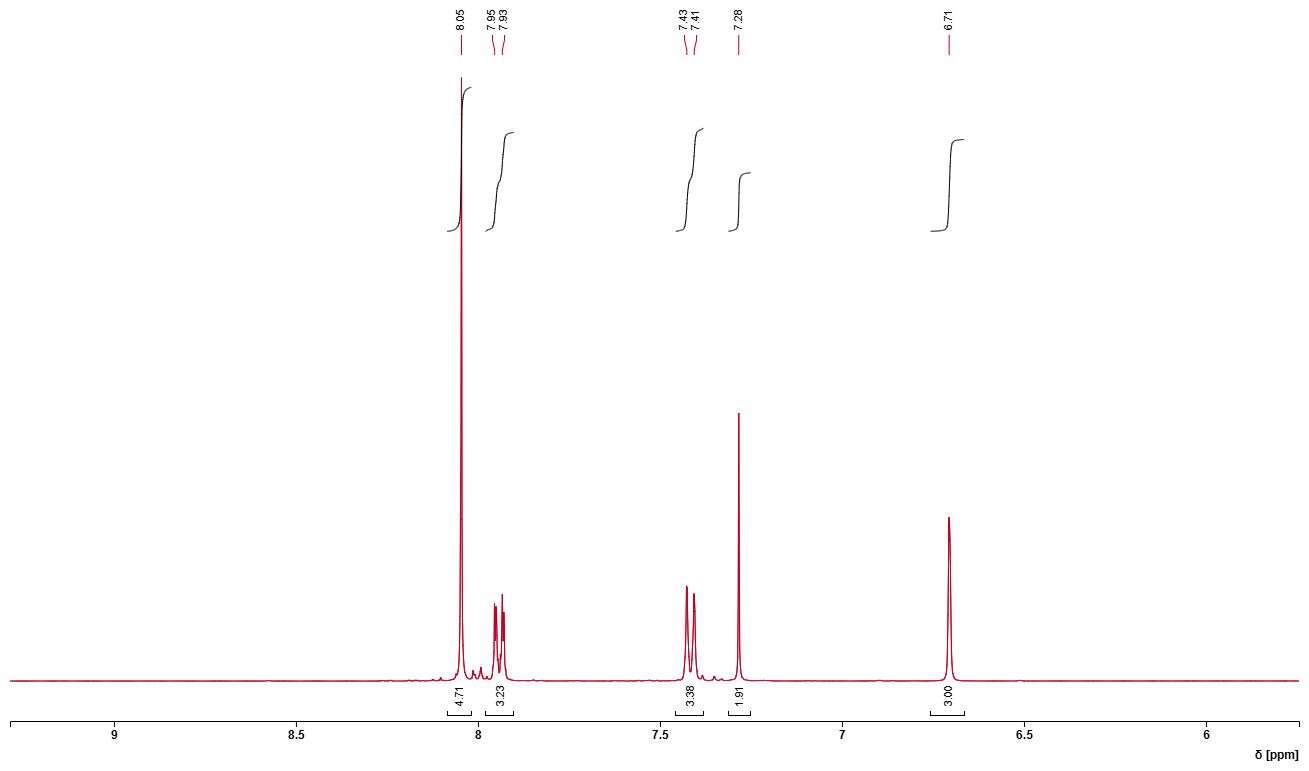


Figure S100. Fragment of ^1^H NMR (400 MHz, methanol-d4) spectrum of reaction mixture from Entry 6, Table S10.

Figure S101. Fragment of ^1^H NMR (400 MHz, methanol-d4) spectrum of reaction mixture from Entry 7, Table S10.

Figure S102. Fragment of ^1^H NMR (400 MHz, methanol-d4) spectrum of reaction mixture from Entry 8, Table S10.

Figure S103. Fragment of ^1^H NMR (400 MHz, methanol-d4) spectrum of reaction mixture from Entry 9, Table S10.

Figure S104. Fragment of ^1^H NMR (400 MHz, methanol-d4) spectrum of reaction mixture from Entry 10, Table S10.

Figure S105. Fragment of ^1^H NMR (400 MHz, methanol-d4) spectrum of reaction mixture from Entry 11, Table S10.

Figure S106. Fragment of ^1^H NMR (400 MHz, methanol-d4) spectrum of reaction mixture from Entry 12, Table S10.

# Hydrogenation of PET with 1 in dynamics

A series of experiments was carried out to estimate how fast the polymer dissolves in the chosen reaction medium. According to the data from Table S11, 2 hours is enough for the polymer to be dissolved even without a catalyst. The data also demonstrates (Figures S107, S108) that when 0.05 mol% of **1** is employed, the reaction is fastest in the first hour, while 0.005 mol% of **1** hydrogenate DETP more gradually for 62 hours.

| **Table S11. Hydrogenation of PET with 1 in dynamics.***^[^****^a]^*** | | | | | | |
| --- | --- | --- | --- | --- | --- | --- |
| Entry | Cat. **1**, mol% | Time, h | PET residue, % | Yield of BDM, % | Yield of EHMB, % | Yield of DETP, % |
| 1 | none | 0.5 | 17 | - | - | 81 |
| 2 | none | 1 | 7 | - | - | 92 |
| 3 | none | 2 | <1 | - | - | 99 |
| 4 | 0.005 | 0.5 | 14 | <1 | 7 | 76 |
| 5 | 0.005 | 1 | 6 | <1 | 9 | 84 |
| 6 | 0.005 | 2 | 2 | <1 | 21 | 75 |
| 7 | 0.005 | 18 | <1 | 3 | 74 | 20 |
| 8^[b]^ | 0.005 | 45 | <1 | 8±2 | 85±3 | 5±3 |
| 9 | 0.005 | 62 | <1 | 9 | 87 | 3 |
| 10 | 0.05 | 0.5 | 18 | 2 | 58 | 17 |
| 11 | 0.05 | 1 | 15 | 7 | 70 | 6 |
| 12 | 0.05 | 2 | 4 | 20 | 72 | 2 |
| 13^[b]^ | 0.05 | 18 | <1 | 72±12 | 26±12 | <1 |
| 14 | 0.05 | 67 | <1 | 88 | 11 | <1 |
| *[a]* General procedure L. *[b]* Confidence interval of 95% for 4 experiments was calculated. | | | | | | |

Figure S107. Reaction mixture composition based on ^1^H NMR with mesitylene as internal standard depending on time based on data from Table S11 for reaction with 0.05 mol% catalytic loadings. R3 – approximate time of the start of reaction regime R3 from Figure 3D.

Figure S108. Reaction mixture composition based on ^1^H NMR with mesitylene as internal standard depending on time based on data from Table S11 for reaction with 0.005 mol% catalytic loadings.

# Reactions of Ruthenium Hydrides

## 4.1 Synthesis of Ruthenium hydrides 1-EtOH and *fac-*1-H_2_

A mixture of complex **1** (1.0 g, 1.6 mmol) and NaOEt (325 mg, 4.6 mmol) in 15 mL of ethanol was refluxed for 1 h to give a yellow solution. The solvent was removed under reduced pressure (without heating) to give a dark yellow oil. The product was mixed with 20 mL of diethyl ether/toluene (3:1) and placed in a freezer for 1 h, then filtered through a glass frit, using 4–5 mL of hexane to wash the collected solids. The solids were then washed with toluene. The first filtrate was evaporated under vacuum to dryness (without heating), then the residue was triturated in diethyl ether, filtered, washed with hexane, and dried under vacuum for an hour to yield a yellow-orange solid. According to NMR data, the solid represents a mixture of **1-EtOH** and ***fac-*1-H_2_** in a ratio 95:5 (454 mg, 44 % yield). The second toluene filtrate was evaporated to dryness, triturated with hexane, filtered and dried under vacuum for 2 h, giving a yellow solid which, according to NMR, represents ***fac*-1-H_2_** (188 mg, 21% yield).

**1-EtOH**: ^1^H NMR (500 MHz, toluene-d8, 25 °C), *δ*: 7.94 (m, 8H, {PPh_3_}-H-ortho, O-H, N-H), 7.15 (m, 6H, {PPh_3_}H-meta), 7.07 (m, 3H, {PPh_3_}H-para), 3.89 (q, *J* = 6.8 Hz, 4H, OCH_2_), 1.75–3.14 (m, 12H, CH_2_), 1.38 (t, *J* = 6.8 Hz, 6H, **CH_3_**CH_2_O), 0.60–0.81 (br, 6H, **CH_3_**CH_2_S), –20.50 (br, RuH), -20.91 (d, *J* = 28.6 Hz, RuH).

^13^C{^1^H} NMR (125 MHz, toluene-d8), *δ*: 141.9, 141.6, 141.3, 134.2 (d, *J* = 11.0 Hz), 128.4, 127.6 (d, J = 8.6 Hz), 60.8, 53.6, 52.9, 50.6, 38.8, 37.4, 36.8, 36.1, 35.5, 30.7, 21.9, 14.2, 14.1, 13.4.

^31^P{^1^H} NMR (202 MHz, toluene-d8), *δ*: 69.05 (br. s), 67.63 (br. s), 67.51, (br.s).

***Fac-*1-H_2_**: ^1^H NMR (500 MHz, toluene-d8, 25 °C), *δ*: 8.54 (br.s, N-H), 7.91 (t, *J* = 8.3 Hz, 6H, {PPh_3_}-H-ortho), 7.18 (t, *J* = 7.4 Hz, 6H, {PPh_3_}H-meta), 7.08 (m, 3H, {PPh_3_}H-para), 1.89–3.40 (m, 8H, CH_2_), 2.00 (m, 2H, CH**_3_CH_2_**S), 1.80–1.88 (m, 2H, CH**_3_CH_2_**S), 0.87 (t, *J* = 7.3 Hz, 6H, **CH_3_**CH_2_S), -12.16 (d, *J* = 27.4 Hz, 2H, RuH).

^13^C{^1^H} NMR (125 MHz, toluene-d8), *δ*: 145.0 (d, *J* = 33.1 Hz), 134.2 (d, *J* = 11.1 Hz), 127.6, 127.30 (d, *J* = 8.3 Hz), 14.4.

^31^P{^1^H} NMR (202 MHz, toluene-d8), *δ*: 81.16.

Figure S109. ^1^H NMR (500 MHz, 25 °C, toluene-d8) spectrum of complex 1-EtOH.

Figure S110. ^13^C{^1^H} NMR (125 MHz, 25 °C, toluene-d8) spectrum of complex 1-EtOH.

Figure S111. ^31^P{^1^H} NMR (202 MHz, 25 °C, toluene-d8) spectrum of complex 1-EtOH.

Figure S112. ^1^H NMR (500 MHz, 25 °C, toluene-d8) spectrum of complex *fac-*1-H_2_.

Figure S113. ^13^C{^1^H} NMR (125 MHz, 25 °C, toluene-d8) of complex f*ac-*1-H_2_.

Figure S114. ^31^P{^1^H} NMR (202 MHz, 25 °C, toluene-d8) of complex *fac-*1-H_2_.

## 4.2 PET hydrogenation using 1, 1-EtOH, and *fac*-1-H_2_.

**General procedure M.**

Complex **1** (6.3 mg, 0.01 mmol) or **1-EtOH** (6.5 mg, 0.01 mmol) or ***fac*-1-H_2_** (5.6 mg, 0.01 mmol) was weighed in a glovebox under argon and placed into an 8 mL glass vial and sealed. 2-MeTHF (5 mL) was added to the vial under argon via syringe. Polyethylene terephthalate (384.4 mg, 2 mmol, 1 eq.), was weighed in a glovebox, placed into an 8 mL glass vial with a stir bar, and sealed. To the vial, 2-MeTHF (3.9 mL) and EtOH (0.5 mL) were added via syringe, followed by 500 µL of the prepared earlier solution of the catalyst and 0.1 mL of KO*t*Bu solution (5 mol%, in THF). Two needles were added at the top of the vial, and then it was placed inside a 150 mL autoclave with some metal beads to ensure thermal conductivity. The autoclave was purged with argon, then sealed, purged with H_2_ (20 bar), pressurized with H_2_ at 50 bar, and placed in an oil bath preheated to 80 °C. The reaction was stirred at 80 °C for 1 h. After that, the autoclave was cooled down to room temperature in an ice bath and carefully vented to atmosphere. After the reaction mixture was diluted with 2 mL of MeOH and stirred for 10 min at room temperature, the vial was weighed. Then ≈200 µL of mesitylene was added to the vial with the reaction mixture, and the solution was stirred for 10 min at room temperature. Next, 200–300 µL of the reaction mixture was added to an NMR tube, followed by the addition of MeOH-d4 (0.3 mL). Then, the mixture was analysed by ^1^H NMR spectroscopy.

| **Table S12. Hydrogenation of PET with 1, 1-EtOH, *fac-*1-H_2_.***^[^****^a]^*** | | | | | |
| --- | --- | --- | --- | --- | --- |
| Entry | Cat. | PET residue, % | Yield of BDM, % | Yield of EHMB, % | Yield of DETP, % |
| 1 | **1** | 11 | 10 | 72 | 4 |
| 2 | **1-EtOH** | 15 | 12 | 65 | 5 |
| 3 | ***Fac*-1-H_2_** | 14 | 15 | 65 | 4 |
| 4 | **1-EtOH^[b]^** | 98 | 1 | 1 | <1 |
| 5 | ***Fac*-1-H_2_^[b]^** | 95 | 1 | 1 | <1 |
| 6^[c]^ | **1-EtOH** | <1 | <1 | <1 | 96 |
| *[a]* General procedure M. *[b]* Without any base. *[c]* Reaction was run in a closed Young’s flask without any hydrogen. | | | | | |

## 4.3 Stoichiometric reactions of Ruthenium hydrides with esters.

### 4.3.1 Reaction of complex 1-EtOH with DETP.

Complex **1-EtOH** (27 mg, 0.042 mmol) was dissolved in 0.6 mL toluene-d8 in a Young’s NMR tube. DETP (9.0 mg, 0.042 mmol) was added along with 0.2 mL of toluene-d8 and mesitylene (4.7 mg, 0.039 mmol) as internal standard. ^1^H and ^31^P{^1^H} NMR spectra were recorded and demonstrated full conversion of DETP, formation of EtOAc, **1-EHMB** with approx. 86% yield and **1-BDM** with approx. 8% yield.

**1-EHMB + 1-BDM:** ^1^H NMR (500 MHz, toluene-d8, 25 °C), *δ*: 8.27 (d, J = 8.1 Hz, 2H), 7.92 (m, 7H), 7.61 (m, 2H), 7.44 (s, **1-BDM**) 7.09 (m, 9H), 4.99 (br.s, 2H), 4.77 (s, **1-BDM**) 4.15 (q, J = 7.1 Hz, 2H), 3.26 (br.s, 1H), 2.66–2.75 (m, 3H), 2.38–2.48 (m, 4H), 2.18–2.23 (m, 3H), 1.70–1.83 (m, 1H), 1.08 (t, J = 7.1 Hz, 3H), 0.66-0.78 (m, 6H), -20.73- -20.38 (m, 1H). ^13^C{^1^H} NMR (126 MHz, toluene-d8, 25 °C), *δ*: 166.7, 145.9 (d, J = 33.2 Hz), 141.1 (d, J = 36.5 Hz), 134.5 (d, J = 10.9 Hz), 134.3 (d, J = 11.2 Hz), 129.7, 129.6, 128.5, 128.3, 127.7 (d, J = 8.7 Hz), 127.3 (d, J = 8.4 Hz), 126.6, 126.4, 66.7, 66.5, 60.3, 58.9, 52.8, 49.6, 39.0, 36.5, 36.3, 30.7, 14.4, 14.0, 13.3. ^31^P{^1^H} NMR (202 MHz, toluene-d8, 25 °C), *δ*: 68.3 (br. s), 67.1 (br. s), 67.0, (br.s). HRMS (ESI) m/z: [C_26_H_35_NPRuS_2_ (without alkoxide)]^+^ calcd: 558.0987, found: 558.0991; m/z: [C_10_H_11_O_3_ (EHMB alkoxide)]^–^ calcd: 179.0713, found: 179.0717.

Figure S115. ^1^H NMR (500 MHz, 25 °C, toluene-d8) of mixture of complex 1-EtOH and DETP after keeping it at room temperature for 1 h. EA – ethyl acetate; MesH – mesitylene (internal standard).

Figure S116. ^31^P{^1^H} NMR (202 MHz, 25 °C, toluene-d8) of a mixture of complex 1-EtOH and DETP after keeping it at room temperature for 1 h.

Figure S117. ^13^C{^1^H} DEPTQ NMR (126 MHz, 25 °C, toluene-d8) of a mixture of complex 1-EtOH and DETP after keeping it at room temperature for 1 h.

### 4.3.2 Reaction of 1-EtOH with EHMB.

Complex **1-EtOH** (28 mg, 0.043 mmol) was dissolved in 0.6 mL toluene-d8 in a Young’s NMR tube. EHMB (7.8 mg, 0.043 mmol) was added along with 0.2 mL of toluene-d8. ^1^H, ^13^C{^1^H} and ^31^P{^1^H} NMR spectra were recorded and demonstrated formation of EtOAc, and a species interpreted as **1-EHMB** with approx. 63% yield and **1-BDM** with approx. 31% yield. After keeping the resulting solution 2 days at room temperature, yellow crystals were formed. ^1^H NMR demonstrated that only ethyl acetate, ethanol and minor amount of ***fac*-1-H_2_** were left in the solution while ruthenium alkoxides were not observed. Crystals were collected, washed in toluene and dried on vacuum giving 19.8 mg (93% yield) of **1_2_-BDM_2_** which structure was resolved with X-ray analysis. The crystals were not soluble in aprotic organic solvents and only dissolving the material in methanol-d4allowed to obtain NMR spectra, although spectra demonstrate ligand exchange between BDM and deuterated methanol.

**1_2_-BDM_2_:** ^1^H NMR (400 MHz, CD_3_OD, 25 °C), *δ*: 7.47 (m, 6H), 7.39 (m, 9H), 7.33 (s, 4H), 4.59 (s, 4H), 3.25 (m, 2H), 3.03 (m, 2H), 2.91 (m, 2H), 2.63 (m, 2H), 2.12 (m, 2H), 1.46 (m, 2H), 0.88 (m, 6H), -23.58 (br.s, in exchange with CD_3_OD). ^13^C{^1^H} NMR (101 MHz, CD_3_OD, 25 °C), *δ*: 141.8, 139.6 (d, J = 38.0 Hz), 134.8 (d, J = 10.9 Hz), 130.3, 129.1, 129.0 (d, J = 9.0 Hz), 128.1, 65.0, 52.8, 37.6, 36.7, 14.2, 13.8. ^31^P{^1^H} NMR (162 MHz, CD_3_OD, 25 °C), *δ*: 65.83.

Figure S118. ^1^H NMR (500 MHz, 25 °C, toluene-d8) of mixture of complex 1-EtOH and EHMB after keeping it at room temperature for 1 h. EtOA – ethyl acetate; EtOH – ethanol.

Figure S119. ^31^P{^1^H} NMR (202 MHz, 25 °C, toluene-d8) of a mixture of complex 1-EtOH and EHMB after keeping it at room temperature for 1 h.

Figure S120. ^13^C{^1^H} DEPTQ NMR (126 MHz, 25 °C, toluene-d8) of a mixture of complex 1-EtOH and EHMB after keeping it at room temperature for 1 h.

Figure S121. ^1^H NMR (500 MHz, 25 °C, toluene-d8) of mother liquor solution after precipitation of 1_2_-BDM_2_ from mixture of complex 1-EtOH and EHMB after keeping it at room temperature for 2 days. EtOAc – ethyl acetate; EtOH – ethanol.

Figure S122. ^1^H NMR (400 MHz, 25 °C, CD_3_OD) of 1_2_-BDM_2_ crystals obtained after reaction of 1-EtOH and EHMB at room temperature for 2 days.

Figure S123. ^31^P{^1^H} NMR (162 MHz, 25 °C, CD_3_OD) of 1_2_-BDM_2_ crystals obtained after reaction of 1-EtOH and EHMB at room temperature for 2 days.

Figure S124. ^13^C{^1^H} DEPTQ NMR (101 MHz, 25 °C, CD_3_OD) of 1_2_-BDM_2_ crystals obtained after reaction of 1-EtOH and EHMB at room temperature for 2 days.

# Kinetic Studies

## General Procedure

**General procedure for kinetic experiments**: PET (400 mg, 2.1 mmol) was added to an autoclave (Figure S125, I) and vacuum cycled with argon 3 times. Under the flow of argon, the desired amount of KO*t*Bu (1M in THF) and solvent (4 mL, EtOH/MeTHF) were added to the autoclave before the desired amount of catalyst (in EtOH/MeTHF) was added to a separate injection port (Figure S125, II, III). The entire system was brought to the desired temperature and H_2_ pressure before the system was sealed, and the catalyst solution was added to the reaction mixture (Figure S125, IV). The change in pressure of the system was then monitored to observe H_2_ consumption as a function of time (Figure S125, V).

Figure S125: Diagram supporting procedure for carrying out kinetic experiments.

## Results from maximised TON experiments:

**General procedure for maximised TON experiments:** PET (192 mg, 1 mmol) and the desired amount of KO*t*Bu were added to a glass vial (fitted with a septum) in a glovebox. The solvent (4:1 MeTHF:EtOH, 2.5 mL) and catalyst solution (13.3 μL, 0.001562 M) were then added to the vial before the vials were sealed and placed inside a sealed autoclave. The autoclave was taken outside the glovebox. The autoclave was then cycled with hydrogen 3 times, before being pressurised to 50 bar H_2_. The autoclave was stirred and heated for 72 h before being allowed to cool and analysed by ^1^H NMR spectroscopy.

| **Table S13. Hydrogenation of PET with 1 for TON maximization.***^[^****^a]^*** | | | | | |
| --- | --- | --- | --- | --- | --- |
| Entry | KO*t*Bu, mol% | Yield of BDM, % | Yield of EHMB, % | Yield of DETP, % | TON |
| 1 | 40 | <1 | 45 | 55 | 22,500 |
| 2 | 60 | <1 | 55 | 45 | 27,500 |
| 3 | 80 | <1 | 74 | 26 | 37,000 |
| *[a]* General procedure for maximised TON experiments. | | | | | |

## ^1^H NMR spectra corresponding to *fac*-1-H_2_ preparation

**General procedure for in situ *fac*-1-H_2_ preparation**: A solution of **1** (5 mg, 0.008 mmol) in THF-d8 (5 mL) was added to a high-pressure NMR tube. A small glass insert filled with a solution of KO*t*Bu (0.01 mL, 1M) was added to the same NMR tube such that it rested above the catalyst solution. It was then pressurized with H_2_ (5 bar) and under this pressure, the tube was gently tipped such that the KO*t*Bu solution was added to the catalyst solution. The activation was followed by NMR over 24 h (Figure S126 and Figure S127), after which there was full convergence of the species present to the ***fac*-1-H_2_** complex by ^1^H and ^31^P{^1^H} NMR.

**a**

**a**

**a**

**b**

**b**

t = 24 h

t = 5 h

t = 1.5 h

t < 1 h

**a**

**b**

Figure S126: Selected region of the ^1^H NMR spectra (d_8_-THF, 400 MHz, 298 K) showing temporal profile of the reaction of complex 1 with KO*t*Bu under H_2_ (5 bar).

**a**

**a**

**b**

**a**

**b**

**b**

t = 24 h

t = 5 h

t = 1.5 h

t < 1 h

**a**

**b**

Figure S127: Selected region of the ^31^P{^1^H} NMR spectra (d_8_-THF, 162 MHz, 298 K) showing temporal profile of the reaction of complex 1 with KO*t*Bu under H_2_ (5 bar).

## Kinetic plots of PET and DET hydrogenation

i) **1**

ii) **1** (0.05 mol%) half of KO^t^Bu added initially

iii) ***fac*-1-H_2_** (0.025 mol%)

iv) ***fac*-1-H_2_** (0.025 mol%)

i) **1**

ii) **1** (0.05 mol%) half of KO^t^Bu added initially

iii) ***fac*-1-H_2_** (0.025 mol%)

iv) ***fac*-1-H_2_** (0.025 mol%)

Figure S128. Hydrogenation yield vs time, and hydrogenation rate vs hydrogenation yield graphs describing varying activation methods and pressure.

Figure S129. Hydrogenation yield vs time, and hydrogenation rate vs hydrogenation yield graphs describing varying temperatures.

**Figure S130**: Hydrogenation yield vs time, and hydrogenation rate vs hydrogenation yield graphs describing varying base and base loading.

**Figure S131**: Hydrogenation yield vs time, and hydrogenation rate vs hydrogenation yield graphs describing varying base and base loading.

**Figure S132**: Hydrogenation yield vs time, and hydrogenation rate vs hydrogenation yield graphs describing varying base and base loading.

**Figure S133:** Hydrogenation yield vs time, and hydrogenation rate vs hydrogenation yield graphs describing varying base and base loading.

## ^1^H NMR spectra corresponding to kinetic plots of PET and DET hydrogenation

**Figure S134.** ^1^H NMR (400 MHz, CDCl_3_, 298 K) spectrum for the hydrogenation of PET (2 mmol) under conditions: **1** (0.05 mol%), KO*t*Bu (10 mol%), 4:1 MeTHF:EtOH (5 mL), 80 °C, corresponding to *Figure S1*28, i.

**Figure S135*.*** ^1^H NMR (400 MHz, CDCl_3_, 298 K) spectrum for the hydrogenation of PET (2 mmol) under conditions: **1** (0.05 mol%), KO*t*Bu (10 mol%), 4:1 MeTHF:EtOH (5 mL), 80 °C, corresponding to *Figure S1*28, ii.

**Figure S136*.*** ^1^H NMR (400 MHz, CDCl_3_, 298 K) spectrum for the hydrogenation of PET (2 mmol) under conditions: **1** (0.025 mol%), KO*t*Bu (10 mol%), 4:1 MeTHF:EtOH (5 mL), 80 °C, corresponding to *Figure S1*28, iii.

**Figure S137*.*** ^1^H NMR (400 MHz, CDCl_3_, 298 K) spectrum for the hydrogenation of PET (2 mmol) under conditions: **1** (0.05 mol%), KO*t*Bu (10 mol%), 4:1 MeTHF:EtOH (5 mL), 80 °C, corresponding to *Figure S1*28, iv.

**Figure S138*.*** ^1^H NMR (400 MHz, CDCl_3_, 298 K) spectrum for the hydrogenation of PET (2 mmol) under conditions: **1** (0.05 mol%), KO*t*Bu (10 mol%), 4:1 MeTHF:EtOH (5 mL), 70 °C, corresponding to *Figure S*129, i.

**Figure S139*.*** ^1^H NMR (400 MHz, CDCl_3_, 298 K) spectrum for the hydrogenation of PET (2 mmol) under conditions: **1** (0.05 mol%), KO*t*Bu (10 mol%), 4:1 MeTHF:EtOH (5 mL), 90 °C, corresponding to *Figure S*129, ii.

**Figure S140*.*** : ^1^H NMR (400 MHz, CDCl_3_, 298 K) spectrum for the hydrogenation of PET (2 mmol) under conditions: **1** (0.05 mol%), KO*t*Bu (10 mol%), 4:1 MeTHF:EtOH (5 mL), 110 °C, corresponding to *Figure S*129, iii.

**Figure S141*.*** ^1^H NMR (400 MHz, DMSO-d6, 298 K) spectrum for the hydrogenation of PET (2 mmol) under conditions: **1** (0.025 mol%), KO*t*Bu (10 mol%), 4:1 MeTHF:EtOH (5 mL), 80 °C, corresponding to Figure S131, i.

**Figure S142*.*** ^1^H NMR (400 MHz, DMSO-d6, 298 K) spectrum for the hydrogenation of PET (2 mmol) under conditions: **1** (0.025 mol%), KO*t*Bu (10 mol%), 4:1 MeTHF:*t*BuOH (5 mL), 80 °C, corresponding to Figure S131, ii.

**Figure S143*.*** ^1^H NMR (400 MHz, DMSO-d6, 298 K) spectrum for the hydrogenation of PET (2 mmol) under conditions: **1** (0.025 mol%), KO*t*Bu (10 mol%), 4:1 MeTHF:*i*PrOH (5 mL), 80 °C, corresponding to Figure S131, iii.

**Figure S144*.*** ^1^H NMR (400 MHz, DMSO-d6, 298 K) spectrum for the hydrogenation of PET (2 mmol) under conditions: **1** (0.025 mol%), KO*t*Bu (10 mol%), 4:1 MeTHF:MeOH (5 mL), 80 °C, corresponding to Figure S131, iv.

**Figure S145*.*** ^1^H NMR (400 MHz, CDCl_3_, 298 K) spectrum for the hydrogenation of DETP (2 mmol) under conditions: **1** (0.025 mol%), KO*t*Bu (10 mol%), 4:1 MeTHF:EtOH (5 mL), 80 °C, corresponding to Figure S132, i.

**Figure S146*.*** ^1^H NMR (400 MHz, CDCl_3_, 298 K) spectrum for the hydrogenation of DETP (2 mmol) in the presence of ethylene glycol (30 mol%) under conditions: **1** (0.025 mol%), KO*t*Bu (10 mol%), 4:1 MeTHF:EtOH (5 mL), 80 °C, corresponding to Figure S132, ii.

**Figure S147**.^1^H NMR (400 MHz, CDCl_3_, 298 K) spectrum for the hydrogenation of DETP (2 mmol) in the presence of 1,4-benzenedimethanol (30 mol%) under conditions: **1** (0.025 mol%), KO*t*Bu (10 mol%), 4:1 MeTHF:EtOH (5 mL), 80 °C, corresponding to Figure S132, iii.

**Figure S148**: ^1^H NMR (400 MHz, CDCl_3_, 298 K) spectrum for the hydrogenation of DETP (2 mmol) under conditions: **1** (0.025 mol%), KO*t*Bu (10 mol%), 9:1 MeTHF:MeOH (5 mL), 80 °C, corresponding to Figure S133, ii.

## ^1^H NMR spectra corresponding to maximising TON experiments

Figure S149: ^1^H NMR (400 MHz, DMSO-d6, 298 K) spectrum for the hydrogenation of PET (1 mmol) under conditions: 1 (0.002 mol%), KO*t*Bu (40 mol%), 4:1 MeTHF:MeOH (2.5 mL), 80 °C, 50 bar H_2_ corresponding to Table S13, entry 1.

Figure S150: ^1^H NMR (400 MHz, DMSO-d6, 298 K) spectrum for the hydrogenation of PET (1 mmol) under conditions: 1 (0.002 mol%), KO*t*Bu (60 mol%), 4:1 MeTHF:MeOH (2.5 mL), 80 °C, 50 bar H_2_ corresponding to Table S13, entry 2.

Figure S151: ^1^H NMR (400 MHz, DMSO-d6, 298 K) spectrum for the hydrogenation of PET (1 mmol) under conditions: 1 (0.002 mol%), KO*t*Bu (80 mol%), 4:1 MeTHF:MeOH (2.5 mL), 80 °C, 50 bar H_2_ corresponding to Table S13, entry 3.

# ^1^H NMR CEST

## General Procedure

**General procedure for CEST NMR experiments**: The ***fac-1-H_2_*** complex was prepared as described above (Section 5.1.). To this solution, the desired equivalents of varying additives were added to the NMR tube in a glovebox, before the NMR tube was repressurized with H_2_ (5 bar), and the corresponding CEST measurements were taken.

## ^1^H NMR CEST spectra

Chemical exchange saturation transfer experiment (CEST)^5^ is a magnetization transfer experiment, where a narrow-band low-energy saturation pulse at frequency *v* is applied prior to broad-band hard pulse NMR detection. By varying the saturation frequency *v*, the CEST spectrum is recorded.^5^ In our study, we use a continuous wave (CW) 50 Hz saturation pulse applied for 2–3 sec., whose effects are detected by virtue of the reduction of the easily detectable signal of free dissolved H_2_, which is reduced by the saturation of exchanging hydrides bound to one of the organometallic complexes.

## ^1^H NMR spectra of *fac*-1-H_2_ with ethylene glycol used in CEST

Figure S152: ^1^H NMR spectrum (d_8_-THF, 400 MHz, 298 K) of *fac*-1-H_2_ (0.008 mmol) solution described in Section 5.1., with ethylene glycol (0.08 mmol).

Figure S153. A) ^1^H NMR CEST spectra recorded after adding ethylene glycol (5–40 equivalents relative to 1) to the reaction mixture obtained by mixing complex 1 (5 mg) with KO*t*Bu (5 eq.) and 5 bar H_2_ in THF-d_8_. B) ^1^H NMR CEST spectra showing reappearance of signals around -15 and -20 ppm (possible active species) after the addition of base (1 (5 mg), d_8_- THF (0.5 mL), 5 bar H_2_).

# Multi-gram scale hydrogenation.

To confirm the applicability of the developed method for larger-scale hydrogenation, we also performed several hydrogenations of PET on a 5–10 g scale (Table S14). Excellent yield of BDM was obtained when 5 g of post-consumer PET-b was hydrogenated; 60 bar was applied at the start of the reaction, and 23 bar was consumed (Entry 3, Table S14). When 10 g of plastic was used with 50 bar hydrogen pressure, the reaction would have to consume almost all the hydrogen applied (50 bar). During the first 3 h it consumed 10 bar, and around 28 bar was consumed overnight. The reaction was cooled down, and the autoclave was pressurized up to 50 bar again, but the next night, only 5 bar were consumed.

Figure S154. Illustration of bench scale hydrogenative depolymerization of PET-b derived from post-consumer plastic bottle. A. The virgin plastic bottle. B. PET chips made from bottle. C. PET chips in reaction vessel. D. Reaction mixture before hydrogenation. E. The autoclave used for hydrogenation. F. Reaction mixture after hydrogenation.

**General procedure O.**

Post-consumer polyethylene terephthalate (8–52 mmol, 1 eq.), was weighed in a glovebox, placed into a glass vessel for autoclave with a stir bar. 2-MeTHF (45–46.8 mL) and EtOH (1.5–5.2 mL) were added via syringe. Next, KO*t*Bu or KOEt (5 mol%) was added. The mixture was stirred at room temperature for 5 min. Next, the specified amount of complex **1** (0.001–0.05 mol%) was added as freshly prepared 0.002 M solution in 2-MeTHF. The autoclave was sealed, purged with H_2_ (20 bar), pressurized with H_2_ (50 or 60 bar), and placed in an oil bath preheated to 80 °C. The reaction was stirred at 80 °C for 42–72 h. After that, the autoclave was cooled down to room temperature in air and then an ice bath and carefully vented to atmosphere. The crude yield of the product was estimated by NMR spectroscopy using mesitylene or dichloromethane as an internal standard.

**Isolation procedure for Entry 3, Table S14.** Solvents were evaporated on a rotary evaporator. The residue was recrystallized from EtOH/Et_2_O=10/1 mixture, giving 1.94 g of 1,4-benzenedimethanol. The solvents from the mother liquor were evaporated and the residue was subjected to Kugelrohr distillation at 120 °C and 1 mbar to collect ethylene glycol. As a result, 1.354 g (84% yield) of ethylene glycol were collected. The residue after distillation was combined with the isolated earlier precipitate and the combined solid was washed with diethyl ether giving 3.01 g (84% yield) of 1,4-benzenedimethanol.

**Isolation procedure for Entry 4, Table S14.** Solvents were evaporated on a rotary evaporator. The residue was subjected to Kugelrohr distillation at 120 °C and 1 mbar to collect ethylene glycol. As a result, 1.524 g (94% yield) of ethylene glycol were collected. The solid residue was refluxed in 1 M solution of KOH (100 mL) for 1 h and then extracted with isobutyl alcohol (3x50 mL). The combined organic fractions were dried over anhydrous Na_2_SO_4_ and the solvent was evaporated. The residue was dried under vacuum giving 3.03 g (84% yield) of 1,4-benzenedimethanol.

**Isolation procedure for Entry 5, Table S14.** Solvents were evaporated on a rotary evaporator. The residue was subjected to Kugelrohr distillation at 120 °C and 1 mbar to collect ethylene glycol. As a result, 1.085 g (67% yield) of ethylene glycol was collected. The solid residue was refluxed in a 1 M solution of KOH (100 mL) for 2 h and then extracted with isobutyl alcohol (3x10 mL). The water layer was acidified to pH 1 with 1 M hydrochloric acid, and the resulting mixture was extracted with isobutyl alcohol (3x50 mL). The combined organic fractions were dried over anhydrous Na_2_SO_4,_ and the solvent was evaporated. The residue was dried under vacuum, giving 2.97 g of 4-(hydroxymethyl)benzoic acid of 96 wt% purity (72% yield).

| **Table S14. Hydrogenation of PET with 1 on multi-gram scale in an autoclave.***^[a]^* | | | | | | | | | | | |
| --- | --- | --- | --- | --- | --- | --- | --- | --- | --- | --- | --- |
| Entry | Start mat., mmol | 2-MeTHF, mL | EtOH, mL | Base | Base, mol% | cat. **1,** mol% | P (H_2_), bar (eq.) | Time, h | Yield  BDM, % | Yield  EHMB, % | Isolated Yield EG, % |
| 1 | 10 | 45 | 5 | KO*t*Bu | 5 | 0.05 | 50 (≈22 eq.) | 66 | 52 | 41 | - |
| 2 | 52 | 46.8 | 5.2 | KO*t*Bu | 5 | 0.05 | 50 (≈4 eq.) | 42 | 53 | 45 | - |
| 3 | 26 | 46.8 | 5.2 | KO*t*Bu | 5 | 0.05 | 60 (≈10 eq.) | 72 | 95 (84^[b]^) | 5 | 84 |
| 4 | 26 | 46.8 | 5.2 | KOEt | 5 | 0.05 | 60 (≈10 eq.) | 72 | 93 (84^[b]^) | 7 | 94 |
| 5 | 26 | 46.8 | 5.2 | KOEt | 5 | 0.005 | 60 (≈10 eq.) | 72 | 8 | 84 (72^[c]^) | 67 |
| 6 | 26^[d]^ | 46.8 | 5.2 | KO*t*Bu | 5 | 0.005 | 60 (≈10 eq.) | 48 | 12 | 80 | - |
| 7 | 8.2^[e]^ | 45 | 1.5 | KO*t*Bu | 5 | 0.005 | 60 (≈32 eq.) | 48 | 8 | 83 | - |
| 8 | 26^[f]^ | 45 | 5 | KO*t*Bu | 5 | 0.005 | 60 (≈10 eq.) | 48 | 18 | 82 | - |
| 9 | 8.2^[g]^ | 45 | 5 | KO*t*Bu | 10 | 0.01 | 60 (≈32 eq.) | 48 | 7 | 86 | - |
| 10 | 26 | 30 | 3.3 | KO*t*Bu | 25 | 0.002 | 60 (≈12 eq.) | 72 | 2 | 60 | - |
| 11 | 20 | 45 | 5 | NaOEt | 20 | 0.001 | 70 (≈15 eq.) | 42 | <1 | 14 | - |
| *[a]* General procedure O. *[b]* Isolated yield of 1,4-benzenedimethanol. *[c]* Isolated yield of 4-(hydroxymethyl)benzoic acid. *[d]* Coloured plastic was used. *[e]* Cloth from hairband was used*. [f]* Green ribbon was used. *[g]* Blue fleece jacket was used. | | | | | | | | | | | |

Figure S155. ^1^H NMR (400 MHz, methanol-d4) spectrum of reaction mixture from Entry 1, Table S14.

Figure S156. Fragment of ^1^H NMR (400 MHz, methanol-d4) spectrum of reaction mixture from Entry 1, Table S14.

Figure S157. ^1^H NMR (400 MHz, methanol-d4) spectrum of reaction mixture from Entry 2, Table S14.

Figure S158. Fragment of ^1^H NMR (400 MHz, methanol-d4) spectrum of reaction mixture from Entry 2, Table S14.

Figure S159. ^1^H NMR (400 MHz, methanol-d4) spectrum of reaction mixture from Entry 3, Table S14.

Figure S160. Fragment of ^1^H NMR (400 MHz, methanol-d4) spectrum of reaction mixture from Entry 3, Table S14.

Figure S161. ^1^H NMR (400 MHz, methanol-d4) spectrum of ethylene glycol isolated from reaction in Entry 3, Table S14.

Figure S162. ^1^H NMR (400 MHz, methanol-d4) spectrum of 1,4-benzenedimethanol isolated from reaction in Entry 3, Table S14.

Figure S163. Fragment of ^1^H NMR (400 MHz, methanol-d4) spectrum of reaction mixture from Entry 4, Table S14.

Figure S164. ^1^H NMR (400 MHz, methanol-d4) spectrum of ethylene glycol isolated from reaction in Entry 4, Table S14.

Figure S165. ^1^H NMR (400 MHz, methanol-d4) spectrum of 1,4-benzenedimethanol isolated from reaction in Entry 4, Table S14. MesH – mesitylene used as internal standard for purity estimation.

Figure S166. ^1^H NMR (400 MHz, methanol-d4) spectrum of reaction mixture from Entry 5, Table S14.

Figure S167. Fragment of ^1^H NMR (400 MHz, methanol-d4) spectrum of reaction mixture from Entry 5, Table S14.

Figure S168. ^1^H NMR (400 MHz, methanol-d4) spectrum of ethylene glycol isolated from reaction in Entry 5, Table S14.

Figure S169. ^1^H NMR (400 MHz, methanol-d4) spectrum of 4-(hydroxymethyl)benzoic acid isolated from reaction in Entry 5, Table S14. MesH – mesitylene used as internal standard for purity estimation.

Figure S170. Fragment of ^1^H NMR (400 MHz, methanol-d4) spectrum of reaction mixture from Entry 6, Table S14.

Figure S171. Fragment of ^1^H NMR (400 MHz, methanol-d4) spectrum of reaction mixture from Entry 7, Table S14.

Figure S172. Fragment of ^1^H NMR (400 MHz, methanol-d4) spectrum of reaction mixture from Entry 8 Table S14.

Figure S173. Fragment of ^1^H NMR (400 MHz, methanol-d4) spectrum of reaction mixture from Entry 9, Table S14.

Figure S174. Fragment of ^1^H NMR (400 MHz, methanol-d4) spectrum of reaction mixture from Entry 10, Table S14.

Figure S175. Fragment of ^1^H NMR (400 MHz, methanol-d4) spectrum of reaction mixture from Entry 11, Table S14.

# Ethyl 4-(hydroxymethyl)benzoate as a feedstock.

## 7.1 Synthesis of small organic molecules from ethyl 4-(hydroxymethyl)benzoate.

### 7.1.1 Preparation of 4-(chloromethyl)benzoyl chloride 8^7^

4-(Hydroxymethyl)benzoic acid (1.0 g, 6.6 mmol) was dissolved in dichloromethane (15 mL), followed by a drop of dimethyl formamide (approx. 10 mg) and thionyl chloride (5 mL, 25.6 mmol). After gases stopped evolving, the mixture was stirred for another hour, and then all volatiles were removed under vacuum and collected in a cold trap prior to disposal. The product was isolated as a yellow solid (6.2 g, 100%). ^1^H NMR (400 MHz, CDCl_3_, 298 K), *δ*: 8.12 (d, J = 8.2 Hz, 2H), 7.54 (d, J = 8.2 Hz, 2H), 4.63 (s, 2H).

### 7.1.2 Preparation of ethyl 4-formylbenzoate 9^8^

To a solution of ethyl 4-(hydroxymethyl)benzoate (500 mg, 2.8 mmol) in dry acetonitrile (5 mL) in a 20 mL round-bottom flask were added CuBr (40 mg, 0.28 mmol), 2,2’-bipyridyl (44 mg, 0.28 mmol), TEMPO (44 mg, 0.28 mmol) and N-methylimidazole (23 mg, 0.28 mmol). The dark red-brown reaction mixture was stirred rapidly open to air. It was stopped when its colour changed to green. Next, the reaction mixture was diluted with 1:1 ether:pentane (20 mL) and filtered through a plug of silica with rinsing. The volatiles were evaporated, and the residue was dried under vacuum giving the product as a yellow oil (460 mg, 93%). ^1^H NMR (400 MHz, CDCl_3_, 298 K), *δ*: 10.13 (s, 1H), 8.23 (d, J = 8.0 Hz, 2H), 7.97 (d, J = 8.0 Hz, 2H), 4.44 (q, J = 7.1 Hz, 2H), 1.45 (t, J = 7.1 Hz, 3H). ^13^C NMR (101 MHz, CDCl_3_, 298 K), *δ*:170.0, 138.5, 134.9, 129.6, 128.9, 77.0, 61.0, 13.7.

### 7.1.3 Preparation of ethyl (4-bromomethyl)benzoate 10^9^

To a solution of ethyl 4-(hydroxymethyl)benzoate (517 mg, 2.9 mmol) in dry dichloromethane (10 mL) in a 25 mL Schlenk tube under Argon, phosphorous (III) bromide (931 mg, 3.4 mmol) was added dropwise at 0 °C. The resulting solution was stirred at room temperature overnight. Next, the reaction was quenched with ice and saturated sodium bicarbonate solution (20 mL) followed by extraction with dichloromethane (3x10 mL). Organic fractions were combined, dried over anhydrous sodium sulphate. The solvent was evaporated and the residue was dried under vacuum giving the product as a white solid (598 mg, 86%). ^1^H NMR (400 MHz, CDCl_3_, 298 K), *δ*: 8.01 (d, J = 8.2 Hz, 2H), 7.45 (d, J = 8.2 Hz, 2H), 4.49 (s, 2H), 4.37 (t, J = 7.1 Hz, 2H), 1.39 (t, J = 7.1 Hz, 3H). ^13^C NMR (101 MHz, CDCl_3_, 298 K), *δ*: 166.0, 142.5, 130.4, 130.0, 128.9, 61.0, 32.2, 14.3.

### 7.1.4 Preparation of ethyl 4-(aminomethyl)benzoate 11^10^

In a high pressure Schlenk tube with Young’s cap ethyl 4-(hydoxymethyl)benzoate (180 mg, 1 mmol) was loaded followed by 1 mol% of Ru(CO)ClH(PPh_3_)_3_ (9.5 mg, 0.01 mmol) and 1.5 mol% of Xantphos (8.7 mg, 0.015 mmol) and anhydrous *tert*-amyl alcohol (1.5 mL) under argon atmosphere. Next, the Young’s tube was closed and cooled in dry ice so that ammonia (0.6 g) could be condensed into it, and then the reaction mixture was stirred at 150 °C for 20 hrs. The solution was allowed to cool down to room temperature, leading to the formation of a precipitate. The solution was separated by decantation and volatiles were removed under vacuum to yield a white solid (130.5 mg, 79% yield). ^1^H NMR (500 MHz, CDCl_3_, 298 K), *δ*: 7.93 (d, J = 8.2 Hz, 2H), 7.31 (d, J = 8.2 Hz, 2H), 4.31 (q, J = 7.1 Hz, 2H), 3.86 (s, 2H), 1.61 (br.s, 2H), 1.35 (t, J = 7.1 Hz, 3H). ^13^C NMR (126 MHz, CDCl_3_, 298 K), *δ*: 166.6, 146.2, 129.8, 129.2, 127.1, 60.9, 45.2, 14.1.

## 7.2 Synthesis and depolymerization of polyester from 4-(hydroxymethyl)benzoic acid

4-(Hydroxymethyl)benzoic acid (HMBA) (1.0 g, 6.6 mmol) and aluminium isopropoxide (3 mg, 0.25 mol%) were added to a 25 mL round-bottom flask, which was equipped with an adapter connected to a Schlenk line and refilled with Argon. The first step was esterification at 260 °C for 1 h under an Argon atmosphere. On the second step vacuum was applied and the reaction mixture was stirred at 260 °C under 1 mbar of pressure for 10 min. Next, the flask was refilled with Argon and the heating was continued for an additional hour. After the polycondensation reaction was completed, the reaction mixture was cooled down to room temperature and removed from the flask as 651 mg (74%) of yellow glass, which was separated from the sublimated white powder of unreacted starting material. The degree of polymerization estimated by the end group analysis using ^1^H NMR spectroscopy was found to be 14, which gives molecular weight M_n_ = 1,894.6 g/mol.

DSC: T_g_ = 49.3 °C; T_m_ = 245.2 °C; T_c_ = 164.8 °C. TGA: T_d_(5 wt%) = 387 °C.

^1^H NMR (500 MHz, CF_3_COOD, 25 °C, relative to D_2_O), *δ*: 8.59 (m, 2H), 8.58 (m, 26H), 8.02 (m, 26H), 5.96 (s, 26H), 5.30 (s, 2H). ^13^C NMR (125 MHz, CF_3_COOD, 25 °C, relative to D_2_O), *δ*: 171.9, 143.9, 132.3, 130.8, 129.7, 69.3.

FT-IR (ATR): 2941w (*ν*_CH2_), 1711s (*ν*_C=O_), 1258s (*ν*_C-O_), 1088s (*ν*_C-O_), 748s, (*ν*_CH_).

The obtained above PHMB (210 mg, 1.57 mmol) was mixed with KOH (352 mg, 6.3 mmol) in water (2 mL) and ethanol (2 mL). The resulting suspension was refluxed for 2 hours giving yellowish solution. The mixture was acidified with HCl_(conc)_ (2 mL) and cooled down to room temperature. The formed precipitate was filtered, washed with water and dried on vacuum giving HMBA as a white solid (213 mg, 89% yield).

^1^H NMR (500 MHz, CF_3_COOD, relative to D_2_O, 25 °C; HMBA polymerizes in TFAA giving complex NMR, only major signals are given), *δ*: 8.52 (d, J = 7.9 Hz, 2H), 7.90 (d, J = 7.9 Hz, 2H), 5.32 (s, 2H). ^13^C NMR (125 MHz, CF_3_COOD, 25 °C), *δ*: 175.3, 147.3, 132.8, 129.8, 129.3, 66.6.

Figure S176. ^1^H NMR (500 MHz, CF_3_COOD, relative to D_2_O) of poly(4-(hydroxymethyl)benzoate) (PHMB) at room temperature.

Figure S177. ^13^C NMR (125 MHz, CF_3_COOD) of poly(4-(hydroxymethyl)benzoate) (PHMB) at room temperature.

Figure S178. FT-IR (ATR) spectrum of polyethylene poly(4-(hydroxymethyl)benzoate) (PHMB).

Figure S179. DSC traces of the polyester PHMB.

Figure S180. Mass loss as a function of temperature for polyamide poly(4-(hydroxymethyl)benzoate) (PHMB). T_d_(5 wt%) = 387 °C.

Figure S181. ^1^H NMR (500 MHz, CF_3_COOD, relative to D_2_O) of 4-(hydroxymethyl)benzoic acid (HMBA) at room temperature.

Figure S182. ^13^C NMR (125 MHz, CF_3_COOD) of 4-(hydroxymethyl)benzoic acid (HMBA) at room temperature.

Figure S183. ^1^H NMR (500 MHz, CF_3_COOD, relative to D_2_O) of virgin PET powder at room temperature. Estimated degree of polymerization = 14, M_n_ = 2,708.7 g/mol.

Figure S184. ^13^C NMR (125 MHz, CF_3_COOD) of virgin PET powder at room temperature.

Figure S185. FT-IR (ATR) spectrum of 4-(hydroxymethyl)benzoic acid (HMBA).

Figure S186. FT-IR (ATR) spectrum of polyethylene terephthalate powder (PET-p).

Figure S187. DSC traces of the virgin PET powder.

Figure S188. Mass loss as a function of temperature for polyamide 4-(hydroxymethyl)benzoic acid (HMBP). T_d_(5 wt%) = 226 °C.

Figure S189. Mass loss as a function of temperature for polyamide polyethylene terephthalate powder (PET-p). T_d_(5 wt%) = 404 °C.

## 7.3 Mechanical properties of PHMB and PET

Polyethylene terephthalate (PET) and poly(4-(hydroxymethyl)benzoate) (PHMB) polymer films were prepared using a Specac constant pressure film maker. Polymer powder was carefully placed between the aligned top and bottom plates of the mould. The mould was then transferred to the heated platen of the film maker, set to 260 °C for PET and 220 °C for PHMB.

A constant pressure of 2000 N was applied for a minimum of 20 minutes to form smooth, uniform films. After cooling to room temperature, the films were removed and cut into rectangular specimens measuring 10 mm in width and 50 mm in gauge length, with a thickness of slightly less than 1 mm.

Mechanical properties—including tensile strength, elastic modulus, and elongation at break—were determined using an Instron 3342 tensile testing machine, with a crosshead speed of 50 mm/min to establish the initial strain rate.

The PET film exhibited an ultimate tensile strength of 42 MPa, elastic modulus of 1.8 GPa, and strain at break of 12%, aligning well with reported values for semi-crystalline PET (typically 40–50 MPa tensile strength, 1.5–2.5 GPa modulus, and 10–15% elongation)^11^. In contrast, PHMB showed a lower tensile strength of 24 MPa, elastic modulus of 0.95 GPa, and strain at break of 10.5%. These results indicate that PHMB is softer, less stiff, and less strong than PET, reflecting its more amorphous nature and reduced intermolecular bonding. The comparative drop^12^ in modulus and strength suggests PHMB may offer more flexibility, but at the expense of mechanical robustness.

Differential scanning calorimetry (DSC) data (Figures S179 and S187) indicate that PET exhibits a higher thermal transition, consistent with its semi-crystalline nature and mechanical robustness. PHMB, however, shows a broader and lower transition, suggesting an amorphous or less crystalline character, which contributes to its lower modulus and tensile strength.

Thermogravimetric analysis (TGA) (Figures S180 and S189) reveals that PHMB starts degrading at a lower temperature compared to PET, indicating reduced thermal stability. The mass loss profiles highlight that PET maintains its structural integrity up to higher temperatures, consistent with its established performance in demanding thermal environments.

Overall, the synthesis and characterisation of PHMB confirm it as a viable polyester with tuneable properties. However, compared to PET, it exhibits lower mechanical and thermal performance, which may limit its applications in load-bearing or high-temperature environments. Nonetheless, its lower processing temperature (220 °C vs. 260 °C) and amorphous nature may offer advantages in biomedical or flexible device applications where mild processing conditions and ductility are desired.

Figure S190. Stress-strain curve of PHMB and PET.

# Life cycle assessment of the production of EHMB.

To assess the environmental impact, we conducted a preliminary life cycle assessment^13^ of our method and compared it with a conventional method of EHMB production that involves bromination of *p*-toluic acid with N-bromosuccinimide, followed by hydrolysis of the 4-(bromomethyl)benzoic acid to HMBA and its esterification catalysed by sulfuric acid (Scheme S3).^14^

The experimental procedures for synthesis of EHMB from p-Toluic acid were taken from the literature.^14^ The procedures used for calculations are provided below.

Scheme S3. Synthesis of EHMB with conventional method.

**Preparation of 4-(Bromomethyl)benzoic acid (13).** Raw materials: *p*-Toluic acid **12**: 1.8 Kgs; *N*-bromosuccinimide: 2.36 Kgs; Benzoyl peroxide: 0.03 Kgs. Chloroform: 25.71 Kgs. Ethyl acetate: 15.57 Kgs; Water: 16.24 Kgs. 25.7 Kgs of chloroform was charged into the reactor. Next, 1.8 Kgs of **12** was charged to the reactor. Next, 2.36 Kgs of N-bromosuccinimide was charged. Next, 0.03 Kgs of benzoyl peroxide was charged. Reaction mass was heated to reflux temperature and maintained reflux for 2.5–3 hours. Cooled down to 25–35 °C. Crude 4-(bromomethyl)benzoic acid was filtered and washed with water thoroughly. Ethyl acetate was charged to the reactor, and the organic layer was washed with water thoroughly. Ethyl acetate was distilled off under vacuum to a small volume and cooled to 25–35 °C. 4-(Bromomethyl)benzoic acid was filtered and washed with ethyl acetate. It is dried at 30–40 °C. Dry weight: 1.7 Kg (60%).

**Preparation of (4-hydroxy methyl)benzoic acid (HMBA).** Raw materials: 4-(Bromomethyl)benzoic acid **13**: 1.7 Kgs; conc. HCl: 2.6 Kgs. 17.1 L of water was charged into the reactor. Next, 1.7 Kgs of 4-(bromomethyl)benzoic acid **13** was charged. Next, 2.6 Kgs of conc. HCl was charged. Reaction was heated to reflux for 10–12 hours. It was cooled down to 25–35 °C. HMBA was filtered and washed with water. The product was dried in oven at 70-80 °C. Yield: 0.9 g (75%).

**Preparation of ethyl 4-(hydroxymethyl)benzoate (EHMB).** Raw materials: HMBA: 0.9 Kgs; Sulfuric acid (conc.): 0.2 Kgs; Ethanol: 22.3 Kgs; Water: 18.6 Kgs; Sodium bicarbonate: 1.17 Kgs; Diethyl ether: 12.7 Kgs. 0.85 of HMBA was charged to the reactor along with 8 Ls of water and 0.2 Kgs of sulfuric acid. The reaction mixture was heated at reflux for 3 h. Next, the reactor was cooled down to 25–35 °C and ethanol was distilled off under vacuum to a small volume and cooled to 25–35 °C. 12.7 Kgs of Diethyl ether was charged to the reactor along with 10.6 Ls water and 1.17 of sodium bicarbonate. The water layer is separated and diethyl ether was distilled off. The residue is dried on vacuum at 40 °C. Dry weight: 1.0 Kg (95%).

Scheme S4. Synthesis of EHMB with our method.

Preparation of **ethyl 4-(hydroxymethyl)benzoate (EHMB) from PET waste (virtual experiment based on results from Table S14).** Raw materials: PET: 1.58 Kgs; 2-methyltetrahydrofuran: 12.5 Kgs; Ethanol: 1.33 Kgs; Water: 31.6 Kgs; Ammonium chloride: 1.58 Kgs; Sodium bicarbonate: 1.58 Kgs; Potassium ethoxide: 34 g; Complex **1**: 2.6 g. 1.58 Kgs g of post-consumer polyethylene terephthalate, was charged to the reactor. 12.5 Kgs of 2-MeTHF and 1.33 Kgs of EtOH were added to the reactor. Next, 34 g of KOEt was added. The mixture was stirred at room temperature for 5 min. Next, 2.6 g of complex **1** was charged to the reactor. The autoclave was sealed, purged with H_2_, pressurized with H_2_ (60 bar, 0.171 Kgs), and placed in an oil bath preheated to 80 °C. The reaction was stirred at 80 °C for 48 h. After that, the autoclave was cooled down to room temperature in air and then an ice bath and carefully vented to atmosphere. Next, reactor was charged with solution of 1.58 Kgs of ammonium chloride in 15.8 L of water. Water layer was separated and the reactor was charged with 1.58 Kgs of sodium bicarbonate solution in 15.8 L of water. Water layer was separated and all organic solvents were distilled off. The residue was dried on vacuum. Dry weight: 1.0 Kg (68%).

The Life Cycle Assessment (LCA) in this study refers to a cradle-to-gate footprint evaluation, focusing on four environmental footprint impact categories: Global Warming Potential (GWP), Acidification, Eutrophication, and Water consumption. These impact categories are the key metrics provided by the ACS Green Chemistry Institute’s streamlined PMI-LCA tool^13,15^ to assess and compare environmental footprints for complex organic reactions. The goal and scope of the calculations is to compare the two synthesis routes for the production of EHMB and to identify hot spots. The streamlined PMI-LCA tool only considers the footprints related to the (purchased) raw materials. Since this tool does not provide comprehensive data for all the solvents and metals, we used data provided for THF to calculate impact of 2-MeTHF, data for Li instead of K, data for Rh instead of Ru. According to information provided by International Platinum Group Metals Association,^16^ contributions of Rh and Ru should be very similar considering the fact amount of Ru in our process is extremely low (S/C=20,000). In addition, emissions related to the incineration of solvents were calculated separately based on available data provided from the pharmaceutical industry to complement the results from the PMI-LCA tool.^17^ Environmental burdens associated with energy consumption of the modelled chemical processes are not considered, but often only play a minor role compared to the resource-intensive raw material and waste-related footprints. Overall, this gives a holistic understanding to compare the chemical processes.

The Life Cycle Assessment revealed that the use of PET-waste as a feedstock for EHMB production offers a markedly reduced environmental footprint compared to the conventional route from *p*-toluic acid (Table S15).

| **Table S15. Processes metrics overview.*^[a]^*** | | | | | | |
| --- | --- | --- | --- | --- | --- | --- |
| Synthesis of EHMB from *p-*toluic acid. | | | | | | |
| Entry | Process Metrics per kg EHMB | Total | Reagent | Metal | Solvent | Water |
| 1 | PMI | 136.38 | 6.55 | 0.00 | 76.28 | 53.55 |
| 2 | GWP (kg CO_2_ equiv.) | 373.4 | 14.28 | 0.00 | 183.60 (+175.5)*^b^* | 0.05 |
| 3 | Acidification (kg SO_2_ equiv.) | 0.53 | 0.08 | 0.00 | 0.45 | 0.00 |
| 4 | Eutrophication (kg posphate equiv.) | 0.27 | 0.04 | 0.00 | 0.23 | 0.00 |
| 5 | Water (kg) | 788.67 | 63.26 | 0.00 | 657.75 | 67.65 |
| Synthesis of EHMB from PET waste. | | | | | | |
| Entry | Process Metrics per kg EHMB | Total | Reagent | Metal | Solvent | Water |
| 6 | PMI | 50.41 | 4.91 | 0.02 | 13.84 | 31.65 |
| 7 | GWP (kg CO_2_ equiv.) | 114.3 | 8.78 | 0.47 | 73.17 (+31.7)*^b^* | 0.03 |
| 8 | Acidification (kg SO_2_ equiv.) | 0.37 | 0.06 | 0.07 | 0.24 | 0.00 |
| 9 | Eutrophication (kg posphate equiv.) | 0.11 | 0.02 | 0.00 | 0.09 | 0.00 |
| 10 | Water (kg) | 328.02 | 25.52 | 6.83 | 255.67 | 39.98 |
| *[a]* The calculations are done using the MS Excel Tool provided by ACS.^13,15^ *[b]* Final GWP of solvents contains contribution from waste solvent incineration which is shown in brackets. It is based on available data provided from the pharmaceutical industry.^17^ | | | | | | |

# DFT calculations

Geometry optimization was done starting from the geometries from gas-phase exploration done with MACE^18^ at PBE0-D3(BJ)/def2-TZVPP/SMD(THF) level of theory in Gaussian 16C0.2.^19^ The NMR chemical shielding constants of metal hydrides were calculated with relativistic 4-component Dirac-Coulomb Hamiltonian in the ReSpect software.^20^ The exchange-correlation energy was represented by the PBE0 functional.^21^ For these calculations, the Dyall triple-zeta (dyall-cVTZ) basis set was applied to Ru atom,^22,23^ while uncontracted pcS-2 was used for hydride atoms.^24^ The remaining elements were characterized using the uncontracted pc-0 basis set.^25^ Chemical shifts were calculated with respect to shielding of fac-1-H_2_: δ = 42.5 – σ–12.4.

| **Table S16. DFT-calculated optimized 3D structures Ruthenium complexes with SNS Gusev ligand and their predicted NMR chemical shifts.*^[a]^*** | | | |
| --- | --- | --- | --- |
| Entry | Complex | 3D structure | Predicted 𝛿(^1^H), ppm |
| 1 | ***fac*-1-H_2_** |  | -11.9; -12.9 |
| 2 | **1-EtOH** |  | -21.3 |
| 3 | **K-*fac*-1-H_2_** |  | -14.5; -13.8 |
| 4 | ***fac*-1-H_2_-KO*t*Bu** |  | -14.7; -13.4 |
| 5 | ***mer*-1-H_2_** |  | -8.8; -6.9 |
| 6 | **1-H** |  | -27.1 |
| 7 | ***fac*-1-H_2_-EG** |  | -14.0, -13.9 |
| 8 | ***mer*-1-H_2_-EG** |  | -7.9, -9.3 |
| 9 | **1-RuH_4_** |  | -8.0, -9.4  -4.3(central); -4.0(central); |
| 10 | ***mer*-1-EG** |  | -21.5 |
| 11 | ***mer-1-OtBu*** |  | -21.8 |
| 12 | ***fac-1-H_2_-tBuOH*** |  | -13.4 |
| *[a]* Hydrogen atoms on the ligands have been omitted for clarity. Colour scheme: hydrogen (grey), carbon (black), oxygen(red), nitrogen (violet), phosphorus (orange), potassium (blue), ruthenium (pink). | | | |

# X-ray Crystallography

X-ray diffraction data for compound **1_2_-BDM_2_** were collected at 173 K using a Rigaku FR-X Ultrahigh Brilliance Microfocus RA generator/confocal optics with XtaLAB P200 diffractometer [Mo Kα radiation (λ = 0.71073 Å)]. Data were collected (using a calculated strategy) and processed (including correction for Lorentz, polarization and absorption) using CrysAlisPro.^26^ Structure was solved by dual-space methods (SHELXT^27^) and refined by full-matrix least-squares against F^2^ (SHELXL-2019/3^28^). Non-hydrogen atoms were refined anisotropically, and hydrogen atoms were refined using a riding model except for the hydrogen atoms on Ru1, N1, and O31 which were located from the difference Fourier map and refined isotropically subject to distance restraints. All calculations were performed using the Olex2^29^ interface. Selected crystallographic data: C_68_H_88_N_2_O_4_P_2_Ru_2_S_4_, *M* = 1389.72, triclinic, *a* = 10.5110(2), *b* = 12.6296(3), *c* = 13.2346(3) Å, *α* = 84.6640(18), *β* = 71.1833(18), *γ* = 89.4753(17) °, *U* = 1655.39(6) Å^3^, *T* = 173 K, space group *P*$\bar{1}$ (no. 2), *Z* = 1, 36748 reflections collected, 7892 unique (*R*_int_ = 0.0388), which were used in all calculations. The final *R*_1_ [*I* > 2*σ*(*I*)] was 0.0328 and *wR*_2_ (all data) was 0.0752. CCDC 2464903 contains the supplementary crystallographic data for this paper. These data can be obtained free of charge from The Cambridge Crystallographic Data Centre via [www.ccdc.cam.ac.uk/structures](http://www.ccdc.cam.ac.uk/structures).

# References

(1) Hu, Y.; Zhang, S.; Xu, J.; Liu, Y.; Yu, A.; Qian, J.; Xie, Y. Highly Efficient Depolymerization of Waste Polyesters Enabled by Transesterification/Hydrogenation Relay Under Mild Conditions. *Angew. Chemie - Int. Ed.* **2023**, *62* (45), e202312564. https://doi.org/10.1002/anie.202312564.

(2) Zanotti-gerosa, A.; Grainger, D.; Todd, L.; Grasa, G.; Milner, L.; Boddie, E.; Browne, L.; Egerton, I.; Wong, L. The Coming of Age of Homogeneous Ester Hydrogenation Gusev Catalysts : Why , How and When to Use Them. *Chim. Oggi - Chem. Today* **2019**, *37* (4), 8–11.

(3) Anaby, A.; Schelwies, M.; Schwaben, J.; Rominger, F.; Hashmi, A. S. K.; Schaub, T. Study of Precatalyst Degradation Leading to the Discovery of a New Ru0 Precatalyst for Hydrogenation and Dehydrogenation. *Organometallics* **2018**, *37* (13), 2193–2201. https://doi.org/10.1021/acs.organomet.8b00353.

(4) Spasyuk, D.; Smith, S.; Gusev, D. G. Replacing Phosphorus with Sulfur for the Efficient Hydrogenation of Esters. *Angew. Chemie - Int. Ed.* **2013**, *52* (9), 2538–2542. https://doi.org/10.1002/anie.201209218.

(5) Knecht, S.; Hadjiali, S.; Barskiy, D. A.; Pines, A.; Sauer, G.; Kiryutin, A. S.; Ivanov, K. L.; Yurkovskaya, A. V; Buntkowsky, G. Indirect Detection of Short-Lived Hydride Intermediates of Iridium N-Heterocyclic Carbene Complexes via Chemical Exchange Saturation Transfer Spectroscopy. *J. Phys. Chem. C* **2019**, *123* (26), 16288–16293. https://doi.org/10.1021/acs.jpcc.9b04179.

(6) Aguilar, J. A.; Elliott, P. I. P.; López-Serrano, J.; Adams, R. W.; Duckett, S. B. Only Para-Hydrogen Spectroscopy (OPSY), a Technique for the Selective Observation of Para-Hydrogen Enhanced NMR Signals. *Chem. Commun.* **2007**, No. 11, 1183–1185. https://doi.org/10.1039/b616307f.

(7) Liu, Y. F.; Wang, C. L.; Bai, Y. J.; Han, N.; Jiao, J. P.; Qi, X. L. A Facile Total Synthesis of Imatinib Base and Its Analogues. *Org. Process Res. Dev.* **2008**, *12* (3), 490–495. https://doi.org/10.1021/op700270n.

(8) Shang, R.; Fu, Y.; Li, J. Bin; Zhang, S. L.; Guo, Q. X.; Liu, L. Synthesis of Aromatic Esters via Pd-Catalyzed Decarboxylative Coupling of Potassium Oxalate Monoesters with Aryl Bromides and Chlorides. *J. Am. Chem. Soc.* **2009**, *131* (16), 5738–5739. https://doi.org/10.1021/ja900984x.

(9) Weßler, C.; Homann, A.; Fricke, U.; Lehmann, J. NO Donors, Part 8 [1]: Synthesis and Vasodilating Activities of Substituted Benzylnitrates Compared to Cyclohexylmethylnitrate and GTN. *Eur. J. Med. Chem.* **2003**, *38* (6), 581–586. https://doi.org/10.1016/S0223-5234(03)00079-5.

(10) Huang, Z.; Wang, S.; Zhu, X.; Yuan, Q.; Wei, Y.; Zhou, S.; Mu, X. Well-Defined Amidate-Functionalized N-Heterocyclic Carbene -Supported Rare-Earth Metal Complexes as Catalysts for Efficient Hydroboration of Unactivated Imines and Nitriles. *Inorg. Chem.* **2018**, *57* (24), 15069–15078. https://doi.org/10.1021/acs.inorgchem.8b02067.

(11) Singh, A. K.; Bedi, R.; Kaith, B. S. Composite Materials Based on Recycled Polyethylene Terephthalate and Their Properties – A Comprehensive Review. *Composites Part B: Engineering*. Elsevier Ltd 2021, p 108928. https://doi.org/10.1016/j.compositesb.2021.108928.

(12) Fink, J. K. Reactive Polymers: Fundamentals and Applications. *React. Polym. Fundam. Appl. A Concise Guid. to Ind. Polym.* **2017**, 1–687. https://doi.org/10.1016/C2017-0-01641-5.

(13) *ACS PMI Life Cycle Assessment Tool*. https://acsgcipr.org/tools/pmi-life-cycle-assessment/ (accessed 2025-08-07).

(14) Kompella, A.; Bhujanga Rao, A. K. S.; Venkaiah Chowdary, N. Process for the Preparation of the Anti-Cancer Drug Imatinib and Its Analogues. WO 2002108599 A1, 2004.

(15) Rose, H. B.; Kosjek, B.; Armstrong, B. M.; Robaire, S. A. Green and Sustainable Metrics: Charting the Course for Green-by-Design Small Molecule API Synthesis. *Curr. Res. Green Sustain. Chem.* **2022**, *5*, 100324. https://doi.org/10.1016/j.crgsc.2022.100324.

(16) *Home - IPA - International Platinum Group Metals Association*. https://www.ipa-news.com/ (accessed 2025-08-07).

(17) Onken, U.; Koettgen, A.; Scheidat, H.; Schueepp, P.; Gallou, F. Environmental Metrics to Drive a Cultural Change: Our Green Eco-Label. *Chimia (Aarau).* **2019**, *73* (9), 730–736. https://doi.org/10.2533/chimia.2019.730.

(18) Chernyshov, I. Y.; Pidko, E. A. MACE: Automated Assessment of Stereochemistry of Transition Metal Complexes and Its Applications in Computational Catalysis. *J. Chem. Theory Comput.* **2024**, *20* (5), 2313–2320. https://doi.org/10.1021/acs.jctc.3c01313.

(19) Frisch, M. J.; Trucks, G. W.; Schlegel, H. B.; Scuseria. Gaussian 16, Revision A.03; Gaussan, Inc. Wallingford: CT 2016.

(20) Repisky, M.; Komorovsky, S.; Kadek, M.; Konecny, L.; Ekström, U.; Malkin, E.; Kaupp, M.; Ruud, K.; Malkina, O. L.; Malkin, V. G. ReSpect: Relativistic Spectroscopy DFT Program Package. *J. Chem. Phys.* **2020**, *152* (18). https://doi.org/10.1063/5.0005094.

(21) Adamo, C.; Barone, V. Toward Reliable Density Functional Methods without Adjustable Parameters: The PBE0 Model. *J. Chem. Phys.* **1999**, *110* (13), 6158–6170. https://doi.org/10.1063/1.478522.

(22) Dyall, K. G. Relativistic Double-Zeta, Triple-Zeta, and Quadruple-Zeta Basis Sets for the 5d Elements Hf-Hg. *Theor. Chem. Acc.* **2004**, *112* (5–6), 403–409. https://doi.org/10.1007/s00214-004-0607-y.

(23) Dyall, K. G. Relativistic Double-Zeta, Triple-Zeta, and Quadruple-Zeta Basis Sets for the 4d Elements Y-Cd. *Theor. Chem. Acc.* **2007**, *117* (4), 483–489. https://doi.org/10.1007/s00214-006-0174-5.

(24) Jensen, F. Segmented Contracted Basis Sets Optimized for Nuclear Magnetic Shielding. *J. Chem. Theory Comput.* **2015**, *11* (1), 132–138. https://doi.org/10.1021/ct5009526.

(25) Jensen, F. Polarization Consistent Basis Sets: Principles. *J. Chem. Phys.* **2001**, *115* (20), 9113–9125. https://doi.org/10.1063/1.1413524.

1. CrysAlisPro v1.171.43.142a Rigaku Oxford Diffraction, Rigaku Corporation, Tokyo, Japan, **2023**.
2. Sheldrick, G. M. SHELXT – Integrated space-group and crystal structure determination. *Acta Crystallogr., Sect. A: Found. Adv.* **2015**, 71, 3-8. doi: 10.1107/S2053273314026370
3. Sheldrick, G. M. Crystal structure refinement with SHELXL. *Acta Crystallogr., Sect. C: Struct*. Chem. **2015**, 71, 3-8. Doi: 10.1107/S2053229614024218
4. Dolomanov, O. V.; Bourhis, L. J.; Gildea, R. J.; Howard, J. A. K.; Puschmann, H. OLEX2: a complete structure solution, refinement and analysis program. *J. Appl. Crystallogr.* **2009**, 42, 339-341. doi: 10.1107/S0021889808042726
